# Transformation of 5-acylated *N*-fluoroalkyl-1,2,3-triazoles to trifluoromethylated ring-fused isoquinolines, 1,3-oxazines, and 1,3-oxazin-6-ones *via* ketenimines†

**DOI:** 10.1039/d4ra04794j

**Published:** 2024-08-27

**Authors:** Lukáš Janecký, Blanka Klepetářová, Petr Beier

**Affiliations:** a The Institute of Organic Chemistry and Biochemistry of the Czech Academy of Sciences Flemingovo nam. 2 16610 Prague 6 Czech Republic beier@uochb.cas.cz; b Department of Organic Chemistry, Faculty of Science, Charles University Hlavova 2030/8 128 43 Prague 2 Czech Republic

## Abstract

A one-pot multistep methodology leading to trifluoromethylated cyclopenta[*c*]isoquinolines, indeno[1,2-*c*]isoquinolines, 6,6-difluoro-1,3-oxazines, or 1,3-oxazin-6-ones, based on the reaction of 5-acylated *N*-pentafluoroethyl-substituted 1,2,3-triazoles is presented. A thermal ring opening of the starting triazoles, followed by a 1,2-acyl shift formed reactive ketenimines which cyclized after a rearrangement in a substrate-specific manner to provide new trifluoromethylated heterocyclic products.

## Abstract 2

A new synthetic approach towards trifluoromethylated heterocycles, based on the reaction of 5-acylated *N*-fluoroalkyl substituted 1,2,3-triazoles under microwave heating, proceeding *via* ketenimines is presented.

## Graphical abstract

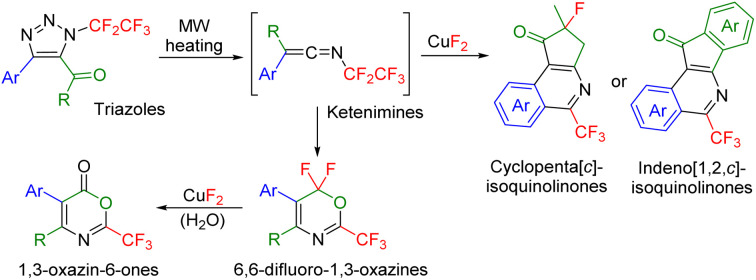

## Introduction

**Fig. 1:**
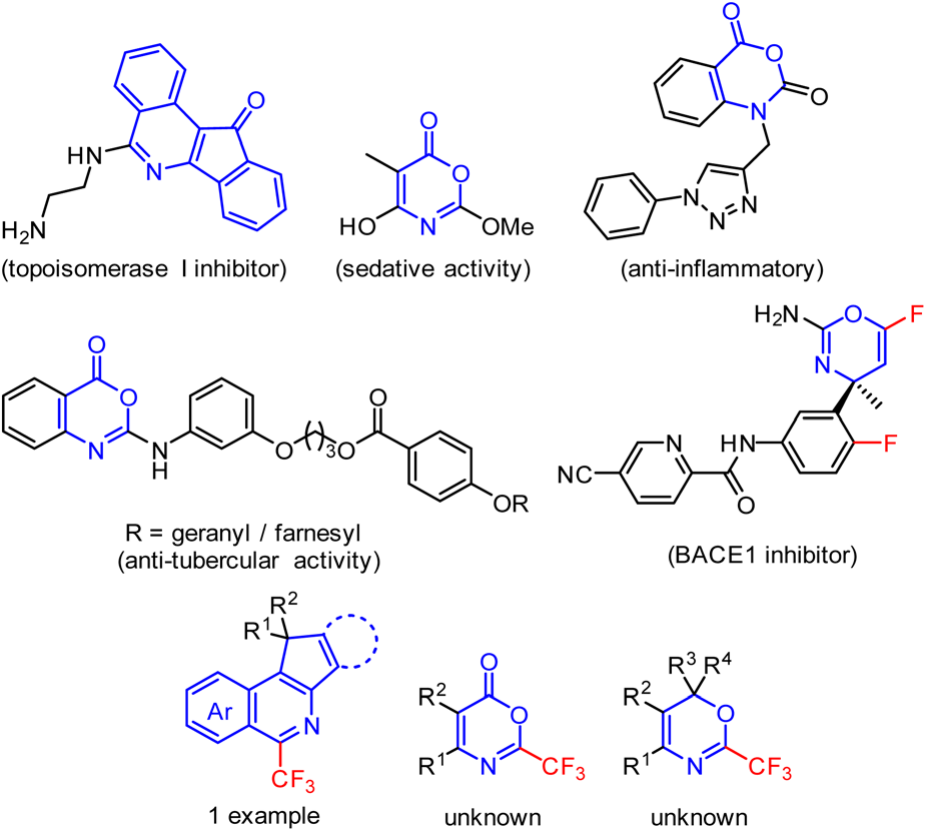
Selected examples of bioactive 5-membered ring fused isoquinolines or 1,3-oxazine and 1,3-oxazin-6-ones and compounds of interest – their trifluoromethylated derivatives.

Isoquinolines with fused 5-membered rings, 6*H*-1,3-oxazines, or oxazin-6-ones constitute important classes of biologically active compounds known as anti-tubercular, anti-inflammatory, sedative agents, or enzyme inhibitors (Fig. 1).^1–5^ Despite the few known synthetic strategies to indeno[1,2-*c*]isoquinolines^1,6–8^ or cyclopenta[*c*]isoquinolines,^9^ the preparation of 2-trifluoromethyl-5-membered ring-fused isoquinolines was described for only one specific example.^10^ Similarly, non-fluorinated fully substituted 6*H*-1,3-oxazin-6-ones can be synthesized from β-lactams,^11^ isoxazolones,^12–14^ cyclopropenones^15^ or ynamides.^16^ However, 2-trifluoromethyl-substituted 1,3-oxazin-6-ones or 1,3-oxazines remain unexplored (Fig. 1). Since trifluoromethylated heteroaromatics of novel structures are highly valued chemicals, which find use in medicinal chemistry^17–20^ and agrochemistry^21–23^ research programmes, we set out to investigate the synthetic approaches towards the proposed novel trifluoromethylated heteroarenes shown in Fig. 1.

**Scheme 1:**
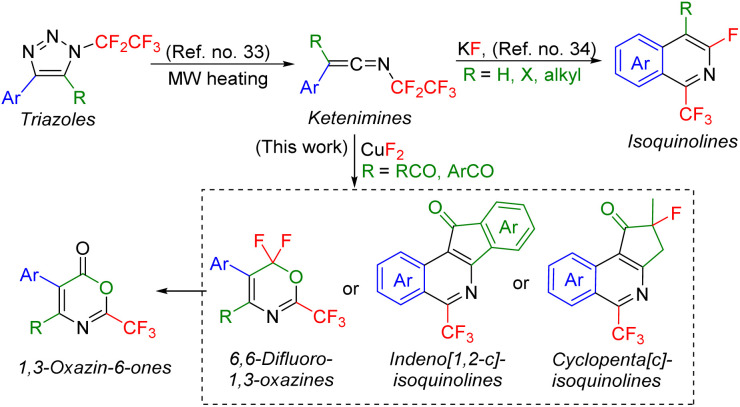
Microwave-assisted transformation of *N*-pentafluoroethyl-1,2,3-triazoles to trifluoromethylated isoquinolines *via* ketenimines (previous work) or to (fused)isoquinolines, 1,3-oxazines, or 1,3-oxazin-6-ones (this work).

We recently reported a denitrogenation strategy for multisubstituted *N*-fluoroalkylated 1,2,3-triazoles^24–31^ with Brønsted or Lewis acids proceeding *via* vinyl cation intermediates and leading to various *N*-alkenyl compounds.^10,24,30–32^ We also showed that *N*-fluoroalkyl 1,2,3-triazoles in microwave reaction conditions undergo a rearrangement to form ketenimines,^33^ which can further cyclize to isoquinolines (Scheme 1).^34^

Herein, we propose a new synthetic methodology to prepare trifluoromethylated 5-membered ring fused isoquinolines, 6,6-difluoro-1,3-oxazines or 1,3-oxazin-6-ones from 5-acyl-*N*-pentafluoroethyl-1,2,3-triazoles involving ketenimine intermediates (Scheme 1).

## Results and discussion

Denitrogenation of *N*-fluoroalkylated 1,2,3-triazoles to ketenimines by microwave heating^33^ was extended to 5-acylated triazoles.^24^ Thus, microwave heating of 5-methacryloyl-substituted triazole 1a resulted in the formation of a mixture of ring-fused 1-trifluoromethylisoquinolines 2a and 3a, presumably *via* ketenimine A, imidoyl fluoride B and isoquinoline C intermediates (Table 1, entry 1). The addition of fluoride salts can enhance the 1,3-fluorine shift of A to B, therefore an optimization study was conducted to improve the selectivity of the reaction. Copper(ii) fluoride was identified as the most effective fluoride additive (entry 8). A combination of potassium fluoride and sodium hydroxide was used to obtain dehydrofluorinated 1-trifluoromethyl-isoquinoline 3a (entry 9).

**Table 1:** Optimization of the reaction conditions leading to cyclopenta[*c*]isoquinolines 2a and 3a from 5-acylated triazole 1a

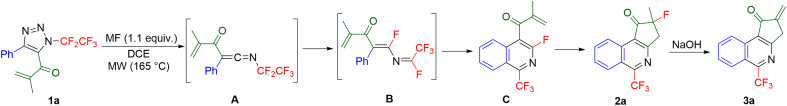					
Entry	Time (min)	Additive	Ratio 2a/3a^a^	2a Yield^b^ (%)	3a Yield^b^ (%)
1	120	—	48 : 52	n.d.	n.d.
2	60	KF	27 : 73	12	23
3	60	AlF_3_	37 : 63	n.d.	n.d.
4	60	CsF	42 : 58	n.d.	n.d.
5	60	AgF	78 : 22	42	10
6	60	NaF	54 : 46	n.d.	n.d.
7	60	FeF_3_	38 : 62	n.d.	n.d.
8	30	CuF_2_	84 : 16	41	12
9	30	KF^c^	13 : 87	Traces	31

a ^19^F NMR ratio.

b Isolated yield. n.d. not determined.

c With added NaOH (3 equiv.).

**Scheme 2:**
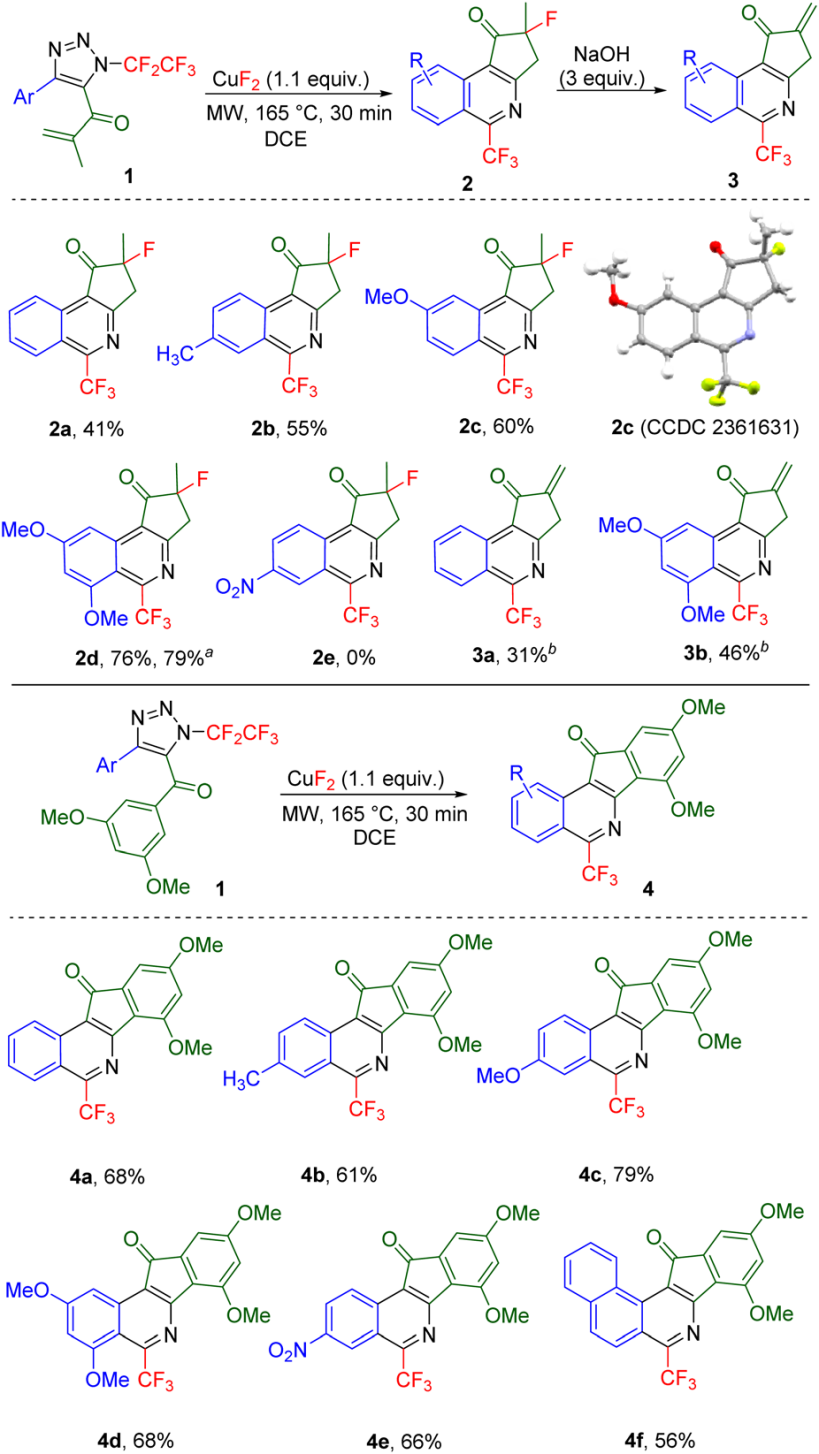
Scope of products of the microwave-assisted transformation of 5-acyl-*N*-pentafluoroethylated 1,2,3-triazoles 1 (0.1–0.2 mmol) to cyclopenta[*c*]isoquinolines 2 and 3 or indeno[1,2-*c*]-isoquinolines 4. ^*a*^2.11 mmol scale. ^*b*^Using KF (1.1 equiv.) and NaOH (3 equiv.) instead of CuF_2_.

A small library of 4-aryl-5-methacryloyl triazoles, obtained from the intercepted click reaction of aromatic copper(i) acetylides, azidopentafluoroethane and methacrylic chloride in the presence of DIPEA (see ESI† for details), was subjected to the reaction providing ring-fused isoquinolines 2 in moderate to good yields (Scheme 2). The structure of derivative 2c was confirmed by crystallography. Substrate with electron-acceptor group (nitro) on the aryl ring did not form the product (2e). Additionally, two examples of dehydrofluorinated isoquinolines 3 were prepared albeit in moderate to low yields.

When electron-rich 5-(3,5-dimethoxybenzoyl)-substituted 1,2,3-triazoles were used, ring-fused 1-trifluoromethyl-isoquinolines 4 bearing various substituents on the isoquinoline ring formed in good yields (Scheme 2).

**Scheme 3:**
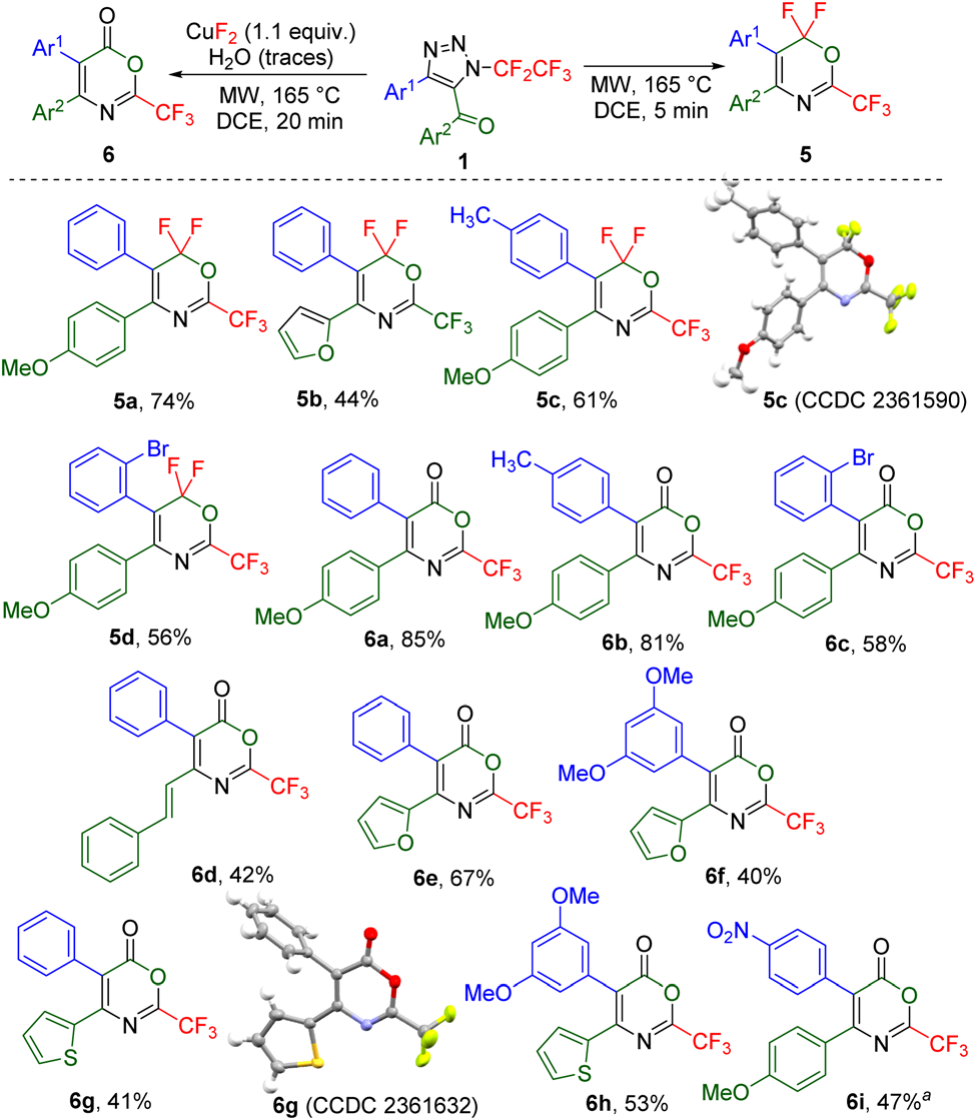
Scope of products of the microwave-assisted transformation of 5-acyl-*N*-pentafluoroethyl-1,2,3-triazoles 1 (0.1–0.25 mmol) to trifluoromethylated 6,6-difluoro-1,3-oxazines 5 and 1,3-oxazin-6-ones 6. ^*a*^195 °C, 120 min.

All other investigated 5-acylated 1,2,3-triazoles except strongly electron-rich 5-(3,5-dimethoxybenzoyl)- or 5-methacryloyl-substituted ones afforded different products under the thermal denitrogenation conditions. Thus, 5-(4-methoxyphenyl)-substituted triazole underwent a unique transformation presumably *via* ketenimine D, followed by 1,3-aryl group transfer to ketene E,^35^ 1,5-fluorine shift to intermediate F, and cyclization involving another 1,5-fluorine shift to 6,6-difluoro-2-trifluoromethyl-1,3-oxazine 5a or a product of its hydrolysis 1,3-oxazin-6-one 6a (Table 2). Short reaction time (5 min) and no additive favoured the formation of product 5a, while a longer reaction time (20 min) and the use of CuF_2_ favoured the product of hydrolysis 6a. Four examples of 1,3-oxazines 5 were prepared in moderate to good yields, including the crystal structure of 5c and nine examples of 1,3-oxazinones 6 were synthesized in moderate to high yields including the crystal structure of 6g (Scheme 3). While the presence of an alkenyl group led to oxazinone 6d with this substitution in position 4, the products with alkyl groups in position 4 or 5 or an alkenyl group in position 5 did not form. Also, products 6 with the difluoromethyl or ethoxycarbonyl groups in position 2 did not form.

**Table 2:** Optimization of the reaction conditions leading to 6,6-difluoro-1,3-oxazine 5a and 1,3-oxazinone 6a

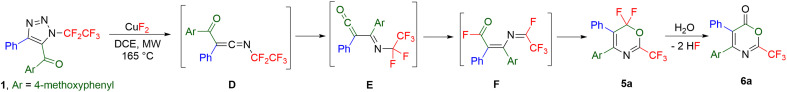					
Entry	CuF_2_ (equiv.)	Reaction time (min)	Ratio 5a/6a^a^	5a Yield^b^ (%)	6a Yield^b^ (%)
1	0	20	78 : 22	28	n.d.
2	0	5	92 : 8	60	n.d.
3	1.1	10	8 : 92	n.d.	39
4	1.1	20	1 : 99	n.d.	85

a ^19^F NMR ratio.

b Isolated yield. n.d. not determined.

## Conclusions

In conclusion, thermal denitrogenation of *N*-pentafluoroethylated 4-substituted-5-acyl-1,2,3-triazoles in the presence of copper(ii) fluoride affords depending on the nature of 5-acyl substitution 1-trifluoromethylcyclopenta[*c*]-isoquinolines, indeno[1,2-*c*]-isoquinolines, 2-trifluoromethyl-6,6-difluoro-1,3-oxazines, or products of their hydrolysis 2-trifluoromethyl-1,3-oxazin-6-ones. All these compounds result from the formation of ketenimine intermediates which undergo either 1,3-fluorine shift, S_E_Ar and S_N_Ar sequence, or 1,3-aryl shift, 1,5-fluorine shift, cyclization and another 1,5-fluorine shift sequence. The presented methodology showcases advanced cyclization of ketenimine intermediates generated from triazoles and their application in the C–C bond formation for the synthesis of new heterocyclic structures.

## Data availability

The data supporting this article have been included as part of the ESI.†

## Author contributions

PB supervised the project. LJ contributed to experiments and product characterization. BK solved the crystal structures. LJ and PB jointly conceived the project, prepared the manuscript, and contributed to discussions.

## Conflicts of interest

There are no conflicts to declare.

## Supplementary Material

RA-014-D4RA04794J-s001

RA-014-D4RA04794J-s002
