# Supplementary material for: Transformation of 5-acylated N-fluoroalkyl-1,2,3-triazoles to trifluoromethylated ring-fused isoquinolines, 1,3-oxazines, and 1,3-oxazin-6-ones via ketenimines
Source: RSC Adv. 2024 Aug 27;14(37):26938–42. doi: 10.1039/d4ra04794j (PMC11348844; doi:10.1039/d4ra04794j)

Supplementary Information (SI) for RSC Advances.
This journal is © The Royal Society of Chemistry 2024

Transformation of 5-Acylated *N*-Fluoroalkyl-1,2,3-Triazoles to Trifluoromethylated Ring-Fused Isoquinolines, 1,3-Oxazines, and 1,3-Oxazin-6-ones via Ketenimines

Lukáš Janecký[a,b], Blanka Klepetářová[a], Petr Beier[a,*]

[a]Institute of Organic Chemistry and Biochemistry, Academy of Sciences of the Czech Republic, Flemingovo nám. 2, 166 10 Prague, Czech Republic
[b]Department of Organic Chemistry, Faculty of Science, Charles University, Hlavova 2030/8, CZ-128 43 Prague 2, Czech Republic

*Corresponding author: beier@uochb.cas.cz

Supporting information

Contents

General information S2
X-ray crystallography S3
Crystal data for **2c** S3
Crystal data for **5c** S3
Crystal data for **6g** S4
Synthesis of previously unpublished triazoles **1** S5
General procedure: S5
Characterization of triazoles **1** S5
Synthesis of cyclopenta[*c*]isoquinolines **2** and **3** S11
General procedure: S11
Characterization of cyclopenta[*c*]isoquinolines **2** and **3** S11
Synthesis of indeno[1,2-*c*]isoquinolines **4** S13
General procedure: S13
Characterization of indeno[1,2-*c*]isoquinolines **4** S13

Synthesis of 6,6-difluoro-1,3-oxazines **5** S15
General procedure: S15
Characterization of indeno[1,2-*c*]isoquinolines **5** S15
Synthesis of 1,3-oxazin-6-ones **6** S16
General procedure: S16
Characterization of 1,3-oxazin-6-ones **6** S16
References S18
Copies of NMR spectra S19

General information

All commercially available chemicals were used as received unless stated otherwise. Flash column chromatography was performed using silica gel 60 (0.040–0.063 mm). Automated flash column chromatography was performed on Teledyne ISCO CombiFlash Rf$^+$ Lumen Automated Flash Chromatography System with UV/Vis detection. ^{1}H, ^{13}C, and ^{19}F NMR spectra were measured at ambient temperature using 5 mm diameter NMR tubes. ^{13}C NMR spectra were proton decoupled. The chemical shift values (*δ*) are reported in ppm relative to internal Me_4Si (0 ppm for ^{1}H and ^{13}C NMR) or residual solvents and internal $CFCl_3$ (0 ppm for ^{19}F NMR). Coupling constants (*J*) are reported in Hertz. Structural elucidation was aided by additional acquisition of various 2D NMR spectra (^{1}H-^{1}H COSY, ^{1}H-^{13}C HSQC, ^{1}H-^{13}C HMBC). High resolution mass spectra (HRMS) were recorded on a Waters Micromass AutoSpec Ultima or Agilent 7890A GC coupled with Waters GCT Premier orthogonal acceleration time-of-flight detector using electron impact (EI) or chemical ionization (CI), on an LTQ Orbitrap XL using electrospray ionization (ESI), Q-Tof micro (Waters) is a quadrupole orthogonal acceleration time-of-flight tandem mass spectrometer using atmospheric-pressure chemical ionization (APCI), and on a Bruker solariX 94 ESI/MALDI-FT-ICR using dual ESI/MALDI ionization. Microwave experiments were done on CEM Focused Microwave™ Synthesis System, Model Discover. The method was set-up to 300 W, temperature 165-195 °C, hold time 5-120 min.

X-ray crystallography

Single-crystal diffraction data of **2c**, **5c** and **6g** were collected at 180 K using Bruker D8 VENTURE system equipped with a Photon 100 CMOS detector, a multilayer monochromator, and a CuKα Incoatec microfocus sealed tube (λ = 1.54178 Å). The frames were integrated with the Bruker SAINT[1] software package. The structure was solved by direct methods with SIR92[2] (**2c** and **5c**) or by charge-flipping methods using Superflip[3] (**6g**) and refined by full-matrix least-squares on F^2 with CRYSTALS.[4] The positional and anisotropic thermal parameters of all non-hydrogen atoms were refined. All hydrogen atoms were located in a difference Fourier map, but repositioned geometrically and then refined with riding constraints.

Crystal data for **2c** (light yellow, 0.046 x 0.053 x 0.358 mm):

$C_{15}H_{11}F_4NO_2$, triclinic, space group *P*-1, *a* = 4.8502(2) Å, *b* = 10.1006(3) Å, *c* = 14.4402(5) Å, α = 109.3028(12)°, β = 97.3122(13)°, γ = 95.9100(13)°, *V* = 654.23(4) Å^3, *Z* = 2, *M* = 313.25, 10818 reflections measured, 2461 independent reflections. Final *R* = 0.0335, *wR* = 0.0929, *GoF* = 0.9780 for 2292 reflections with $I > 2\sigma(I)$ and 199 parameters. CCDC 2361631.

Figure S1 ORTEP diagram of **2c.** Displacement ellipsoids are drawn at the 50% probability level.

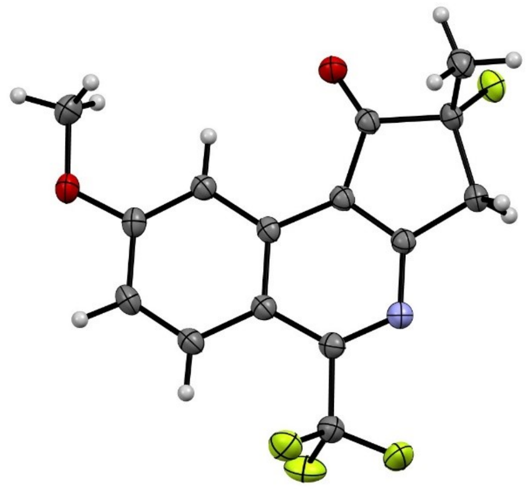

Crystal data for **5c** (light yellow, 0.080 x 0.127 x 0.516 mm):

$C_{19}H_{14}F_5NO_2$, orthorhombic, space group *Pbca*, *a* = 18.7156(7) Å, *b* = 8.5123(3) Å, *c* = 21.5158(9) Å, *V* = 3427.7(2) Å^3, *Z* = 8, *M* = 383.32, 46820 reflections measured, 3381 independent reflections. Final *R* = 0.0398, *wR* = 0.1033, *GoF* = 0.9901 for 2934 reflections with $I > 2\sigma(I)$ and 245 parameters. CCDC 2361590.

Figure S2. ORTEP diagram of **5c.** Displacement ellipsoids are drawn at the 50% probability level.

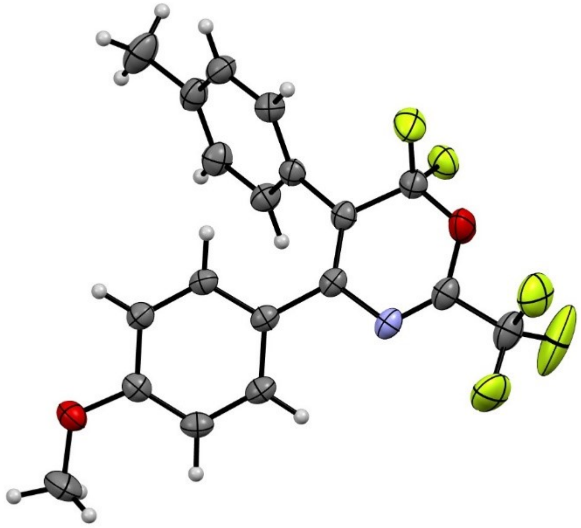

Crystal data for **6g** (colorless, 0.030 x 0.516 x 0.572 mm):

$C_{15}H_8F_3NO_2S$, monoclinic, space group $P2_1/c$, a = 9.1250(3) Å, b = 14.3039(5) Å, c = 11.2617(4) Å, β = 109.1605(11)°, V = 1388.48(9) Å^3, Z = 4, M = 323.29, 32093 reflections measured, 2637 independent reflections. Final R = 0.0284, wR = 0.0719, GoF = 0.9336 for 2497 reflections with I > 2σ(I) and 218 parameters. The thiophene ring displays substitutional disorder of sulphur and carbon atoms with occupancy ratios of 0.827(2) and 0.173(2), the original positions are rotated around the bonding axis by 180°. CCDC 2361632.

Figure S3. ORTEP diagram of **6g.** Displacement ellipsoids are drawn at the 50% probability level. Only the more occupied part of the disordered group is shown for clarity.

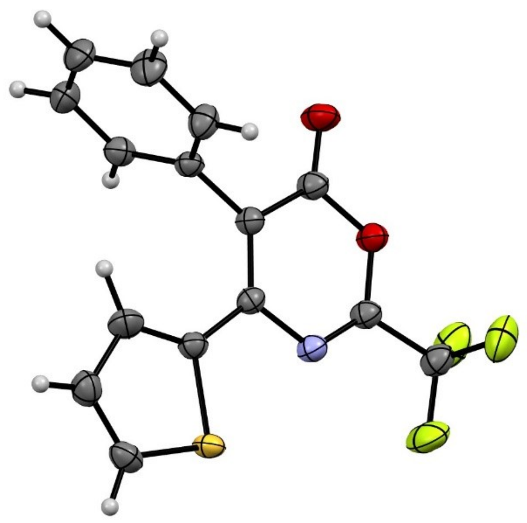

Synthesis of previously unpublished triazoles **1**

General procedure:

Under air atmosphere, a 10 mL screw-cap glass reaction tube was charged with the corresponding copper(I) acetylide (1.0 mmol, 1.0 equiv.), 240 mg 3Å molecular sieves and a stirring bar. Then it was cooled to 0 °C, followed by the addition of a solution of azide in THF (~1.5 mmol, 4 mL), EDIPA (523 µl, 3.0 mmol, 3.0 equiv.) and the corresponding acyl chloride (2.0 mmol, 2.0 equiv.). The reaction mixture was warmed to room temperature and stirred for 16-20 h. The crude suspension was filtered via a short plug of silica gel and washed by THF (3 x 10 mL). The filtrate was evaporated with Celite (water bath 40 °C) and purified by column chromatography (cyclohexane/EtOAc or pentane/toluene) to obtain pure triazole **1**.

Characterization of triazoles **1**

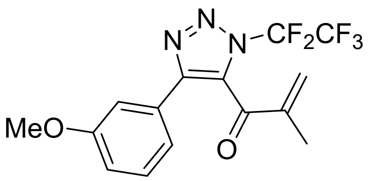

*1-(4-(3-Methoxyphenyl)-1-(perfluoroethyl)-1H-1,2,3-triazol-5-yl)-2-methylprop-2-en-1-one (***1a***):*

Prepared according to the general procedure. Yield: 318.3 mg colorless oil; 71%; ^{1}H NMR (401 MHz, $CDCl_3$) δ 7.31 (t, *J* = 7.9 Hz, 1H), 7.22 (dd, *J* = 2.6, 1.5 Hz, 1H), 7.11 (ddd, *J* = 7.7, 1.7, 1.0 Hz, 1H), 6.94 (ddd, *J* = 8.3, 2.6, 1.0 Hz, 1H), 6.07 (q, *J* = 1.5 Hz, 1H), 5.60 (t, *J* = 1.0 Hz, 1H), 3.81 (s, 3H), 2.04 (dd, *J* = 1.5, 0.9 Hz, 3H); ^{13}C NMR (101 MHz, $CDCl_3$) δ 187.7, 160.1, 146.4, 144.5, 133.0, 130.5 (d, *J* = 1.8 Hz), 130.2, 129.2, 119.9, 117.0 (dt, *J* = 287.9, 40.0 Hz), 115.8, 112.7, 110.9 (q, *J* = 43.5 Hz), 55.4, 16.6; ^{19}F NMR (377

MHz, $CDCl_3$) δ –82.7 (s, 3F), –94.9 (s, 2F); HRMS (ESI^+) *m/z* calcd for $C_{15}H_{13}F_5N_3O_2$ $[M+H]^+$: 362.09224, found 362.09220.

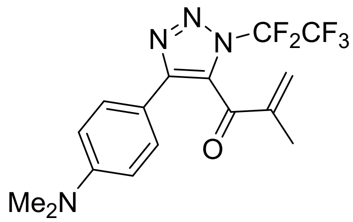

*1-(4-(4-(Dimethylamino)phenyl)-1-(perfluoroethyl)-1H-1,2,3-triazol-5-yl)-2-methylprop-2-en-1-one (***1b***):*

Prepared according to the general procedure. Yield: 193.0 mg yellow solid; 52%; m.p. 82 – 84 °C; ^{1}H NMR (400 MHz, $CDCl_3$) δ 7.50–7.46 (m, 2H), 6.72–6.68 (m, 2H), 6.03 (q, *J* = 1.5 Hz, 1H), 5.63 (q, *J* = 0.9 Hz, 1H), 2.99 (s, 6H), 2.05 (dd, *J* = 1.6, 0.9 Hz, 3H); ^{13}C NMR (101 MHz, $CDCl_3$) δ 188.2, 151.0, 147.2, 144.3, 132.5, 128.5, 128.2 (t, *J* = 2.0 Hz), 117.0 (qt, *J* = 287.9, 40.3 Hz), 115.2, 112.0, 110.8 (tq, *J* = 271.4, 43.2 Hz), 40.1, 16.5; ^{19}F NMR (376 MHz, $CDCl_3$) δ –82.6 (s, 3F), –94.8 (s, 2F); HRMS (ESI^+) *m/z* calcd for $C_{16}H_{16}F_5N_4O$ $[M+H]^+$: 375.12388, found 375.12394.

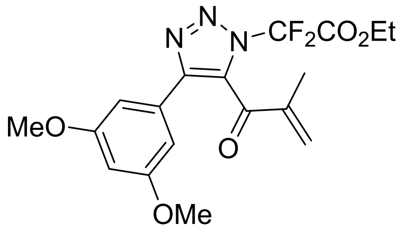

*Ethyl 2-(4-(3,5-dimethoxyphenyl)-5-methacryloyl-1H-1,2,3-triazol-1-yl)-2,2-difluoroacetate (***1c***):*

Prepared according to the general procedure. Yield: 301.1 mg pale yellow oil; 38%; ^{1}H NMR (401 MHz, $CDCl_3$) δ 6.74 (d, *J* = 2.3 Hz, 2H), 6.48 (t, *J* = 2.3 Hz, 1H), 6.05 (q, *J* = 1.5 Hz, 1H), 5.67 (q, *J* = 0.9 Hz, 1H), 4.51 (q, *J* = 7.1 Hz, 2H), 3.79 (s, 6H), 2.05–2.04 (m, 3H), 1.41 (t, *J* = 7.2 Hz, 3H); ^{13}C NMR (101 MHz, $CDCl_3$) δ 188.0, 161.2, 158.2 (t, *J* = 33.9 Hz), 146.9, 144.2, 132.8, 130.1, 129.8 (t, *J* = 1.8 Hz), 110.2 (t, *J* = 269.2 Hz), 105.8, 102.0, 65.2, 55.6, 16.7, 13.9; ^{19}F NMR (376 MHz, $CDCl_3$) δ –85.7 (s); HRMS (ESI^+) *m/z* calcd for $C_{18}H_{20}F_2N_3O_5$ $[M+H]^+$: 396.13655, found 396.13633.

*1-(4-(3,5-Dimethoxyphenyl)-1-(1,1,2,2-tetrafluoroethyl)-1H-1,2,3-triazol-5-yl)-2-methylprop-2-en-1-one (***1d***):*

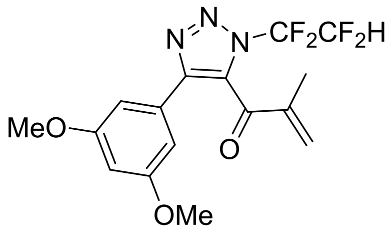

Prepared according to the general procedure. Yield: 192.5 mg pale yellow oil; 52%; ^{1}H NMR (401 MHz, $CDCl_3$) δ 6.75 (d, *J* = 2.2 Hz, 2H), 6.74 (tt, *J* = 52.2, 5.1 Hz, 1H); 6.49 (t, *J* = 2.3 Hz, 1H), 6.08 (q, *J* = 1.5 Hz, 1H), 5.62 (q, *J* = 0.9 Hz, 1H), 3.79 (s, 6H), 2.06 (dd, *J* = 1.5, 0.9 Hz, 3H); ^{13}C NMR (101 MHz, $CDCl_3$) δ 188.0, 161.3, 146.6, 144.5, 133.0, 130.3, 129.8, 112.9 (tt, *J* = 268.5, 29.3 Hz), 107.8 (tt, *J* = 254.5, 33.7 Hz), 105.6, 102.1, 55.6, 16.6; ^{19}F NMR (377 MHz, $CDCl_3$) δ –97.4 (q, *J* = 8.9, 8.2 Hz, 2F), –137.8 (dt, *J* = 52.6, 8.4 Hz, 2F); HRMS (ESI^+) *m/z* calcd for $C_{16}H_{16}F_4N_3O_3$ $[M+H]^+$: 374.11223, found 374.11229.

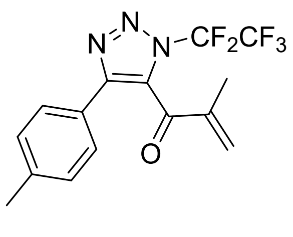

*2-Methyl-1-(1-(perfluoroethyl)-4-(p-tolyl)-1H-1,2,3-triazol-5-yl)prop-2-en-1-one (***1e***):*

Prepared according to the general procedure. Yield: 226.7 mg pale yellow oil; 53%; ^{1}H NMR (401 MHz, $CDCl_3$) δ 7.52–7.48 (m, 2H), 7.24–7.21 (m, 2H), 6.05

(q, J = 1.5 Hz, 1H), 5.60 (q, J = 1.0 Hz, 1H), 2.37 (s, 3H), 2.04 (dd, J = 1.5, 1.0 Hz, 3H); ^{13}C NMR (101 MHz, $CDCl_3$) δ 187.9, 146.7, 144.5, 140.0, 132.9, 129.9 (t, J = 1.8 Hz), 129.9, 127.5, 125.1, 117.1 (qt, J = 287.9, 40.0 Hz), 110.9 (tq, J = 271.9, 43.5 Hz), 21.4, 16.6; ^{19}F NMR (377 MHz, $CDCl_3$) δ –82.7 (s, 3F), –94.9 (s, 2F); HRMS (ESI$^+$) *m/z* calcd for $C_{15}H_{13}F_5N_3O$ [M+H]$^+$: 346.09733, found 346.09737.

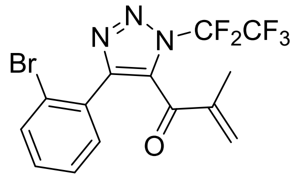

*1-(4-(2-Bromophenyl)-1-(perfluoroethyl)-1H-1,2,3-triazol-5-yl)-2-methylprop-2-en-1-one (***1f***):*

Prepared according to the general procedure. After column chromatography (cyclohexane/EtOAc), the pure product was obtained as by crystallization from pentane/Et_2O. Yield: 274.0 mg white solid; 53%; m.p. 67 – 69 °C; ^{1}H NMR (401 MHz, $CDCl_3$) δ 7.70–7.63 (m, 1H), 7.39–7.29 (m, 3H), 5.93 (q, J = 1.5 Hz, 1H), 5.59 (q, J = 1.0 Hz, 1H), 1.90 (s, 3H); ^{13}C NMR (101 MHz, $CDCl_3$) δ 185.8, 146.7, 144.2, 133.6, 132.7 (t, J = 1.8 Hz), 132.4, 132.2, 131.5, 129.5, 127.8, 123.8, 117.1 (qt, J = 288.3, 39.6 Hz), 111.0 (tq, J = 271.9, 43.3 Hz), 16.6; ^{19}F NMR (377 MHz, $CDCl_3$) δ –82.3 (s, 3F), –94.5 (s, 2F); HRMS (ESI$^+$) *m/z* calcd for $C_{14}H_{10}F_5BrN_3O$ [M+H]$^+$: 409.99219, found 409.99224.

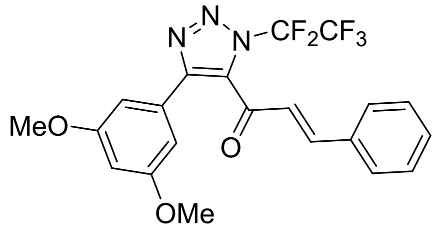

*(E)-1-(4-(3,5-Dimethoxyphenyl)-1-(perfluoroethyl)-1H-1,2,3-triazol-5-yl)-3-phenylprop-2-en-1-one (***1g***):*

Prepared according to the general procedure. Yield: 423.0 mg yellow oil; 75%; ^{1}H NMR (401 MHz, $CDCl_3$) δ 7.46–7.41 (m, 4H), 7.40–7.35 (m, 2H), 6.95 (d, J = 16.1 Hz, 1H), 6.88 (d, J = 2.3 Hz, 2H), 6.50 (t, J = 2.3 Hz, 1H), 3.76 (s, 6H); ^{13}C NMR (101 MHz, $CDCl_3$) δ 183.8, 161.3, 149.4, 146.9, 133.3, 132.2, 131.5 (t, J = 1.8 Hz), 129.6, 129.3, 129.1, 125.5, 117.1 (qt, J = 287.9, 39.6 Hz), 111.0 (tq, J = 272.5, 43.1 Hz), 106.1, 102.3, 55.6; ^{19}F NMR (377 MHz, $CDCl_3$) δ –82.2 (s, 3F), –94.4 (s, 2F); HRMS (ESI$^+$) *m/z* calcd for $C_{21}H_{17}F_5N_3O_3$ [M+H]$^+$: 454.11846, found 454.11837.

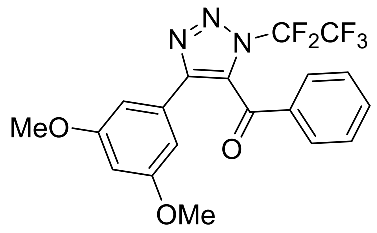

*(4-(3,5-Dimethoxyphenyl)-1-(perfluoroethyl)-1H-1,2,3-triazol-5-yl)(phenyl)methanone (***1h***):*

Prepared according to the general procedure. Yield: 343.4 mg pale yellow oil; 64%; ^{1}H NMR (400 MHz, $CDCl_3$) δ 7.79–7.76 (m, 2H), 7.65–7.60 (m, 1H), 7.48–7.43 (m, 2H), 6.76 (d, J = 2.3 Hz, 2H), 6.39 (t, J = 2.3 Hz, 1H), 3.64 (s, 6H); ^{13}C NMR (101 MHz, $CDCl_3$) δ 185.7, 161.2, 146.6, 135.7, 135.2, 130.7 (t, J = 1.7 Hz), 129.8, 129.4, 129.4, 117.0 (qt, J = 287.9, 40.0 Hz), 110.9 (tq, J = 272.5, 43.3 Hz), 105.6, 102.4, 55.4; ^{19}F NMR (376 MHz, $CDCl_3$) δ –82.6 (s, 3F), –94.5 (s, 2F); HRMS (ESI$^+$) *m/z* calcd for $C_{19}H_{15}F_5N_3O_3$ [M+H]$^+$: 428.10281, found 428.10270.

*(4-Methoxyphenyl)(4-(4-nitrophenyl)-1-(perfluoroethyl)-1H-1,2,3-triazol-5-yl)methanone (***1i***):*

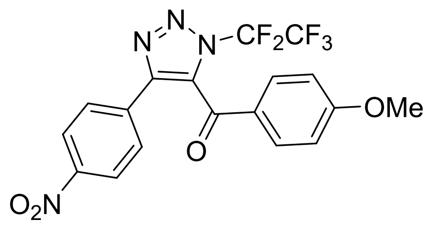

Prepared according to the general procedure. Yield: 170.0 mg colorless oil; 31%; ^{1}H NMR (401 MHz, $CDCl_3$) δ 8.17 (d, *J* = 8.9 Hz, 2H), 7.84 (d, *J* = 8.9 Hz, 2H), 7.73 (d, *J* = 8.6 Hz, 2H), 6.93 (d, *J* = 8.9 Hz, 2H), 3.86 (s, 3H); ^{13}C NMR (101 MHz, $CDCl_3$) δ 183.1, 166.1, 148.2, 143.9, 134.1, 132.4 (2C), 128.2, 127.9, 124.3, 116.9 (qt, *J* = 288.3, 39.7 Hz), 115.0, 110.9 (tq, *J* = 273.5, 43.6 Hz), 55.9; ^{19}F NMR (377 MHz, $CDCl_3$) δ –82.6 (s, 3F), –94.8 (s, 2F); HRMS (APCI$^+$) *m/z* calcd for $C_{18}H_{12}F_5N_4O_4$ [M+H]$^+$: 443.07732, found 443.07684.

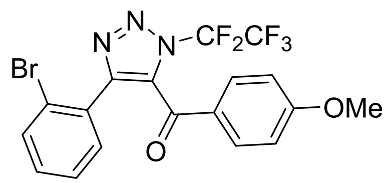

*(4-(2-Bromophenyl)-1-(perfluoroethyl)-1H-1,2,3-triazol-5-yl)(4-methoxyphenyl)methanone (***1j***):*

Prepared according to the general procedure. Yield: 232.3 mg pale yellow oil; 49%; ^{1}H NMR (400 MHz, $CDCl_3$) δ 7.71–7.67 (m, 2H), 7.57 (ddd, *J* = 7.9, 1.3, 0.5 Hz, 1H), 7.34 (ddd, *J* = 7.7, 2.0, 0.6 Hz, 1H), 7.28 (td, *J* = 7.5, 1.3 Hz, 1H), 7.20 (ddd, *J* = 7.9, 7.4, 1.8 Hz, 1H), 6.85–6.81 (m, 2H), 3.83 (s, 3H); ^{13}C NMR (101 MHz, $CDCl_3$) δ 182.3, 165.3, 146.6, 133.5, 133.2 (d, *J* = 2.2 Hz), 132.5, 132.3, 131.3, 129.2, 128.2, 127.6, 123.7, 117.2 (qt, *J* = 288.3, 39.6 Hz), 114.3, 111.0 (tq, *J* = 272.3, 43.3 Hz), 55.8; ^{19}F NMR (376 MHz, $CDCl_3$) δ –82.3 (s, 3F), –94.4 (s, 2F); HRMS (ESI$^+$) *m/z* calcd for $C_{18}H_{12}F_5BrN_3O_2$ [M+H]$^+$: 476.00276, found 476.00287.

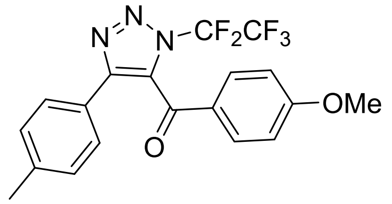

*(4-Methoxyphenyl)(1-(perfluoroethyl)-4-(p-tolyl)-1H-1,2,3-triazol-5-yl)methanone (***1k***):*

Prepared according to the general procedure. Yield: 317.0 mg pale yellow oil; 77%; ^{1}H NMR (401 MHz, $CDCl_3$) δ 7.75–7.70 (m, 2H), 7.55–7.51 (m, 2H), 7.14–7.10 (m, 2H), 6.92–6.87 (m, 2H), 3.85 (s, 3H), 2.31 (s, 3H); ^{13}C NMR (101 MHz, $CDCl_3$) δ 184.2, 165.6, 146.5, 139.7, 132.5, 130.4 (t, *J* = 1.9 Hz), 129.8, 128.4, 127.5, 125.1, 117.1 (qt, *J* = 287.9, 40.0 Hz), 114.7, 111.0 (tq, *J* = 271.9, 43.3 Hz), 55.8, 21.4; ^{19}F NMR (376 MHz, $CDCl_3$) δ –82.3 (s, 3F), –94.4 (s, 2F); HRMS (ESI$^+$) *m/z* calcd for $C_{19}H_{15}F_5N_3O_2$ [M+H]$^+$: 412.10789, found 412.10785.

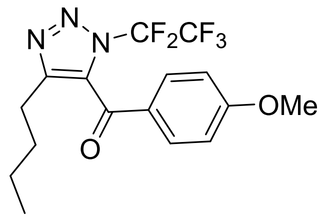

*(4-Butyl-1-(perfluoroethyl)-1H-1,2,3-triazol-5-yl)(4-methoxyphenyl)methanone (***1l***):*

Prepared according to the general procedure. Yield: 167.1 mg colorless oil; 35%; ^{1}H NMR (401 MHz, $CDCl_3$) δ 7.76–7.72 (m, 2H), 7.01–6.97 (m, 2H), 3.91 (s, 3H), 2.60–2.56 (m, 2H), 1.66–1.59 (m, 2H), 1.30–1.22 (m, 2H), 0.82 (t, *J* = 7.3 Hz, 3H); ^{13}C NMR (101 MHz, $CDCl_3$) δ 183.4, 165.5, 148.3, 132.4, 132.0 (t, *J* = 1.8 Hz), 128.9, 117.1 (qt, *J* = 287.9, 40.0 Hz), 114.7, 110.9 (tq, *J* = 271.6, 43.3 Hz), 55.9, 31.0, 24.9, 22.3, 13.7; ^{19}F NMR (377 MHz, $CDCl_3$) δ –82.7 (s, 3F), –94.8 (s, 2F); HRMS (ESI$^+$) *m/z* calcd for $C_{16}H_{17}F_5N_3O_2$ [M+H]$^+$: 378.12354, found 378.12374.

*Ethyl 2,2-difluoro-2-(5-(4-methoxybenzoyl)-4-phenyl-1H-1,2,3-triazol-1-yl)acetate (**1m**):*

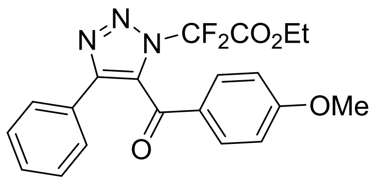

Prepared according to the general procedure. Yield: 328.2 mg pale yellow oil; 41%; ^{1}H NMR (400 MHz, $CDCl_3$) δ 8.24–8.20 (m, 2H), 7.53–7.48 (m, 2H), 7.53–7.48 (m, 2H), 7.47–7.41 (m, 4H), 4.54 (q, *J* = 7.2 Hz, 2H), 1.42 (t, *J* = 7.2 Hz, 3H); ^{13}C NMR (101 MHz, $CDCl_3$) δ 158.4 (t, *J* = 33.2 Hz), 148.9, 131.9, 130.3, 129.6, 129.1, 129.0, 128.9, 126.8, 121.1, 116.0, 110.3 (t, *J* = 267.0 Hz), 104.4, 73.5, 65.1, 14.0; ^{19}F NMR (376 MHz, $CDCl_3$) δ –85.6 (s); HRMS (ESI^+) *m/z* calcd for $C_{20}H_{17}F_2N_3O_4$ $[M+H]^+$: 402.12599, found 402.12576.

*(4-Methoxyphenyl)(4-phenyl-1-(1,1,2,2-tetrafluoroethyl)-1H-1,2,3-triazol-5-yl)methanone (**1n**):*

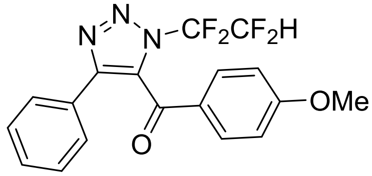

Prepared according to the general procedure. Yield: 215.5 mg pale yellow oil; 57%; ^{1}H NMR (401 MHz, $CDCl_3$) δ 7.78–7.73 (m, 2H), 7.67–7.62 (m, 2H), 7.34–7.30 (m, 3H), 6.92–6.88 (m, 2H), 6.81 (tt, *J* = 52.3, 5.0 Hz, 1H), 3.84 (s, 3H); ^{13}C NMR (101 MHz, $CDCl_3$) δ 184.2, 165.6, 146.7, 132.5, 130.6 (t, *J* = 1.8 Hz), 129.5, 129.0, 128.2, 128.1, 127.7, 114.7, 113.0 (t, *J* = 269.1, 29.3 Hz), 107.9 (tt, *J* = 254.6, 33.9 Hz), 55.8; ^{19}F NMR (377 MHz, $CDCl_3$) δ –97.3 (s, 2F), –137.6 (dt, *J* = 52.2, 8.4 Hz, 2F); HRMS (ESI^+) *m/z* calcd for $C_{18}H_{14}F_4N_3O_2$ $[M+H]^+$: 380.10167, found 380.10171.

*(4-(3,5-Dimethoxyphenyl)-1-(perfluoroethyl)-1H-1,2,3-triazol-5-yl)(p-tolyl)methanone (**1o**):*

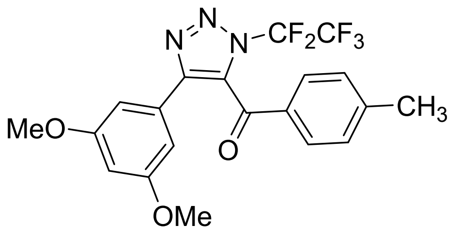

Prepared according to the general procedure. Yield: 193.0 mg colorless oil; 35%; ^{1}H NMR (401 MHz, $CDCl_3$) δ 7.69 (d, *J* = 8.3 Hz, 2H), 7.28–7.25 (m, 2H), 6.80 (d, *J* = 2.3 Hz, 2H), 6.42 (t, *J* = 2.3 Hz, 1H), 3.67 (s, 6H), 2.41 (s, 3H); ^{13}C NMR (101 MHz, $CDCl_3$) δ 185.3, 161.1, 147.3, 146.4, 132.9, 130.9 (d, *J* = 1.8 Hz), 130.2, 129.9, 129.5, 117.0 (dt, *J* = 287.9, 40.0 Hz), 110.9 (q, *J* = 43.5 Hz), 105.5, 102.4, 55.4, 22.0; ^{19}F NMR (377 MHz, $CDCl_3$) δ –82.7 (s, 3F), –94.6 (s, 2F); HRMS (ESI^+) *m/z* calcd for $C_{20}H_{17}F_5N_3O_3$ $[M+H]^+$: 442.11846, found 442.11839.

*(4-(3,5-Dimethoxyphenyl)-1-(perfluoroethyl)-1H-1,2,3-triazol-5-yl)(thiophen-2-yl)methanone (**1p**):*

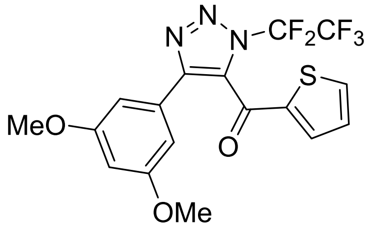

Prepared according to the general procedure. Yield: 366.3 mg pale yellow oil; 68%; ^{1}H NMR (400 MHz, $CDCl_3$) δ 7.82 (dd, *J* = 5.0, 1.2 Hz, 1H), 7.35 (dd, *J* = 3.9, 1.2 Hz, 1H), 7.06 (dd, *J* = 5.0, 3.9 Hz, 1H), 6.81 (d, *J* = 2.3 Hz, 2H), 6.42 (t, *J* = 2.3 Hz, 1H), 3.69 (s, 6H); ^{13}C NMR (101 MHz, $CDCl_3$) δ 177.1, 161.2, 146.7, 142.4, 138.2, 136.7, 130.4 (t, *J* = 1.8 Hz), 129.3, 129.3, 117.0 (qt, *J* = 287.9, 39.6

Hz), 111.0 (tq, J = 272.7, 43.5 Hz), 105.6, 102.5, 55.5; ^{19}F NMR (376 MHz, $CDCl_3$) δ –82.6 (s, 3F), –94.8 (s, 2F); HRMS (ESI$^+$) *m/z* calcd for $C_{17}H_{13}F_5N_3O_3S$ [M+H]$^+$: 434.05923, found 434.05915.

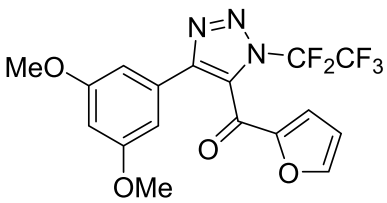

*(4-(3,5-Dimethoxyphenyl)-1-(perfluoroethyl)-1H-1,2,3-triazol-5-yl)(furan-2-yl)methanone (***1q***):*

Prepared according to the general procedure. Yield: 110.0 mg pale yellow oil; 26%; ^{1}H NMR (400 MHz, $CDCl_3$) δ 7.63 (dd, J = 1.7, 0.7 Hz, 1H), 7.16–7.12 (m, 1H), 6.78 (d, J = 2.3 Hz, 2H), 6.56 (dd, J = 3.7, 1.7 Hz, 1H), 6.44 (t, J = 2.3 Hz, 1H), 3.72 (s, 6H); ^{13}C NMR (101 MHz, $CDCl_3$) δ 171.6, 161.2, 151.4, 149.6, 147.2, 129.9 (t, J = 1.8 Hz), 129.5, 122.7, 117.0 (qt, J = 288.3, 39.8 Hz), 113.7, 110.9 (tq, J = 272.6, 43.4 Hz), 105.6, 102.3, 55.5; ^{19}F NMR (376 MHz, $CDCl_3$) δ –82.7 (s, 3F), –95.0 (s, 2F); HRMS (ESI$^+$) *m/z* calcd for $C_{17}H_{13}F_5N_3O_4$ [M+H]$^+$: 418.08207, found 418.08201.

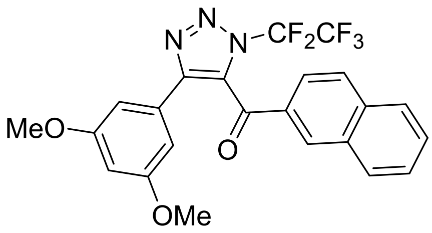

*(4-(3,5-Dimethoxyphenyl)-1-(perfluoroethyl)-1H-1,2,3-triazol-5-yl)(naphthalen-2-yl)methanone (***1r***):*

Prepared according to the general procedure. Yield: 407.8 mg yellow oil; 68%; ^{1}H NMR (401 MHz, $CDCl_3$) δ 8.18 (d, J = 2.1 Hz, 1H), 8.01 (dd, J = 8.6, 1.8 Hz, 1H), 7.94 (d, J = 8.8 Hz, 1H), 7.90–7.84 (m, 2H), 7.65 (ddd, J = 8.2, 7.0, 1.4 Hz, 1H), 7.55 (ddd, J = 8.2, 7.0, 1.3 Hz, 1H), 6.86 (d, J = 2.3 Hz, 2H), 6.38 (t, J = 2.3 Hz, 1H), 3.64 (s, 6H); ^{13}C NMR (101 MHz, $CDCl_3$) δ 185.7, 161.2, 146.7, 136.7, 133.3, 132.8, 132.4, 130.8 (t, J = 1.8 Hz), 130.1, 130.1, 129.7, 129.4, 128.1, 127.6, 123.5, 117.0 (qt, J = 287.9, 39.8 Hz), 111.0 (tq, J = 272.6, 43.3 Hz), 105.5, 102.3, 55.3; ^{19}F NMR (377 MHz, $CDCl_3$) δ –82.6 (s, 3F), –94.5 (s, 2F); HRMS (ESI$^+$) *m/z* calcd for $C_{23}H_{17}F_5N_3O_3$ [M+H]$^+$: 478.11846, found 478.11824.

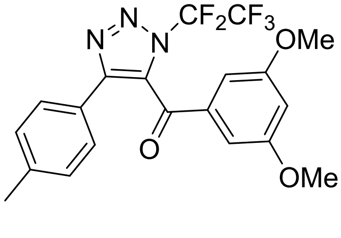

*(3,5-Dimethoxyphenyl)(1-(perfluoroethyl)-4-(p-tolyl)-1H-1,2,3-triazol-5-yl)methanone (***1s***):*

Prepared according to the general procedure. Yield: 124.0 mg pale yellow oil; 19%; ^{1}H NMR (400 MHz, $CDCl_3$) δ 7.53–7.50 (m, 2H), 7.15–7.11 (m, 2H), 6.88 (d, J = 2.3 Hz, 2H), 6.68 (t, J = 2.3 Hz, 1H), 3.73 (s, 6H), 2.30 (s, 3H); ^{13}C NMR (101 MHz, $CDCl_3$) δ 185.6, 161.4, 146.9, 139.9, 136.9, 130.1 (t, J = 1.8 Hz), 129.8, 127.6, 125.0, 117.1 (qt, J = 288.3, 40.0 Hz), 110.9 (tq, J = 272.2, 43.5 Hz), 108.1, 107.4, 55.7, 21.3; ^{19}F NMR (376 MHz, $CDCl_3$) δ –82.5 (s, 3F), –94.4 (s, 2F); HRMS (ESI$^+$) *m/z* calcd for $C_{20}H_{17}F_5N_3O_3$ [M+H]$^+$: 442.11846, found 442.11855.

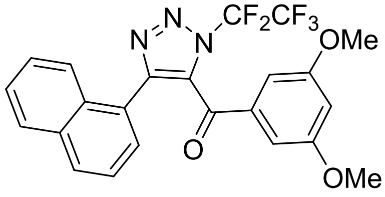

*(3,5-Dimethoxyphenyl)(4-(naphthalen-1-yl)-1-(perfluoroethyl)-1H-1,2,3-triazol-5-yl)methanone (***1t***):*

Prepared according to the general procedure. Yield: 188.3 mg pale orange oil; 33%; ^{1}H NMR (401 MHz, $CDCl_3$) δ 8.46–8.42 (m, 1H), 8.00–7.90 (m, 3H), 7.65 (ddd, *J* = 8.4, 6.9, 1.4 Hz, 1H), 7.58 (ddd, *J* = 8.1, 6.8, 1.3 Hz, 1H), 7.52 (dd, *J* = 8.3, 7.2 Hz, 1H), 7.48 (d, *J* = 2.3 Hz, 2H), 6.75 (t, *J* = 2.3 Hz, 1H), 3.89 (s, 6H); ^{13}C NMR (101 MHz, $CDCl_3$) δ 184.5, 160.9, 147.1, 136.7, 133.8, 133.1 (t, *J* = 1.8 Hz), 131.6, 130.6, 129.2, 128.7, 127.4, 126.6, 125.2, 125.1, 125.0, 117.2 (qt, *J* = 287.9, 39.6 Hz), 111.1 (tq, *J* = 273.2, 43.3 Hz), 108.3, 107.0, 55.5; ^{19}F NMR (376 MHz, $CDCl_3$) δ –82.2 (s, 3F), –94.1 (s, 2F); HRMS (ESI$^+$) *m/z* calcd for $C_{23}H_{17}F_5N_3O_3$ [M+H]$^+$: 478.11846, found 478.11833.

Synthesis of cyclopenta[*c*]isoquinolines **2** and **3**

General procedure:

Under air atmosphere, a 10 mL microwave glass tube was charged with triazole **1** (0.095–0.193 mmol in 0.95–1.93 mL DCE) and CuF_2 (1.1 equiv., pre-dried on vacuum at 90°C for 2 h). The mixture was heated under microwave irradiation (300 W) to 165 °C for 30 min. The crude reaction mixture was filtered via a paper, washed with Et_2O, evaporated with Celite and purified by column chromatography (cyclohexane/EtOAc).

Characterization of cyclopenta[*c*]isoquinolines **2** and **3**

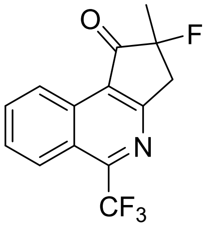

*2-Fluoro-6,8-dimethoxy-2-methyl-5-(trifluoromethyl)-2,3-dihydro-1H-cyclopenta[c]isoquinolin-1-one (***2a***):*

Prepared according to the general procedure. Yield: 22.3 mg yellow solid; 41%; m.p. 63–65 °C; ^{1}H NMR (401 MHz, $CDCl_3$) δ 9.05 (dt, *J* = 8.3, 1.1 Hz, 1H), 8.40 (dtt, *J* = 9.9, 2.2, 1.2 Hz, 1H), 8.00 (ddd, *J* = 8.3, 7.0, 1.2 Hz, 1H), 7.82 (ddd, *J* = 8.6, 7.0, 1.3 Hz, 1H), 3.75 (dd, *J* = 21.6, 18.3 Hz, 1H), 3.59 (dd, *J* = 18.3, 11.1 Hz, 1H), 1.75 (d, *J* = 22.7 Hz, 3H); ^{13}C NMR (101 MHz, $CDCl_3$) δ 200.5 (d, *J* = 18.7 Hz), 165.2 (d, *J* = 5.5 Hz), 153.6 (q, *J* = 33.7 Hz), 134.5, 133.8, 129.7, 125.8 (q, *J* = 3.5 Hz), 124.2 (2C), 123.0, 121.6 (q, *J* = 277.7 Hz), 95.4 (d, *J* = 186.0 Hz), 43.0 (d, *J* = 25.3 Hz), 21.7 (d, *J* = 26.8 Hz); ^{19}F NMR (377 MHz, $CDCl_3$) δ –63.35 (d, *J* = 2.1 Hz, 3F), –151.8 to –152.0 (m, 1F); HRMS (EI) *m/z* calcd for $C_{14}H_9F_4NO$ [M]$^+$: 283.0615, found 283.0611.

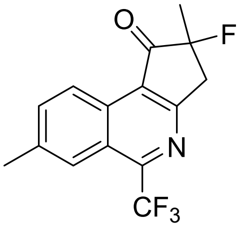

*2-Fluoro-2,7-dimethyl-5-(trifluoromethyl)-2,3-dihydro-1H-cyclopenta[c]isoquinolin-1-one (***2b***):*

Prepared according to the general procedure. Yield: 21.4 mg white solid; 55%; m.p. 128–130 °C; ^{1}H NMR (400 MHz, $CDCl_3$) δ 8.92 (d, *J* = 8.6 Hz, 1H), 8.16–8.11 (m, 1H), 7.83 (dd, *J* = 8.6, 1.8 Hz, 1H), 3.72 (dd, *J* = 21.8, 18.2 Hz, 1H), 3.57 (dd, *J* = 18.2, 11.1 Hz, 1H), 2.63 (d, *J* = 1.1 Hz, 3H), 1.74 (d, *J* = 22.7 Hz, 3H); ^{13}C NMR (101 MHz, $CDCl_3$) δ 200.6 (d, *J* = 19.1 Hz), 164.3 (d, *J* = 5.9 Hz), 152.8 (q, *J* = 33.2 Hz), 140.2, 136.7, 132.0, 124.6 (q, *J* = 3.3 Hz), 124.5, 123.9, 122.9, 121.7 (q, *J* = 277.3 Hz), 95.4 (d, *J* = 186.0 Hz), 43.0 (d, *J* = 24.9

Hz), 22.5, 21.7 (d, *J* = 26.8 Hz); ^{19}F NMR (376 MHz, $CDCl_3$) δ –63.4 (d, *J* = 2.3 Hz, 3F), –151.8 to –152.1 (m, 1F); HRMS (EI) *m/z* calcd for $C_{15}H_{11}F_4NO$ [M]$^+$: 297.0771, found 297.0770.

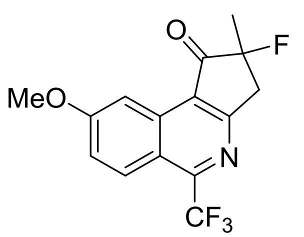

*2-fluoro-8-methoxy-2-methyl-5-(trifluoromethyl)-2,3-dihydro-1H-cyclopenta[c]isoquinolin-1-one (***2c***):*

Prepared according to the general procedure. Yield: 36.0 mg white solid; 60%; m.p. 151–153 °C; ^{1}H NMR (401 MHz, $CDCl_3$) δ 8.32 (dd, *J* = 2.8, 1.4 Hz, 1H), 8.27–8.23 (m, *J* = 9.5, 2.0 Hz, 1H), 7.36 (ddd, *J* = 9.4, 2.6, 1.1 Hz, 1H), 4.05 (s, 3H), 3.69 (dd, *J* = 21.9, 18.3 Hz, 1H), 3.53 (dd, *J* = 18.3, 11.2 Hz, 1H), 1.73 (d, *J* = 22.7 Hz, 3H); ^{13}C NMR (101 MHz, $CDCl_3$) δ 200.6 (d, *J* = 19.1 Hz), 166.1 (d, *J* = 5.5 Hz), 164.4, 152.4 (q, *J* = 33.2 Hz), 136.6, 127.5 (q, *J* = 3.3 Hz), 122.6, 121.9, 121.6 (q, *J* = 277.5 Hz), 119.7, 101.9, 95.2 (d, *J* = 185.6 Hz), 56.1, 42.9 (d, *J* = 24.9 Hz), 21.6 (d, *J* = 26.8 Hz); ^{19}F NMR (377 MHz, $CDCl_3$) δ –63.10 (d, *J* = 2.1 Hz, 3F), –151.59 to –151.86 (m, 1F); HRMS (ESI$^+$) *m/z* calcd for $C_{15}H_{12}F_4NO_2$ [M+H]$^+$: 314.07987, found 314.07961.

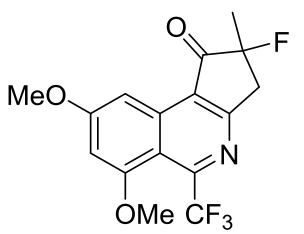

*2-Fluoro-6,8-dimethoxy-2-methyl-5-(trifluoromethyl)-2,3-dihydro-1H-cyclopenta[c]isoquinolin-1-one (***2d***):*

Prepared according to the general procedure. Yield: 24.8 mg yellow solid; 76%. Scale-up reaction: Under air atmosphere, a 10 mL microwave glass tube was charged with triazole **1** (2.11 mmol in 5.0 mL DCE) and CuF_2 (2.321 mmol). The mixture was heated under microwave irradiation (300 W) to 165 °C for 30 min. The crude reaction mixture was filtered via a paper, washed with Et_2O, evaporated with Celite and purified by column chromatography (cyclohexane/EtOAc); yield: 571.0 mg yellow solid; 79%. m.p. 131–132 °C; ^{1}H NMR (400 MHz, $CDCl_3$) δ 8.02 (d, *J* = 2.4 Hz, 1H), 6.69 (d, *J* = 2.4 Hz, 1H), 4.02 (d, *J* = 10.5 Hz, 6H), 3.71–3.43 (m, 2H), 1.72 (d, *J* = 22.7 Hz, 3H); ^{13}C NMR (101 MHz, $CDCl_3$) δ 200.6 (d, *J* = 19.1 Hz), 166.1 (d, *J* = 5.5 Hz), 166.0, 158.1, 151.4 (q, *J* = 35.0 Hz), 138.1, 121.7 (q, *J* = 275.6 Hz), 121.2, 113.9, 101.9, 96.3, 95.1, 94.5, 56.3 (d, *J* = 11.4 Hz), 42.8 (d, *J* = 24.9 Hz), 21.8 (d, *J* = 26.8 Hz); ^{19}F NMR (376 MHz, $CDCl_3$) δ –63.9 (s, 3F), –151.4 to –151.6 (m, 1F); HRMS (ESI$^+$) *m/z* calcd for $C_{16}H_{14}F_4NO_3$ [M+H]$^+$: 344.09043, found 344.09039.

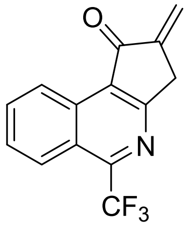

*2-Fluoro-6,8-dimethoxy-2-methyl-5-(trifluoromethyl)-2,3-dihydro-1H-cyclopenta[c]isoquinolin-1-one (***3a***):*

Under air atmosphere, a 10 mL microwave glass tube was charged with the corresponding triazole **1** (37.1 mg, 0.112 mmol in 1.12 mL DCE), KF (7.2 mg, 0.123 mmol) and NaOH (13.4 mg, 0.336 mmol). The mixture was heated under microwave irradiation (300 W) to 165 °C for 30 min. The crude reaction mixture was filtered via a paper, washed with Et_2O, evaporated with Celite and purified by column chromatography (cyclohexane/EtOAc). Yield: 9.1 mg yellow solid; 31%; m.p. 130–131 °C; ^{1}H NMR (400 MHz, $CDCl_3$) δ

9.24–9.22 (m, 1H), 8.39 (dqd, *J* = 8.6, 2.1, 1.0 Hz, 1H), 7.98 (ddd, *J* = 8.3, 7.0, 1.1 Hz, 1H), 7.80 (ddd, *J* = 8.7, 7.0, 1.3 Hz, 1H), 6.53 (td, *J* = 2.1, 0.6 Hz, 1H), 5.81 (td, *J* = 1.7, 0.6 Hz, 1H), 4.03 (t, *J* = 1.9 Hz, 2H); ^{13}C NMR (101 MHz, $CDCl_3$) δ 192.5, 165.0, 152.2 (q, *J* = 33.2 Hz), 142.4, 134.0, 133.9, 129.3, 127.0, 125.6 (q, *J* = 3.3 Hz), 124.3, 124.0, 121.8 (q, *J* = 277.3 Hz), 120.7, 34.3; ^{19}F NMR (377 MHz, $CDCl_3$) δ –63.2 (d, *J* = 2.1 Hz); HRMS (ESI$^+$) *m/z* calcd for $C_{14}H_9F_3NO$ [M+H]$^+$: 264.06308, found 264.06311.

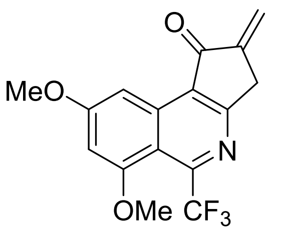

*6,8-Dimethoxy-2-methylene-5-(trifluoromethyl)-2,3-dihydro-1H-cyclopenta[c]isoquinolin-1-one (***3b***):*

Under air atmosphere, a 10 mL microwave glass tube was charged with the corresponding triazole **1** (73.4 mg, 0.188 mmol in 1.88 mL DCE), KF (12.0 mg, 0.206 mmol) and NaOH (22.5 mg, 0.563 mmol). The mixture was heated under microwave irradiation (300 W) to 165 °C for 30 min. The crude reaction mixture was filtered via a paper, washed with Et_2O, evaporated with Celite and purified by column chromatography (cyclohexane/EtOAc). Yield: 28.1 mg pale yellow solid; 46%; m.p. 90–92 °C; ^{1}H NMR (401 MHz, $CDCl_3$) δ 8.26 (d, *J* = 2.3 Hz, 1H), 6.69 (d, *J* = 2.4 Hz, 1H), 6.46 (td, *J* = 2.1, 0.7 Hz, 1H), 5.76 (td, *J* = 1.8, 0.7 Hz, 1H), 4.05 (s, 3H), 4.01 (s, 3H), 3.94 (t, *J* = 2.0 Hz, 2H); ^{13}C NMR (126 MHz, $CDCl_3$) δ 192.8, 165.9, 165.4, 158.0, 150.2 (q, *J* = 35.0 Hz), 142.7, 138.2, 125.4, 121.9 (q, *J* = 275.7 Hz), 119.9, 113.7, 101.7, 95.1, 56.3, 56.2, 34.1; ^{19}F NMR (377 MHz, $CDCl_3$) δ –63.6 (s); HRMS (EI) *m/z* calcd for $C_{16}H_{12}F_3NO_3$ [M]$^+$: 323.0764, found 323.0753.

Synthesis of indeno[1,2-*c*]isoquinolines **4**

General procedure:

Under air atmosphere, a 10 mL microwave glass tube was charged with triazole **1** (0.052–0.188 mmol in 0.52–1.88 mL DCE) and CuF_2 (1.1 equiv., pre-dried on vacuum at 90°C for 2 h). The mixture was heated under microwave irradiation (300 W) to 165 °C for 30 min. The red precipitation of indeno[1,2-*c*]isoquinoline was filtered via a paper and washed with Et_2O (2 x 10 mL). Then isoquinoline **4** on the filtration paper was separated from CuF_2 by dissolving in DCM (limited solubility, 100-150 mL) and the solution was evaporated to obtain the pure product.

Characterization of indeno[1,2-*c*]isoquinolines **4**

*7,9-Dimethoxy-5-(trifluoromethyl)-11H-indeno[1,2-c]isoquinolin-11-one (***4a***):*

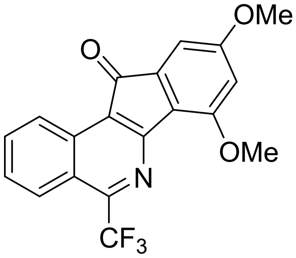

Prepared according to the general procedure. Yield: 34.8 mg red solid; 68%; m.p. 241–243 °C; ^{1}H NMR (400 MHz, $CDCl_3$) δ 8.82 (dt, *J* = 8.6, 1.1 Hz, 1H), 8.18 (dtt, *J* = 8.8, 2.2, 1.1 Hz, 1H), 7.76 (ddd, *J* = 8.6, 6.8, 1.2 Hz, 1H), 7.55 (ddd, *J* = 8.9, 6.8, 1.3 Hz, 1H), 6.88 (d, *J* = 2.1 Hz, 1H), 6.58 (d, *J* = 2.1 Hz, 1H), 4.02 (s, 3H), 3.87 (s, 3H); ^{13}C NMR (101 MHz, $CDCl_3$) δ 193.1, 164.0, 161.3, 156.5, 150.8 (q, *J* = 33.4 Hz), 137.7, 134.1, 133.5, 128.2, 125.4 (q, *J* = 3.3 Hz),

124.0, 123.8, 122.0 (q, J = 276.9 Hz), 121.5, 121.0, 105.5, 102.3, 56.7, 56.1; ^{19}F NMR (376 MHz, $CDCl_3$) δ –62.9 (s); HRMS (ESI$^+$) *m/z* calcd for $C_{19}H_{13}F_3NO_3$ [M+H]$^+$: 360.08420, found 360.08434.

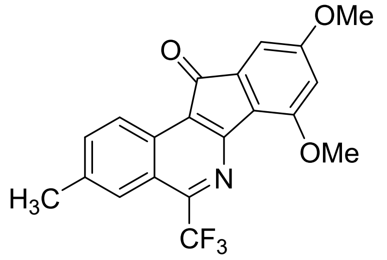

*7,9-Dimethoxy-3-methyl-5-(trifluoromethyl)-11H-indeno[1,2-c]isoquinolin-11-one (***4b***):*

Prepared according to the general procedure. Yield: 42.5 mg red solid; 61%; m.p. 213–215 °C; ^{1}H NMR (401 MHz, $CDCl_3$) δ 8.74 (d, J = 8.7 Hz, 1H), 7.94 (s, 1H), 7.62 (dd, J = 8.7, 1.7 Hz, 1H), 6.90 (d, J = 2.1 Hz, 1H), 6.60 (d, J = 2.1 Hz, 1H), 4.03 (s, 3H), 3.89 (s, 3H), 2.54 (s, 3H); ^{13}C NMR (126 MHz, $CDCl_3$) δ 193.3, 163.9, 160.5, 156.4, 149.9 (q, J = 33.2 Hz), 138.6, 137.7, 136.0, 132.6, 124.4, 124.0 (q, J = 3.0 Hz), 123.6, 122.1 (q, J = 277.0 Hz), 121.8, 121.1, 105.5, 102.3, 56.7, 56.1, 22.5; ^{19}F NMR (377 MHz, $CDCl_3$) δ –63.0 (d, J = 2.1 Hz); HRMS (EI) *m/z* calcd for $C_{20}H_{14}F_3NO_3$ [M]$^+$: 373.0920, found 373.0913.

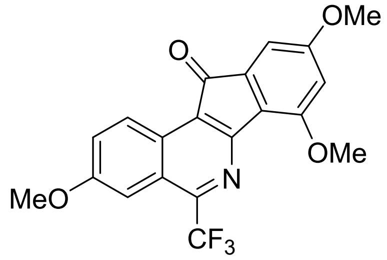

*3,7,9-Trimethoxy-5-(trifluoromethyl)-11H-indeno[1,2-c]isoquinolin-11-one (***4c***):*

Prepared according to the general procedure. Yield: 14.2 mg red solid; 79%; m.p. 248–249 °C; ^{1}H NMR (401 MHz, $CDCl_3$) δ 8.78 (d, J = 9.3 Hz, 1H), 7.46 (dd, J = 9.3, 2.4 Hz, 1H), 7.41–7.38 (m, 1H), 6.92 (d, J = 2.2 Hz, 1H), 6.63 (d, J = 2.2 Hz, 1H), 4.05 (s, 3H), 3.98 (s, 3H), 3.91 (s, 3H); ^{13}C NMR (126 MHz, $CDCl_3$) δ 193.4, 163.7, 159.1, 158.9, 156.3, 148.4 (q, J = 32.4 Hz), 137.6, 130.4, 127.6, 125.7, 125.3, 122.3 (q, J = 276.6 Hz), 122.1, 121.5, 105.7, 102.3 (q, J = 3.4 Hz), 102.2, 56.7, 56.1, 55.6; ^{19}F NMR (377 MHz, $CDCl_3$) δ –63.7 (d, J = 2.1 Hz); HRMS (ESI$^+$) *m/z* calcd for $C_{20}H_{15}F_3NO_4$ [M+H]$^+$: 390.09477, found 390.09494.

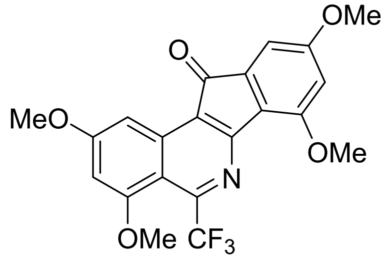

*2,4,7,9-Tetramethoxy-5-(trifluoromethyl)-11H-indeno[1,2-c]isoquinolin-11-one (***4d***):*

Prepared according to the general procedure. Yield: 25.9 mg orange solid; 68%; m.p. 251–253 °C; ^{1}H NMR (400 MHz, $CDCl_3$) δ 7.84 (d, J = 2.3 Hz, 1H), 6.89 (d, J = 2.1 Hz, 1H), 6.59 (d, J = 2.1 Hz, 1H), 6.49 (d, J = 2.3 Hz, 1H), 4.03 (s, 3H), 4.01 (s, 3H), 3.97 (s, 3H), 3.90 (s, 3H); ^{13}C NMR (126 MHz, $CDCl_3$) δ 193.4, 164.9, 164.0, 162.0, 157.7, 156.6, 149.1 (q, J = 35.0 Hz), 138.2, 138.2, 122.1 (q, J = 275.2 Hz), 121.2, 119.3, 114.7, 105.2, 102.2, 101.1, 93.8, 56.8, 56.08, 56.14, 56.03; ^{19}F NMR (376 MHz, $CDCl_3$) δ –62.6 (s); HRMS (ESI$^+$) *m/z* calcd for $C_{21}H_{17}F_3NO_5$ [M+H]$^+$: 420.10533, found 420.10540.

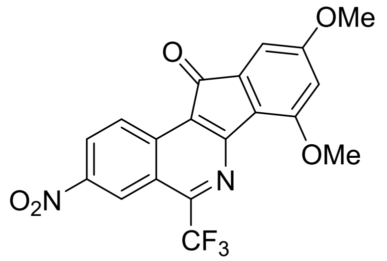

*7,9-Dimethoxy-3-nitro-5-(trifluoromethyl)-11H-indeno[1,2-c]isoquinolin-11-one (***4e***):*

Prepared according to the general procedure. Yield: 32.3 mg purple solid; 66%; m.p. 252–254 °C; ^{1}H NMR (401 MHz, $CDCl_3$) δ 9.13 (s, 1H), 9.00 (d, *J* = 9.5 Hz, 1H), 8.52 (d, *J* = 7.7 Hz, 1H), 6.97 (s, 1H), 6.65 (s, 1H), 4.06 (s, 3H), 3.93 (s, 3H); ^{13}C NMR (126 MHz, $CDCl_3$) δ 192.0, 165.2, 164.2, 157.4, 153.3 (q, *J* = 34.1 Hz), 146.3, 137.8, 135.9, 126.6, 125.8, 122.5 (q, *J* = 3.8, 3.0 Hz, 2C), 121.4 (q, *J* = 276.4 Hz), 120.6, 120.5, 105.6, 103.0, 56.7, 56.3; ^{19}F NMR (377 MHz, $CDCl_3$) δ –62.8 (s); HRMS (ESI$^+$) *m/z* calcd for $C_{19}H_{12}F_3N_2O_5$ $[M+H]^+$: 405.06928, found 405.06948.

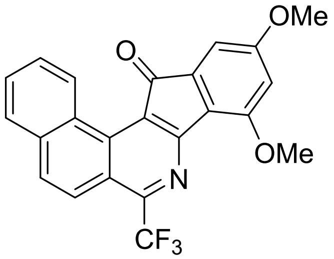

*9,11-Dimethoxy-7-(trifluoromethyl)-13H-benzo[f]indeno[1,2-c]isoquinolin-13-one (**4f**):*

Prepared according to the general procedure. Yield: 27.5 mg red solid; 56%; m.p. 232–234 °C; ^{1}H NMR (401 MHz, $CDCl_3$) δ 9.89–9.86 (m, 1H), 8.01 (dq, *J* = 9.4, 2.2 Hz, 1H), 7.90–7.87 (m, 1H), 7.82–7.74 (m, 3H), 6.99 (d, *J* = 2.1 Hz, 1H), 6.66 (d, *J* = 2.1 Hz, 1H), 4.07 (s, 3H), 3.93 (s, 3H); ^{13}C NMR (101 MHz, $CDCl_3$) δ 191.9, 164.2, 163.4, 156.8, 149.5 (d, *J* = 32.3 Hz), 138.0, 137.6, 134.6, 131.1, 131.1, 130.2, 128.3, 128.0, 127.4, 125.0, 124.2, 123.8, 121.4, 121.1 (q, *J* = 3.6 Hz), 105.9, 101.9, 56.7, 56.1; ^{19}F NMR (377 MHz, $CDCl_3$) δ –62.2 (d, *J* = 2.1 Hz); HRMS (EI) *m/z* calcd for $C_{23}H_{14}F_3NO_3$ $[M]^+$: 409.0920, found 409.0912.

Synthesis of 6,6-difluoro-1,3-oxazines **5**

General procedure:

Under air atmosphere, a 10 mL microwave glass tube was charged with the corresponding triazole **1** (0.118–0.256 mmol in 1.18–2.56 mL DCE). The mixture was heated under microwave irradiation (300 W) to 165 °C for 5 min. The crude reaction mixture was filtered via a paper, washed with Et_2O, evaporated and purified by column chromatography (cyclohexane/EtOAc).

Characterization of 6,6-difluoro-1,3-oxazines **5**

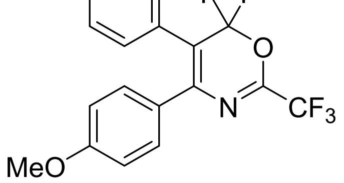

*6,6-Difluoro-4-(4-methoxyphenyl)-5-phenyl-2-(trifluoromethyl)-6H-1,3-oxazine (**5a**):*

Prepared according to the general procedure. Yield: 43.0 mg; 74%; colorless oil. ^{1}H NMR (400 MHz, $CDCl_3$) δ 7.39–7.29 (m, 7H), 6.74–6.70 (m, 2H), 3.77 (s, 3H); ^{13}C NMR (101 MHz, $CDCl_3$) δ 160.7, 143.0 (q, *J* = 42.5 Hz), 142.0 (t, *J* = 4.9 Hz), 131.5, 130.8, 130.6, 129.3, 129.1, 126.3, 120.9 (t, *J* = 254.2 Hz), 117.6 (q, *J* = 275.5 Hz), 115.9 (t, *J* = 29.0 Hz), 113.7, 55.4; ^{19}F NMR (376 MHz, $CDCl_3$) δ –42.4 (s, 2F), –73.2 (s, 3F); HRMS (ESI$^+$) *m/z* calcd for $C_{18}H_{13}F_5NO_2$ $[M+H]^+$: 370.08610, found 370.08642.

*6,6-Difluoro-4-(furan-2-yl)-5-phenyl-2-(trifluoromethyl)-6H-1,3-oxazine (**5b**):*

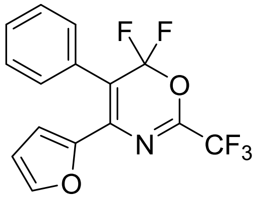

Prepared according to the general procedure. Yield: 17.2 mg; 44%; colorless oil. ^1H NMR (400 MHz, $CDCl_3$) δ 7.49–7.42 (m, 3H), 7.40–7.36 (m, 2H), 7.27–7.26 (m, 1H), 6.66 (dd, *J* = 3.5, 1.0 Hz, 1H), 6.37 (dd, *J* = 3.5, 1.8 Hz, 1H); ^{13}C NMR (101 MHz, $CDCl_3$) δ 148.6, 145.3, 143.8 (q, *J* = 42.7 Hz), 133.1 (t, *J* = 5.0 Hz), 130.4, 129.9, 129.5, 128.7, 120.5, 116.1 (q, *J* = 275.9 Hz), 115.9, 113.2, 111.9; ^{19}F NMR (376 MHz, $CDCl_3$) δ –43.3 (s, 2F), –73.4 (s, 3F); HRMS (EI) *m/z* calcd for $C_{15}H_8F_5NO_2$ $[M]^+$: 329.0470, found 329.0469.

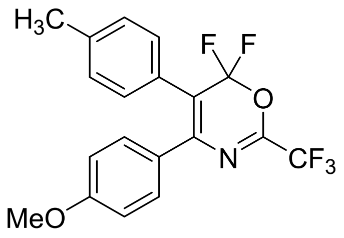

*6,6-Difluoro-4-(4-methoxyphenyl)-5-(p-tolyl)-2-(trifluoromethyl)-6H-1,3-oxazine (***5c***):*

Prepared according to the general procedure. Yield: 59.7 mg; 61%; colorless oil. ^1H NMR (401 MHz, $CDCl_3$) δ 7.35–7.30 (m, 2H), 7.21 (d, *J* = 8.3 Hz, 2H), 7.16 (d, *J* = 8.4, 2H), 6.75–6.72 (m, 2H), 3.77 (s, 3H), 2.37 (s, 3H); ^{13}C NMR (101 MHz, $CDCl_3$) δ 160.7, 142.8 (q, *J* = 42.5 Hz), 141.7 (t, *J* = 5.0 Hz), 139.3, 131.4, 130.4, 129.8, 127.8, 126.6, 121.0 (t, *J* = 254.0 Hz), 116.3 (q, *J* = 275.5 Hz), 115.9 (t, *J* = 28.8 Hz), 113.7, 55.4, 21.5; ^{19}F NMR (377 MHz, $CDCl_3$) δ –42.5 (s, 2F), –73.2 (s, 3F); HRMS (EI) *m/z* calcd for $C_{19}H_{14}F_5NO_2$ $[M]^+$: 383.0939, found 383.0945.

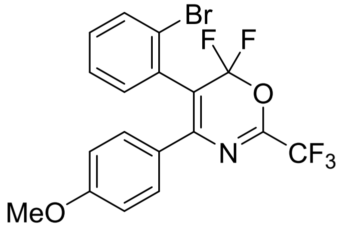

*5-(2-Bromophenyl)-6,6-difluoro-4-(4-methoxyphenyl)-2-(trifluoromethyl)-6H-1,3-oxazine (***5d***):*

Prepared according to the general procedure. Yield: 30.0 mg; 56%; colorless oil. ^1H NMR (400 MHz, $CDCl_3$) δ 7.61–7.59 (m, 1H), 7.49 (dtd, *J* = 7.7, 2.0, 0.4 Hz, 1H), 7.42 (td, *J* = 7.6, 1.3 Hz, 1H), 7.36–7.32 (m, 2H), 7.29 (ddd, *J* = 8.1, 7.3, 1.7 Hz, 1H), 6.77–6.69 (m, 2H), 3.77 (s, 3H); ^{13}C NMR (101 MHz, $CDCl_3$) δ 161.0, 144.0 (t, *J* = 42.6 Hz), 143.4 (q, *J* = 4.2 Hz), 133.7, 132.8, 131.6, 131.0, 130.7, 127.8, 126.2, 125.3, 120.6 (t, *J* = 254.2 Hz), 116.2 (q, *J* = 275.8 Hz), 113.9 (dd, *J* = 29.4 Hz), 113.8, 55.4; ^{19}F NMR (376 MHz, $CDCl_3$) δ –38.6 (d, *J* = 178.5 Hz, 1F), –49.4 (d, *J* = 178.5 Hz, 1F), –73.2 (s, 3F); HRMS (EI) *m/z* calcd for $C_{18}H_{11}F_5BrNO_2$ $[M]^+$: 446.9888, found 446.9894.

Synthesis of 1,3-oxazin-6-ones **6**

General procedure:

Under air atmosphere, a 10 mL microwave glass tube was charged with the corresponding triazole **1** (0.072–0.249 mmol in 0.72–2.49 mL DCE) and CuF_2 (1.1 equiv., pre-dried on vacuum at 90°C for 2 h). The mixture was heated under microwave irradiation (300 W) to 165 °C for 20 min. The crude reaction mixture was filtered via a paper, washed with Et_2O, evaporated with Celite and purified by column chromatography (cyclohexane/EtOAc).

Characterization of 1,3-oxazin-6-ones **6**

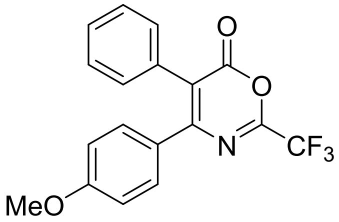

*4-(4-Methoxyphenyl)-5-phenyl-2-(trifluoromethyl)-6H-1,3-oxazin-6-one (**6a**):*

Prepared according to the general procedure. Yield: 58.9 mg; 85%; orange oil. ^{1}H NMR (400 MHz, $CDCl_3$) δ 7.43–7.37 (m, 5H), 7.33–7.29 (m, 2H), 6.76–6.72 (m, 2H), 3.79 (s, 3H); ^{13}C NMR (101 MHz, $CDCl_3$) δ 162.0, 157.9, 156.0, 150.4 (q, *J* = 42.2 Hz), 132.5, 131.9, 130.2, 129.2, 129.1, 126.4, 119.8, 116.0 (q, *J* = 276.6 Hz), 113.9, 55.5; ^{19}F NMR (376 MHz, $CDCl_3$) δ –73.1 (s); HRMS (ESI$^+$) *m/z* calcd for $C_{18}H_{13}F_5NO_2$ $[M+H]^+$: 370.08610, found 370.08642.

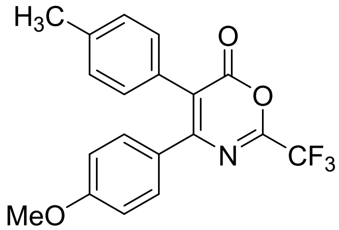

*4-(4-Methoxyphenyl)-5-(p-tolyl)-2-(trifluoromethyl)-6H-1,3-oxazin-6-one (**6b**):*

Prepared according to the general procedure. Yield: 73.0 mg; 81%; orange oil. ^{1}H NMR (401 MHz, $CDCl_3$) δ 7.48–7.44 (m, 2H), 7.21 (s, 4H), 6.79–6.75 (m, 2H), 3.82 (s, 3H), 2.40 (s, 3H); ^{13}C NMR (101 MHz, $CDCl_3$) δ 161.9, 158.0, 155.7, 150.2 (q, *J* = 42.3 Hz), 139.3, 132.5, 130.1, 129.9, 128.9, 126.6, 119.9, 116.0 (q, *J* = 276.6 Hz), 113.9, 55.5, 21.6; ^{19}F NMR (377 MHz, $CDCl_3$) δ –72.6 (s); HRMS (EI) *m/z* calcd for $C_{19}H_{14}F_3NO_3$ $[M]^+$: 361.0920, found 361.0925.

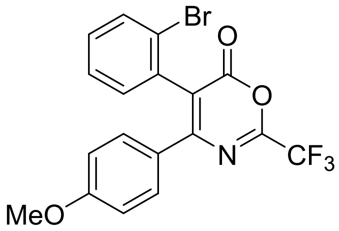

*5-(2-Bromophenyl)-4-(4-methoxyphenyl)-2-(trifluoromethyl)-6H-1,3-oxazin-6-one (**6c**):*

Prepared according to the general procedure. Yield: 35.2 mg; 58%; pale orange oil. ^{1}H NMR (401 MHz, $CDCl_3$) δ 7.72 (dd, *J* = 7.9, 1.4 Hz, 1H), 7.49–7.45 (m, 2H), 7.40–7.30 (m, 2H), 7.21 (dd, *J* = 7.4, 2.0 Hz, 1H), 6.81–6.77 (m, 2H), 3.81 (s, 3H); ^{13}C NMR (101 MHz, $CDCl_3$) δ 162.4, 156.7, 156.6, 151.0 (q, *J* = 42.2 Hz), 133.7, 133.6, 132.0, 131.6, 130.9, 128.4, 126.1, 124.7, 119.3, 116.0 (q, *J* = 276.6 Hz), 114.1, 55.5; ^{19}F NMR (377 MHz, $CDCl_3$) δ –73.0 (s); HRMS (EI) *m/z* calcd for $C_{18}H_{11}F_3BrNO_3$ $[M]^+$: 424.9869, found 424.9867.

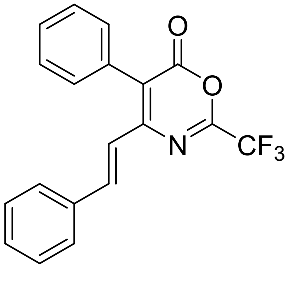

*(E)-5-Phenyl-4-styryl-2-(trifluoromethyl)-6H-1,3-oxazin-6-one (**6d**):*

Prepared according to the general procedure. Yield: 10.3 mg; 42%, yellow solid. m.p. 146–148 °C; ^{1}H NMR (400 MHz, $CDCl_3$) δ 7.99 (d, *J* = 15.5 Hz, 1H), 7.57–7.51 (m, 3H), 7.50–7.44 (m, 4H), 7.40–7.36 (m, 3H), 6.94 (d, *J* = 15.4 Hz, 1H); ^{13}C NMR (101 MHz, $CDCl_3$) δ 157.3, 153.7, 150.4 (q, *J* = 42.4 Hz), 142.6, 135.2, 130.6, 130.5, 130.5, 129.6, 129.1, 128.9, 128.5, 121.3, 120.1, 116.0 (q, *J* = 276.6 Hz); ^{19}F NMR (376 MHz, $CDCl_3$) δ –73.1 (s); HRMS (ESI$^+$) *m/z* calcd for $C_{19}H_{13}F_3NO_2$ $[M+H]^+$: 344.08929, found 344.08957.

*4-(Furan-2-yl)-5-phenyl-2-(trifluoromethyl)-6H-1,3-oxazin-6-one (**6e**):*

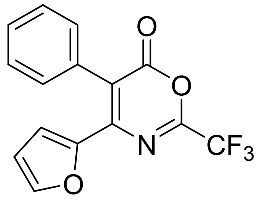

Prepared according to the general procedure. Yield: 26.2 mg; 67%; colorless oil. ^{1}H NMR (401 MHz, $CDCl_3$) δ 7.49–7.45 (m, 3H), 7.39 (dd, *J* = 1.8, 0.9 Hz, 1H), 7.36–7.33 (m, 2H), 6.89 (dd, *J* = 3.6, 0.9 Hz, 1H), 6.45 (dd, *J* = 3.7, 1.7 Hz, 1H); ^{13}C NMR (101 MHz, $CDCl_3$) δ 157.5, 151.4 (q, *J* = 42.3 Hz), 149.0, 147.5, 145.4, 131.2, 129.8, 129.4, 128.8, 120.2, 116.8, 115.9 (q, *J* = 276.9 Hz), 112.9; ^{19}F NMR (377 MHz, $CDCl_3$) δ –73.2 (s); HRMS (EI) *m/z* calcd for $C_{15}H_8F_3NO_3$ $[M]^+$: 307.0451, found 307.0452.

*5-(3,5-Dimethoxyphenyl)-4-(furan-2-yl)-2-(trifluoromethyl)-6H-1,3-oxazin-6-one (**6f**):*

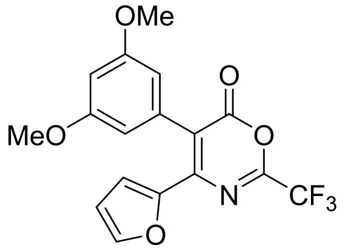

Prepared according to the general procedure. Yield: 28.9 mg; 40%; pale yellow oil. ^{1}H NMR (401 MHz, $CDCl_3$) δ 7.47 (dd, *J* = 1.8, 0.8 Hz, 1H), 6.87 (dd, *J* = 3.6, 0.8 Hz, 1H), 6.56 (t, *J* = 2.3 Hz, 1H), 6.47–6.46 (m, 3H), 3.79 (s, 6H); ^{13}C NMR (101 MHz, $CDCl_3$) δ 161.2, 157.1, 151.3 (q, *J* = 42.5 Hz), 148.5, 147.5, 145.5, 132.9, 120.3, 116.6, 115.8 (q, *J* = 276.6 Hz), 112.9, 107.3, 101.5, 55.5; ^{19}F NMR (377 MHz, $CDCl_3$) δ –73.2 (s); HRMS (EI) *m/z* calcd for $C_{17}H_{12}F_3NO_5$ $[M]^+$: 367.0662, found 367.0664.

*5-Phenyl-4-(thiophen-2-yl)-2-(trifluoromethyl)-6H-1,3-oxazin-6-one (**6g**):*

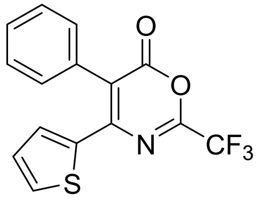

Prepared according to the general procedure. Yield: 14.1 mg; 41%; red solid. m.p. 133–135 °C; ^{1}H NMR (401 MHz, $CDCl_3$) δ 7.56–7.52 (m, 3H), 7.50 (dd, *J* = 5.0, 1.2 Hz, 1H), 7.38–7.34 (m, 2H), 7.29 (dd, *J* = 3.9, 1.2 Hz, 1H), 6.97 (dd, *J* = 5.1, 4.0 Hz, 1H); ^{13}C NMR (101 MHz, $CDCl_3$) δ 157.3, 150.6 (q, *J* = 42.5 Hz), 150.3, 138.1, 134.2, 134.1, 131.1, 130.0, 129.8, 129.7, 128.3, 117.1, 115.8 (q, *J* = 276.9 Hz); ^{19}F NMR (377 MHz, $CDCl_3$) δ –73.2 (s, 3F); HRMS (EI) *m/z* calcd for $C_{15}H_8F_3NO_2S$ $[M]^+$: 323.0222, found 323.0220.

*5-(3,5-Dimethoxyphenyl)-4-(thiophen-2-yl)-2-(trifluoromethyl)-6H-1,3-oxazin-6-one (**6h**):*

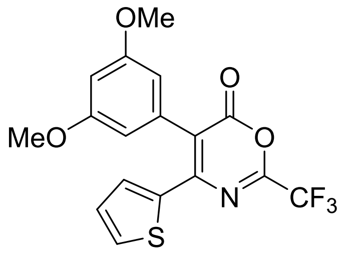

Prepared according to the general procedure. Yield: 42.5 mg; 53%; orange solid. m.p. 242–243 °C; ^{1}H NMR (401 MHz, $CDCl_3$) δ 7.54 (dd, *J* = 5.0, 1.2 Hz, 1H), 7.40 (dd, *J* = 3.9, 1.2 Hz, 1H), 7.00 (dd, *J* = 5.0, 3.9 Hz, 1H), 6.61 (t, *J* = 2.3 Hz, 1H), 6.48 (d, *J* = 2.2 Hz, 2H), 3.80 (s, 6H); ^{13}C NMR (101 MHz, $CDCl_3$) δ 162.2, 157.2, 150.7 (q, *J* = 42.5 Hz), 150.4, 137.9, 134.6, 134.5, 132.9, 128.5, 117.0, 115.9 (q, *J* = 276.6 Hz), 107.2, 102.2, 55.7; ^{19}F NMR (377 MHz, $CDCl_3$) δ –73.2 (s); HRMS (ESI^+) *m/z* calcd for $C_{17}H_{13}F_3NO_4S$ $[M+H]^+$: 384.05119, found 384.05114.

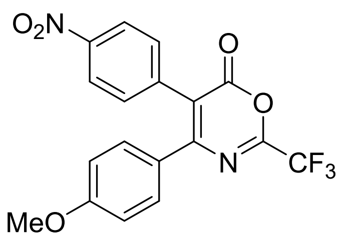

*4-(4-Methoxyphenyl)-5-(4-nitrophenyl)-2-(trifluoromethyl)-6H-1,3-oxazin-6-one (**6i**):*

Under air atmosphere, s 10 mL microwave glass tube was charged with the corresponding triazole **1** (0.226 mmol in 2.26 mL DCE) and CuF_2 (0.249 mmol). The mixture was heated under microwave irradiation (300 W) to 190 °C for 120 min. The crude reaction mixture was filtered via a paper, washed with Et_2O, evaporated with Celite and purified by column chromatography (cyclohexane/EtOAc). Yield: 38.3 mg; 47%; yellow solid. m.p. 147–149 °C; ^{1}H NMR (401 MHz, $CDCl_3$) δ 8.22 (d, *J* = 8.7 Hz, 2H), 7.52 (d, *J* = 8.7 Hz, 2H), 7.38 (d, *J* = 8.9 Hz, 2H), 6.78 (d, *J* = 8.9 Hz, 2H), 3.81 (s, 3H); ^{13}C NMR (101 MHz, $CDCl_3$) δ 162.7, 157.6, 157.0, 151.1 (q, *J* = 42.5 Hz), 148.0, 139.0, 132.7, 131.7, 125.4, 124.2, 117.5, 115.9 (q, *J* = 276.9 Hz), 114.3, 55.6; ^{19}F NMR (377 MHz, $CDCl_3$) δ –73.1 (s); HRMS (ESI$^+$) *m/z* calcd for $C_{18}H_{12}F_3N_2O_5$ $[M+H]^+$: 393.06928, found 393.06941.

References

[1] SAINT. Bruker AXS Inc., Madison, Wisconsin, USA, 2015.

[2] Altomare, A.; Cascarano, G.; Giacovazzo G.; Guagliardi A.; Burla M. C.; Polidori, G.; Camalli, M. *J. Appl. Cryst.* 1994**, 27**, 435.

[3] Palatinus L., Chapuis G. *J. Appl. Cryst.* 2007, **40**, 786-790.

[4] Betteridge, P. W.; Carruthers, J. R.; Cooper, R. I.; Prout, K., Watkin, D. J. *J. Appl. Cryst.* 2003, **36**, 1487.

Copies of NMR spectra

Figure S4. ^{1}H NMR spectrum of **1a** (401 MHz; $CDCl_3$)

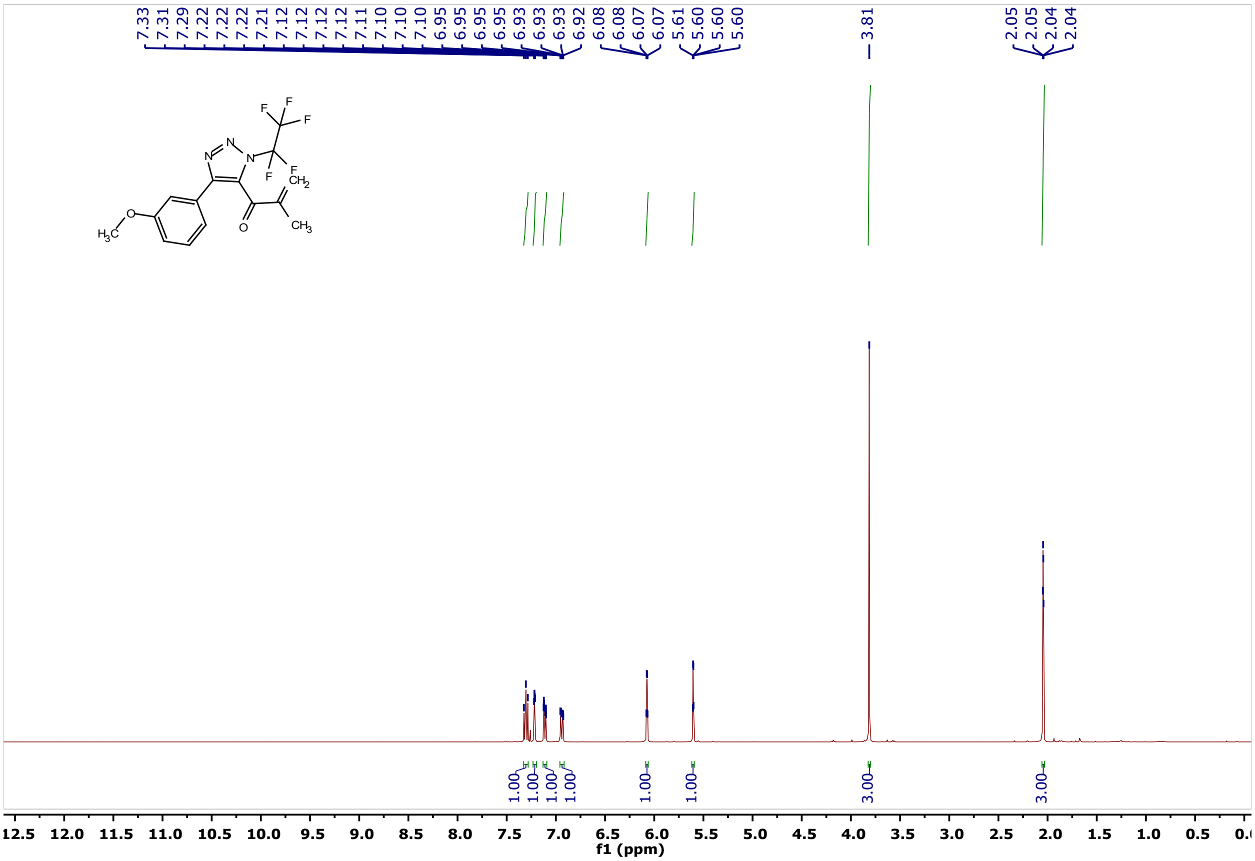

Figure S5. ^{13}C NMR spectrum of **1a** (101 MHz; $CDCl_3$)

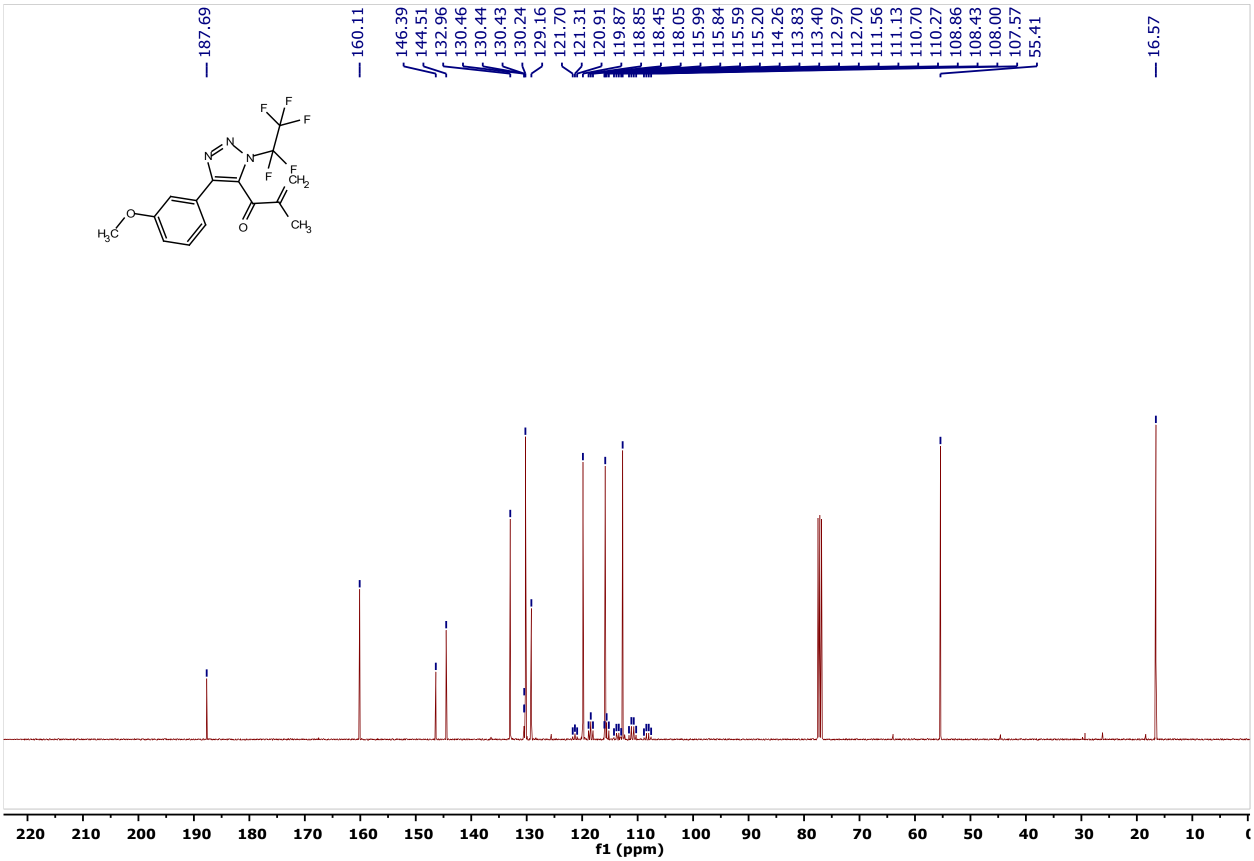

Figure S6. ^{19}F NMR spectrum of **1a** (377 MHz; $CDCl_3$)

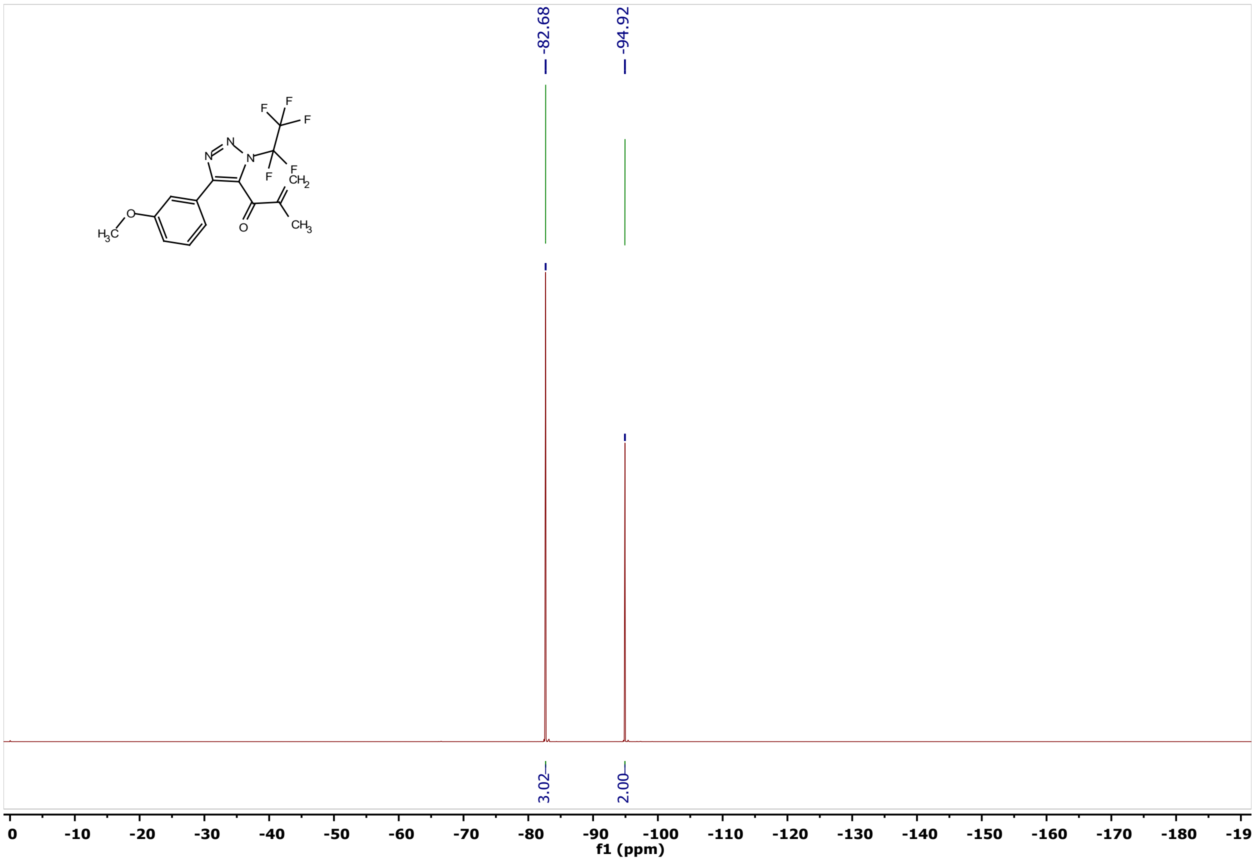

Figure S7. ^{1}H NMR spectrum of **1b** (401 MHz; $CDCl_3$)

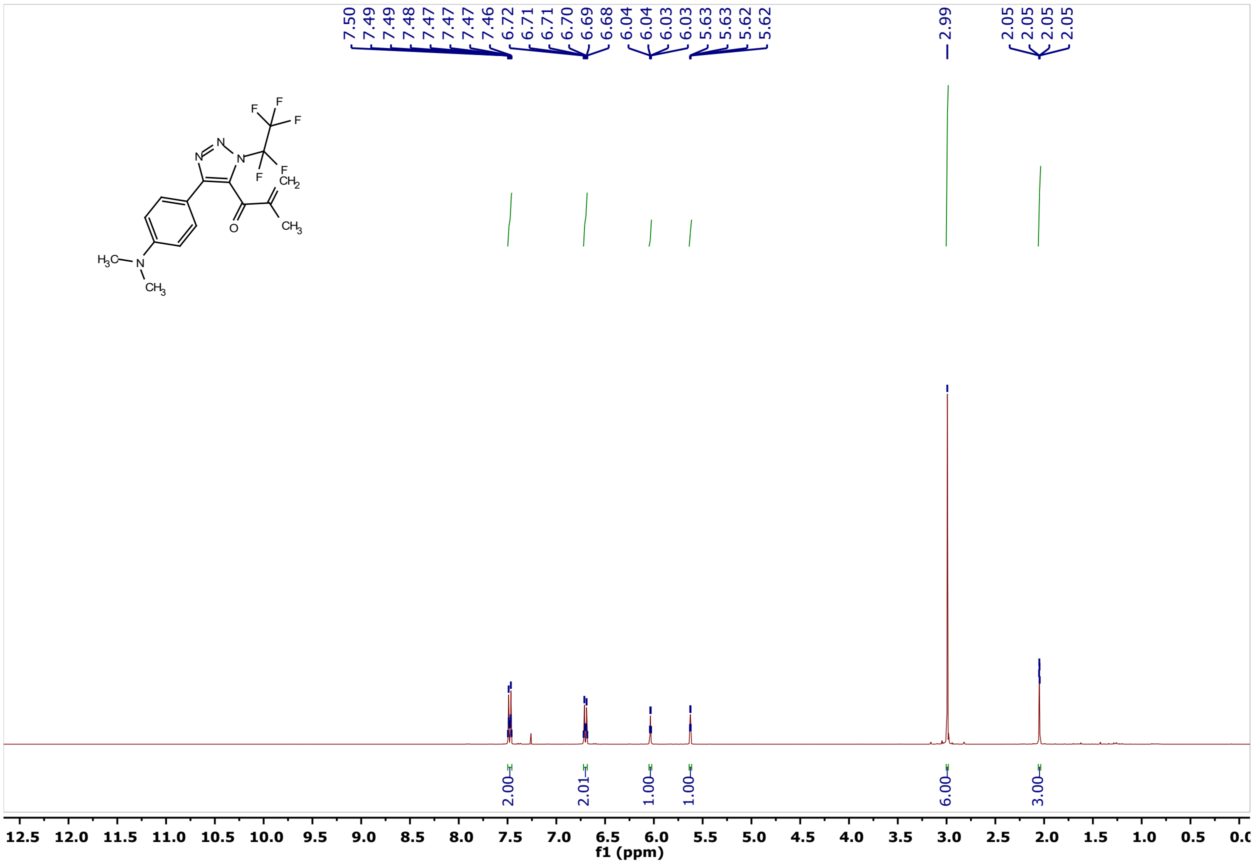

Figure S8. ^{13}C NMR spectrum of **1b** (101 MHz; $CDCl_3$)

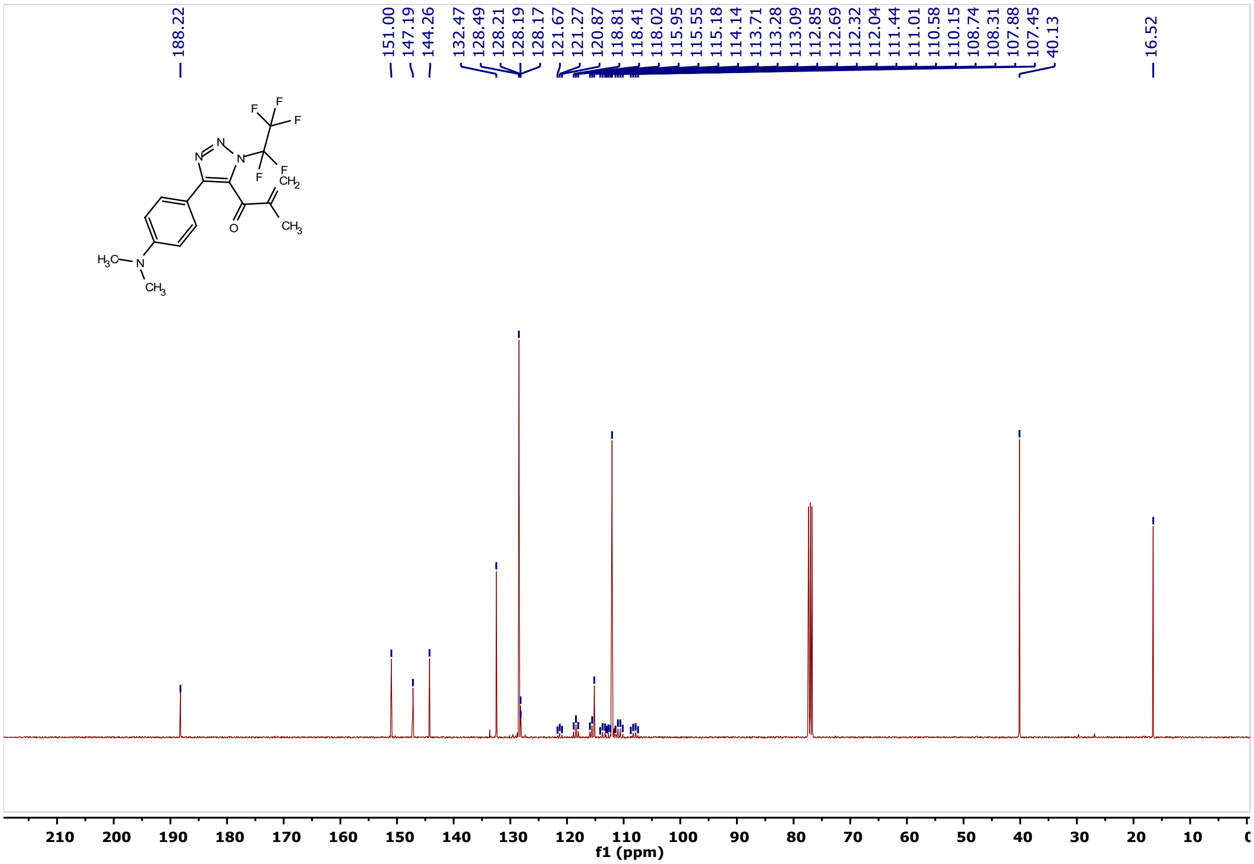

Figure S9. ^{19}F NMR spectrum of **1b** (377 MHz; $CDCl_3$)

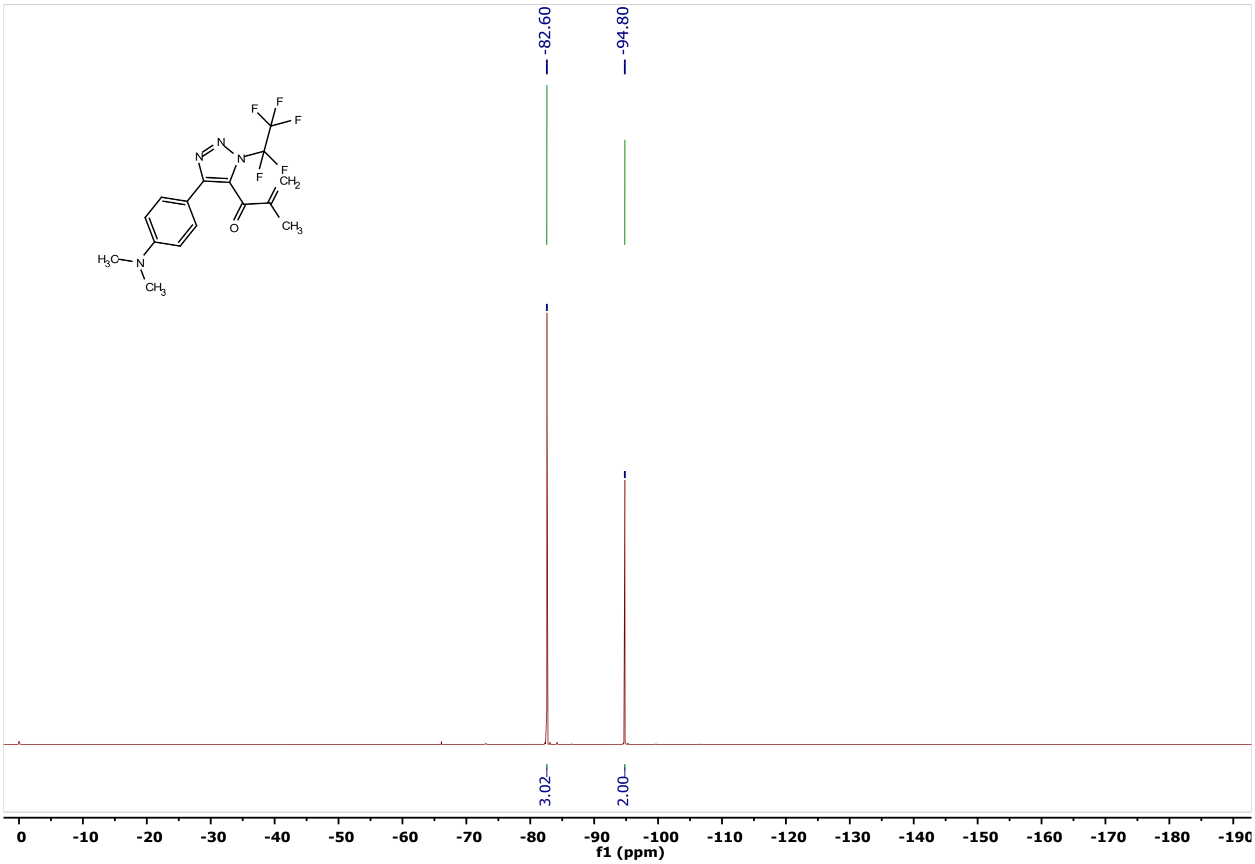

Figure S10. ^{1}H NMR spectrum of **1c** (401 MHz; $CDCl_3$)

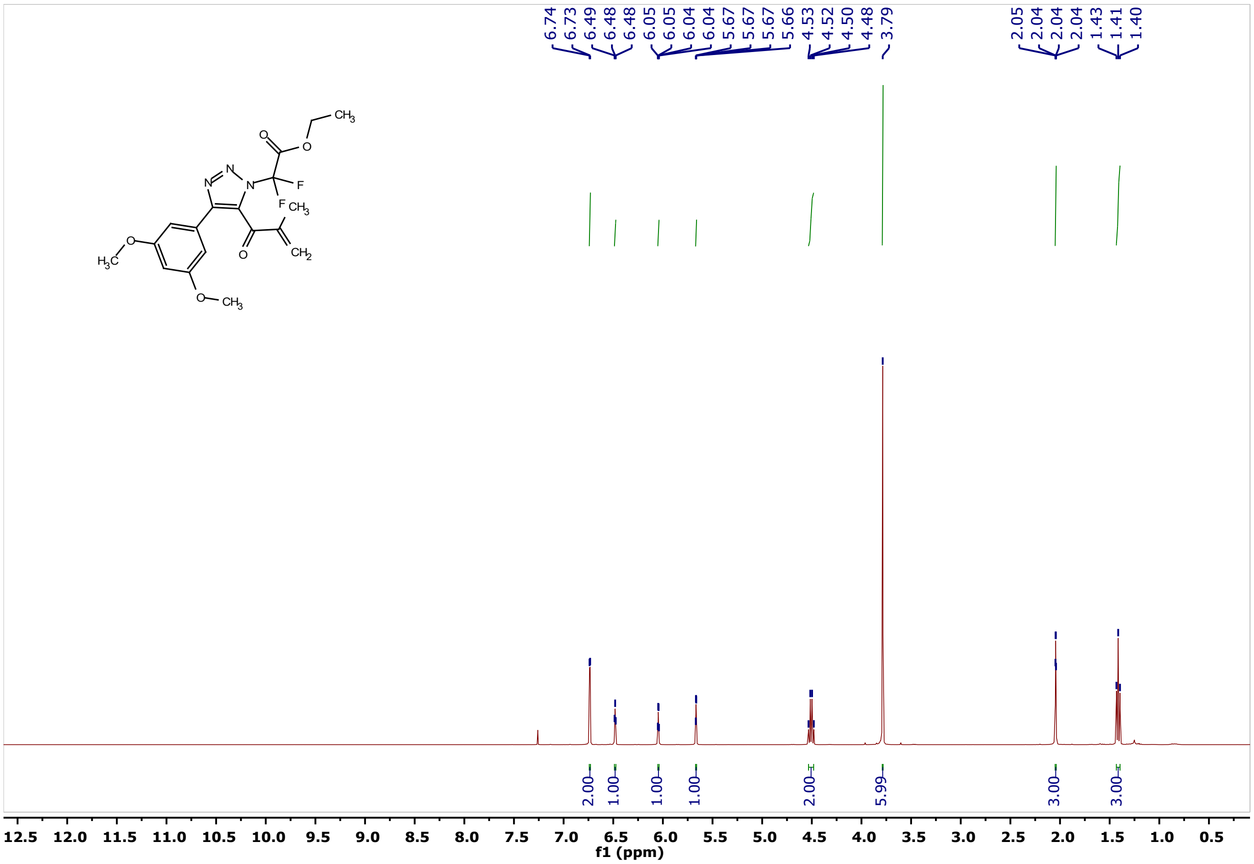

Figure S11. ^{13}C NMR spectrum of **1c** (101 MHz; $CDCl_3$)

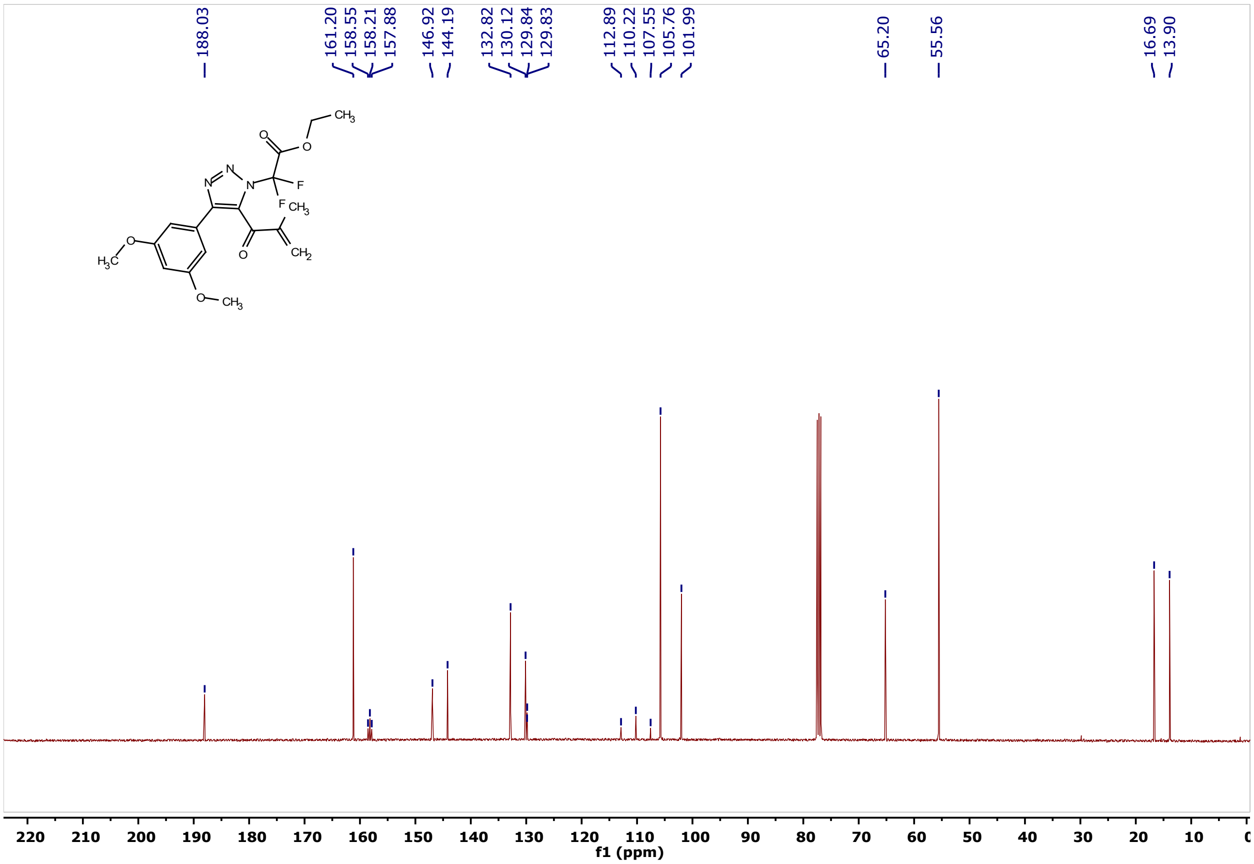

Figure S12. ^{19}F NMR spectrum of **1c** (377 MHz; $CDCl_3$)

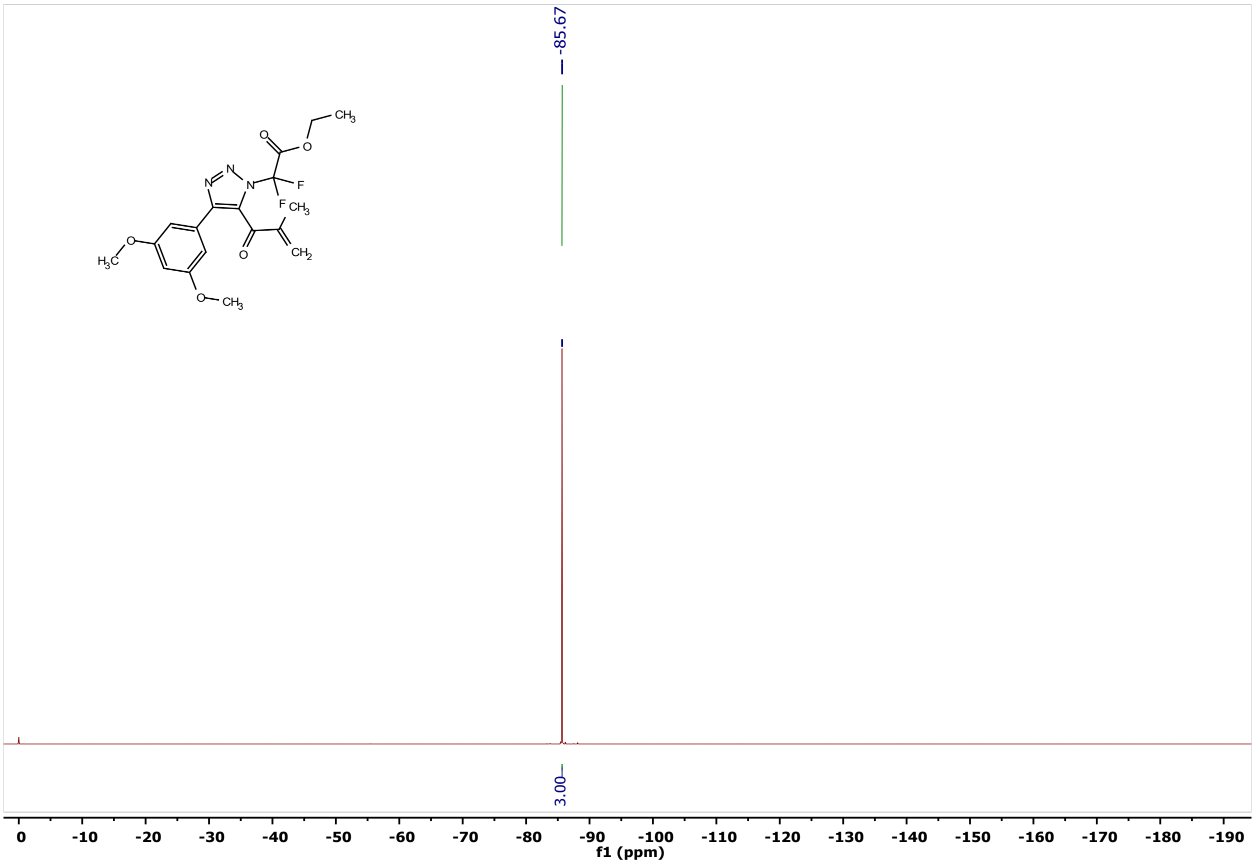

Figure S13. ^{1}H NMR spectrum of **1d** (401 MHz; $CDCl_3$)

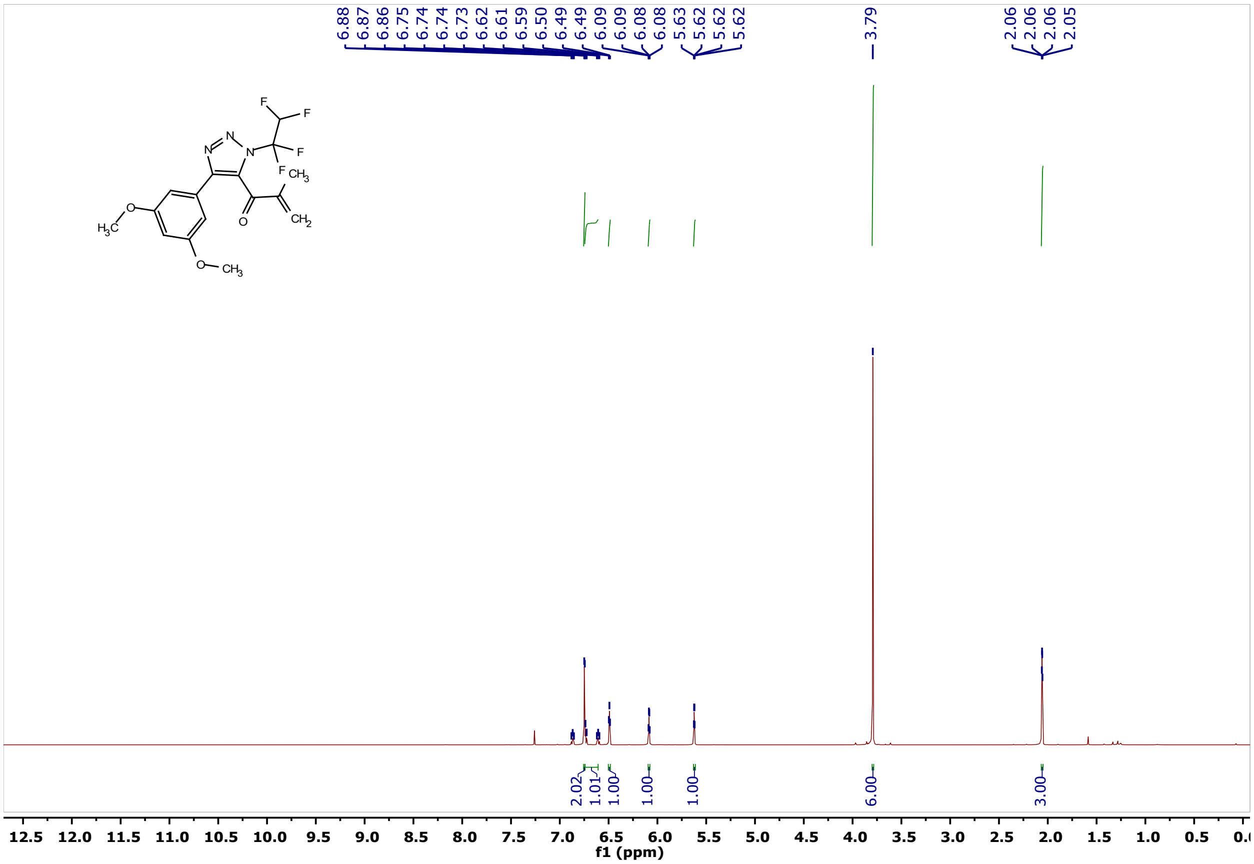

Figure S14. ^{13}C NMR spectrum of **1d** (101 MHz; $CDCl_3$)

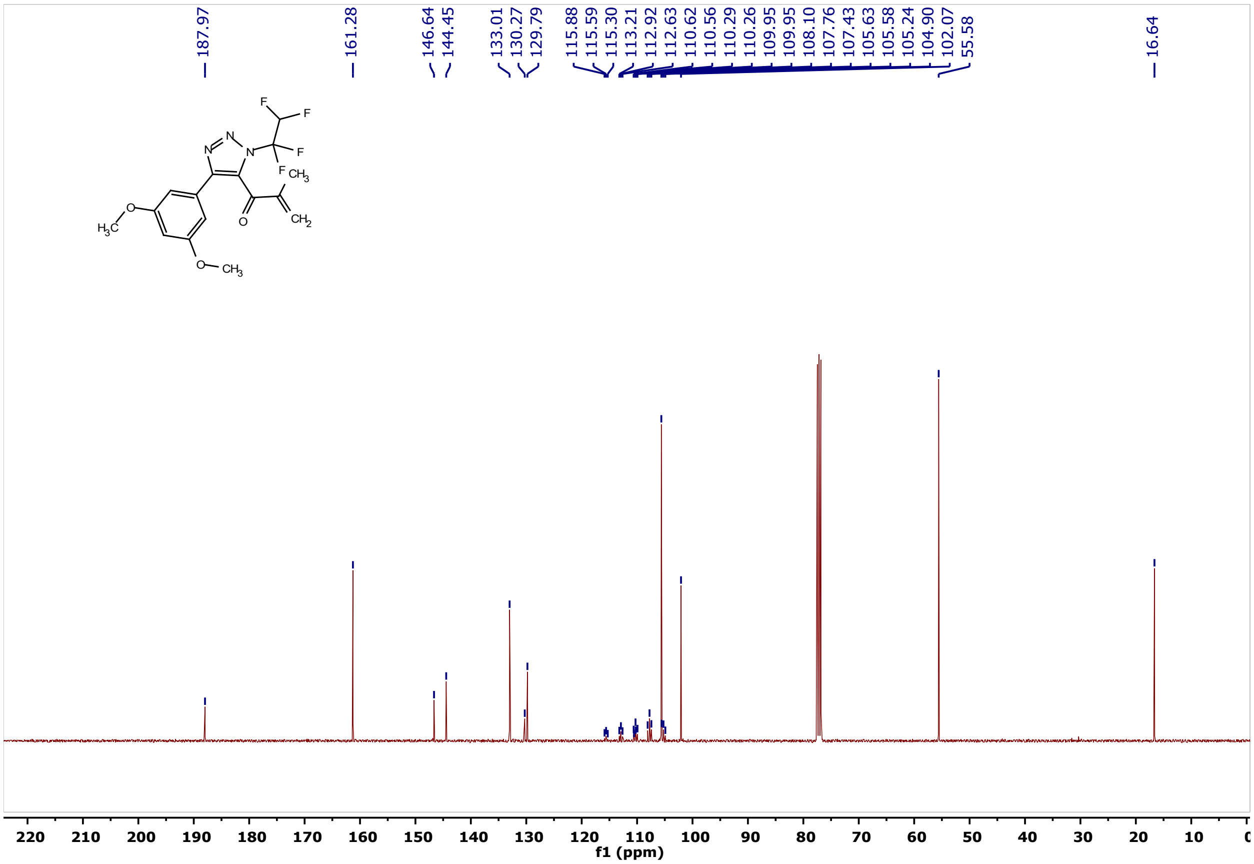

Figure S15. ^{19}F NMR spectrum of **1d** (377 MHz; $CDCl_3$)

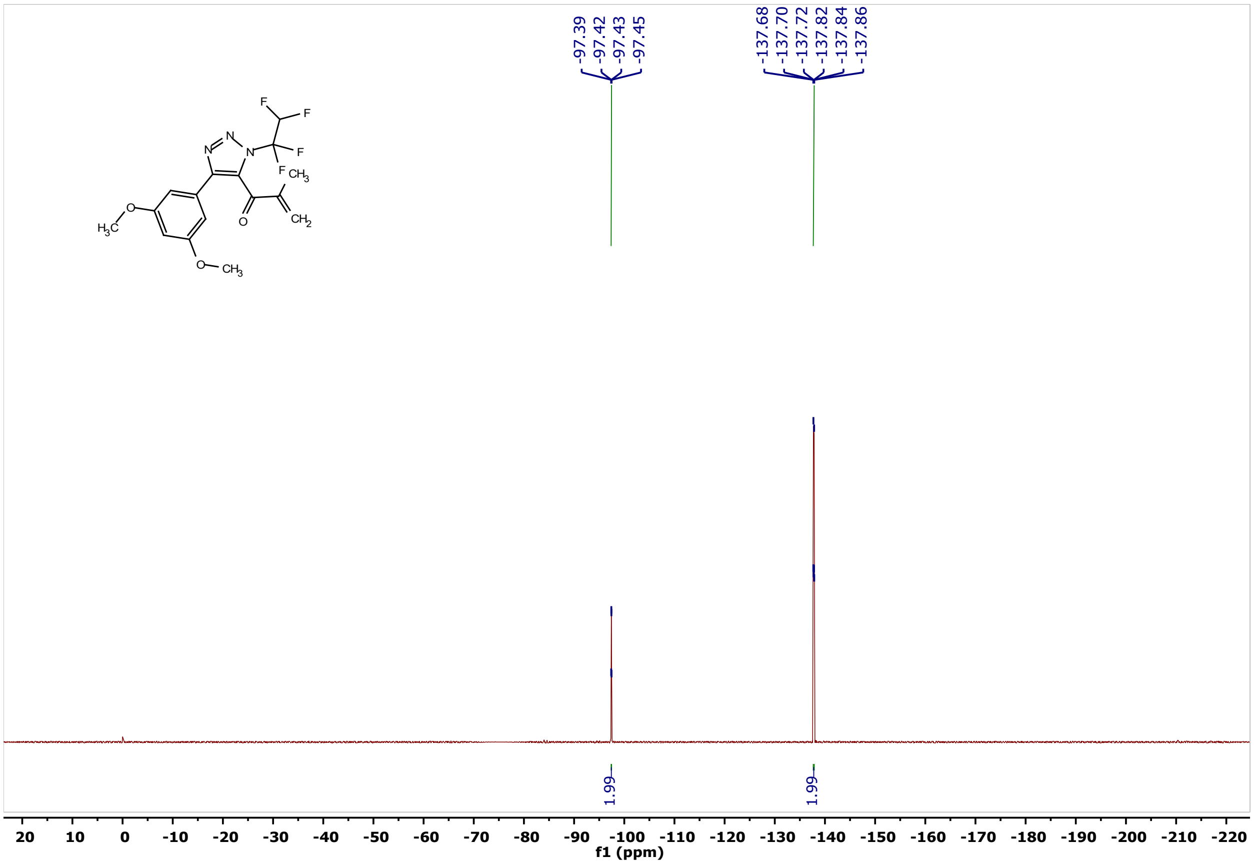

Figure S16. ^{1}H NMR spectrum of **1e** (401 MHz; $CDCl_3$)

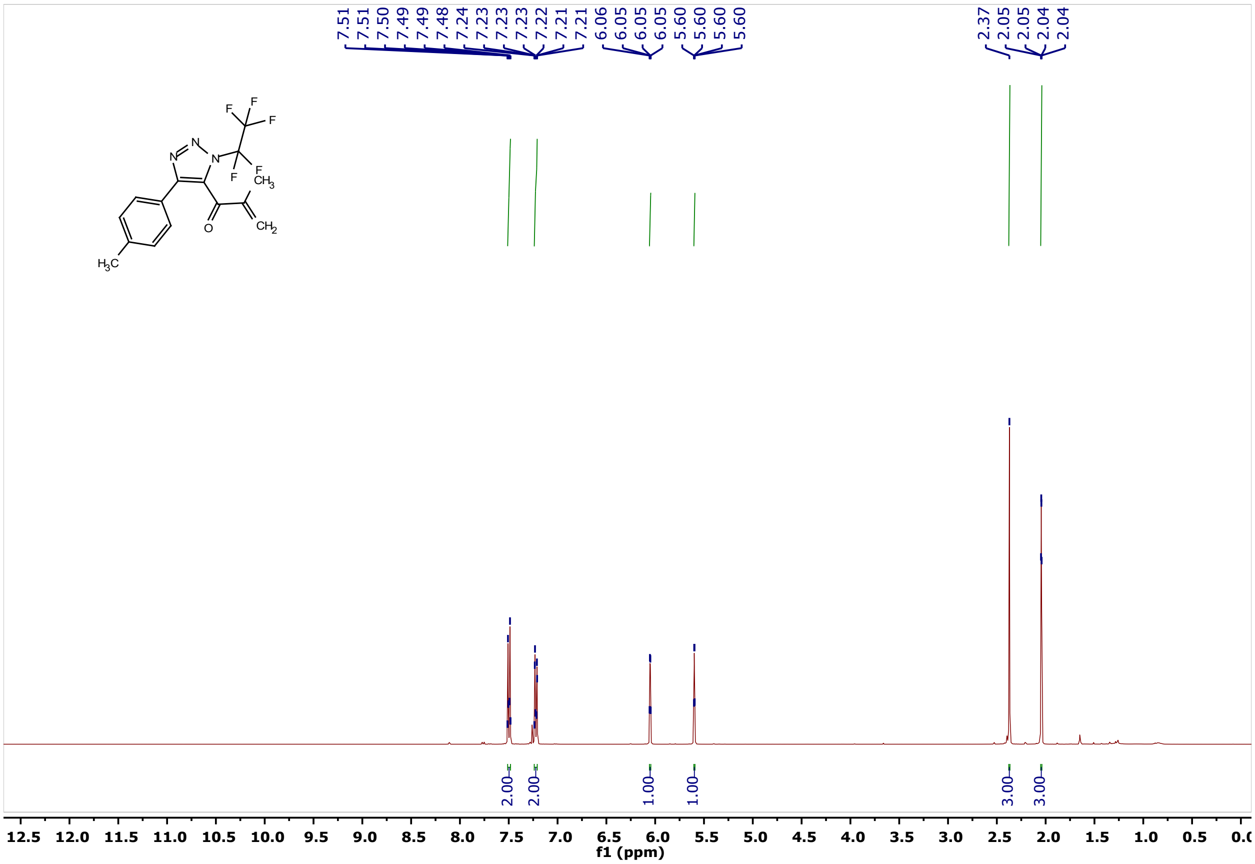

Figure S17. ^{13}C NMR spectrum of **1e** (101 MHz; $CDCl_3$)

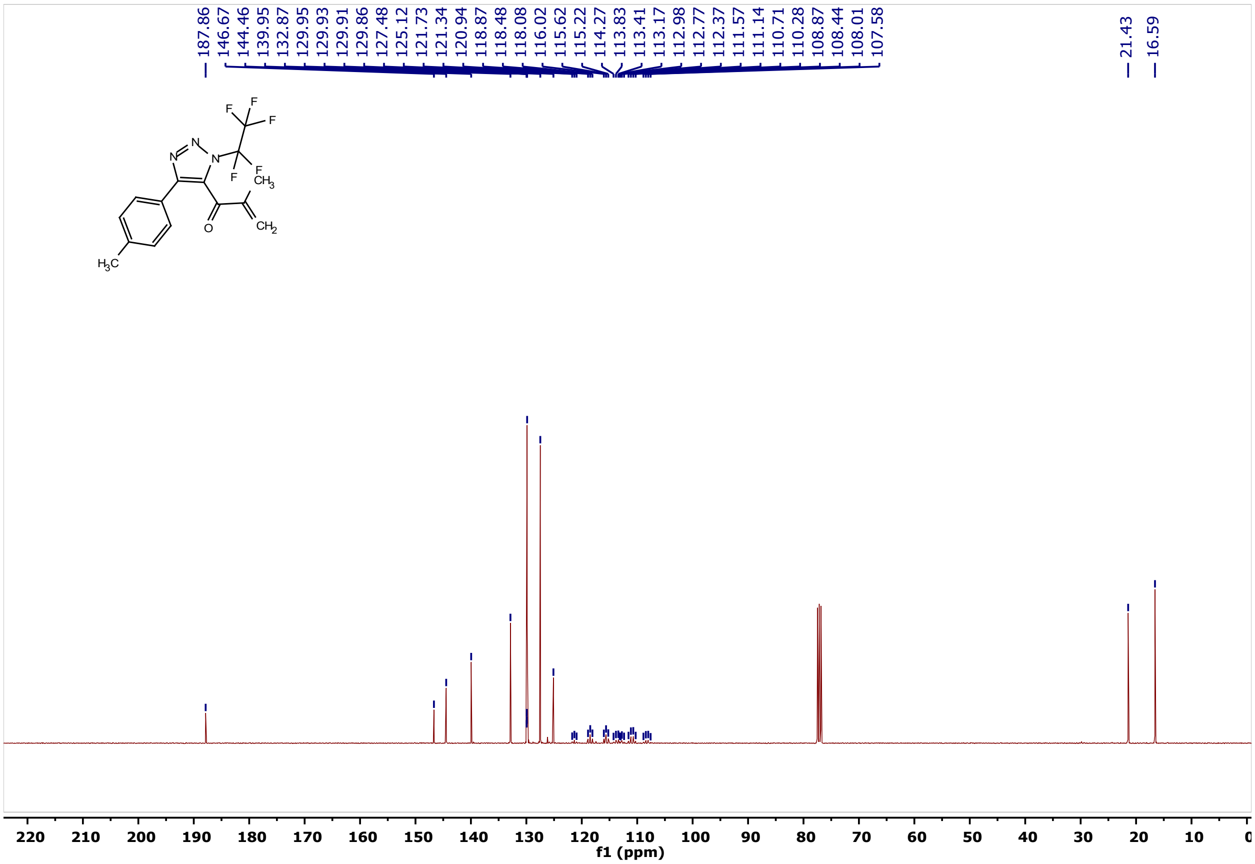

Figure S18. ^{19}F NMR spectrum of **1e** (377 MHz; $CDCl_3$)

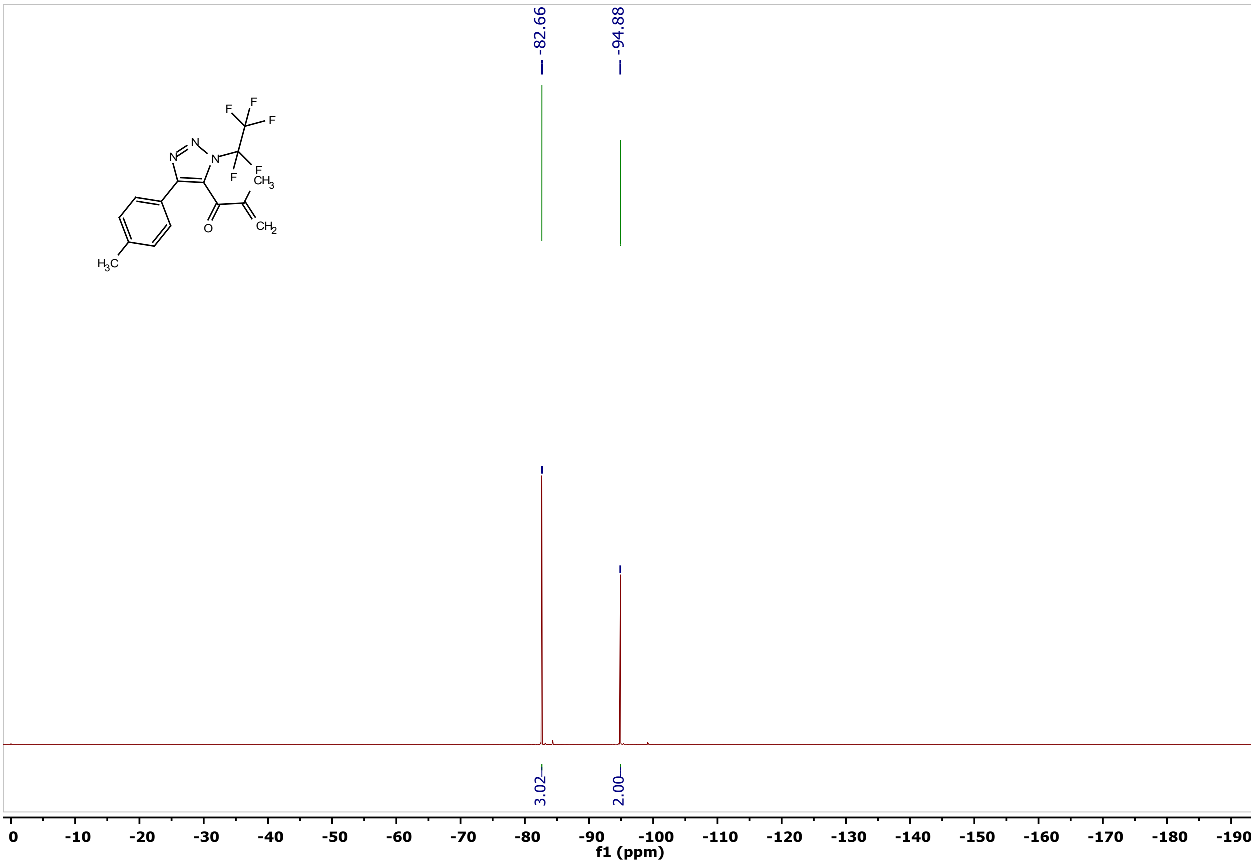

Figure S19. ^{1}H NMR spectrum of **1f** (401 MHz; $CDCl_3$)

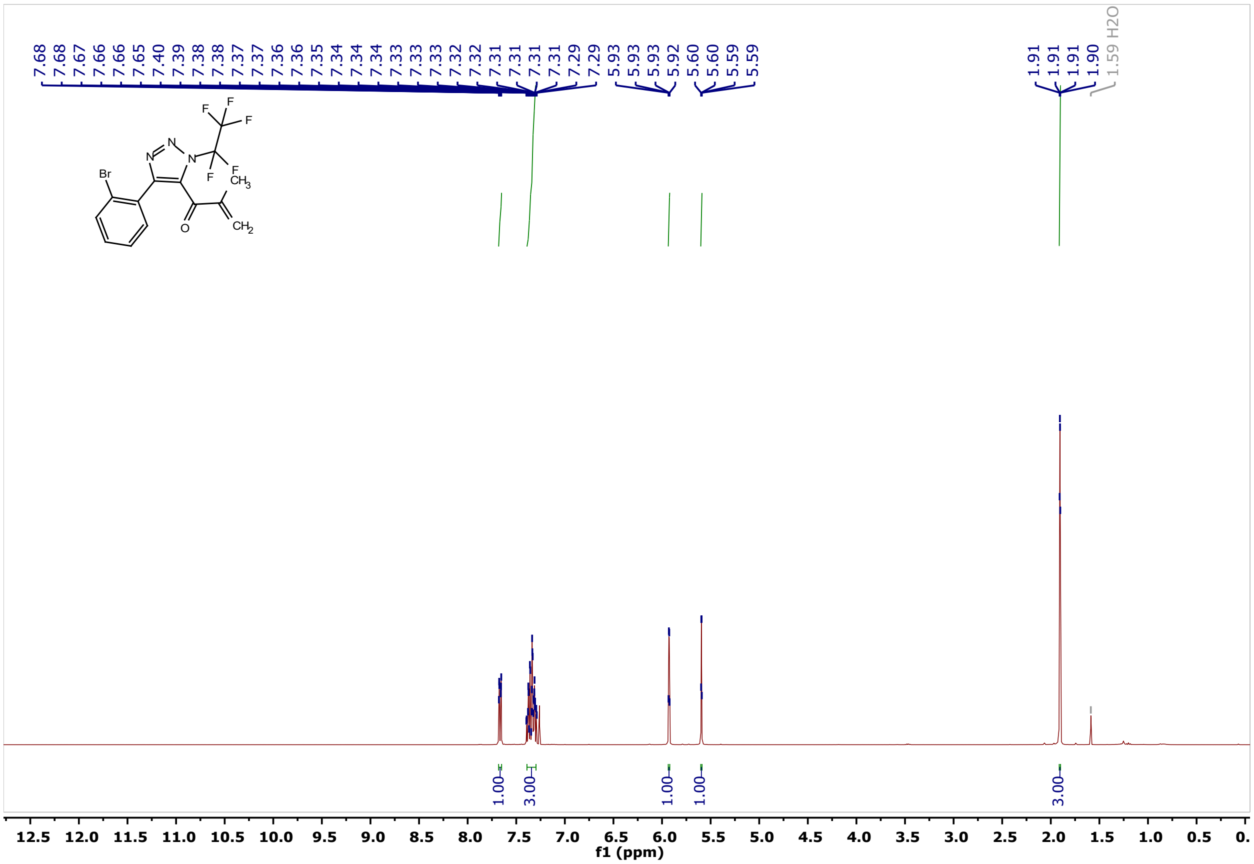

Figure S20. ^{13}C NMR spectrum of **1f** (101 MHz; $CDCl_3$)

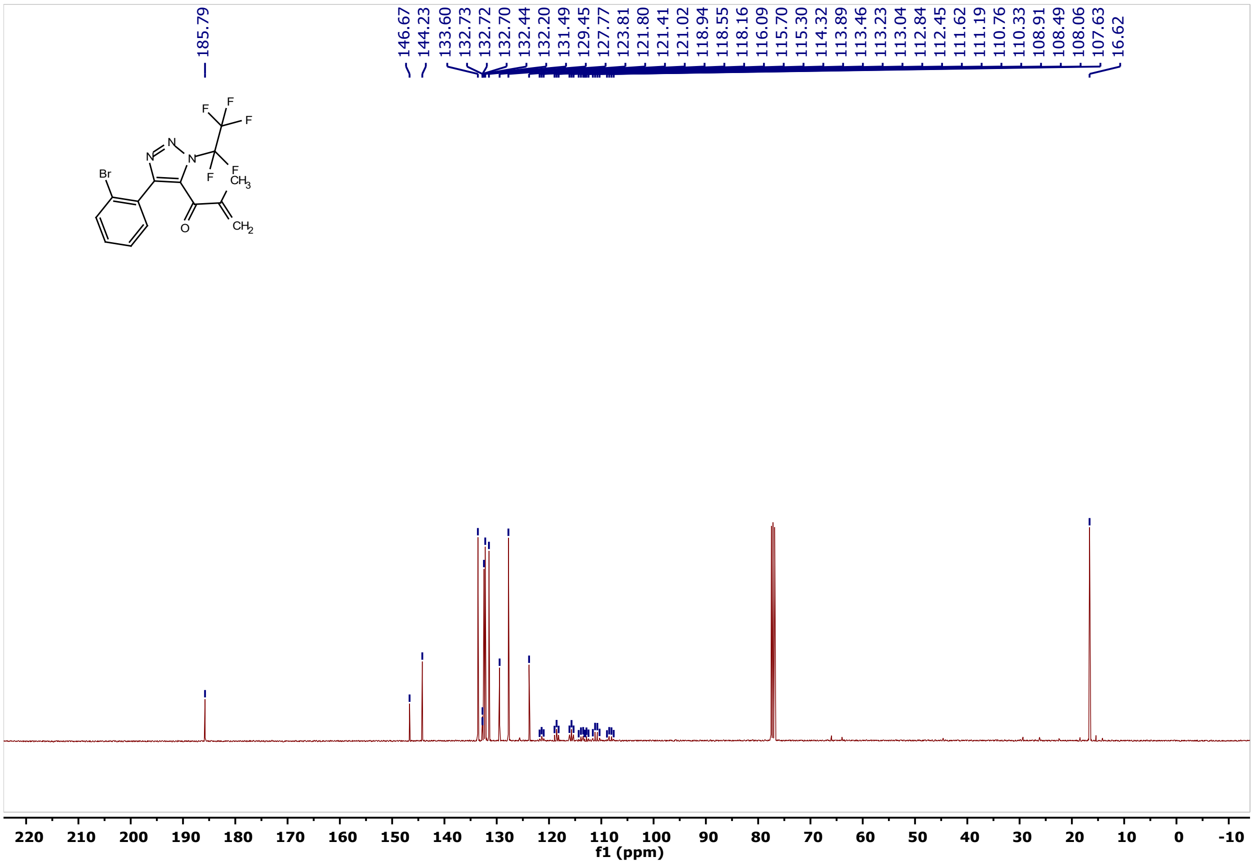

Figure S21. ^{19}F NMR spectrum of **1f** (377 MHz; $CDCl_3$)

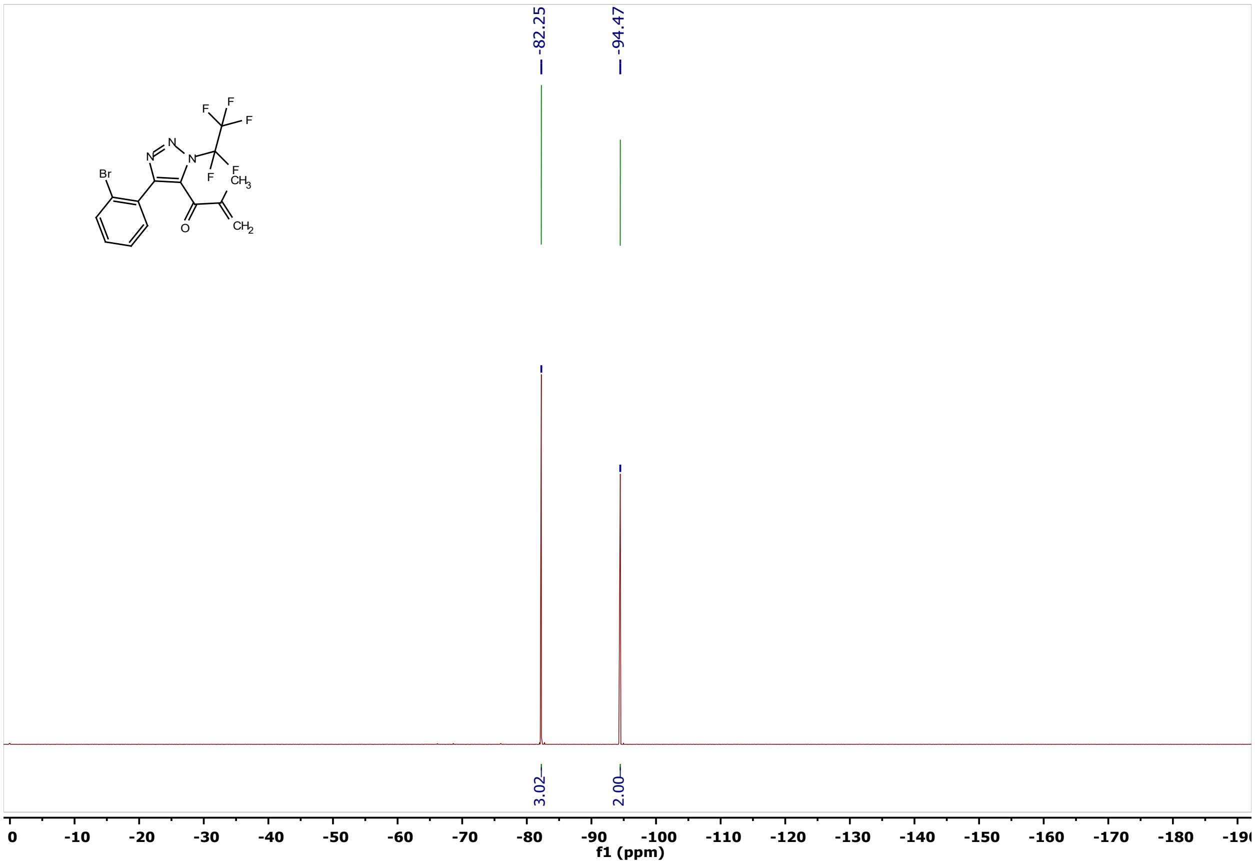

Figure S22. ^{1}H NMR spectrum of **1g** (401 MHz; $CDCl_3$)

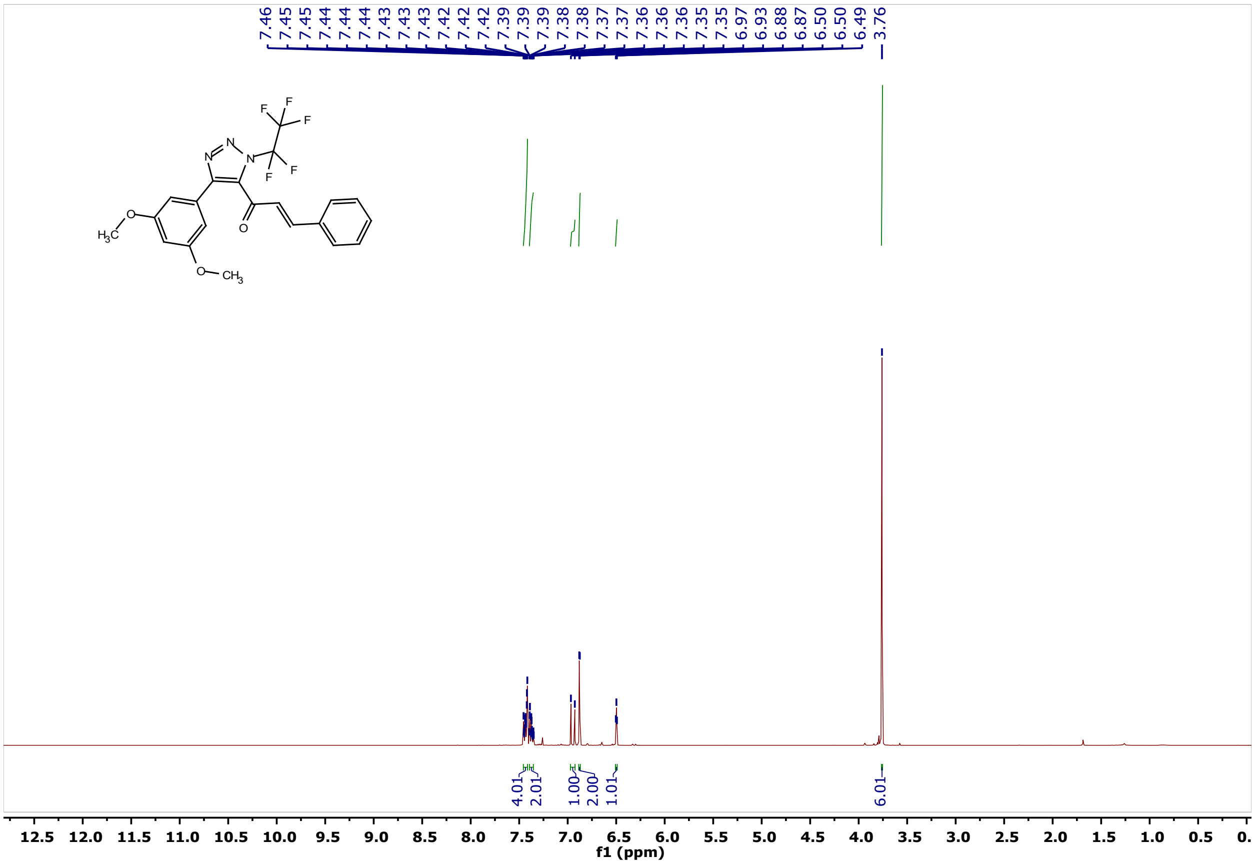

Figure S23. ^{13}C NMR spectrum of **1g** (101 MHz; $CDCl_3$)

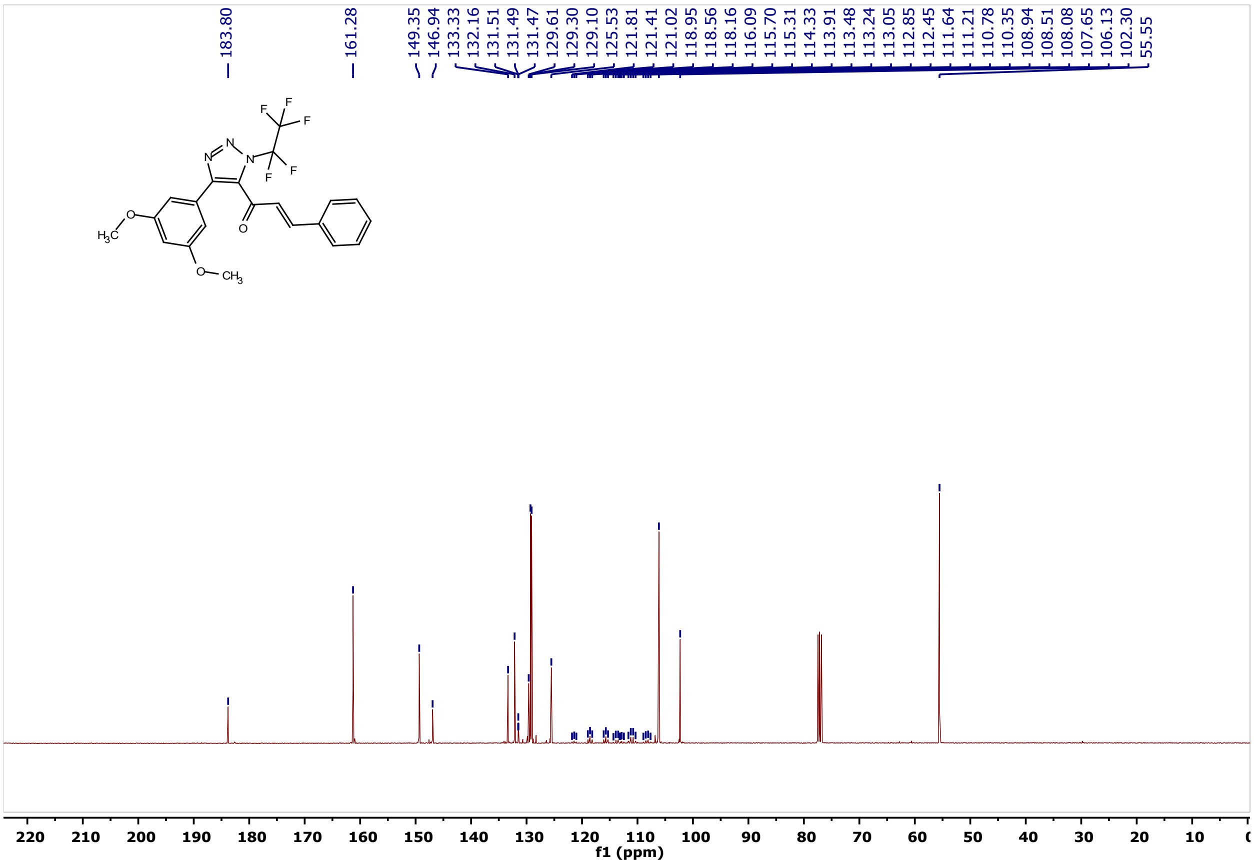

Figure S24. ^{19}F NMR spectrum of **1g** (377 MHz; $CDCl_3$)

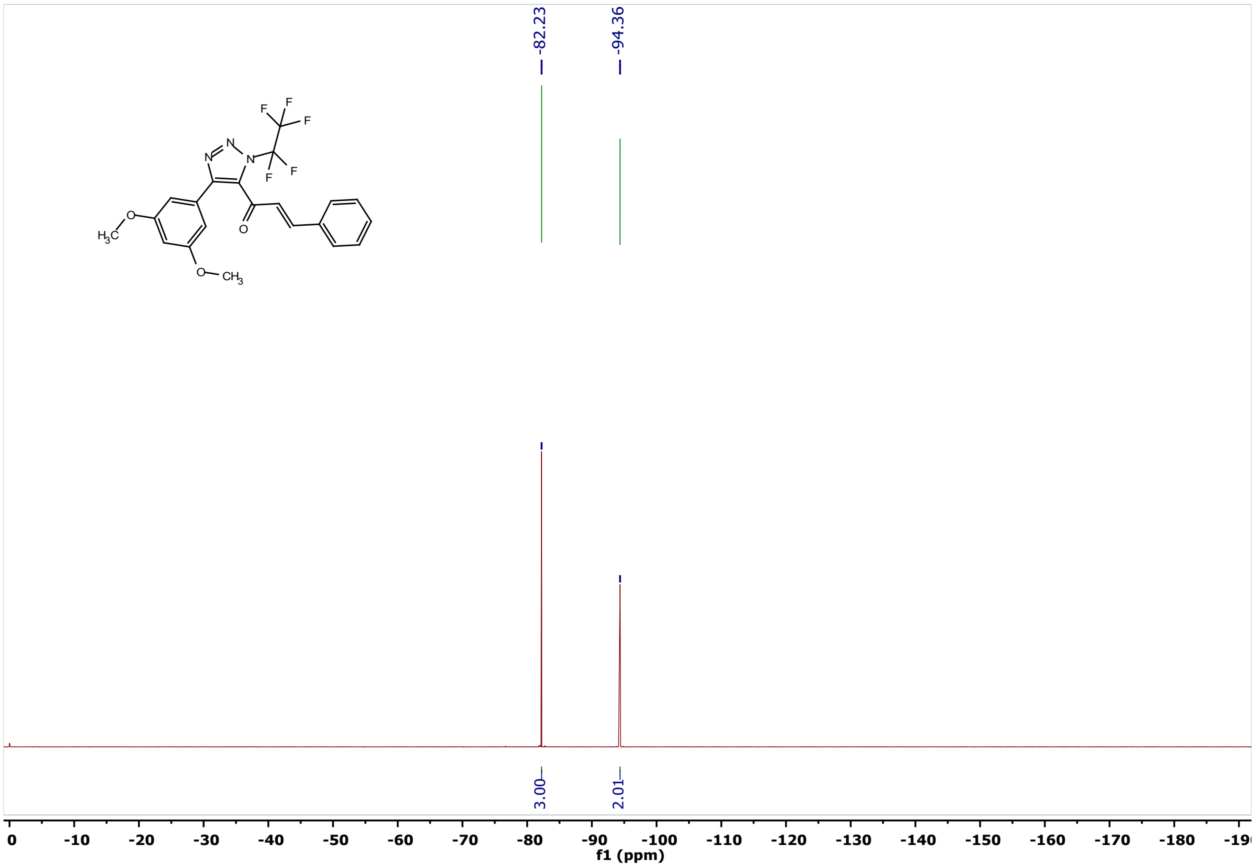

Figure S25. ^{1}H NMR spectrum of **1h** (401 MHz; $CDCl_3$)

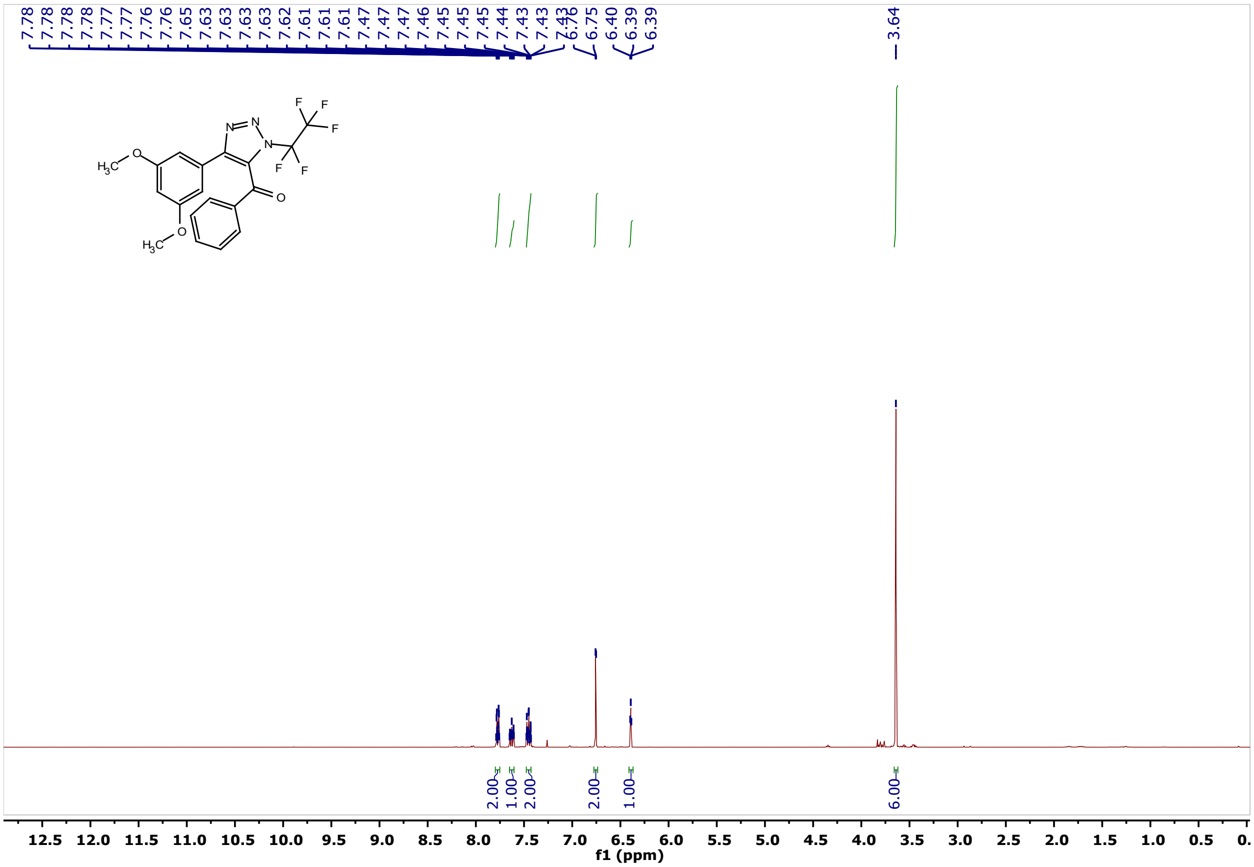

Figure S26. ^{13}C NMR spectrum of **1h** (101 MHz; $CDCl_3$)

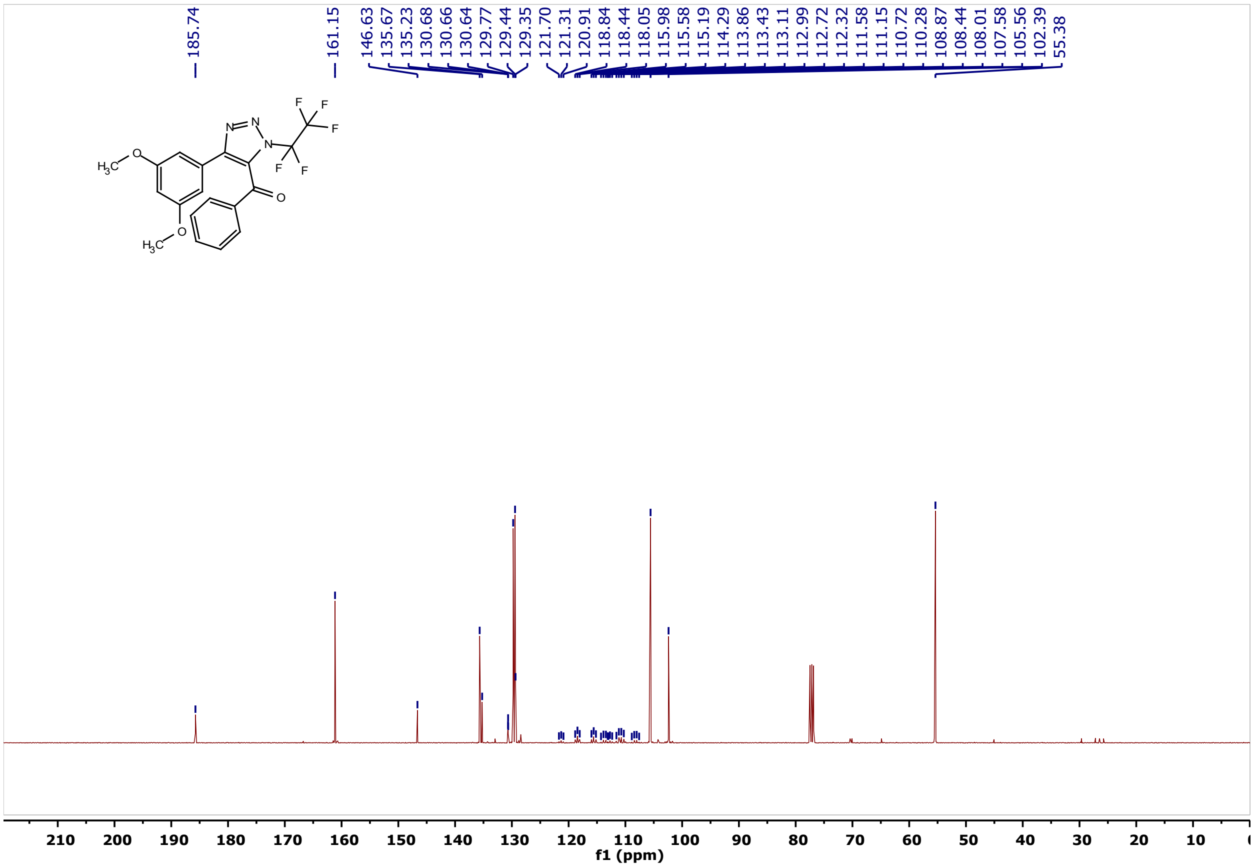

Figure S27. ^{19}F NMR spectrum of **1h** (377 MHz; $CDCl_3$)

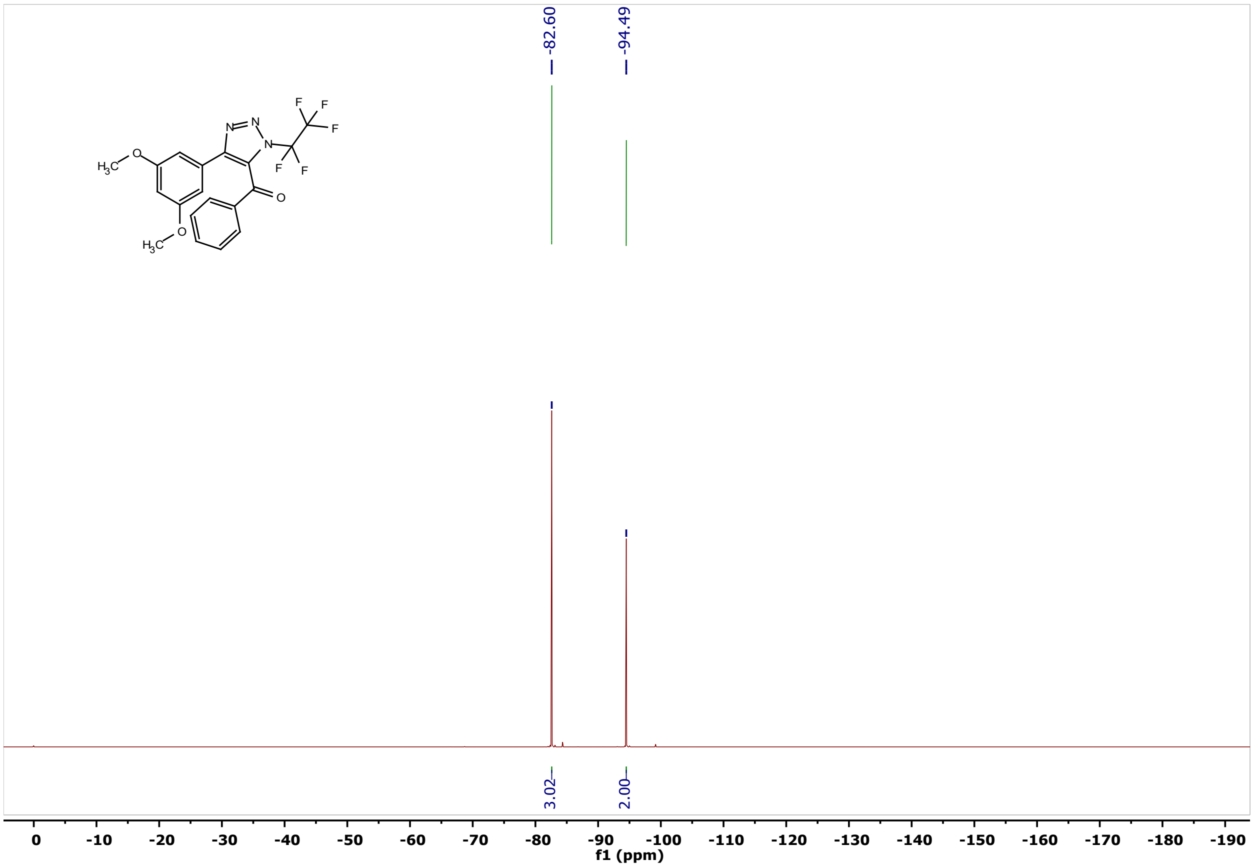

Figure 28. ^{1}H NMR spectrum of **1i** (401 MHz; $CDCl_3$)

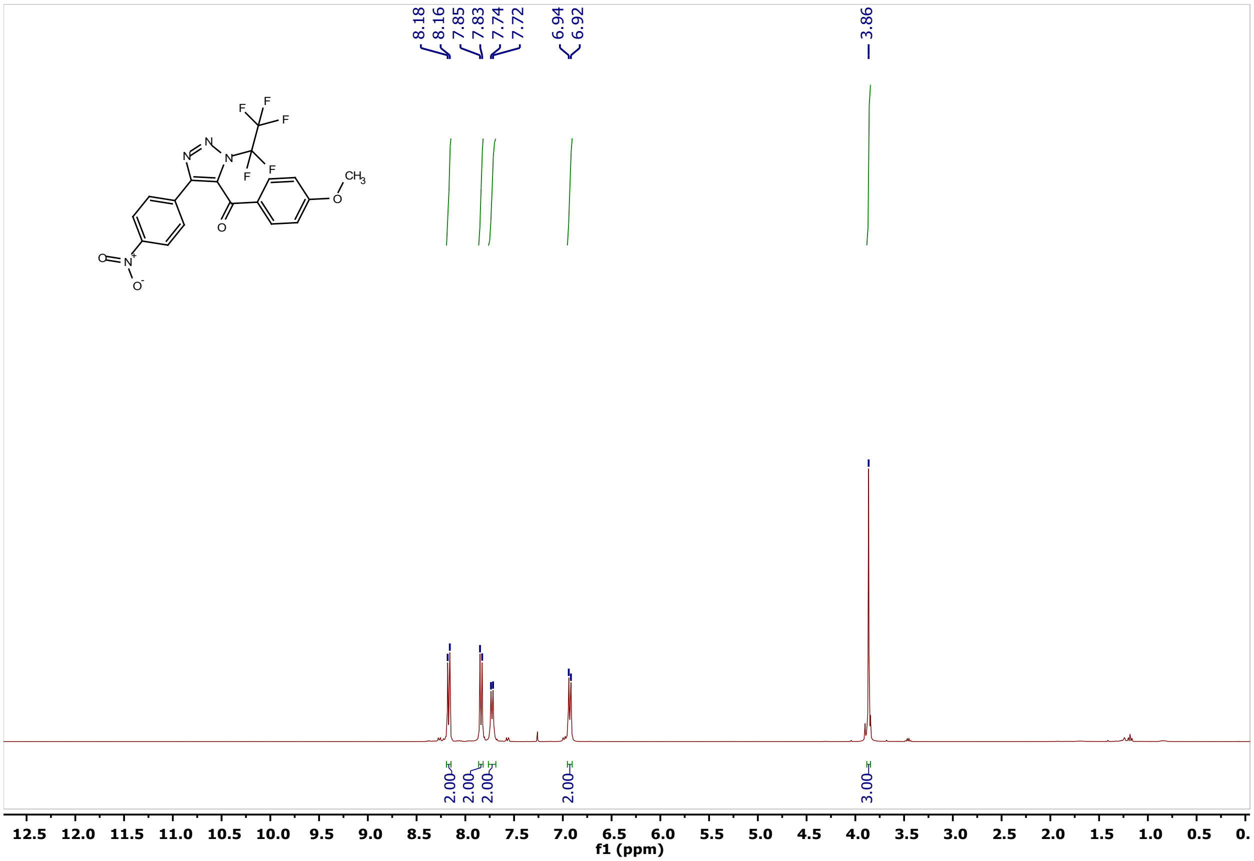

Figure 29. ^{13}C NMR spectrum of **1i** (101 MHz; $CDCl_3$)

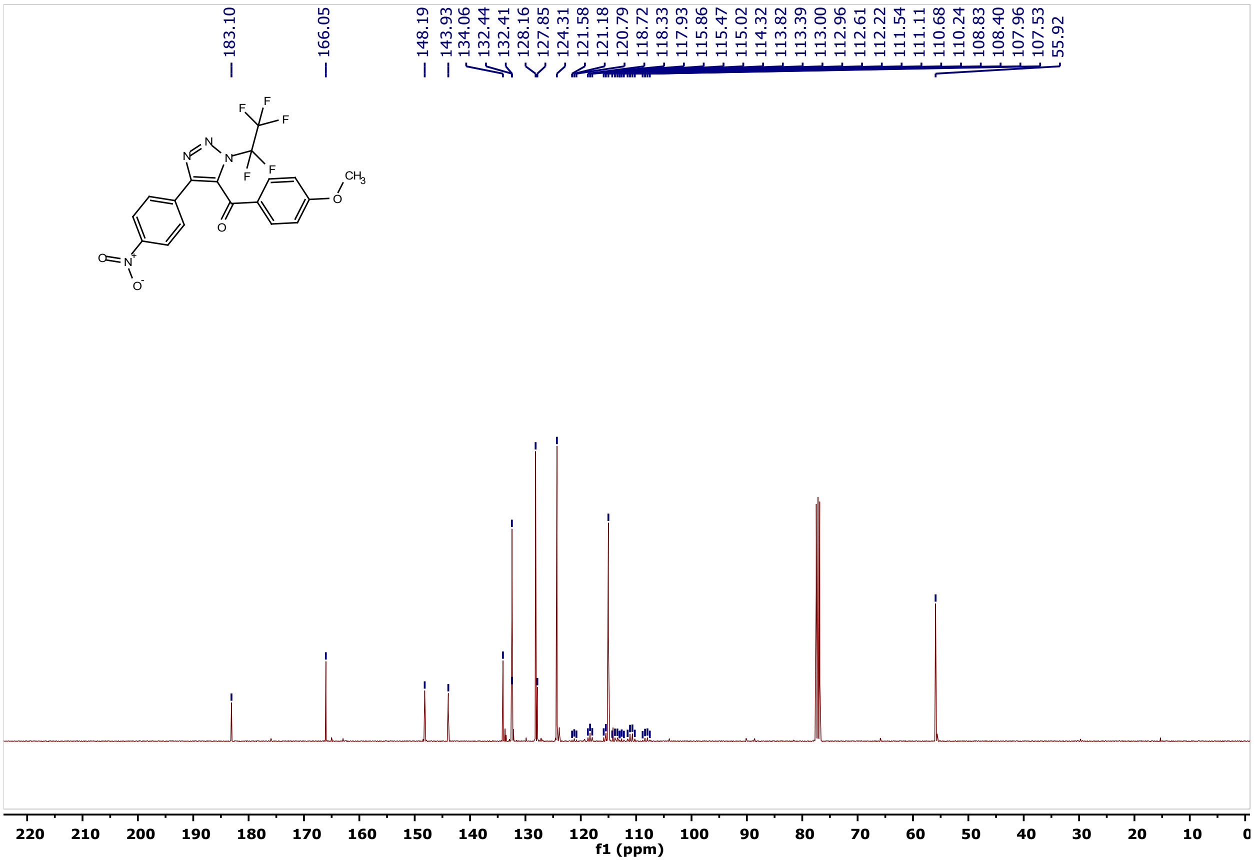

Figure 30. ^{19}F NMR spectrum of **1i** (377 MHz; $CDCl_3$)

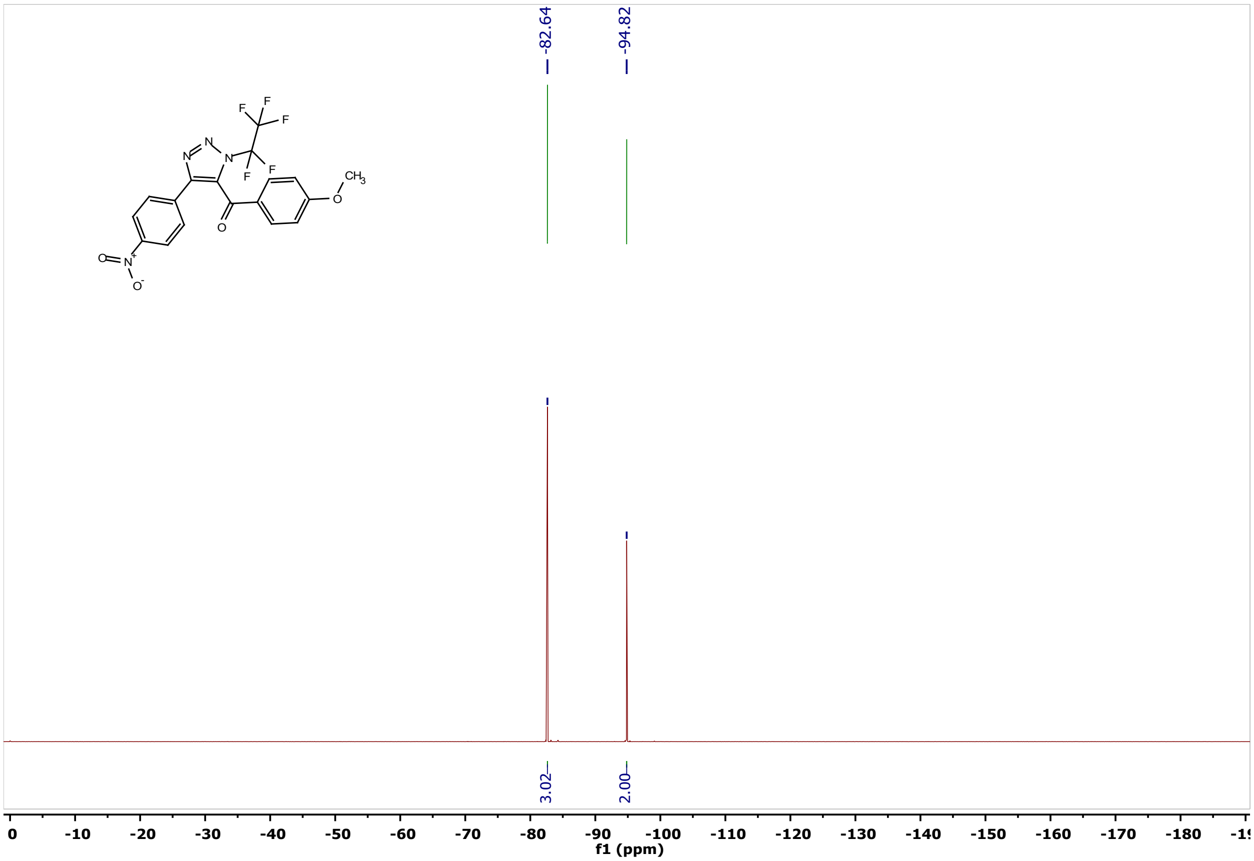

Figure S31. ^{1}H NMR spectrum of **1j** (401 MHz; $CDCl_3$)

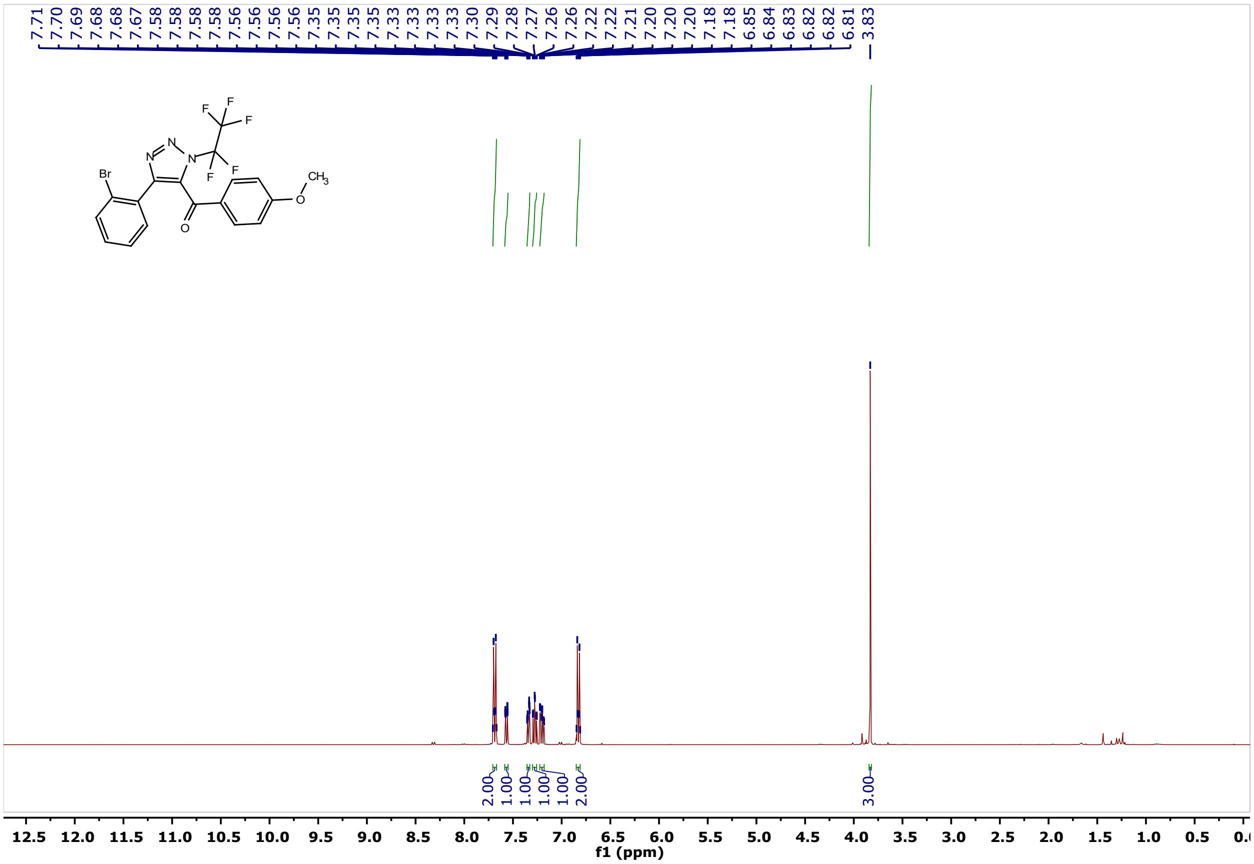

Figure S32. ^{13}C NMR spectrum of **1j** (101 MHz; $CDCl_3$)

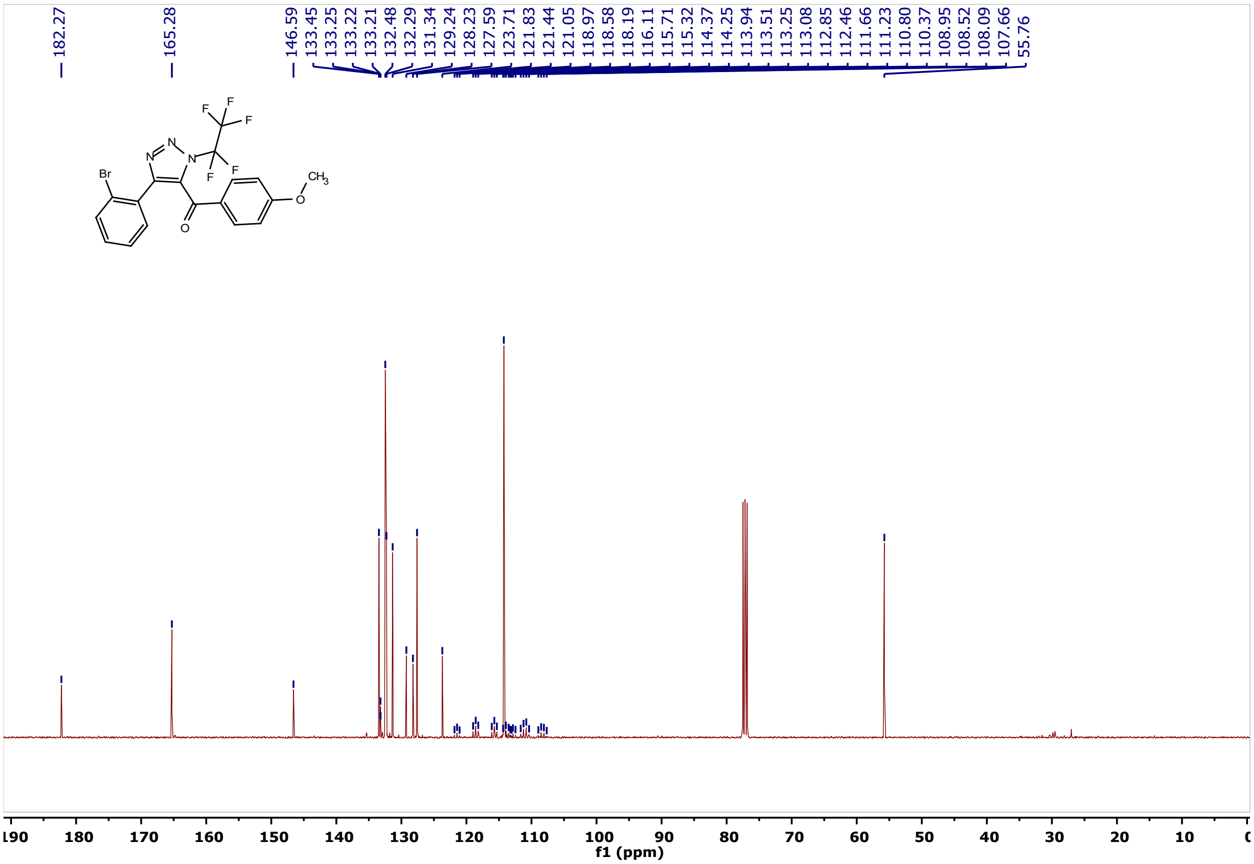

Figure S33. ^{19}F NMR spectrum of **1j** (377 MHz; $CDCl_3$)

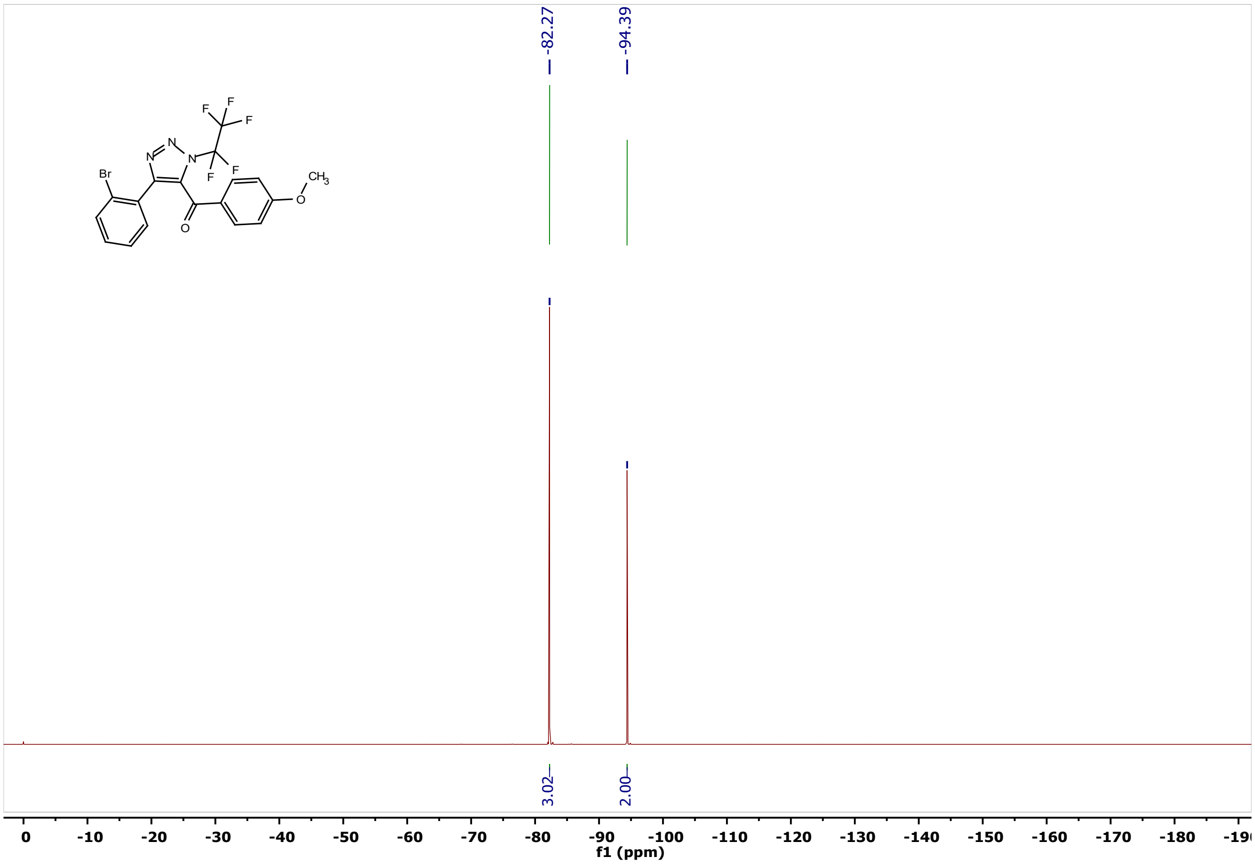

Figure S34. ^{1}H NMR spectrum of **1k** (401 MHz; $CDCl_3$)

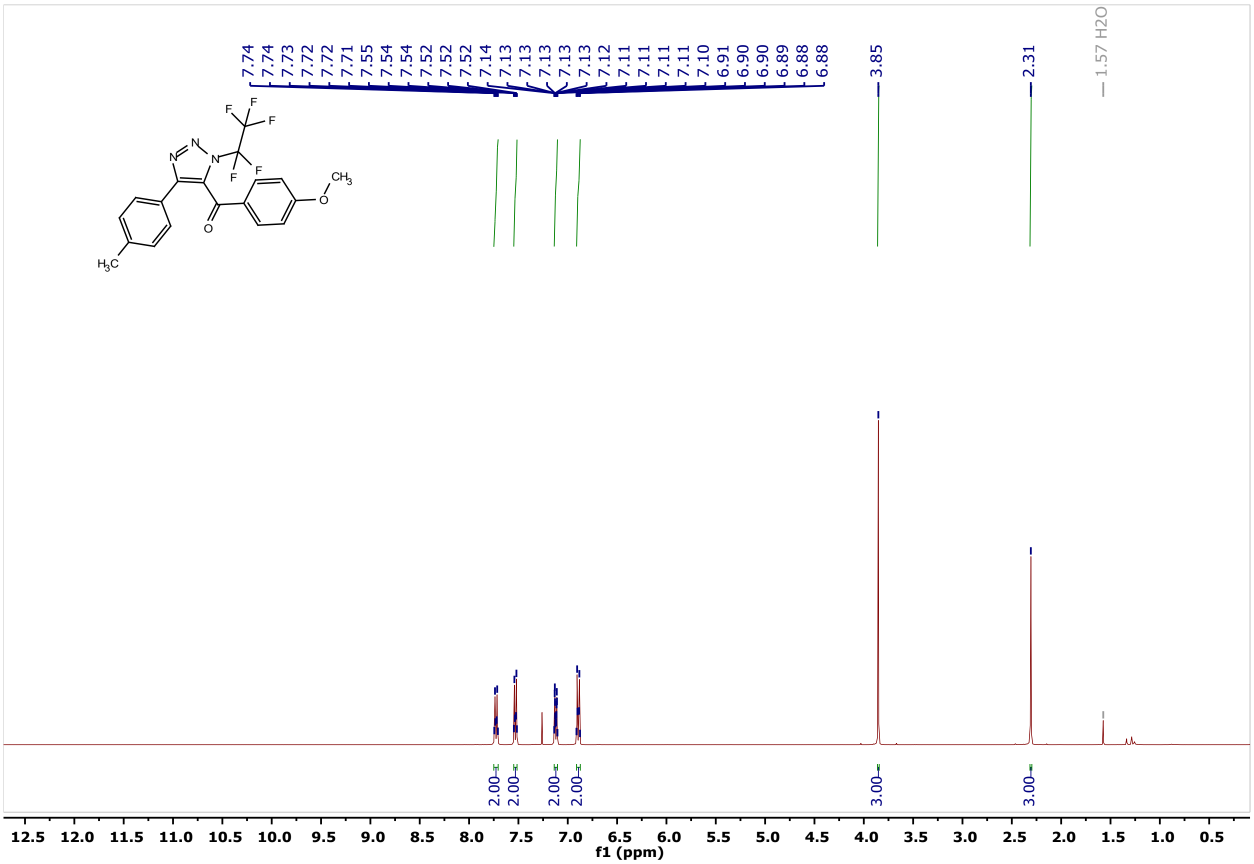

Figure S35. ^{13}C NMR spectrum of **1k** (101 MHz; $CDCl_3$)

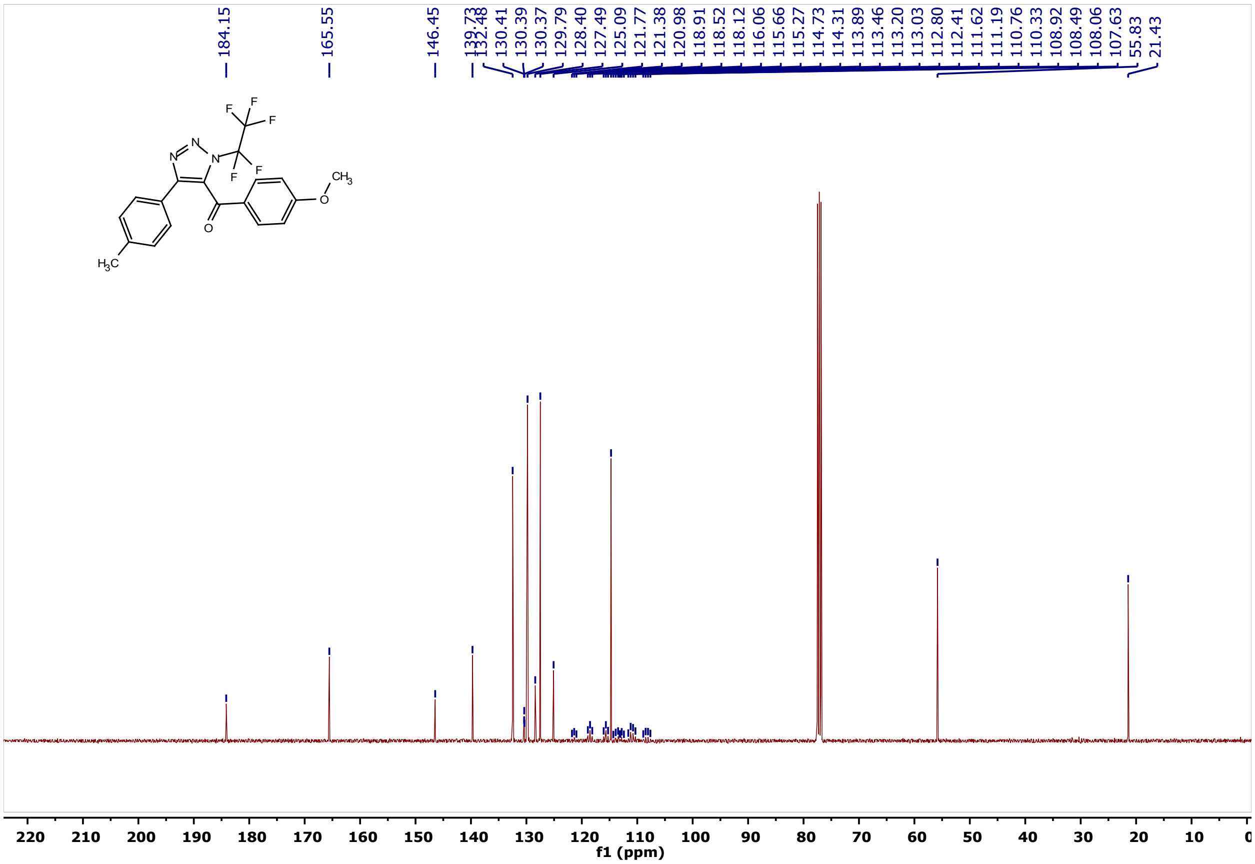

Figure S36. ^{19}F NMR spectrum of **1k** (377 MHz; $CDCl_3$)

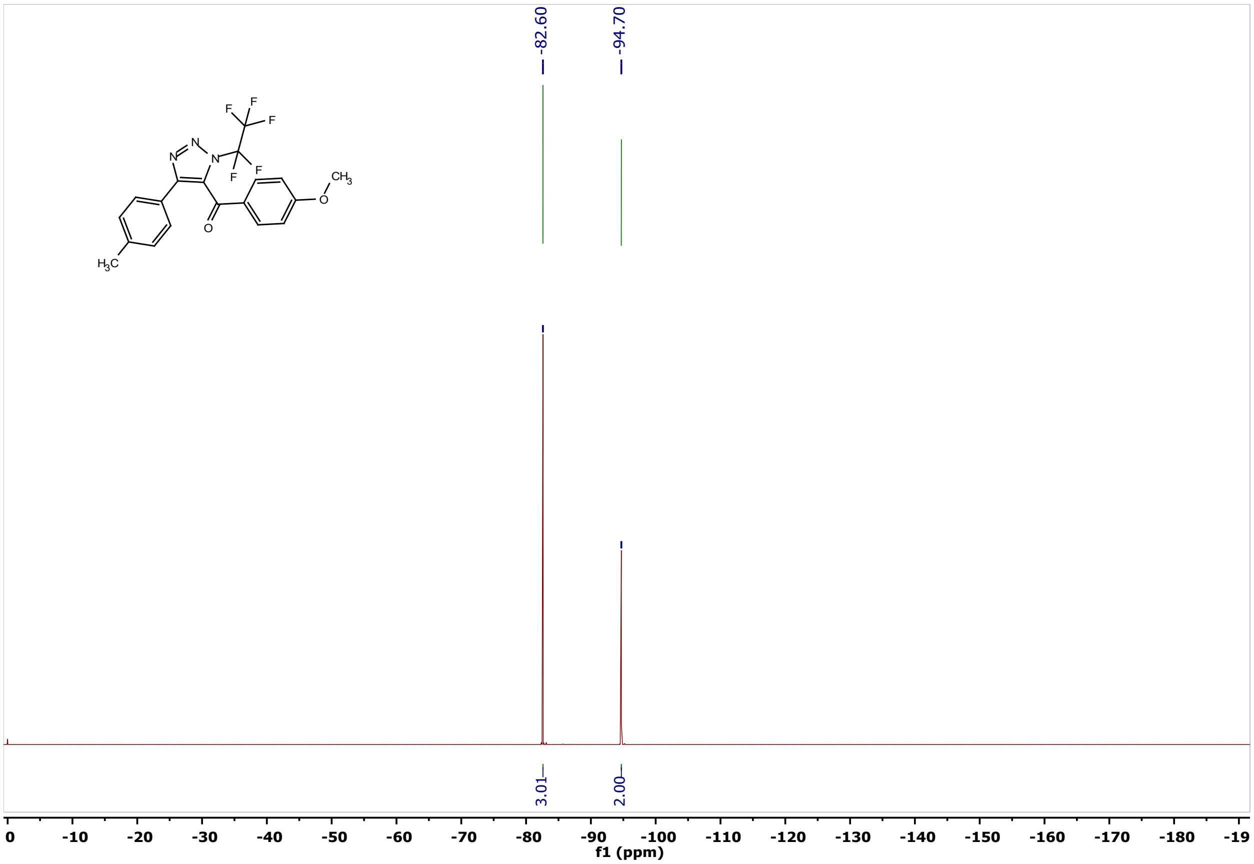

Figure S37. ^{1}H NMR spectrum of **1l** (401 MHz; $CDCl_3$)

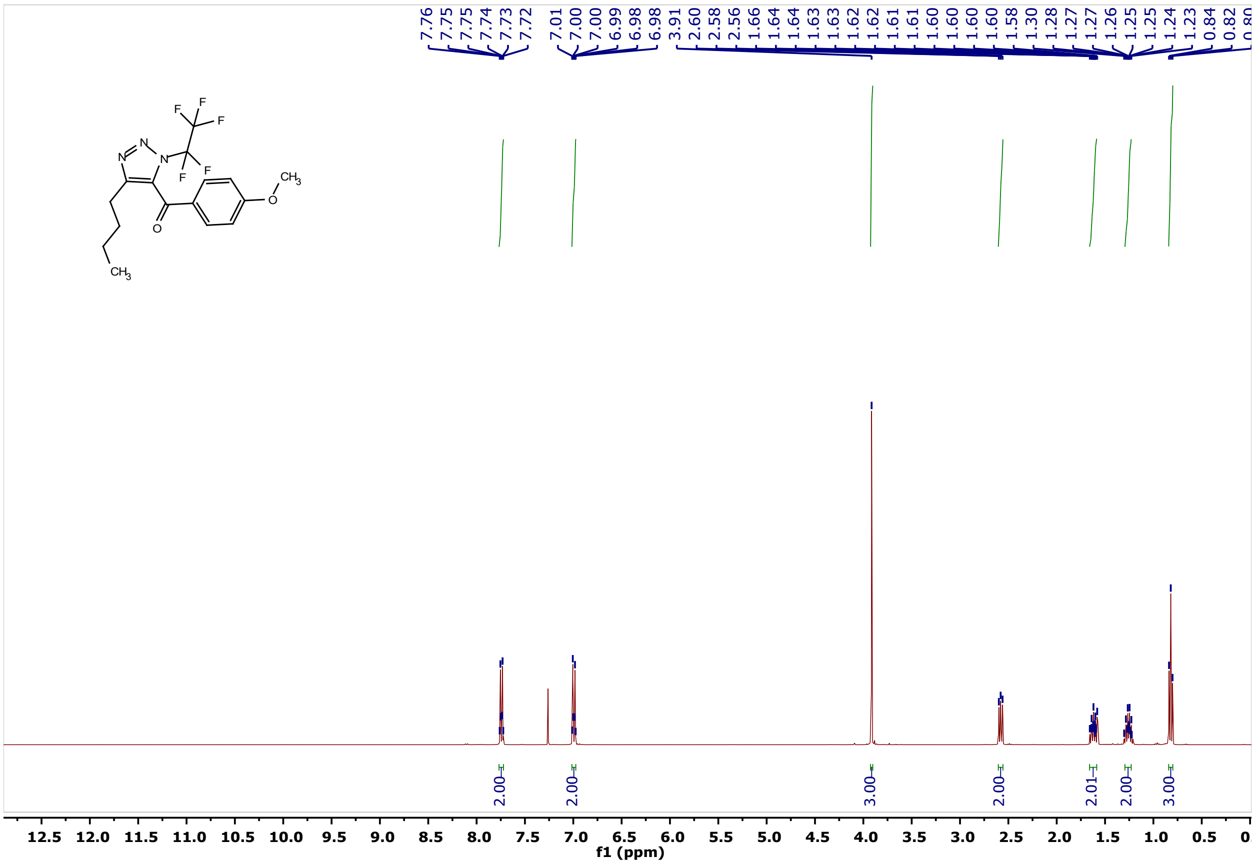

Figure S38. ^{13}C NMR spectrum of **1l** (101 MHz; $CDCl_3$)

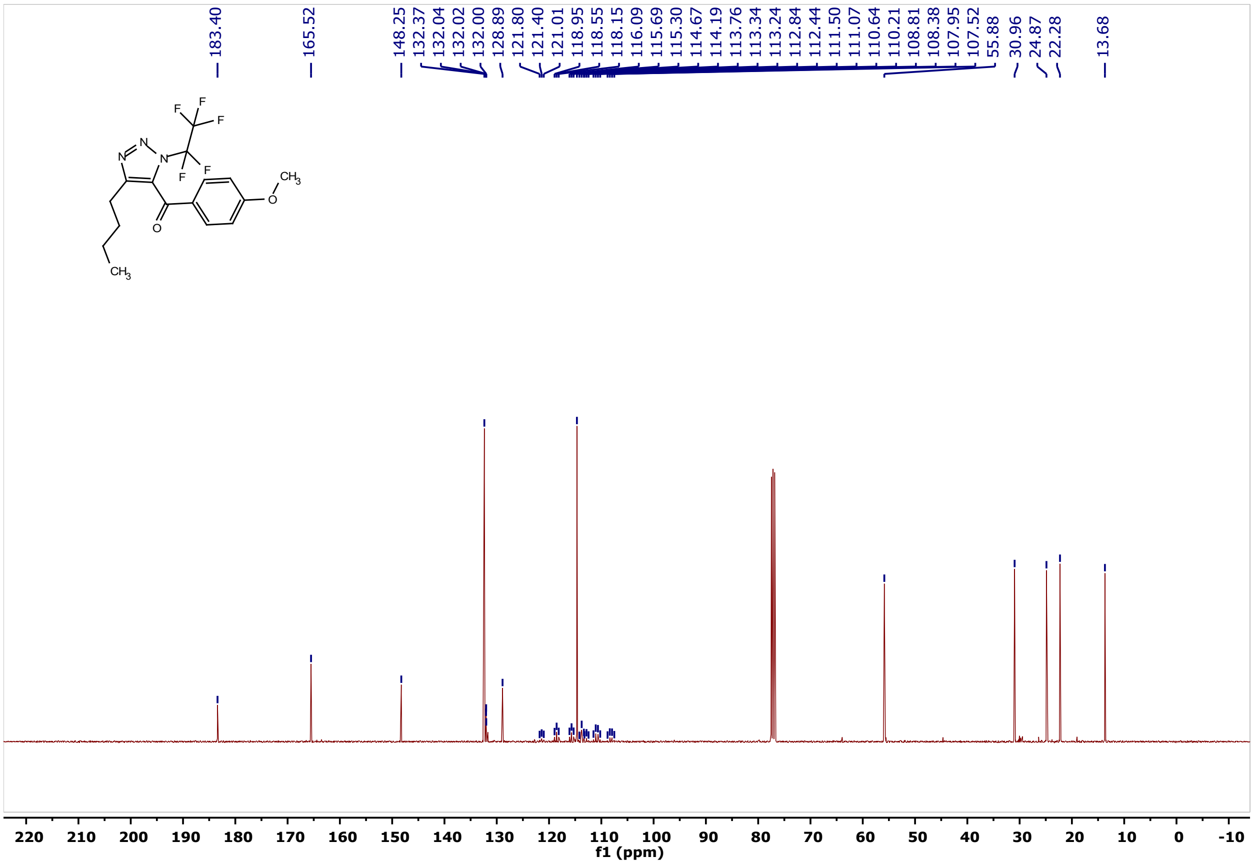

Figure S39. ^{19}F NMR spectrum of **1l** (377 MHz; $CDCl_3$)

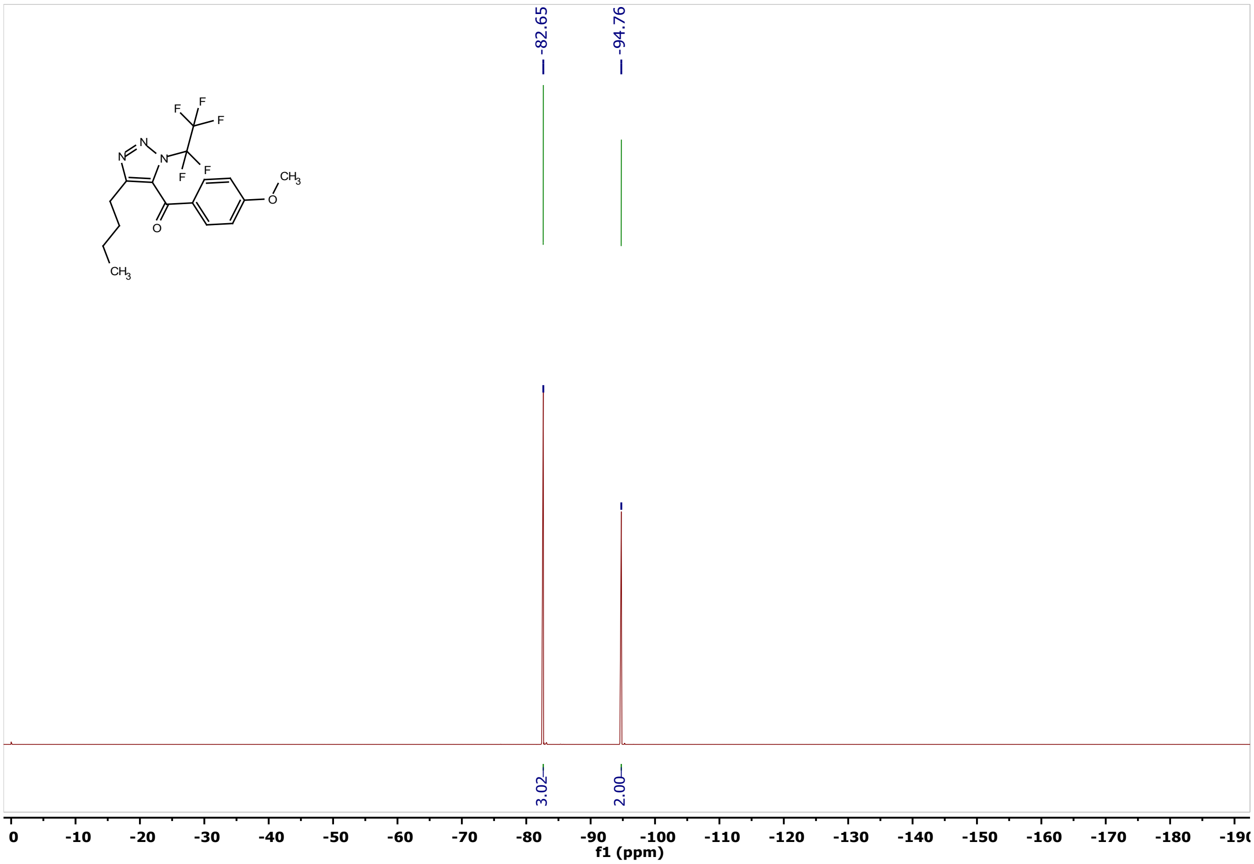

Figure S40. ^{1}H NMR spectrum of **1m** (401 MHz; $CDCl_3$)

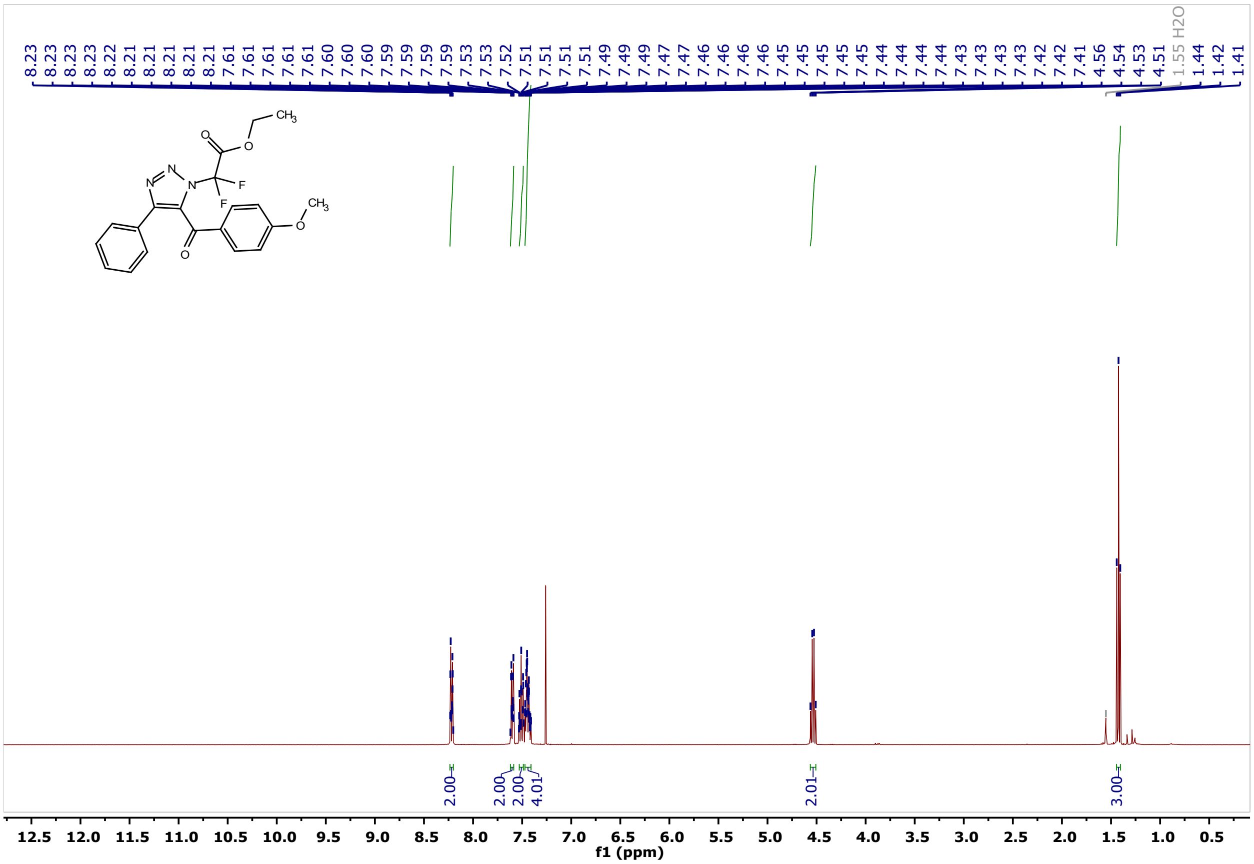

Figure S41. ^{13}C NMR spectrum of **1m** (101 MHz; $CDCl_3$)

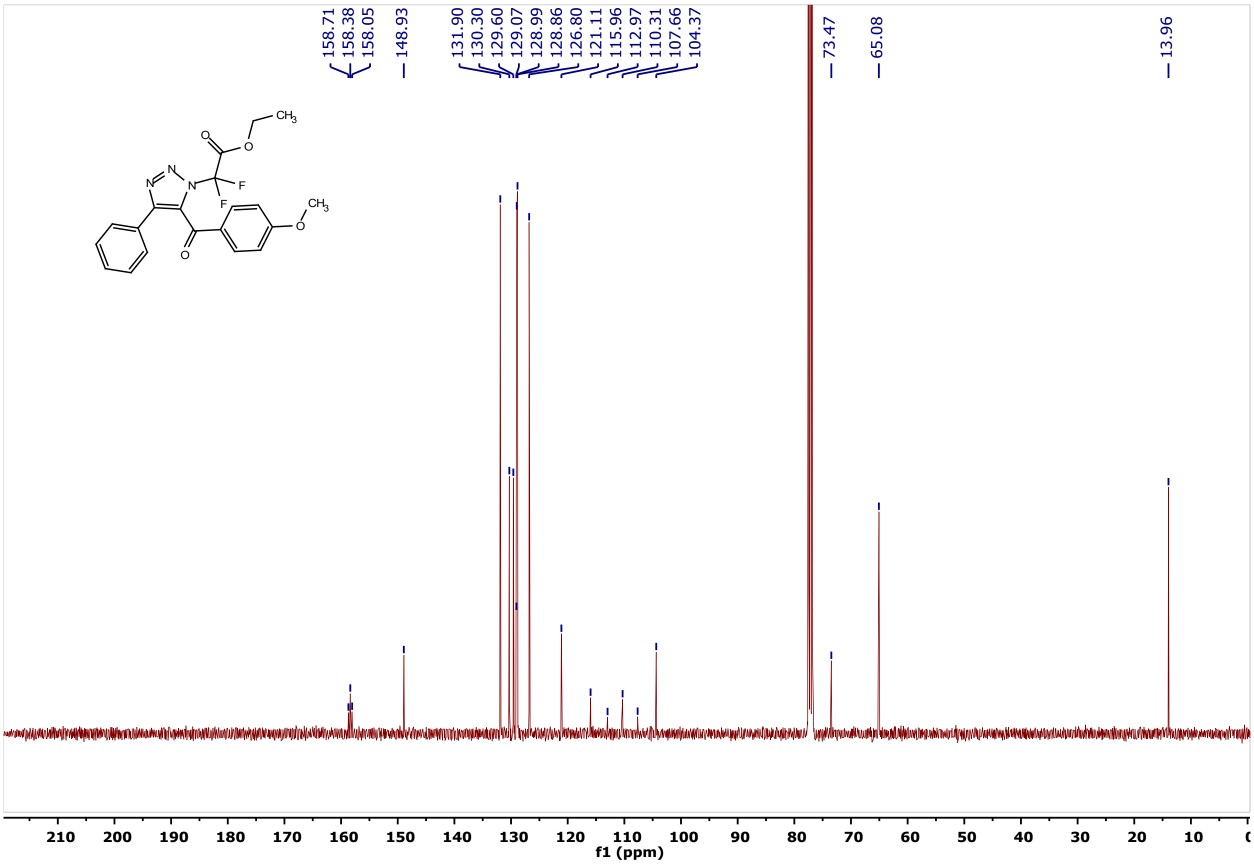

Figure S42. ^{19}F NMR spectrum of **1m** (377 MHz; $CDCl_3$)

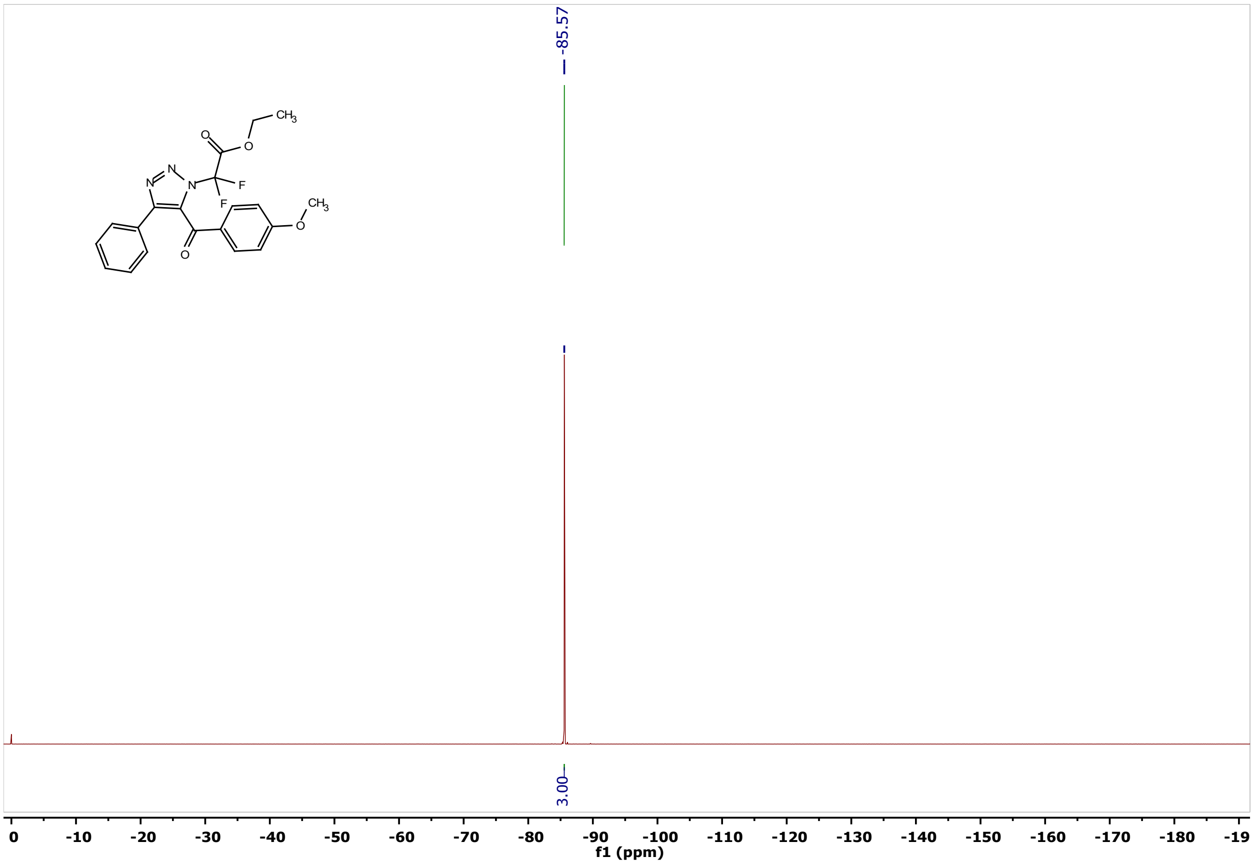

Figure S43. ^{1}H NMR spectrum of **1n** (401 MHz; $CDCl_3$)

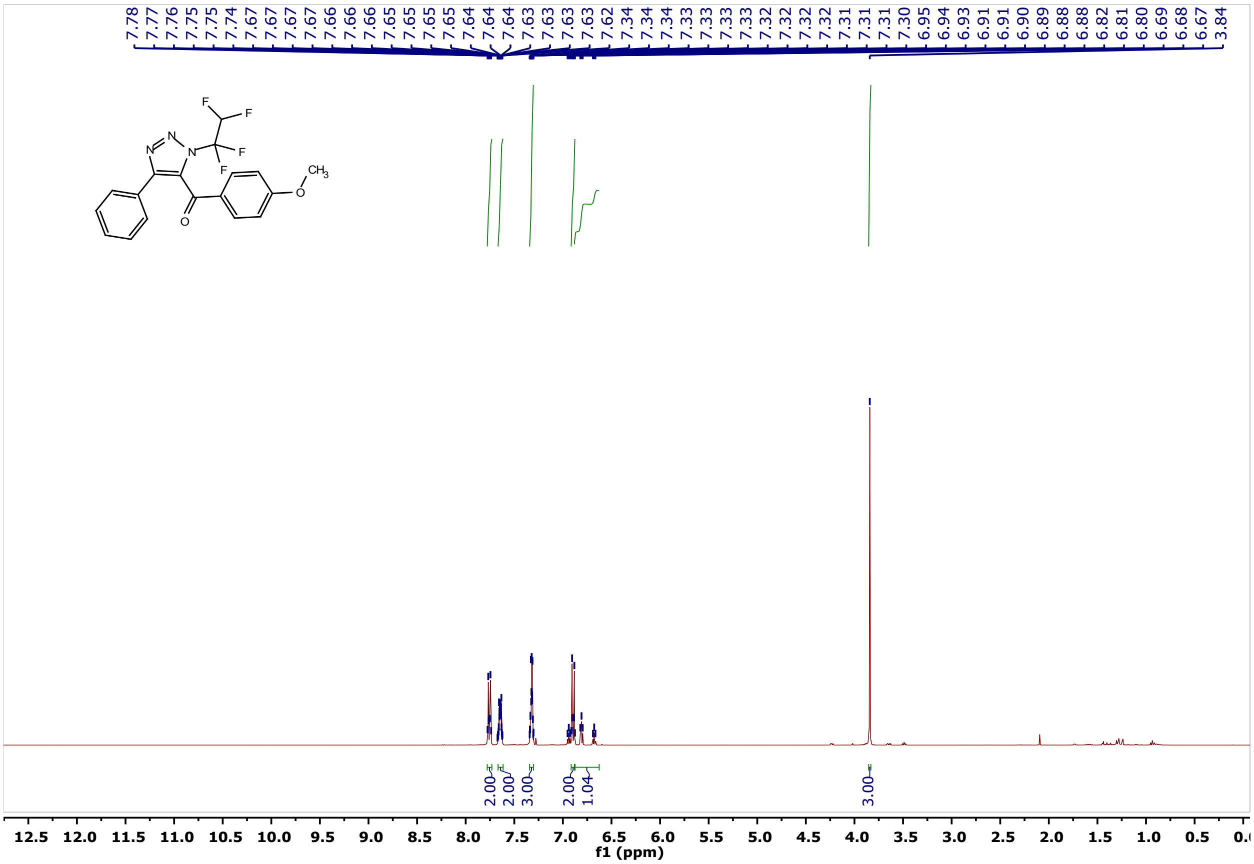

Figure S44. ^{13}C NMR spectrum of **1n** (101 MHz; $CDCl_3$)

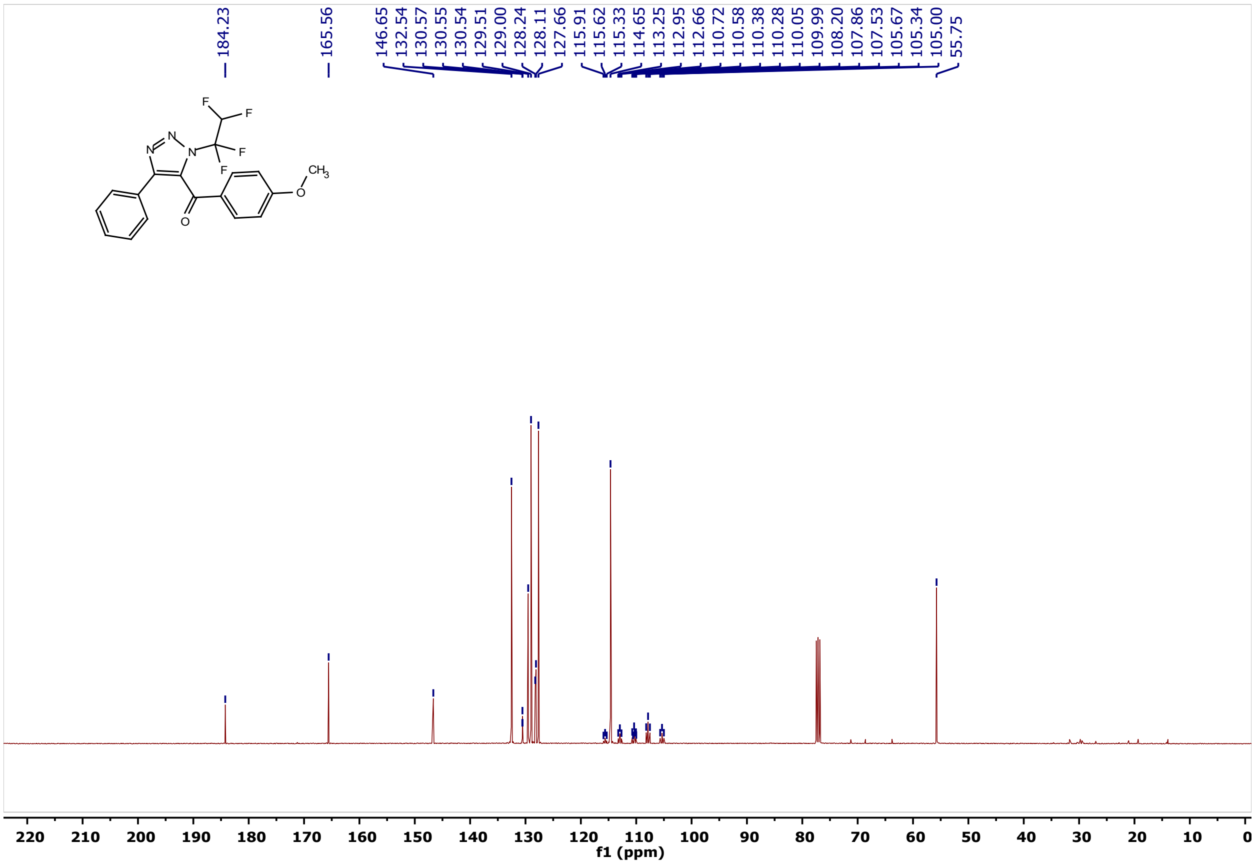

Figure S45. ^{19}F NMR spectrum of **1n** (377 MHz; $CDCl_3$)

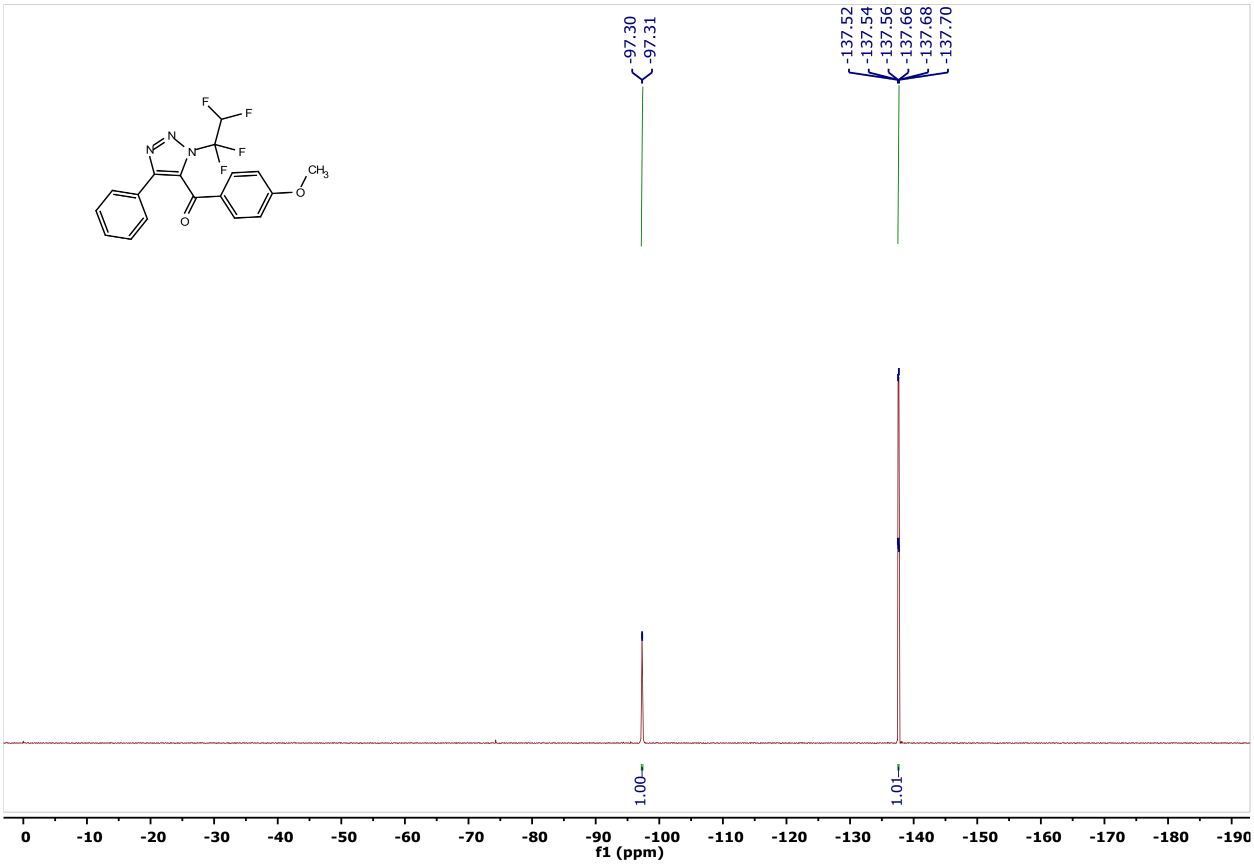

Figure S46. ^{1}H NMR spectrum of **1o** (401 MHz; $CDCl_3$)

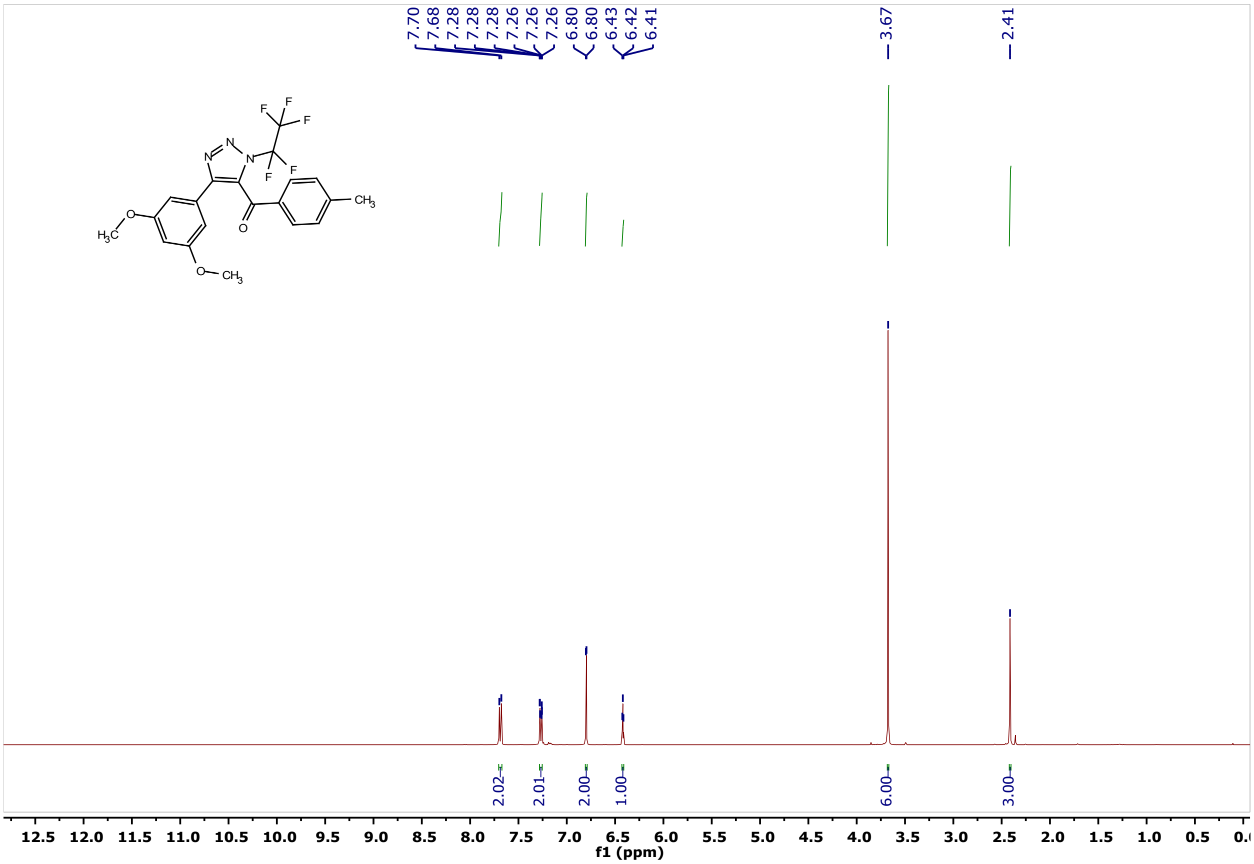

Figure S47. ^{13}C NMR spectrum of **1o** (101 MHz; $CDCl_3$)

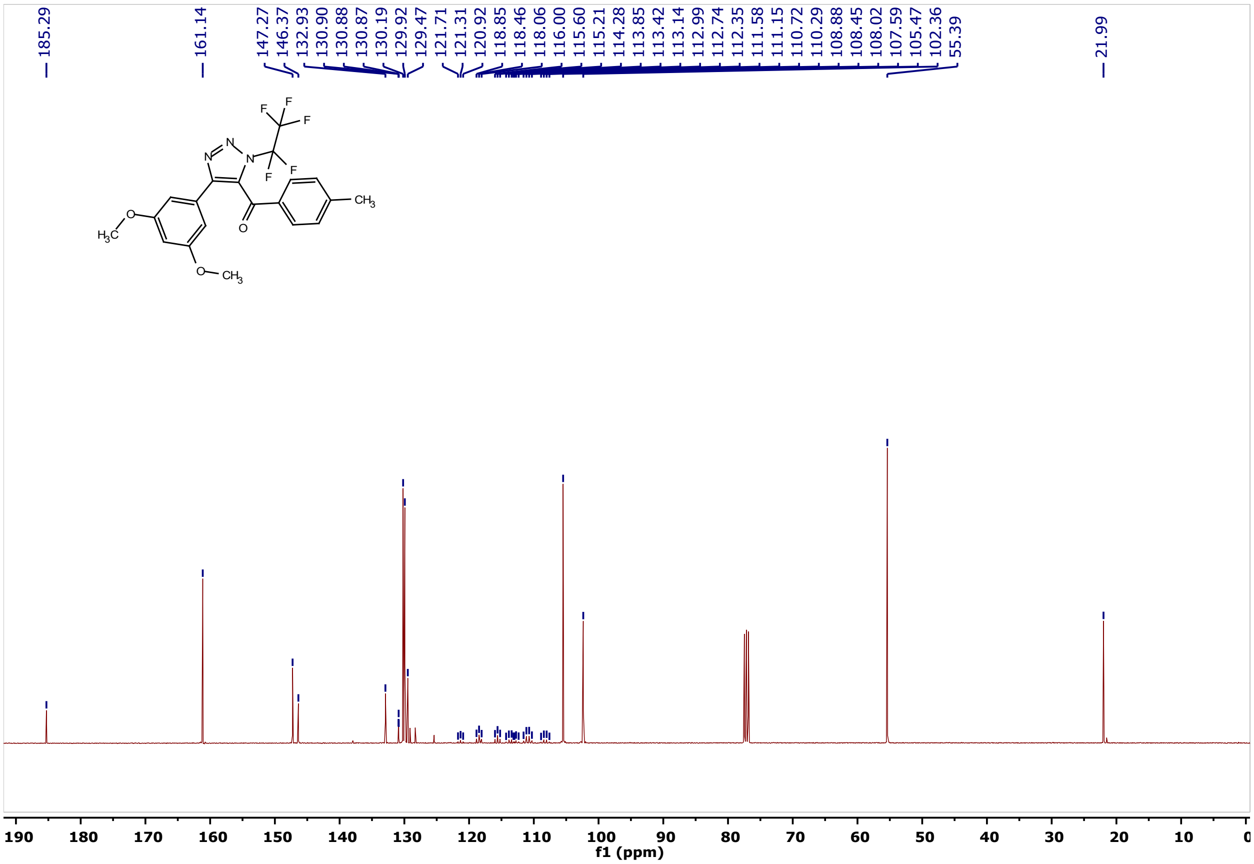

Figure S48. ^{19}F NMR spectrum of **1o** (377 MHz; $CDCl_3$)

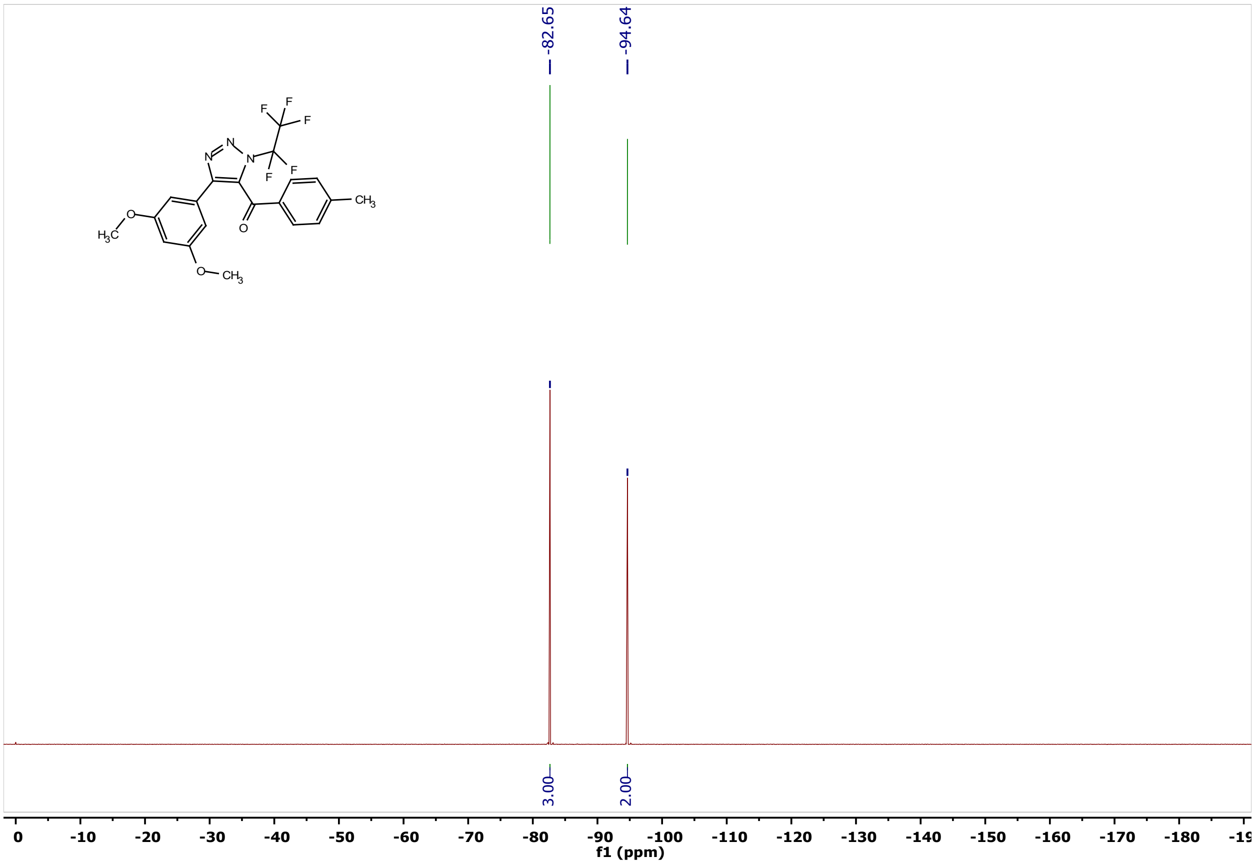

Figure S49. ^{1}H NMR spectrum of **1p** (401 MHz; $CDCl_3$)

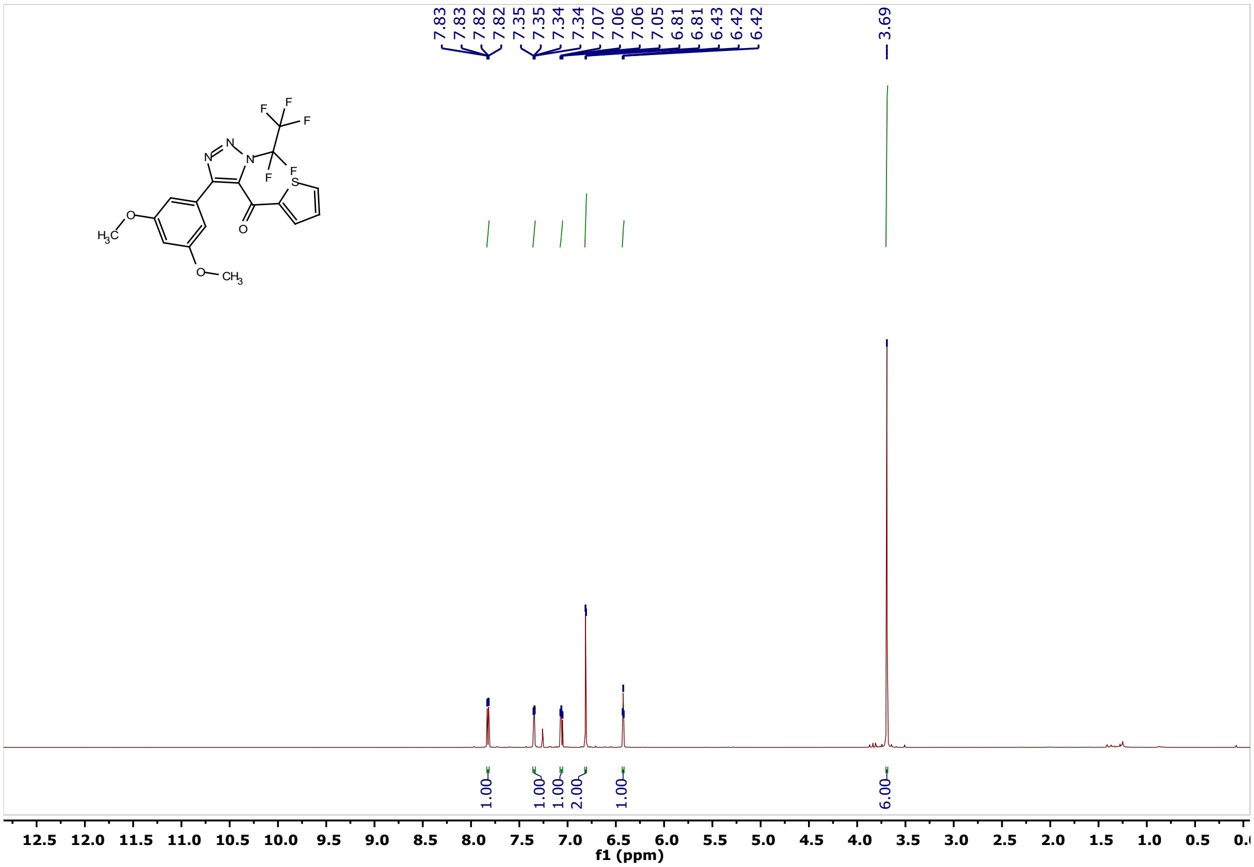

Figure S50. ^{13}C NMR spectrum of **1p** (101 MHz; $CDCl_3$)

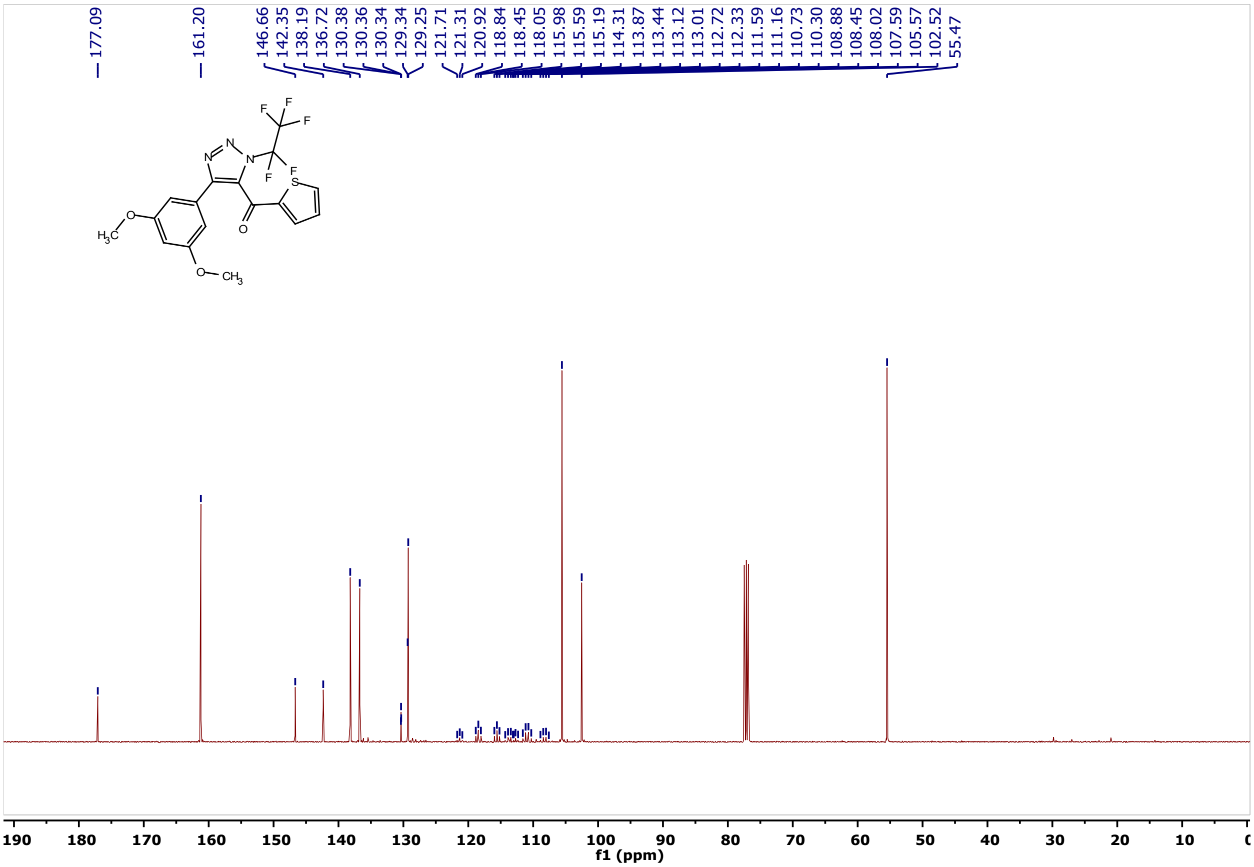

Figure S51. ^{19}F NMR spectrum of **1p** (377 MHz; $CDCl_3$)

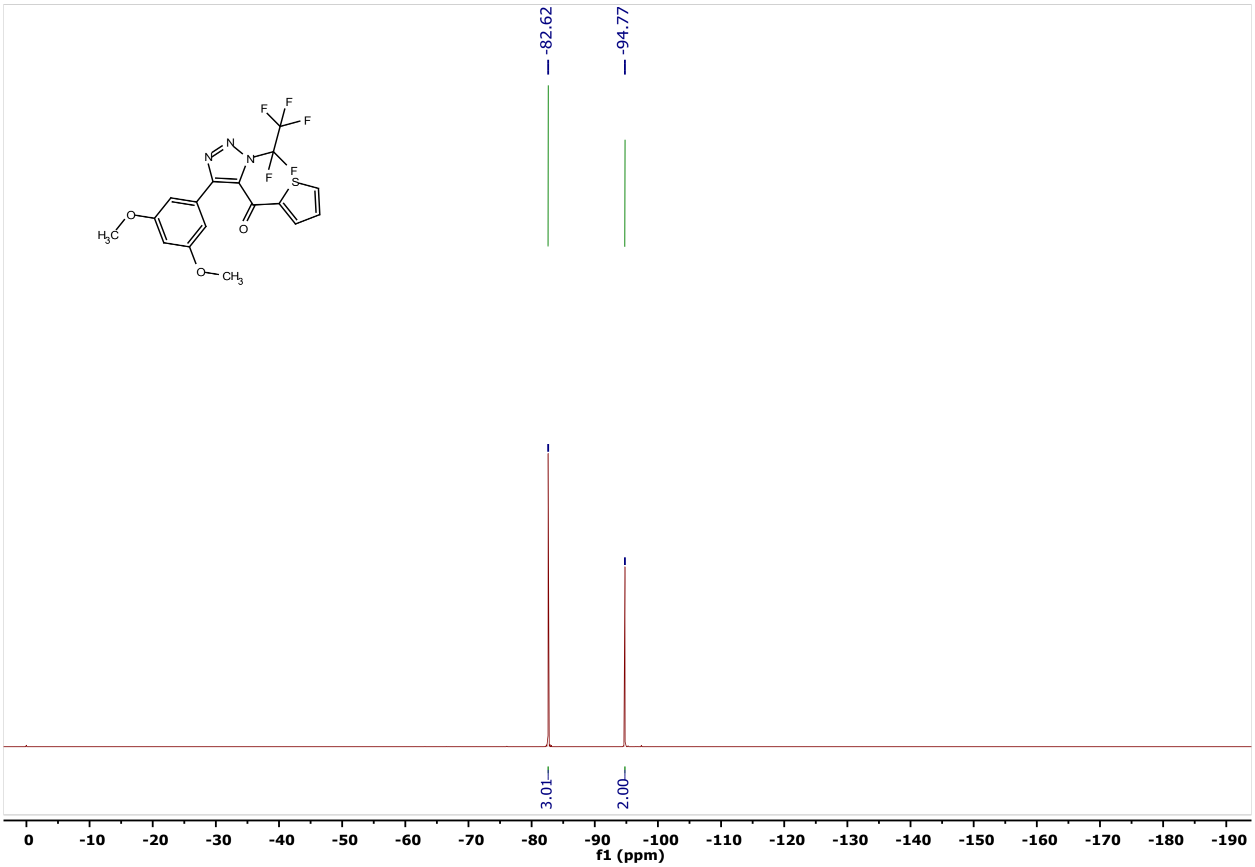

Figure S52. ^{1}H NMR spectrum of **1q** (401 MHz; $CDCl_3$)

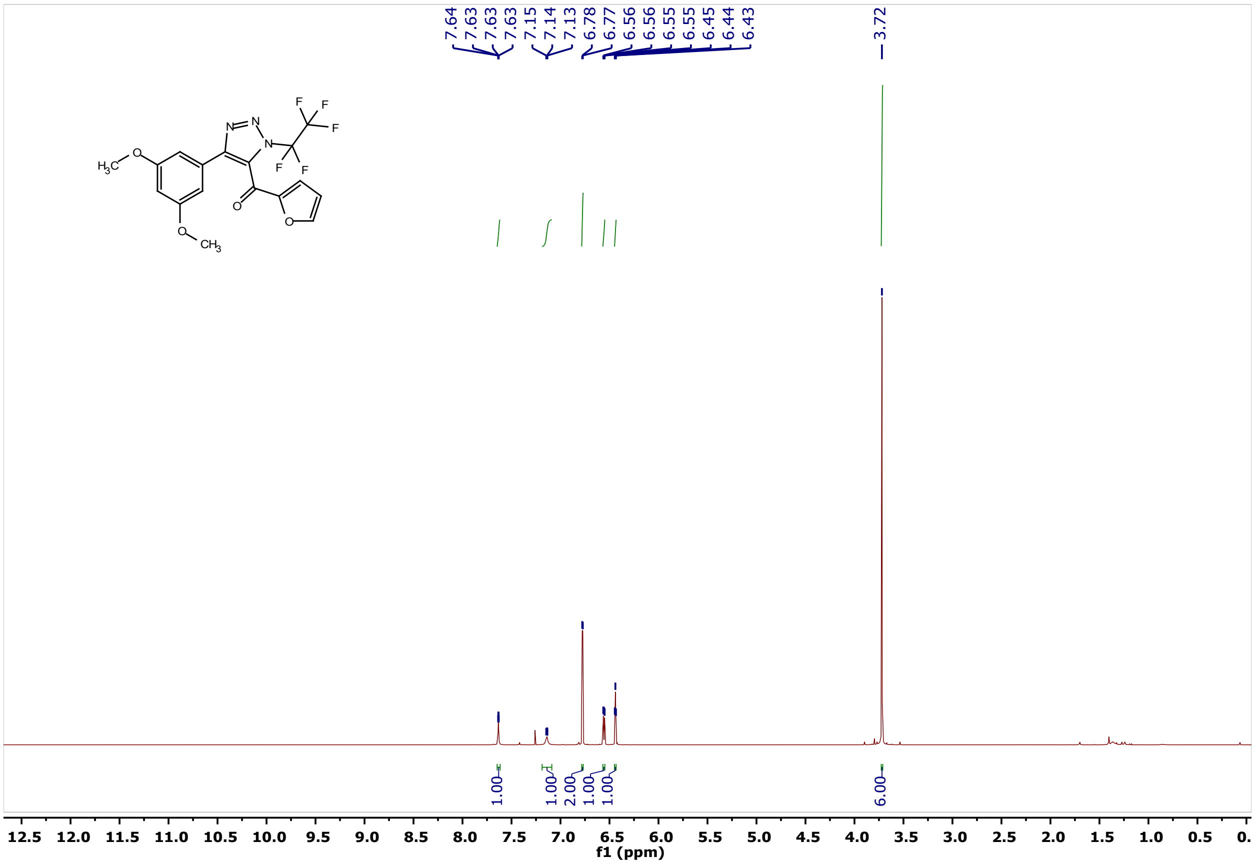

Figure S53. ^{13}C NMR spectrum of **1q** (101 MHz; $CDCl_3$)

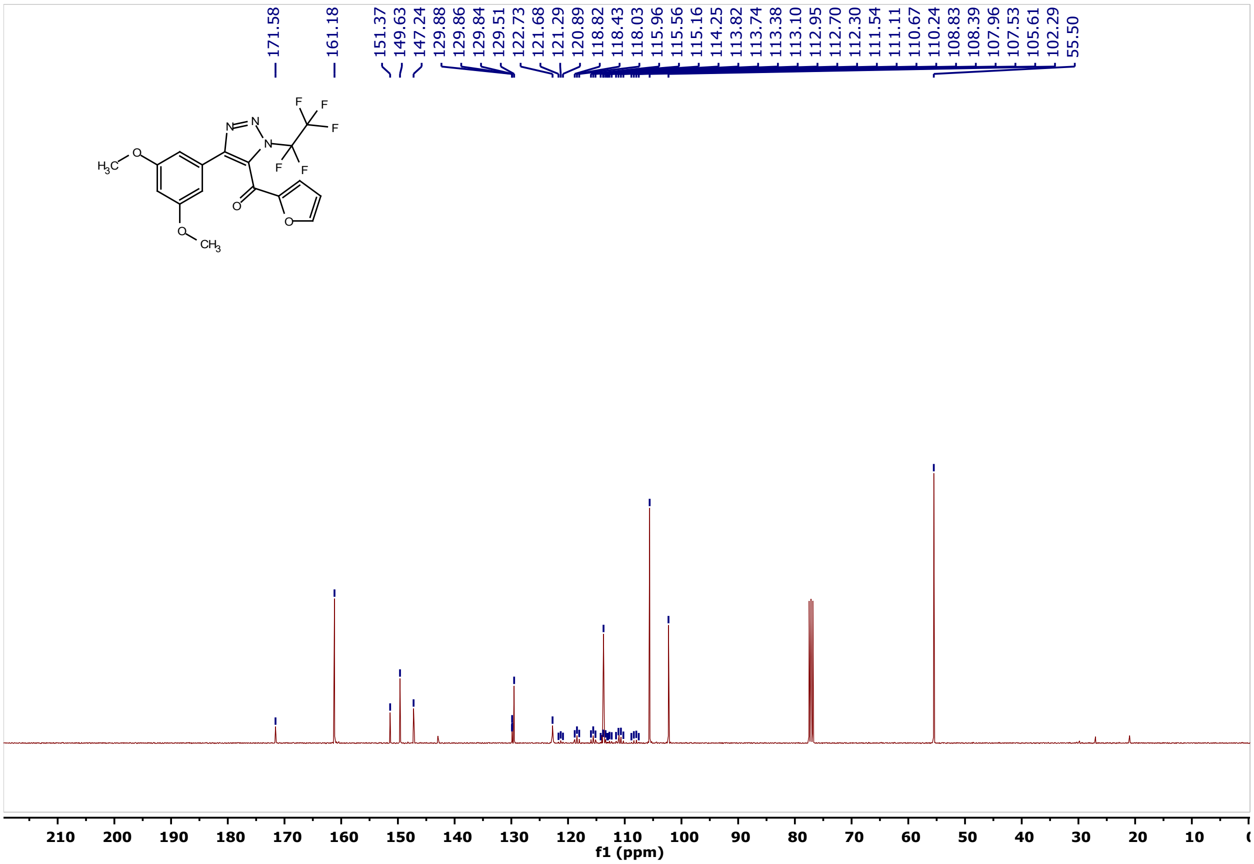

Figure S54. ^{19}F NMR spectrum of **1q** (377 MHz; $CDCl_3$)

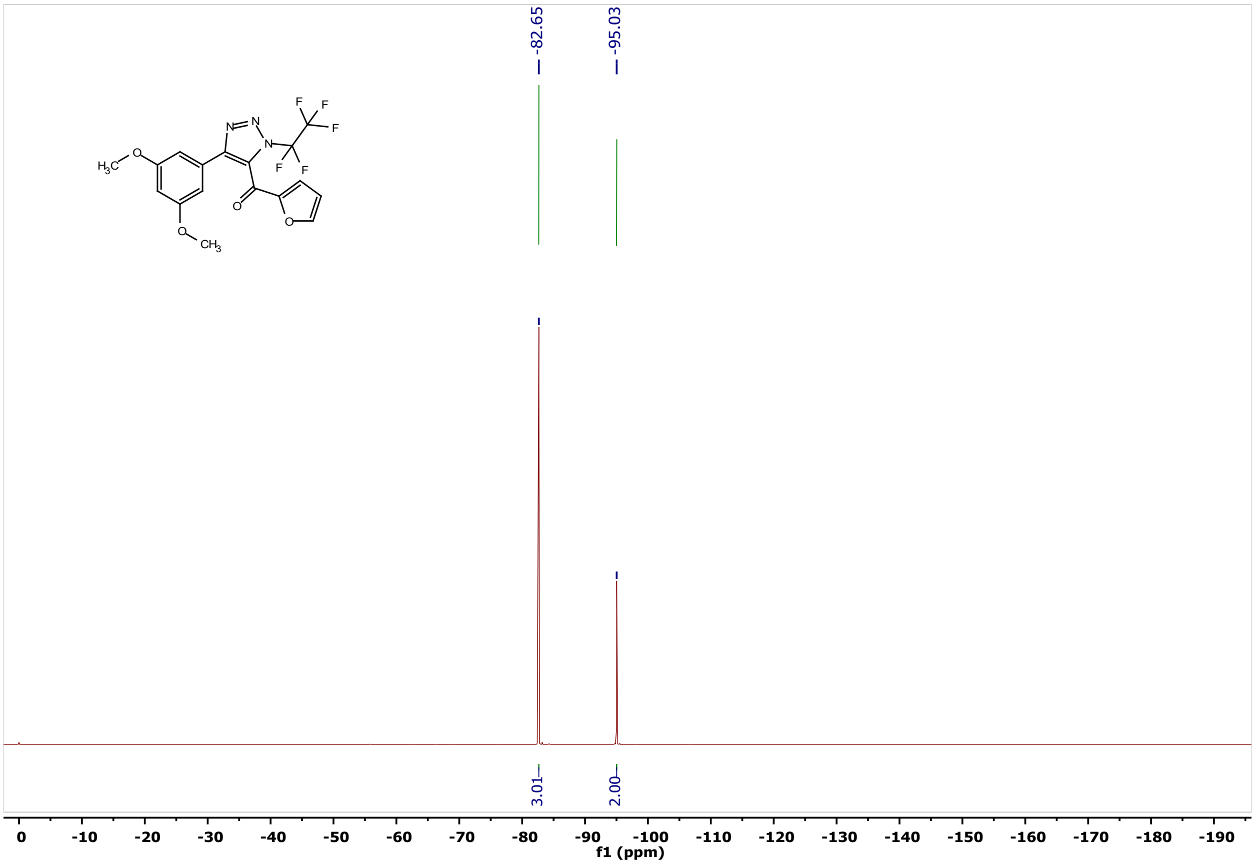

Figure S55. ^{1}H NMR spectrum of **1r** (401 MHz; $CDCl_3$)

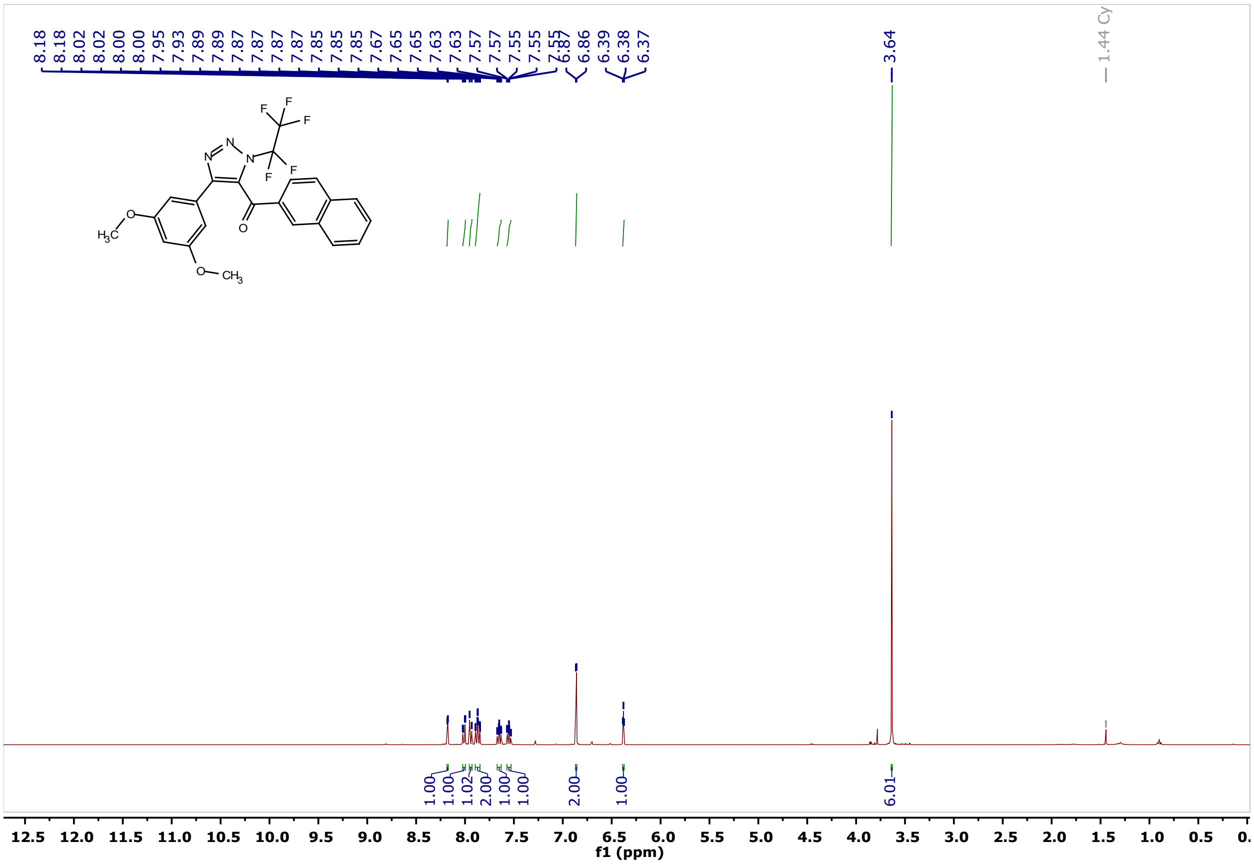

Figure S56. ^{13}C NMR spectrum of **1r** (101 MHz; $CDCl_3$)

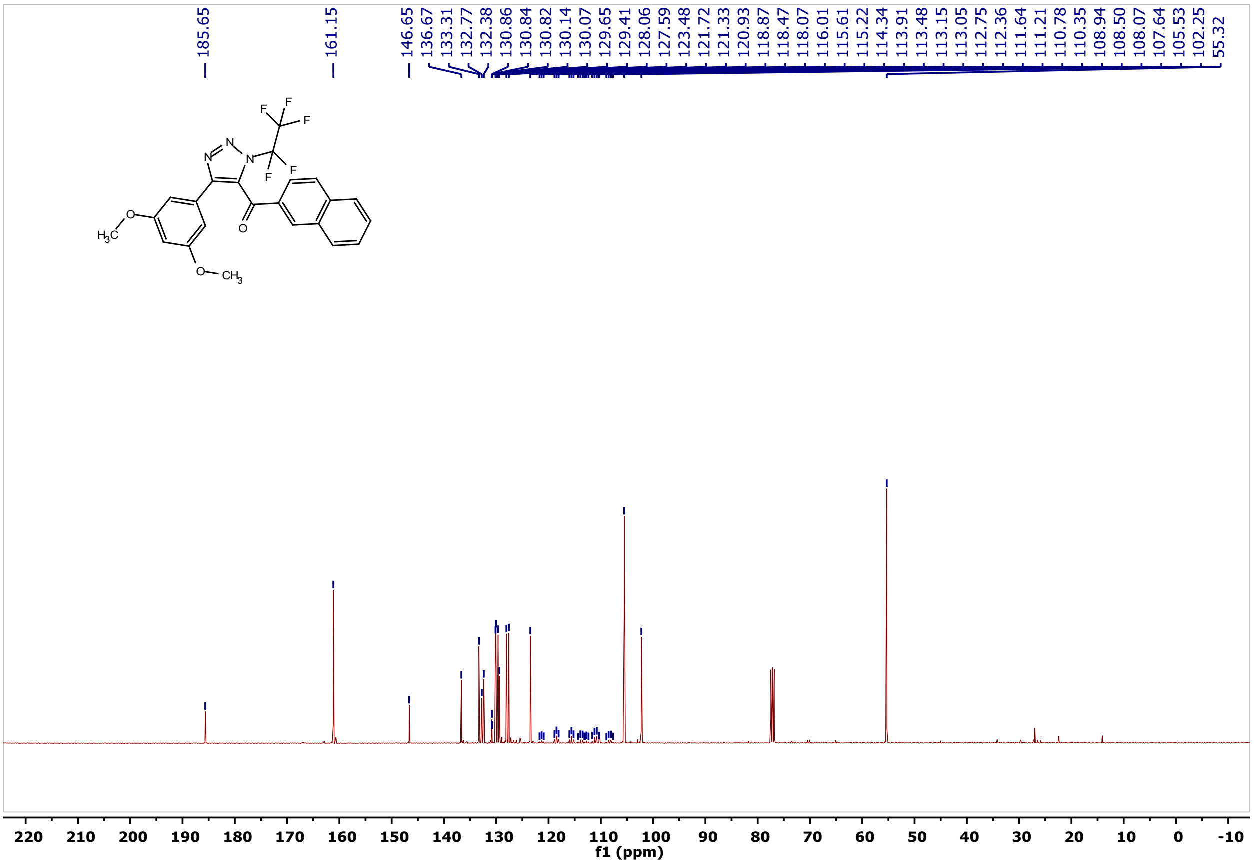

Figure S57. ^{19}F NMR spectrum of **1r** (377 MHz; $CDCl_3$)

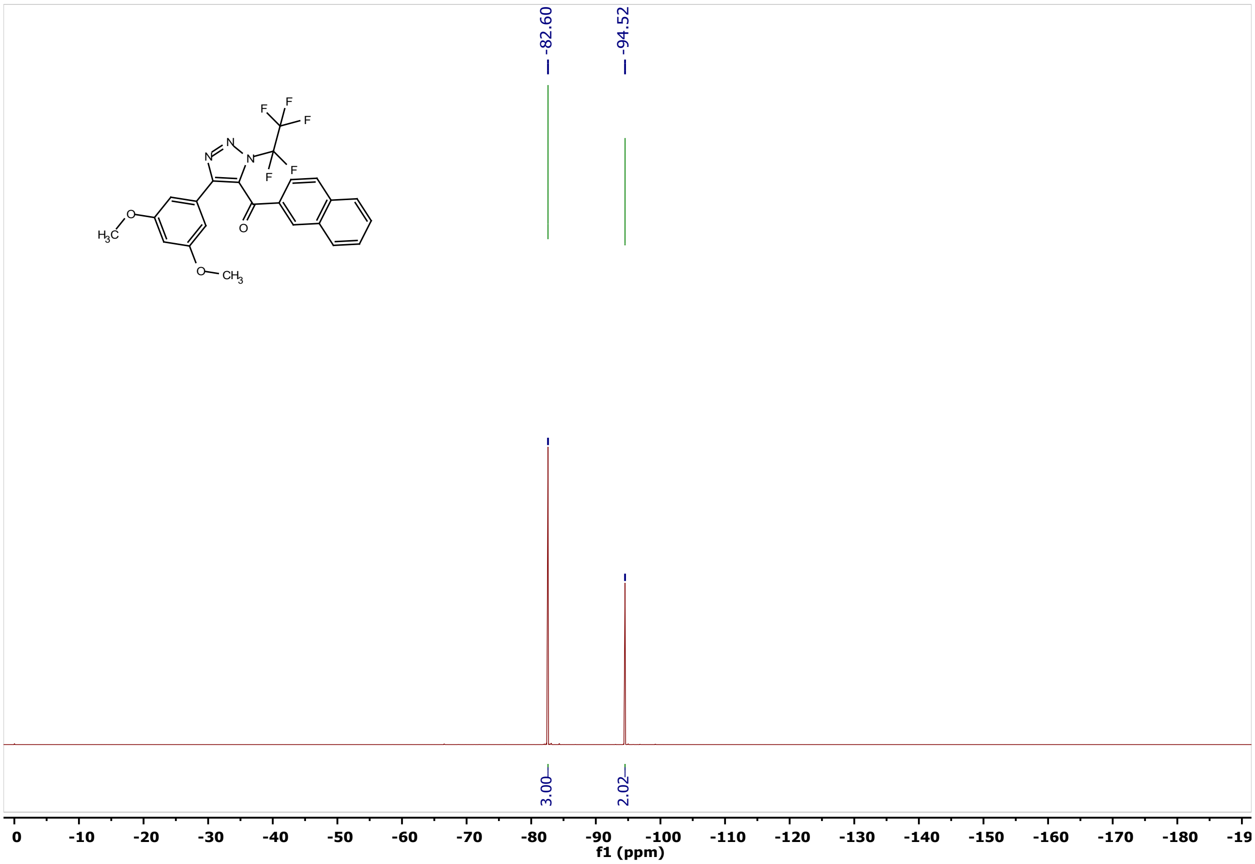

Figure S58. ^{1}H NMR spectrum of **1s** (401 MHz; $CDCl_3$)

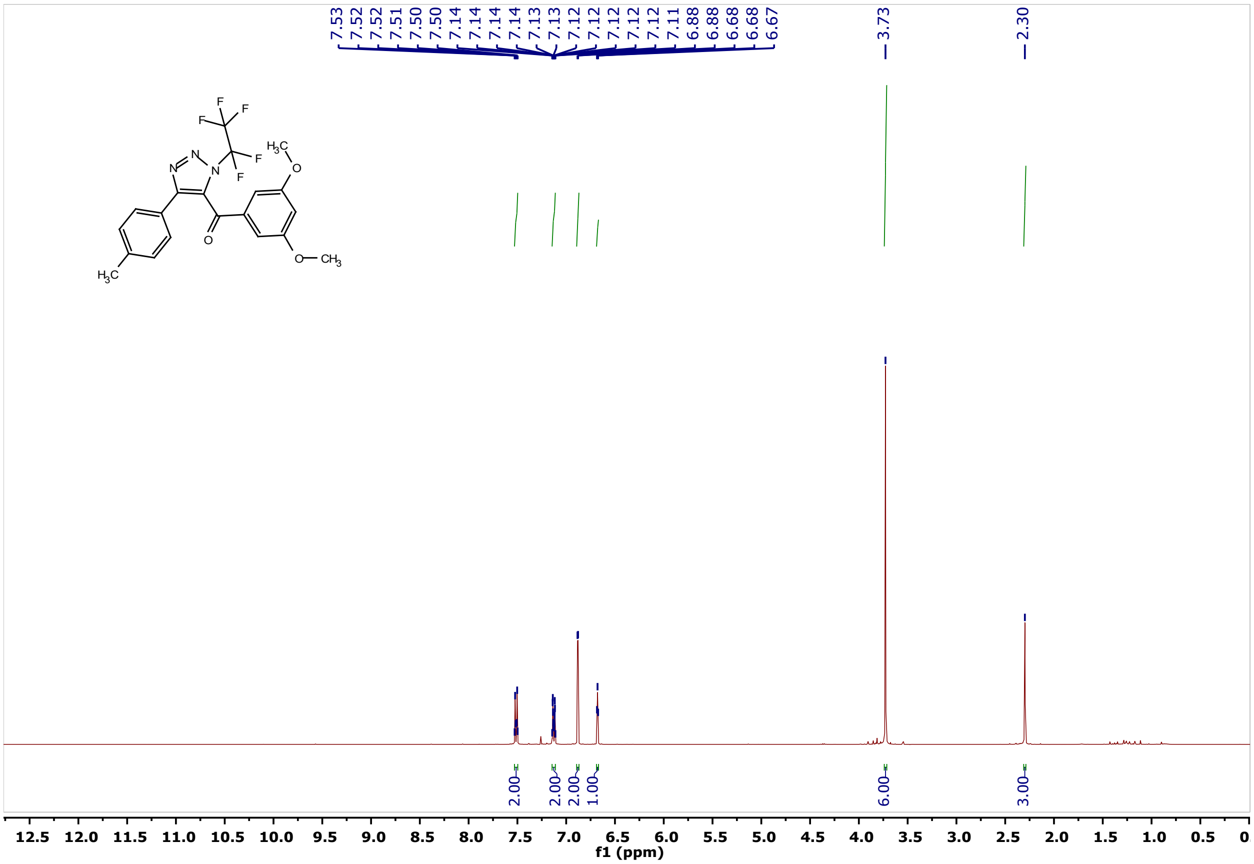

Figure S59. ^{13}C NMR spectrum of **1s** (101 MHz; $CDCl_3$)

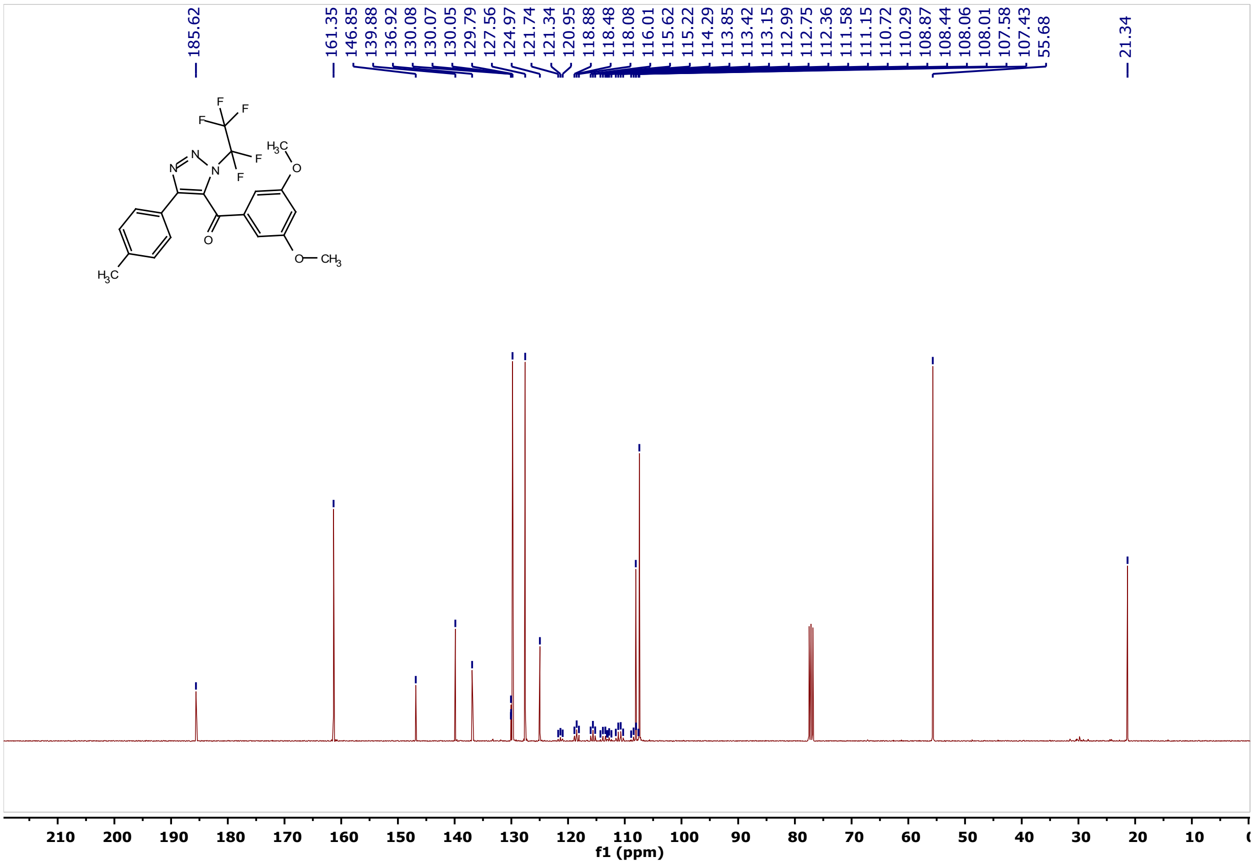

Figure S60. ^{19}F NMR spectrum of **1s** (377 MHz; $CDCl_3$)

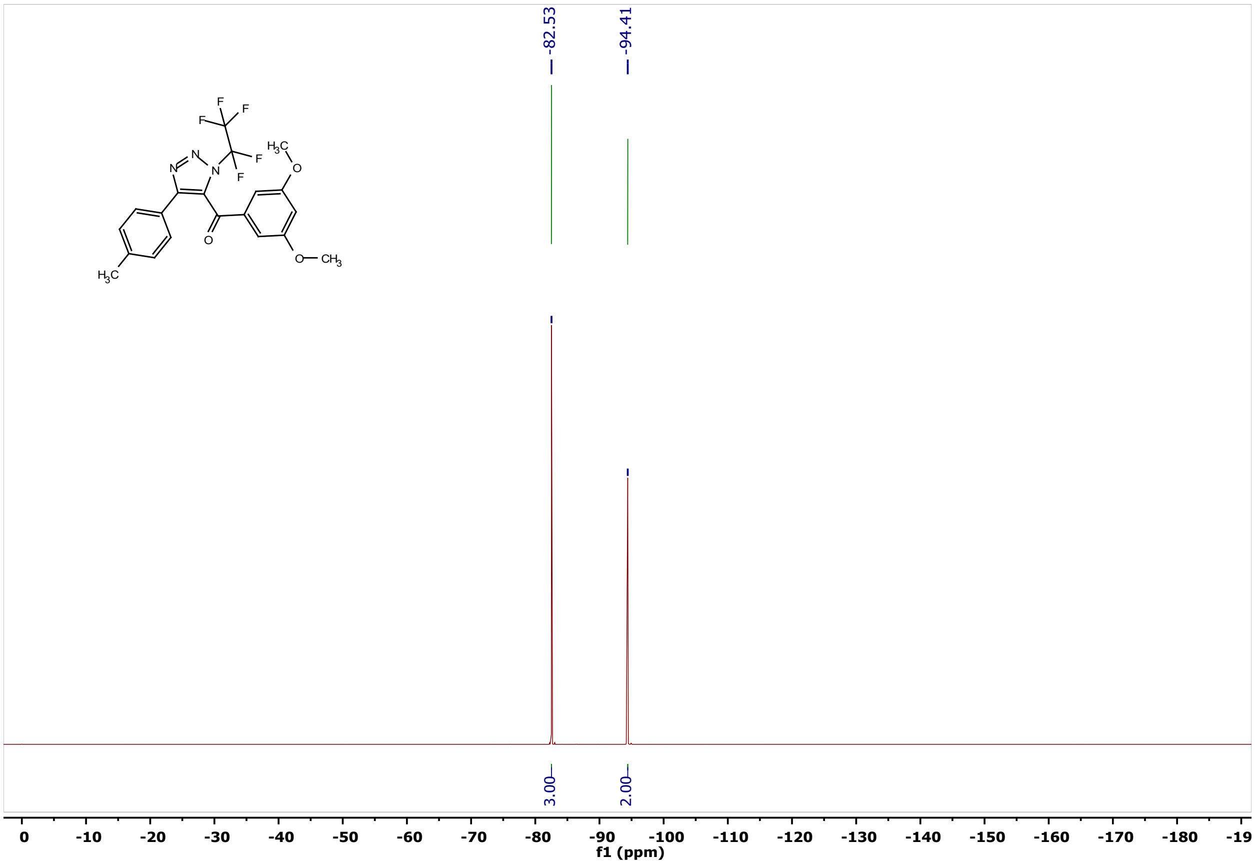

Figure S61. ^{1}H NMR spectrum of **1t** (401 MHz; $CDCl_3$)

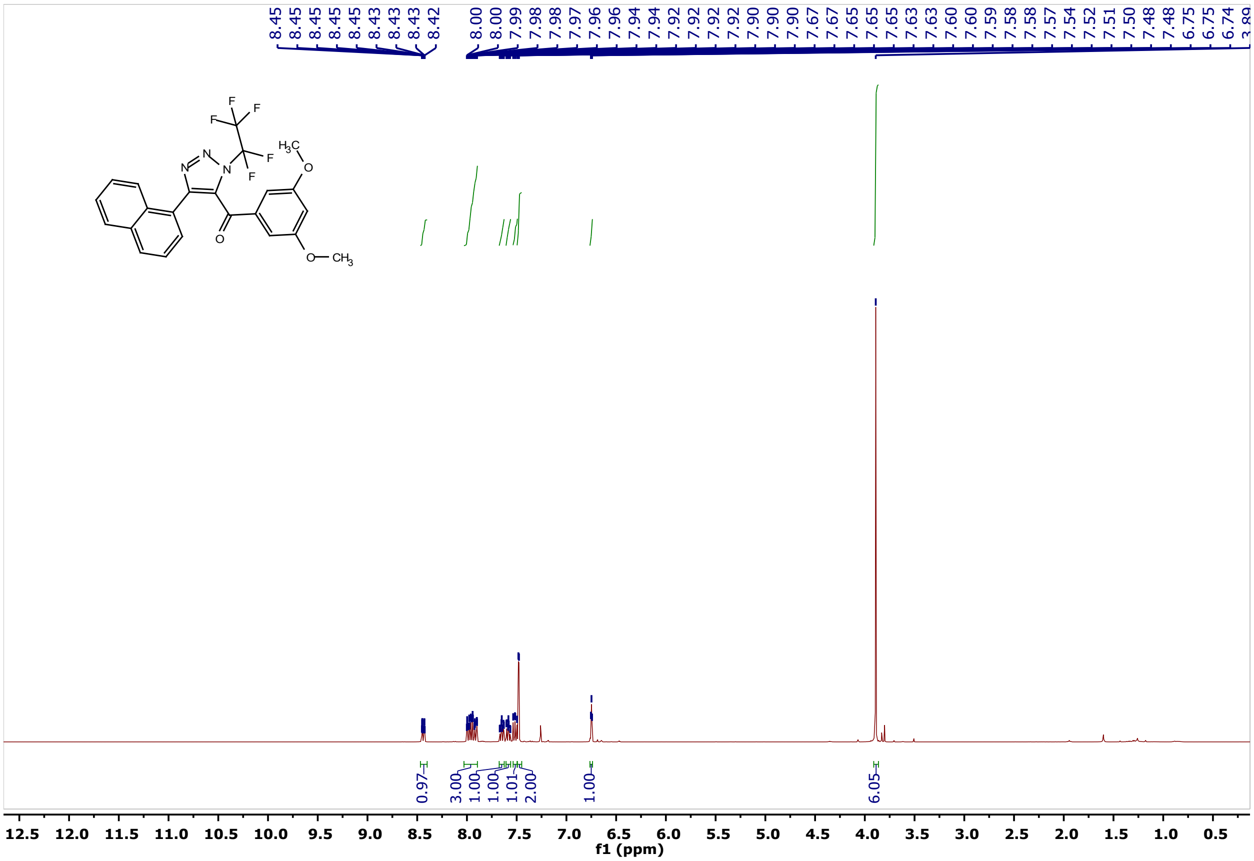

Figure S62. ^{13}C NMR spectrum of **1t** (101 MHz; $CDCl_3$)

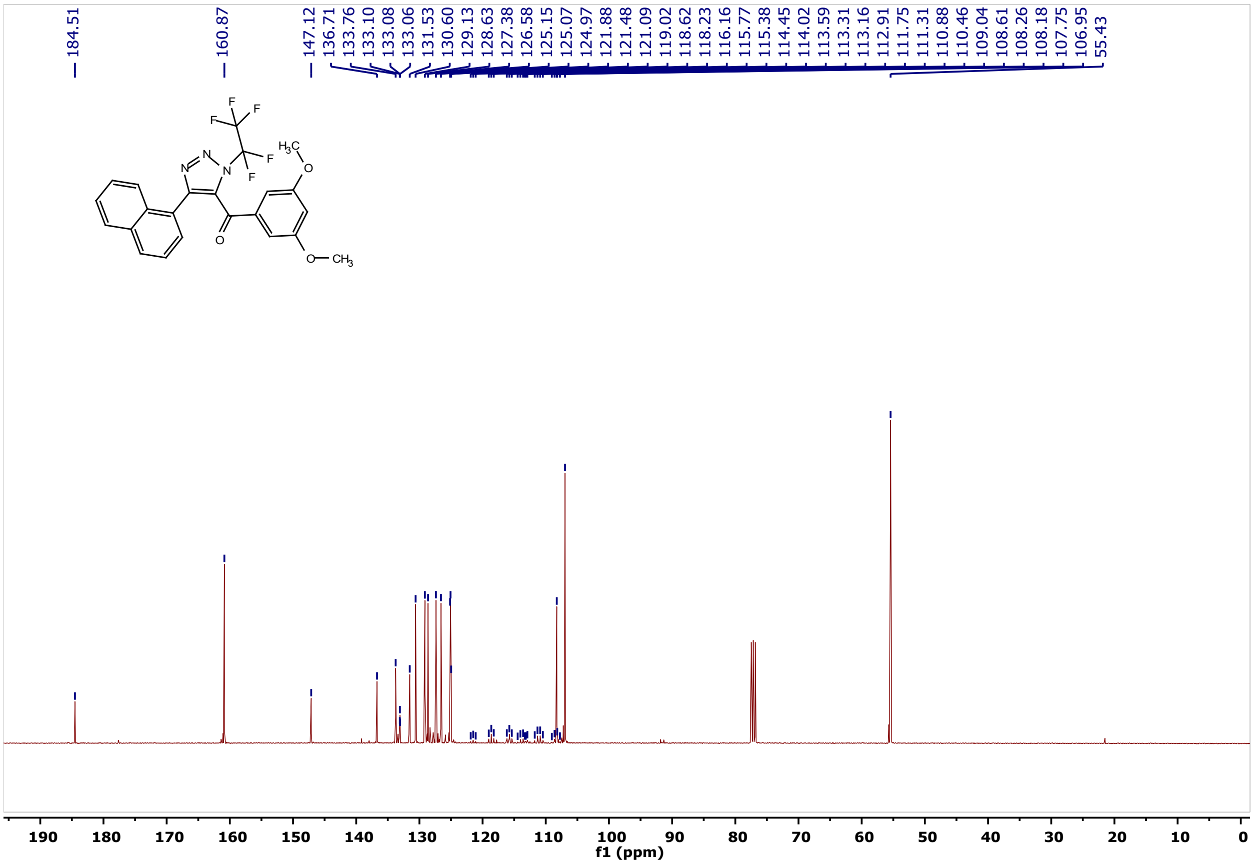

Figure S63. ^{19}F NMR spectrum of **1t** (377 MHz; $CDCl_3$)

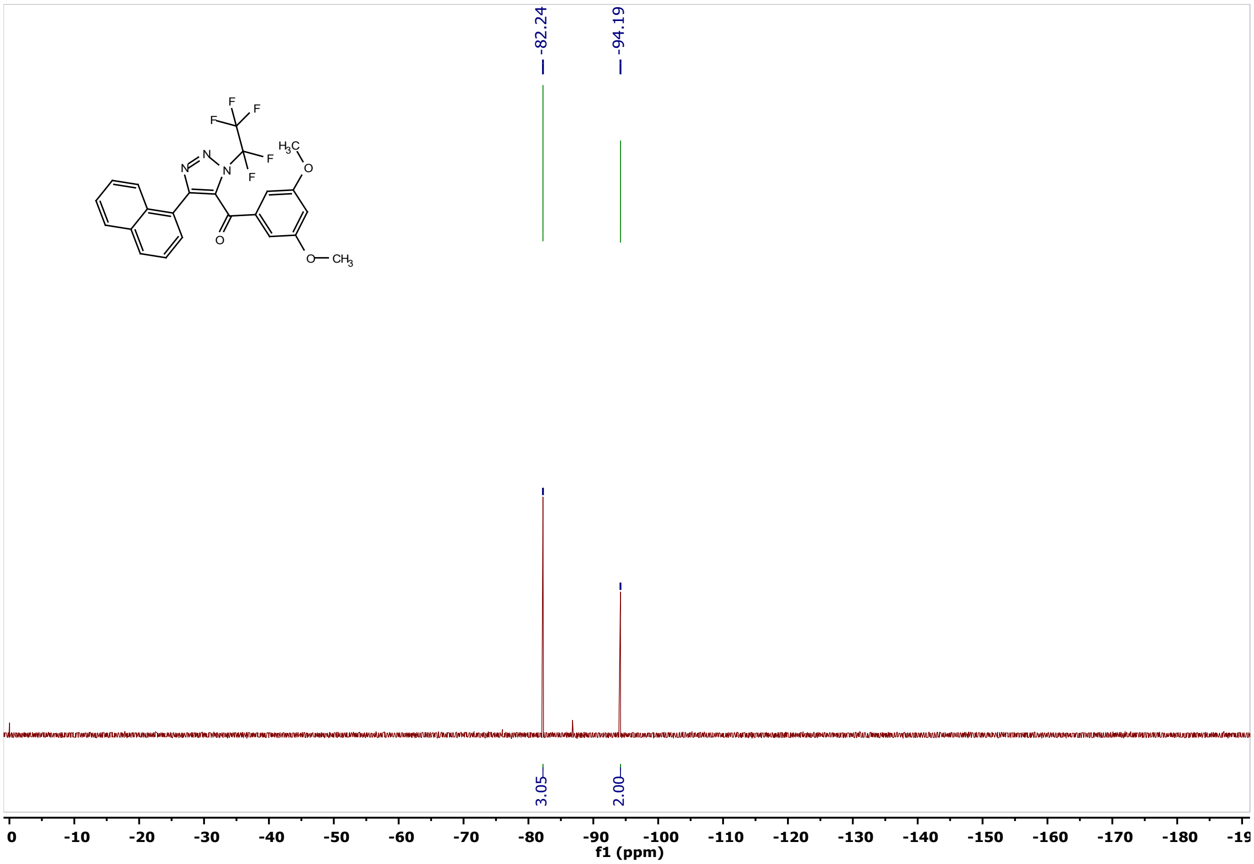

Figure S64. ^{1}H NMR spectrum of **2a** (401 MHz; $CDCl_3$)

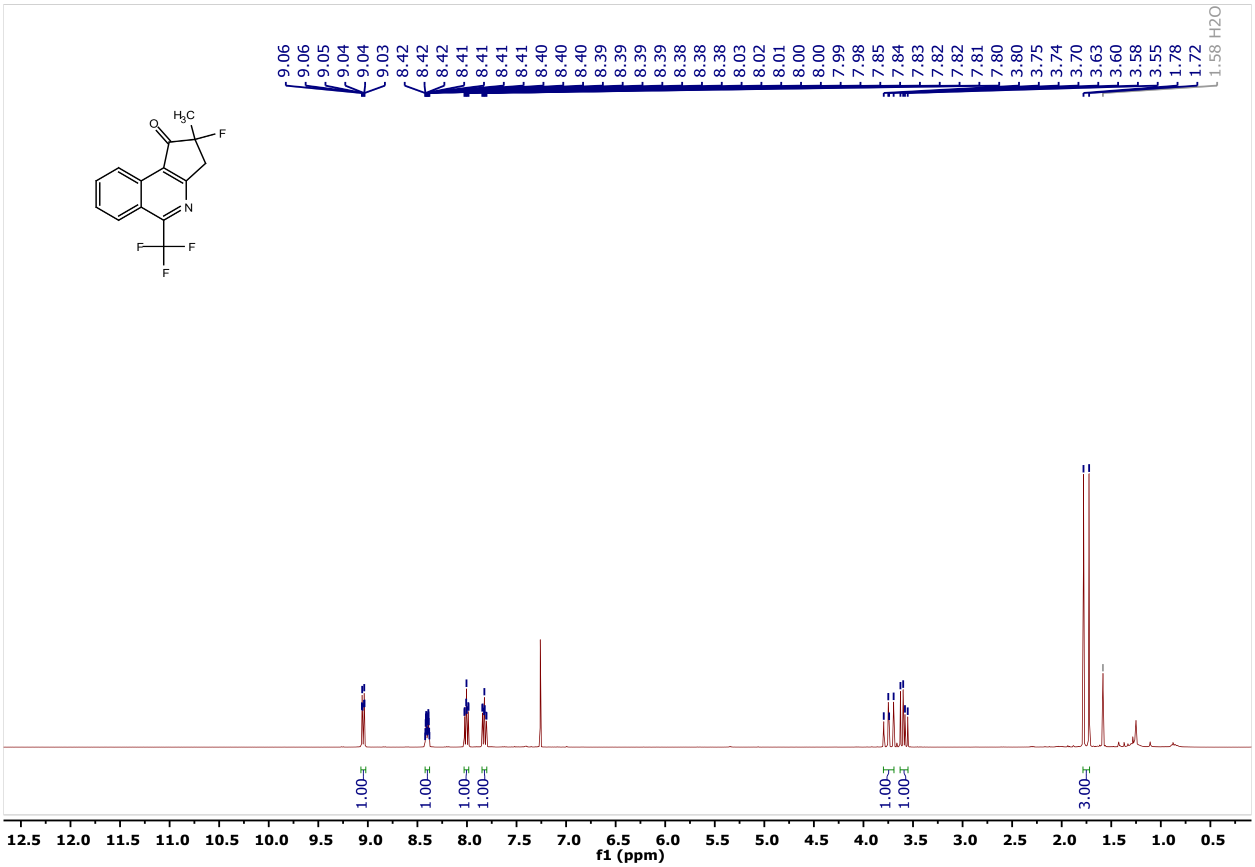

Figure S65. ^{13}C NMR spectrum of **2a** (101 MHz; $CDCl_3$)

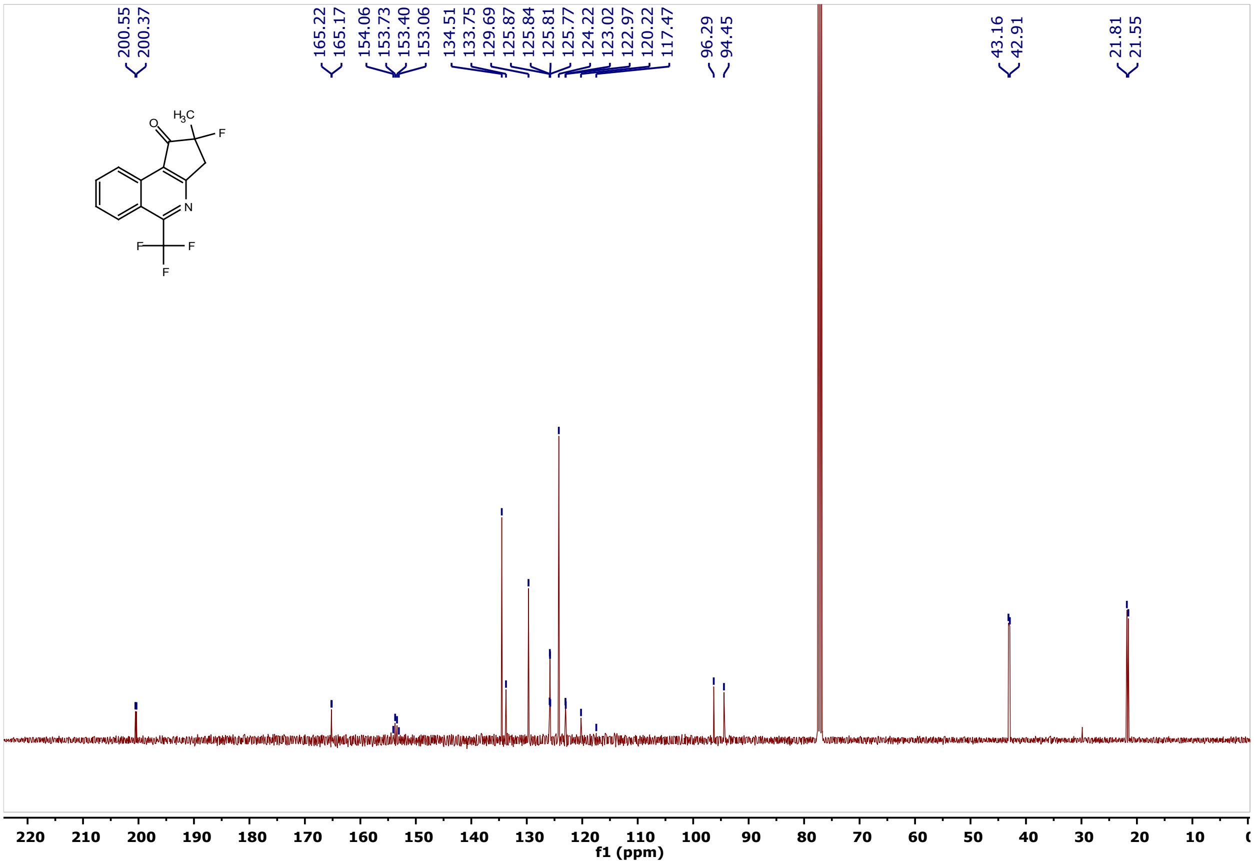

Figure S66. ^{19}F NMR spectrum of **2a** (377 MHz; $CDCl_3$)

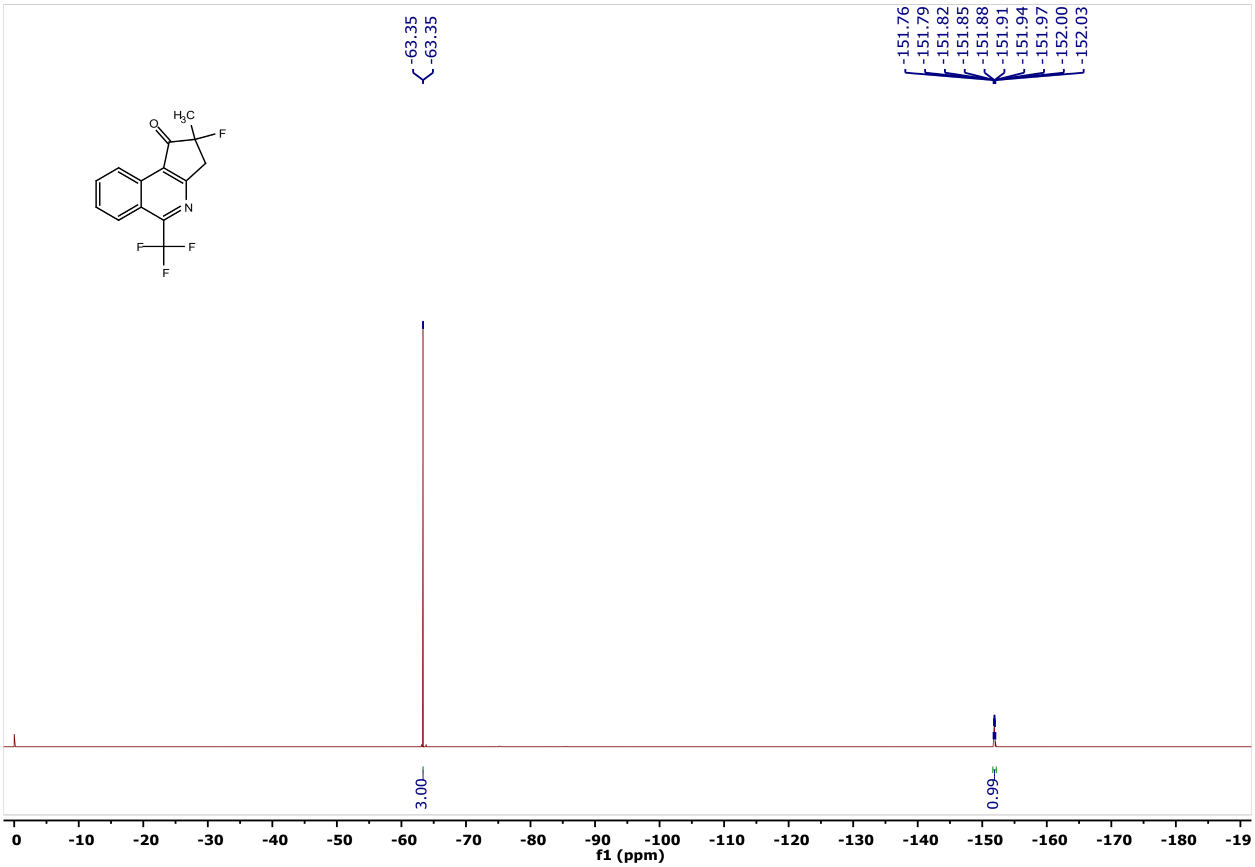

Figure S67. ^{1}H NMR spectrum of **2b** (401 MHz; $CDCl_3$)

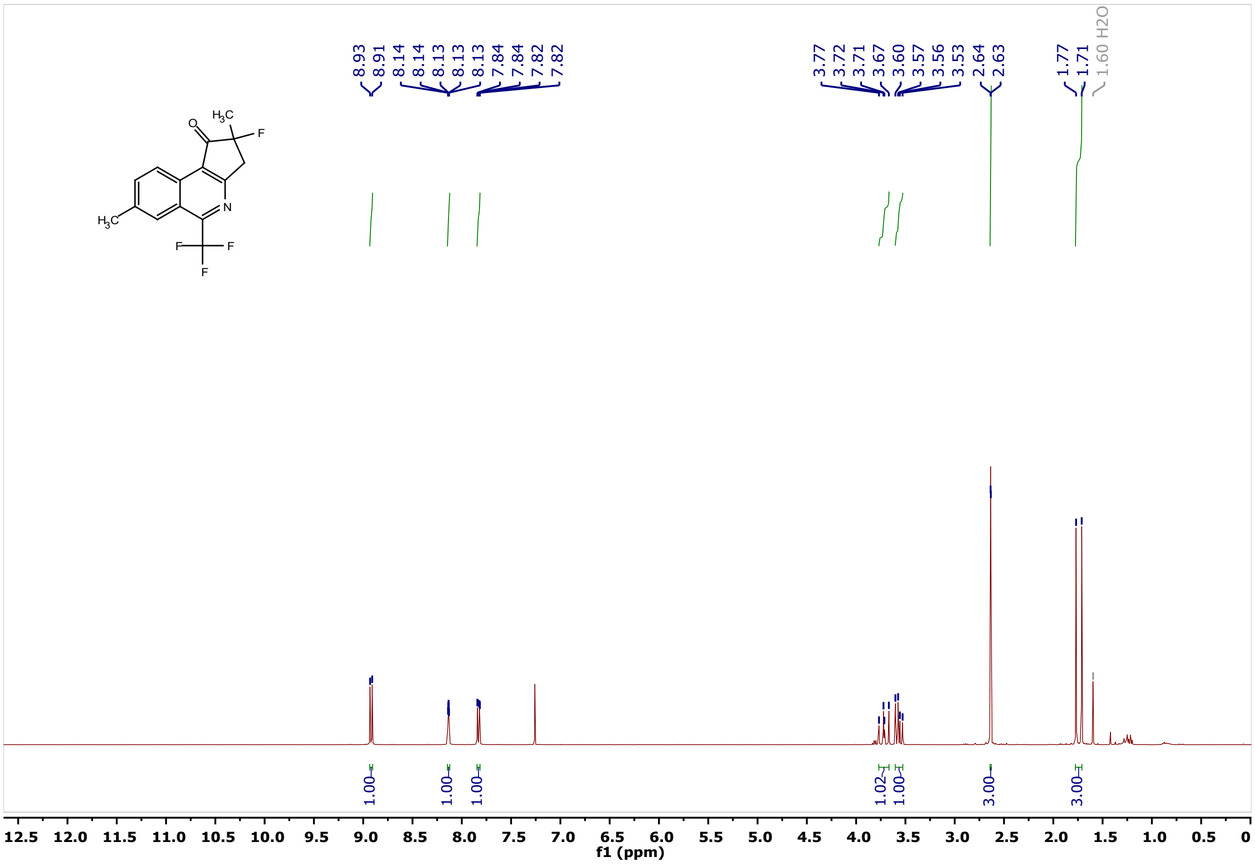

Figure S68. ^{13}C NMR spectrum of **2b** (101 MHz; $CDCl_3$)

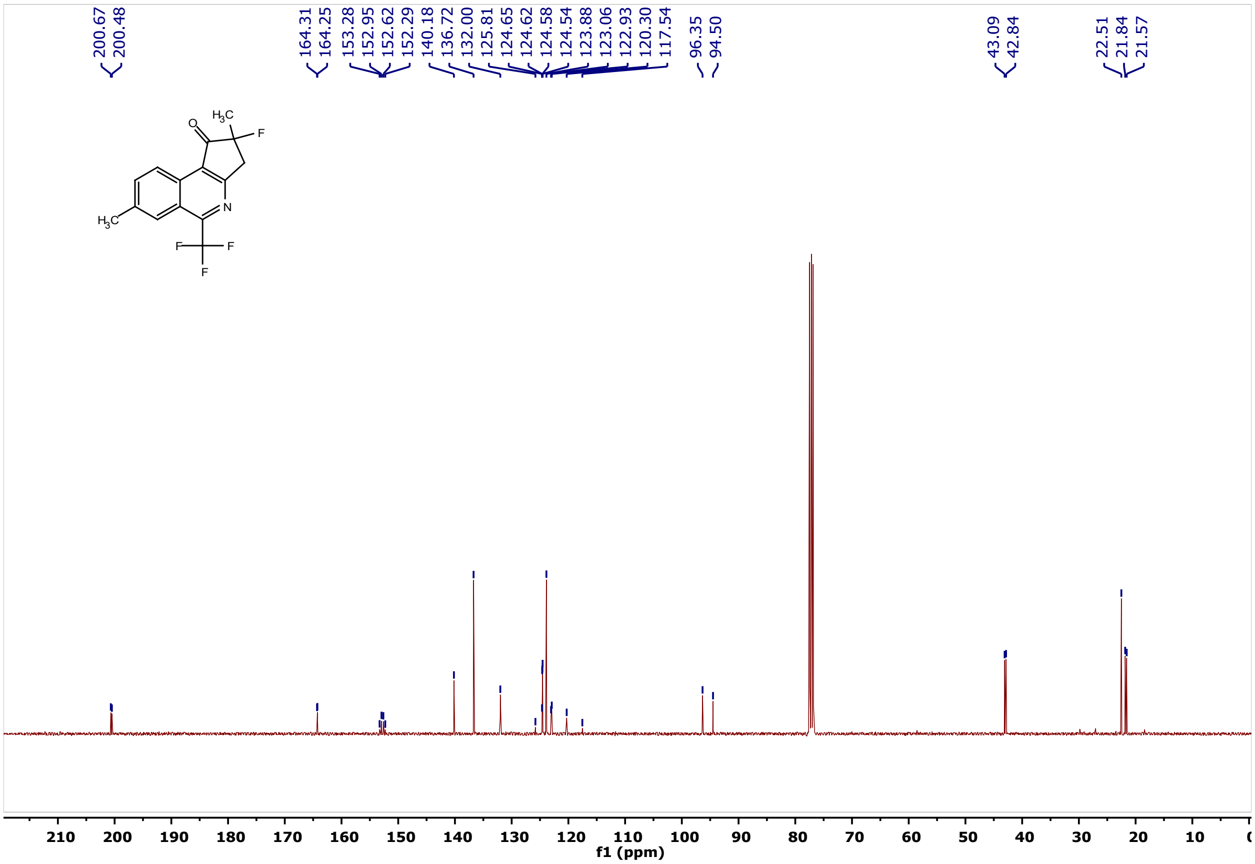

Figure S69. ^{19}F NMR spectrum of **2b** (377 MHz; $CDCl_3$)

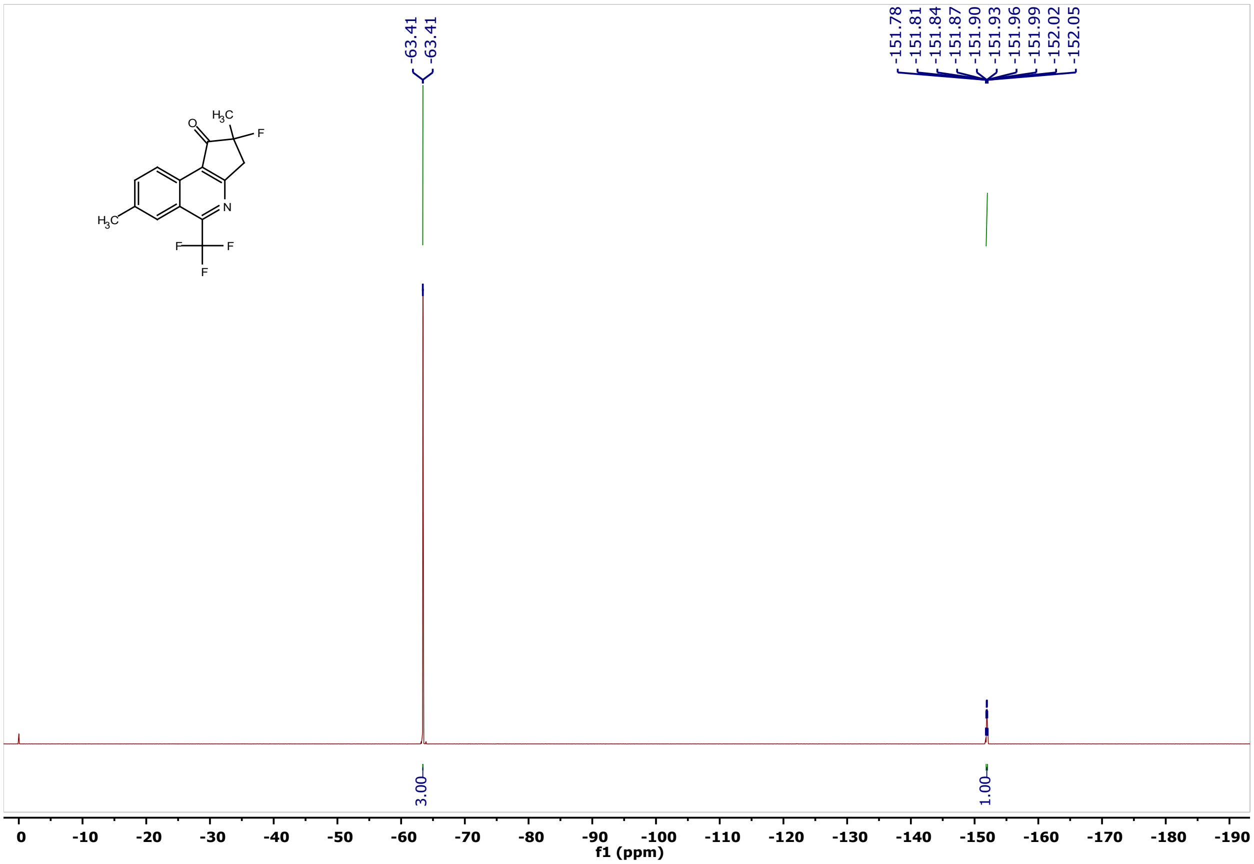

Figure S70. ^{1}H NMR spectrum of **2c** (401 MHz; $CDCl_3$)

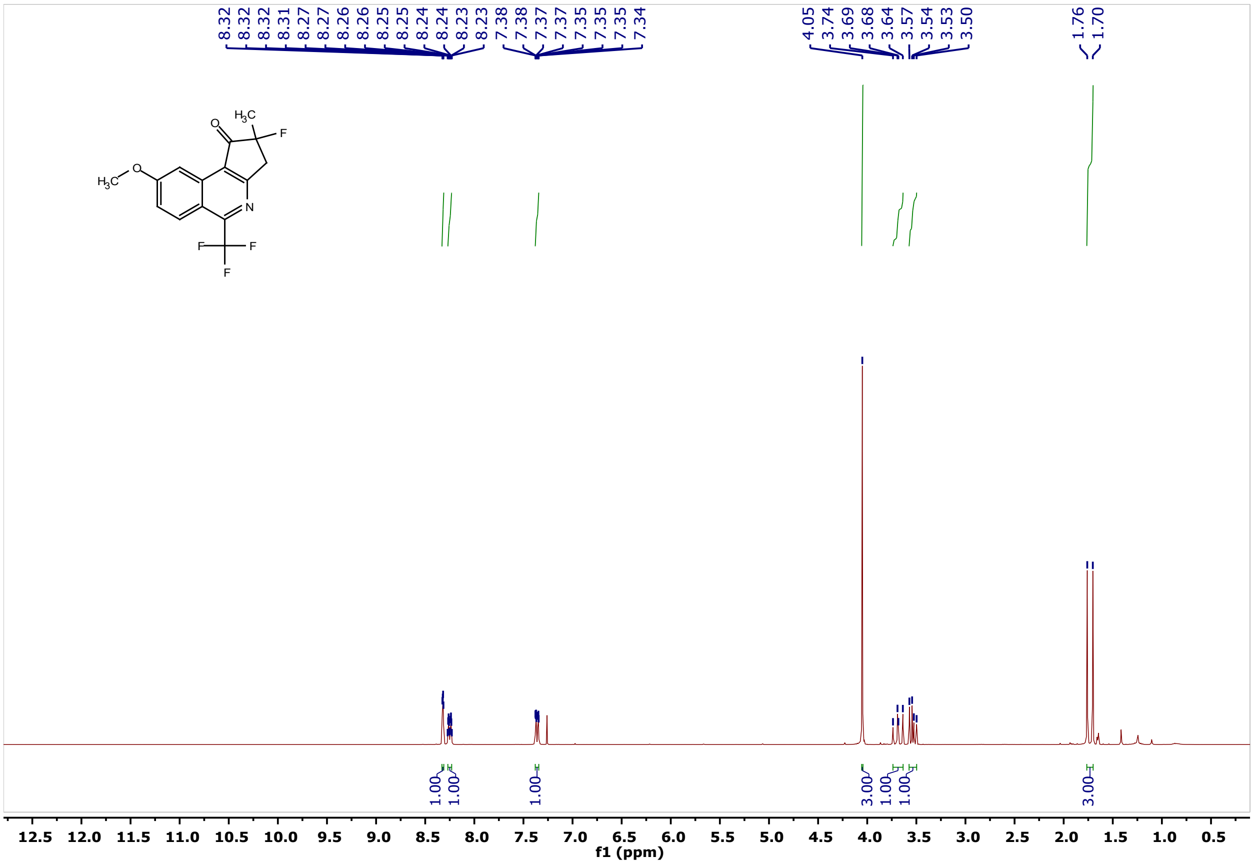

Figure S71. ^{13}C NMR spectrum of **2c** (101 MHz; $CDCl_3$)

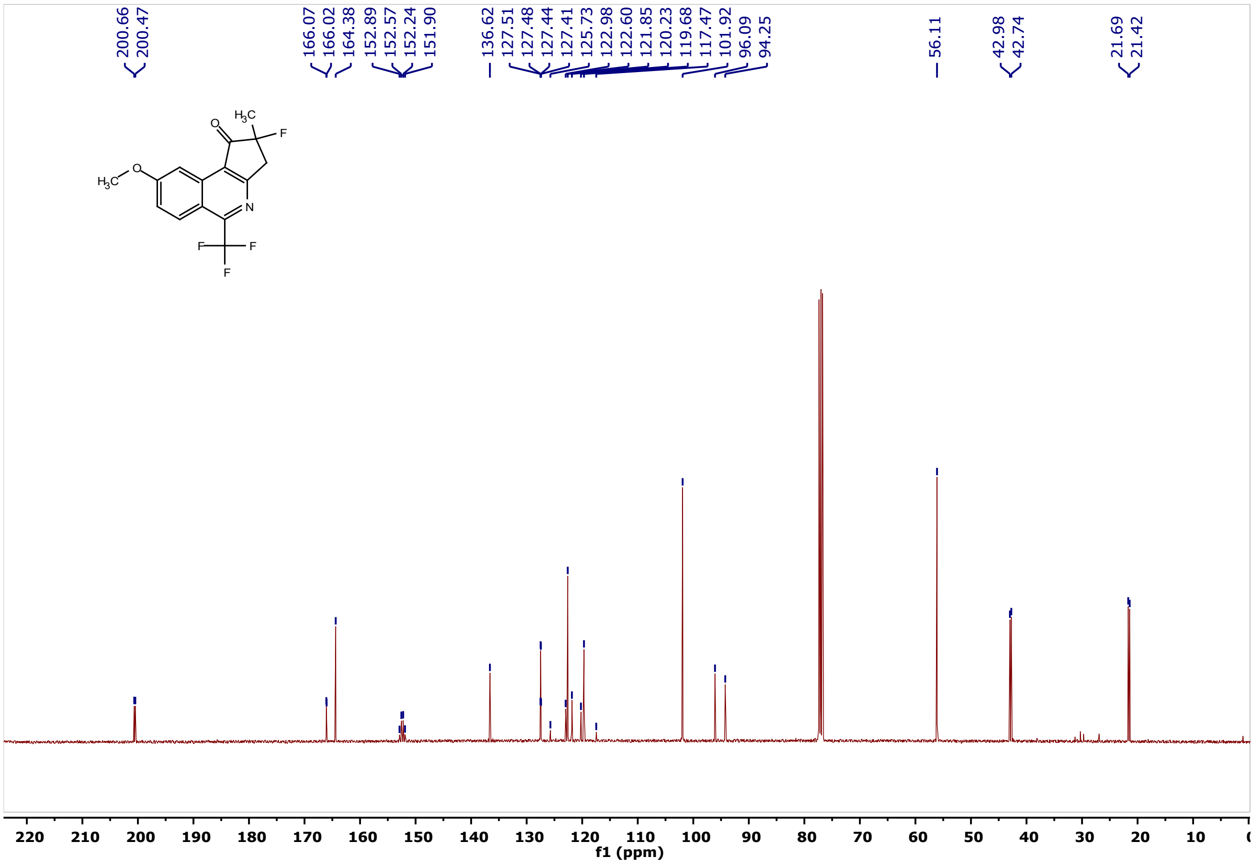

Figure S72. ^{19}F NMR spectrum of **2c** (377 MHz; $CDCl_3$)

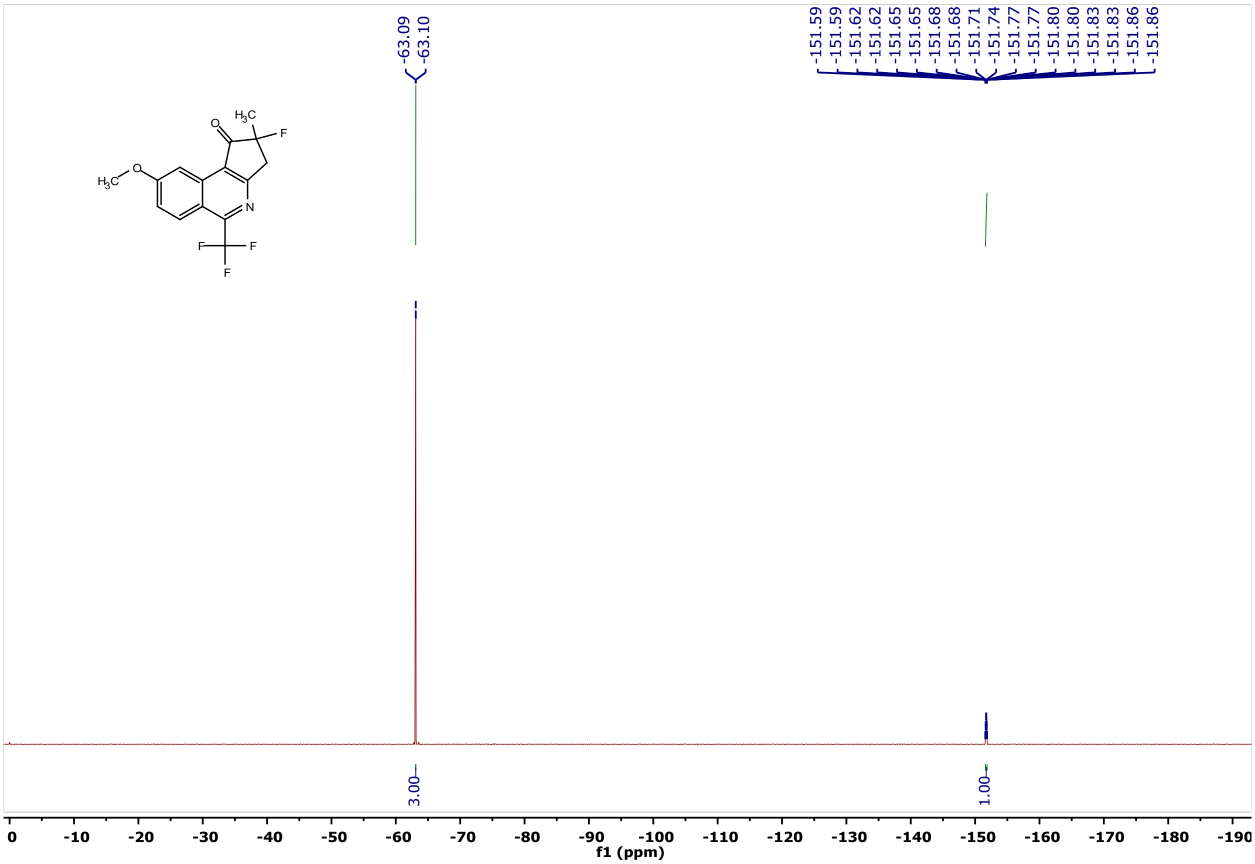

Figure S73. ^{1}H NMR spectrum of **2d** (401 MHz; $CDCl_3$)

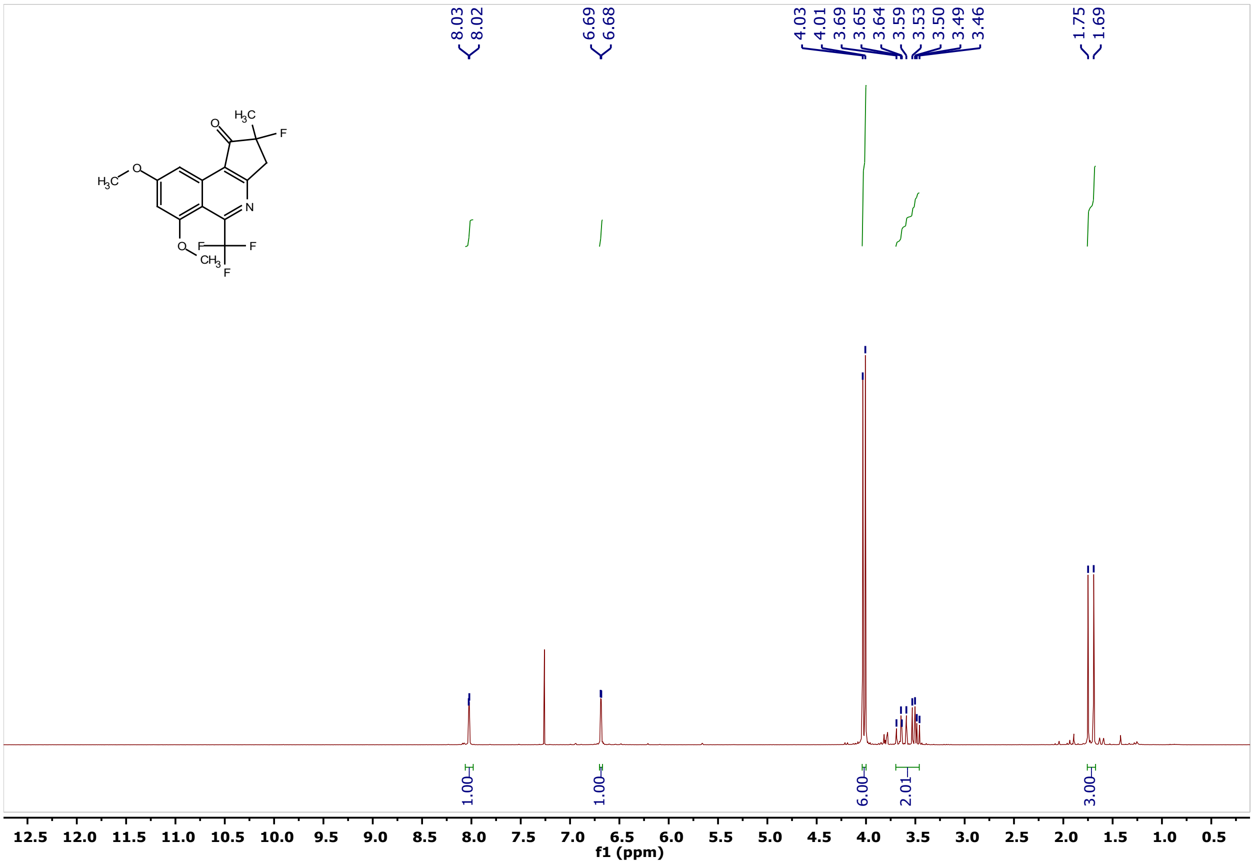

Figure S74. ^{13}C NMR spectrum of **2d** (101 MHz; $CDCl_3$)

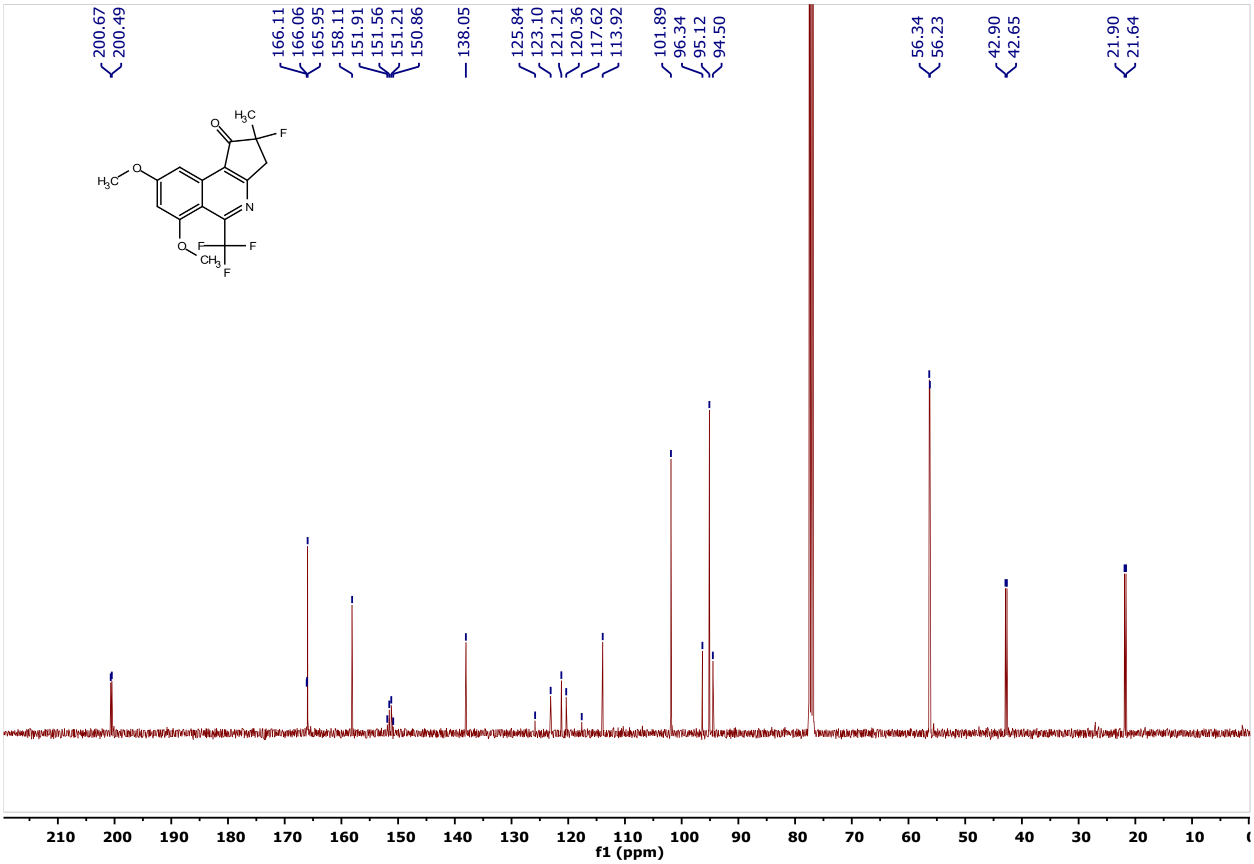

Figure S75. ^{19}F NMR spectrum of **2d** (377 MHz; $CDCl_3$)

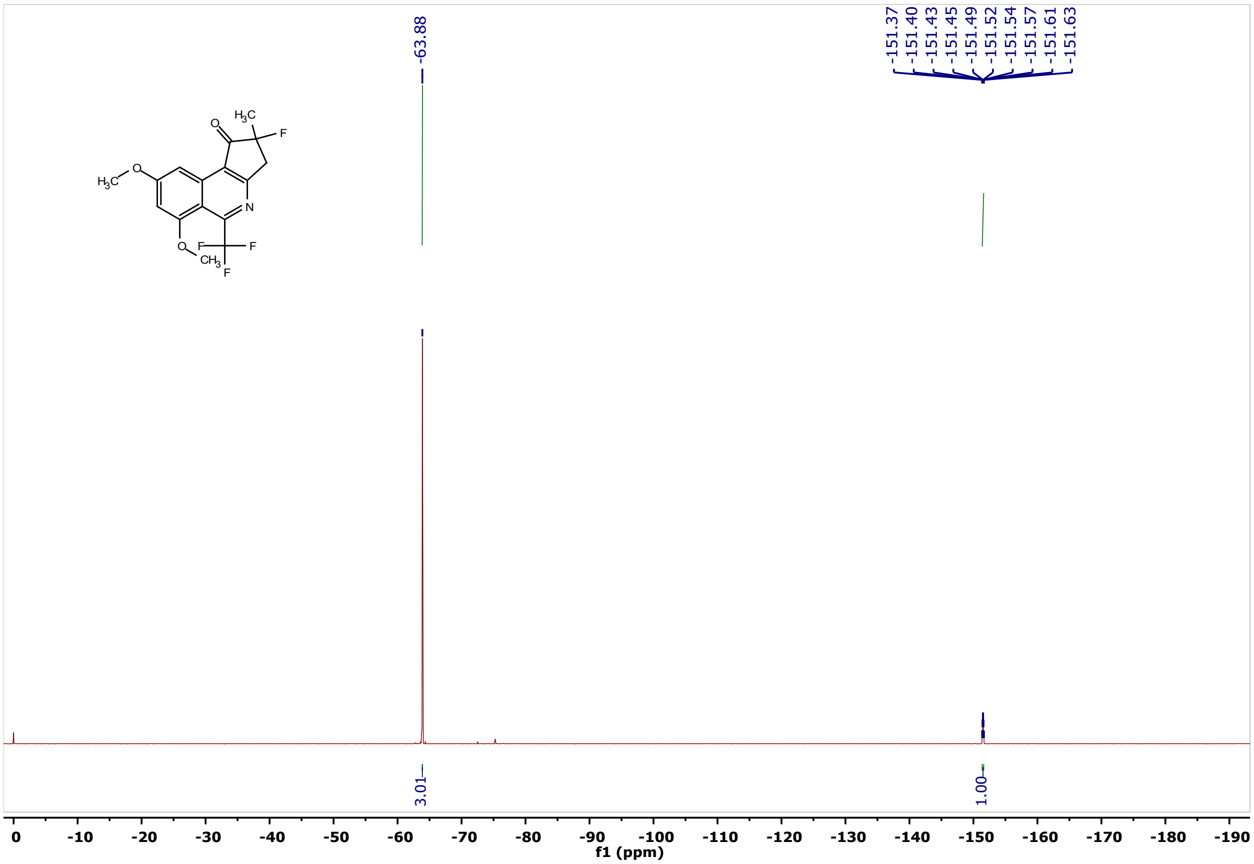

Figure S76. ^{1}H NMR spectrum of **3a** (401 MHz; $CDCl_3$)

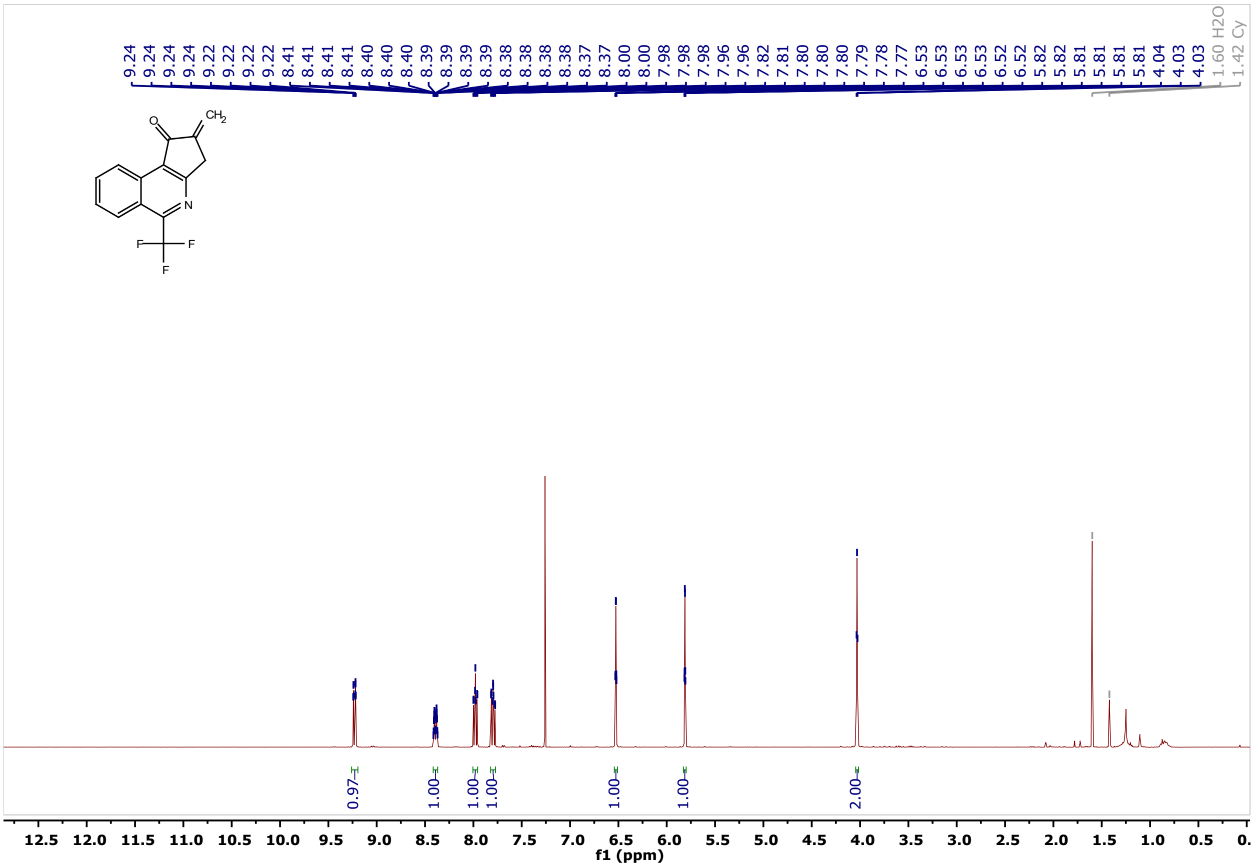

Figure S77. ^{13}C NMR spectrum of **3a** (101 MHz; $CDCl_3$)

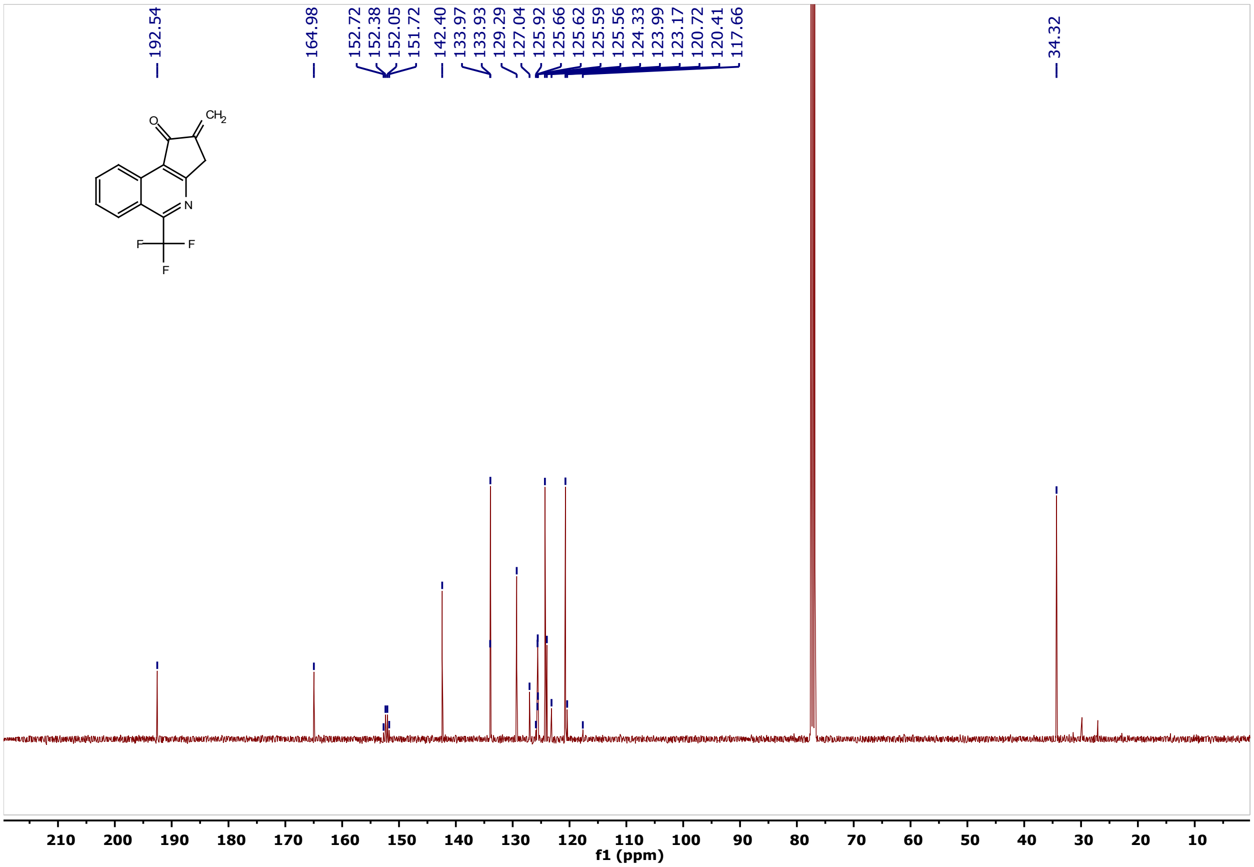

Figure S78. ^{19}F NMR spectrum of **3a** (377 MHz; $CDCl_3$)

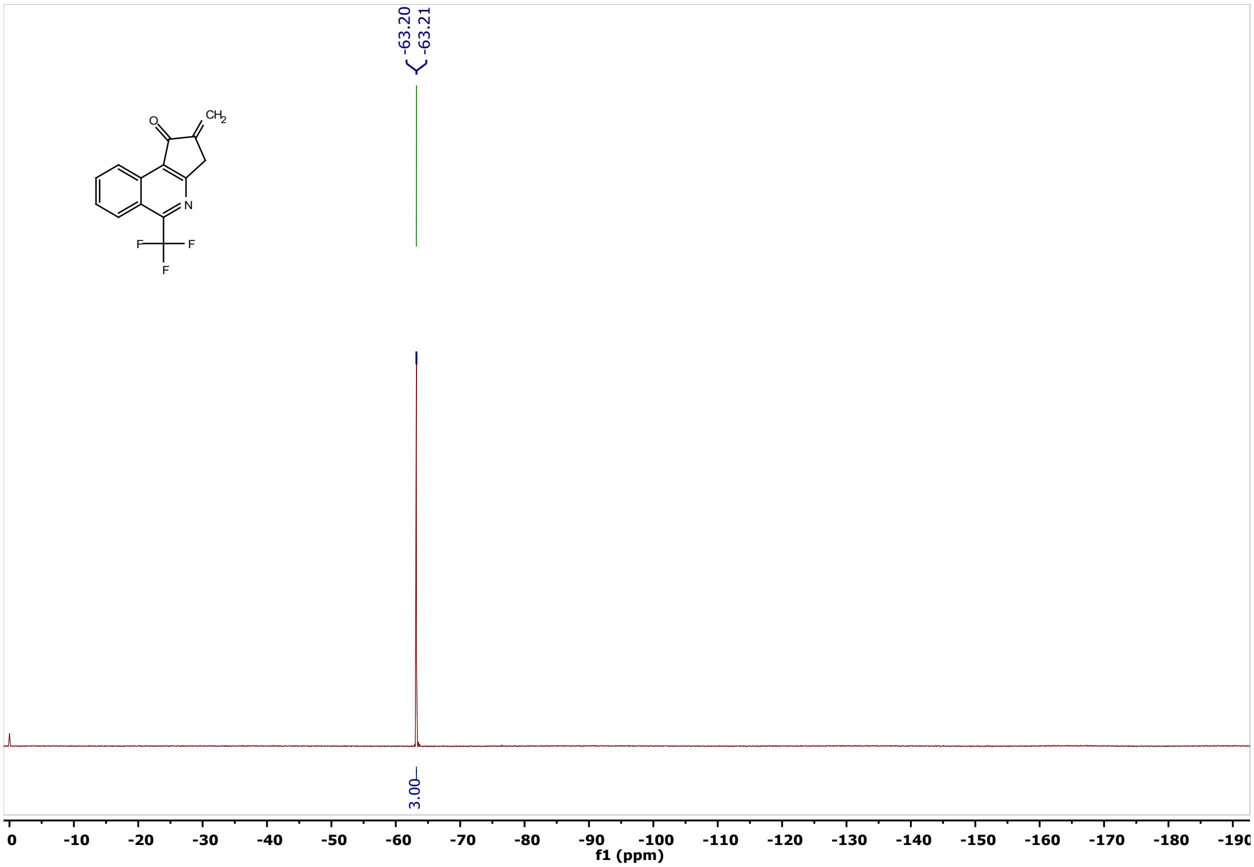

Figure S79. ^{1}H NMR spectrum of **3b** (401 MHz; $CDCl_3$)

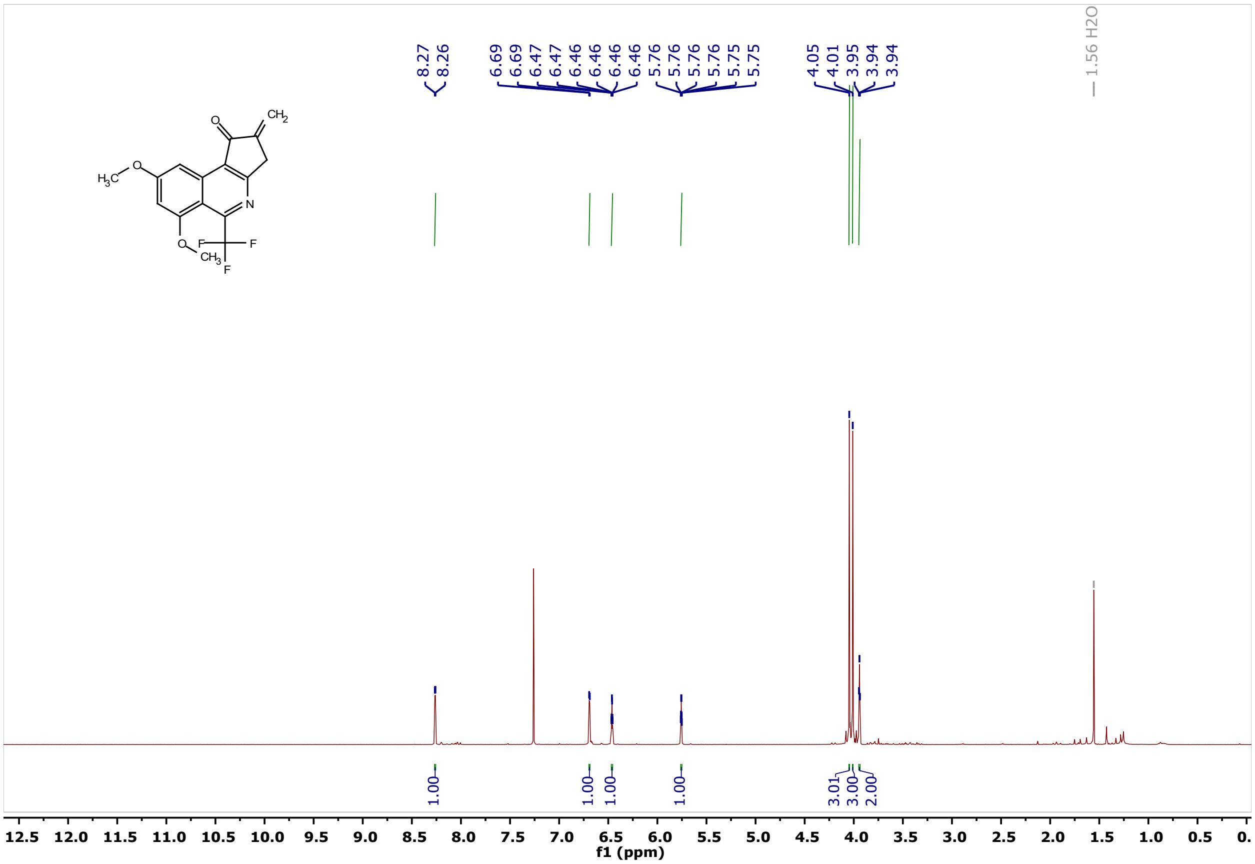

Figure S80. ^{13}C NMR spectrum of **3b** (126 MHz; $CDCl_3$)

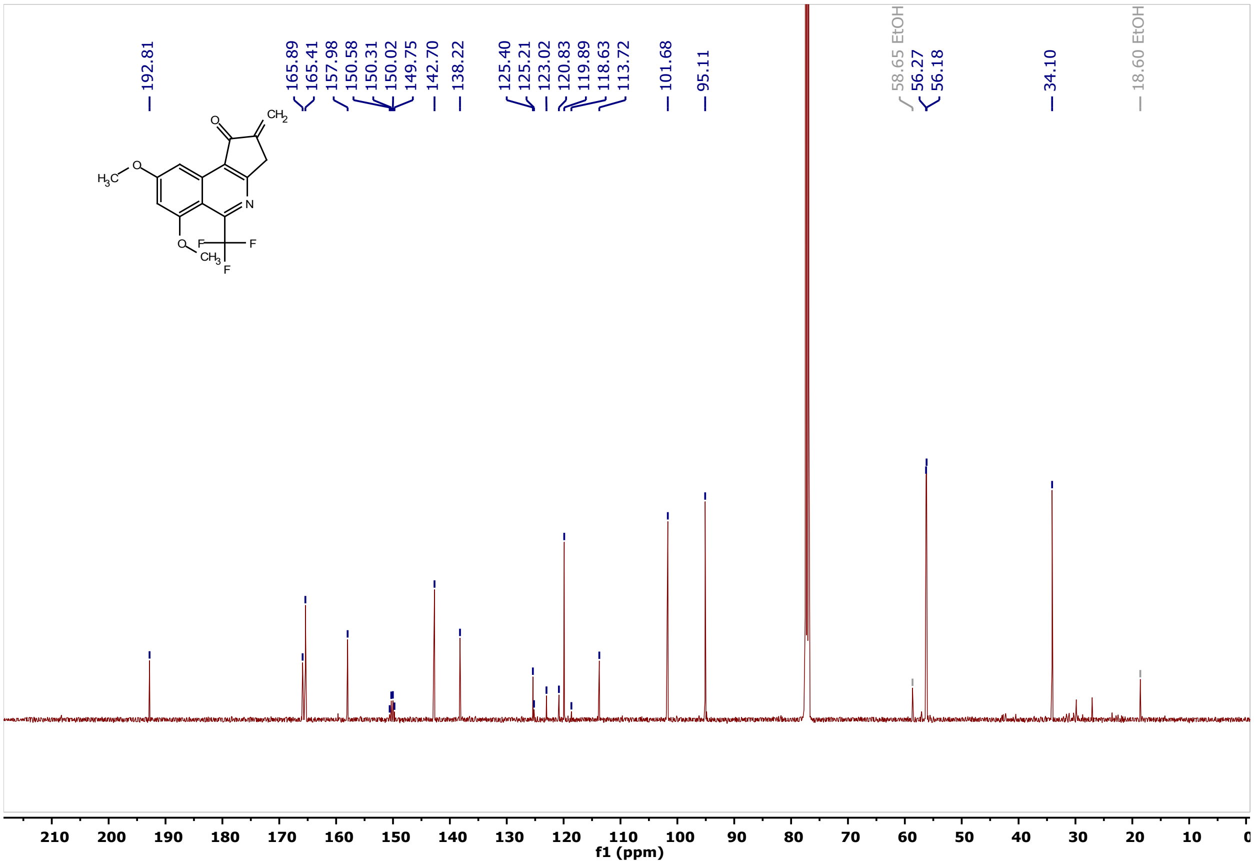

Figure S81. ^{19}F NMR spectrum of **3b** (377 MHz; $CDCl_3$)

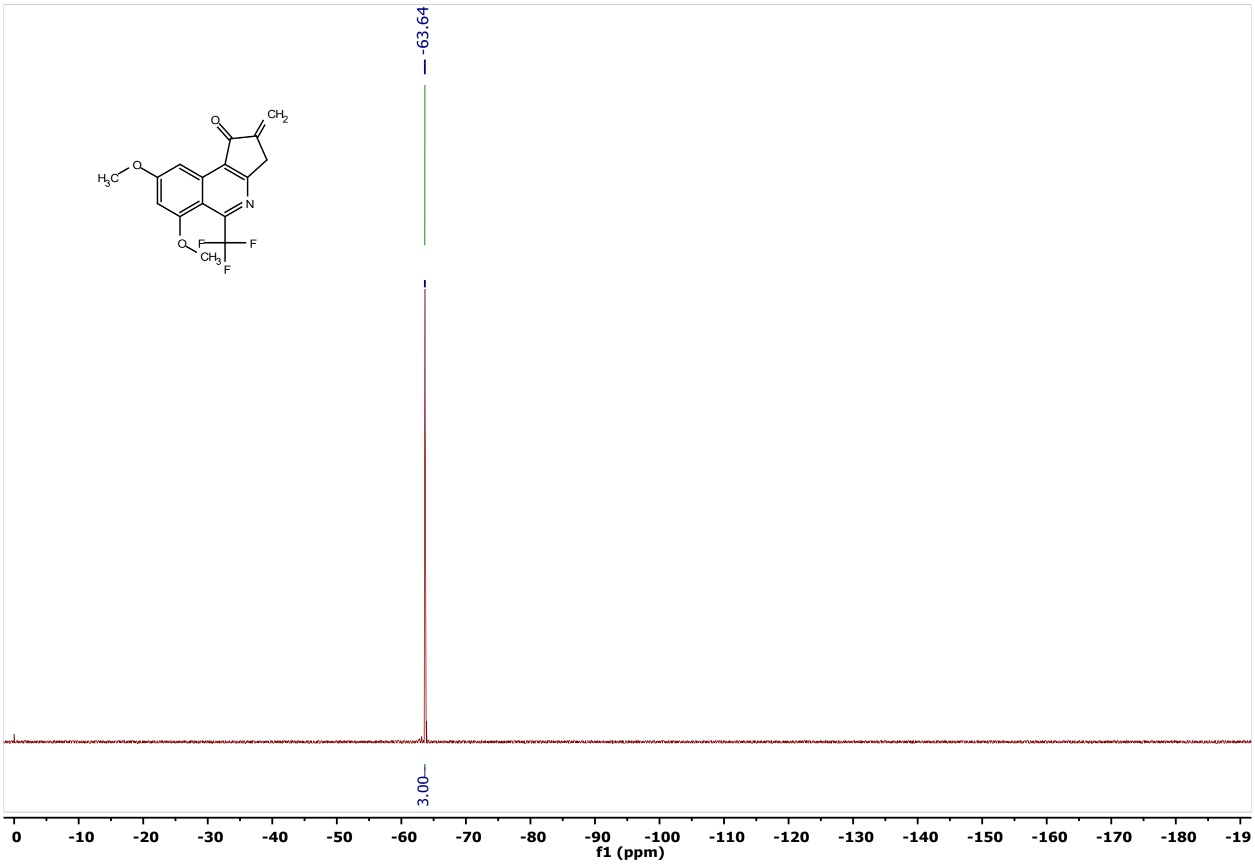

Figure S82. ^{1}H NMR spectrum of **4a** (401 MHz; $CDCl_3$)

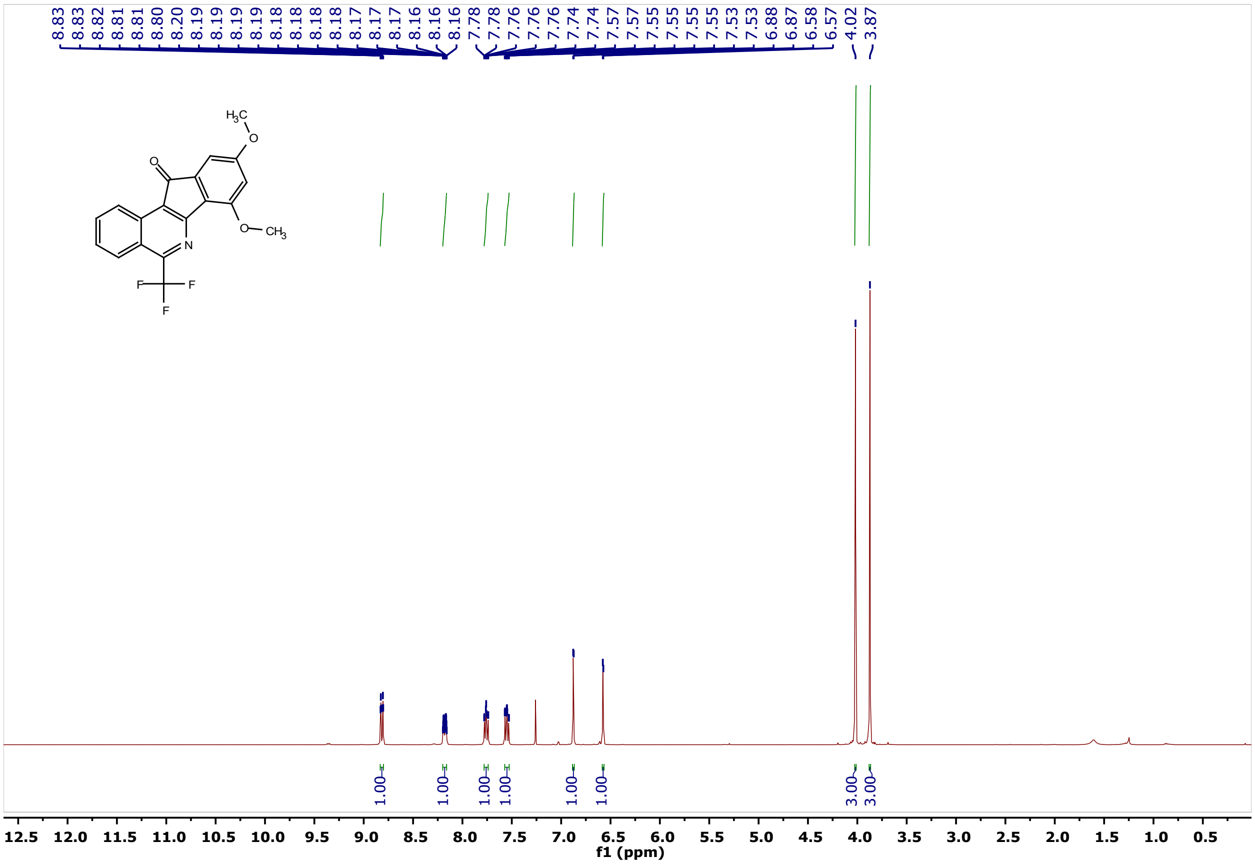

Figure S83. ^{13}C NMR spectrum of **4a** (101 MHz; $CDCl_3$)

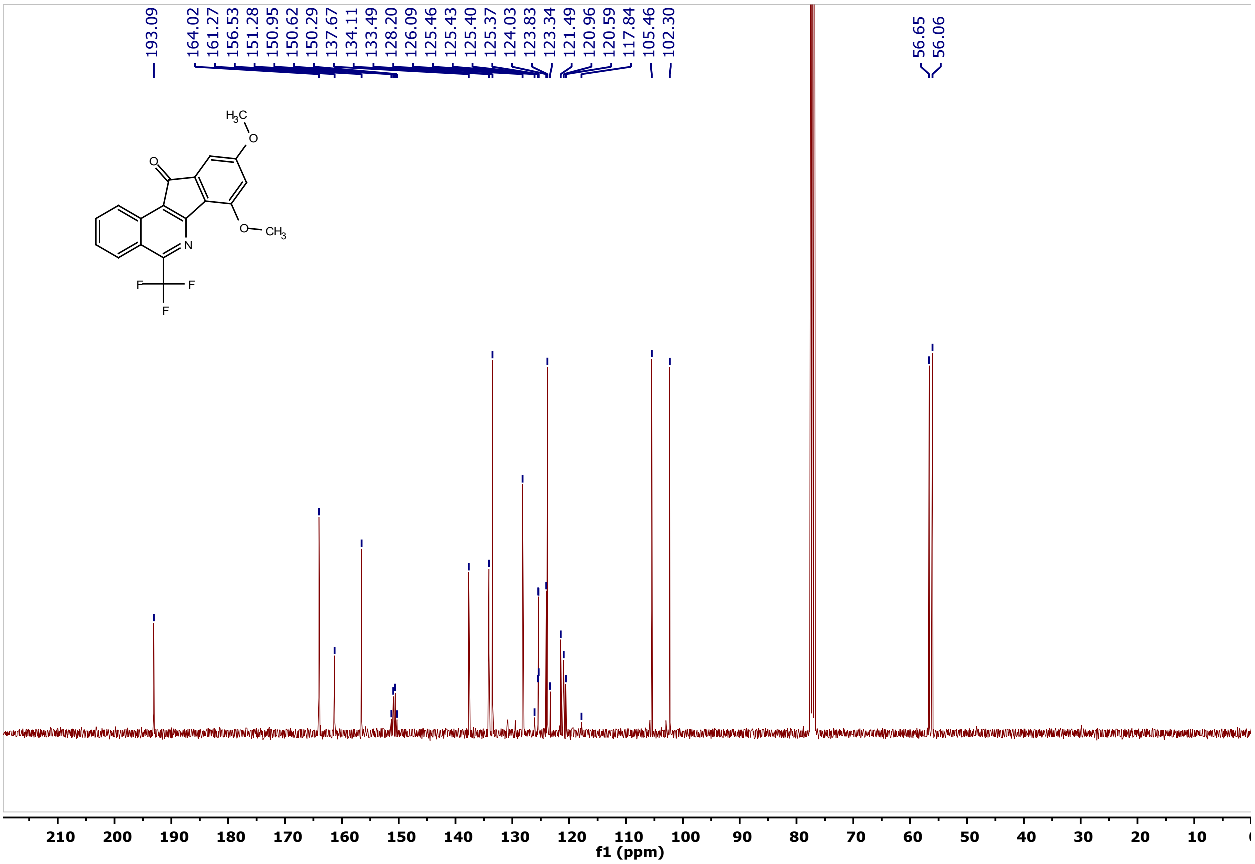

Figure S84. ^{19}F NMR spectrum of **4a** (377 MHz; $CDCl_3$)

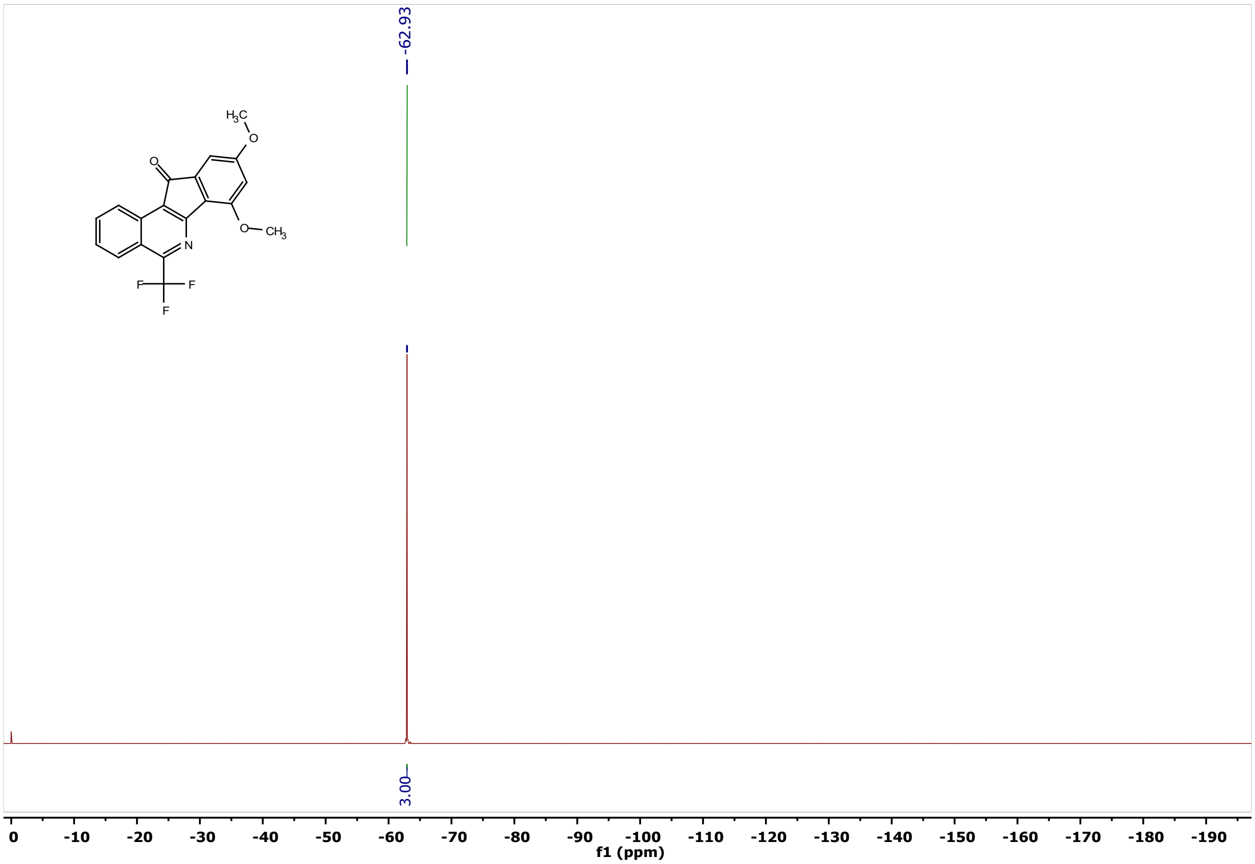

Figure S85. ^{1}H NMR spectrum of **4b** (401 MHz; $CDCl_3$)

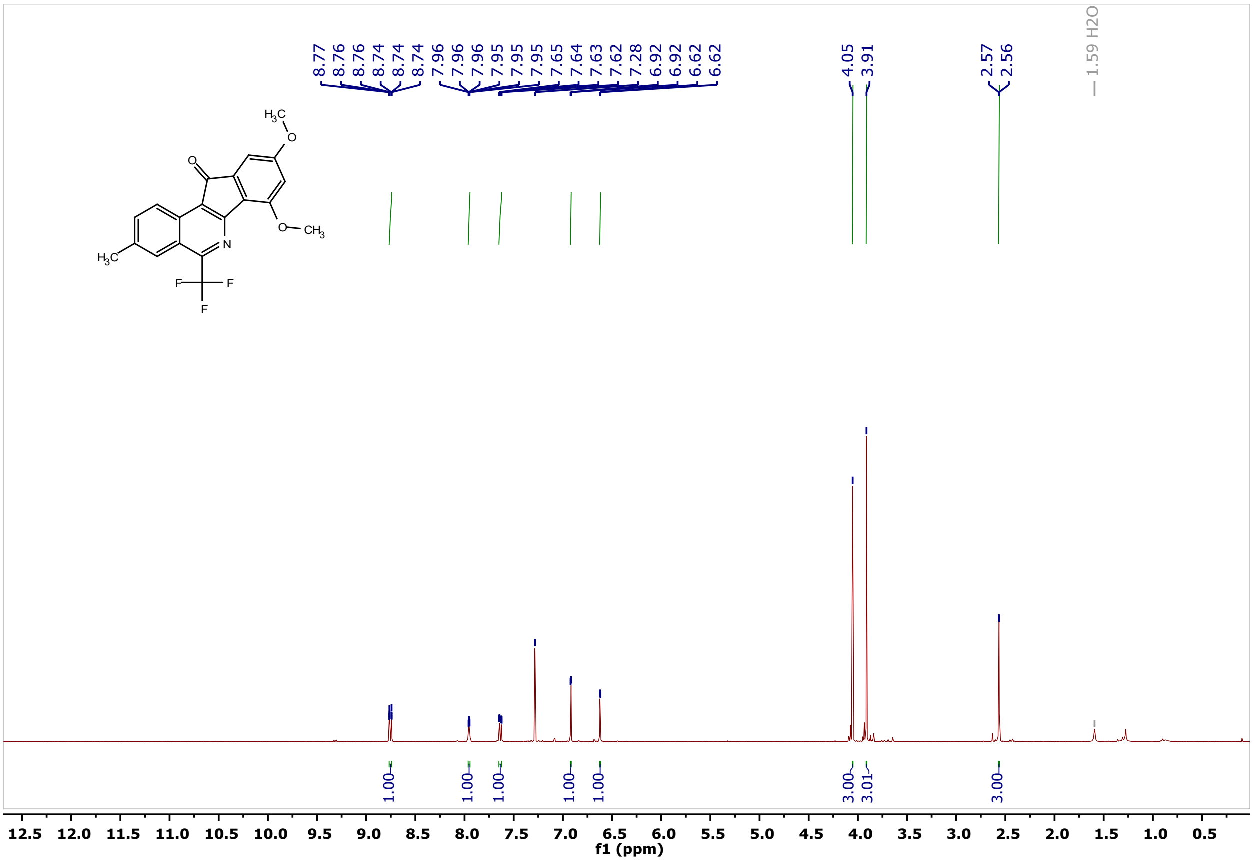

Figure S86. ^{13}C NMR spectrum of **4b** (126 MHz; $CDCl_3$)

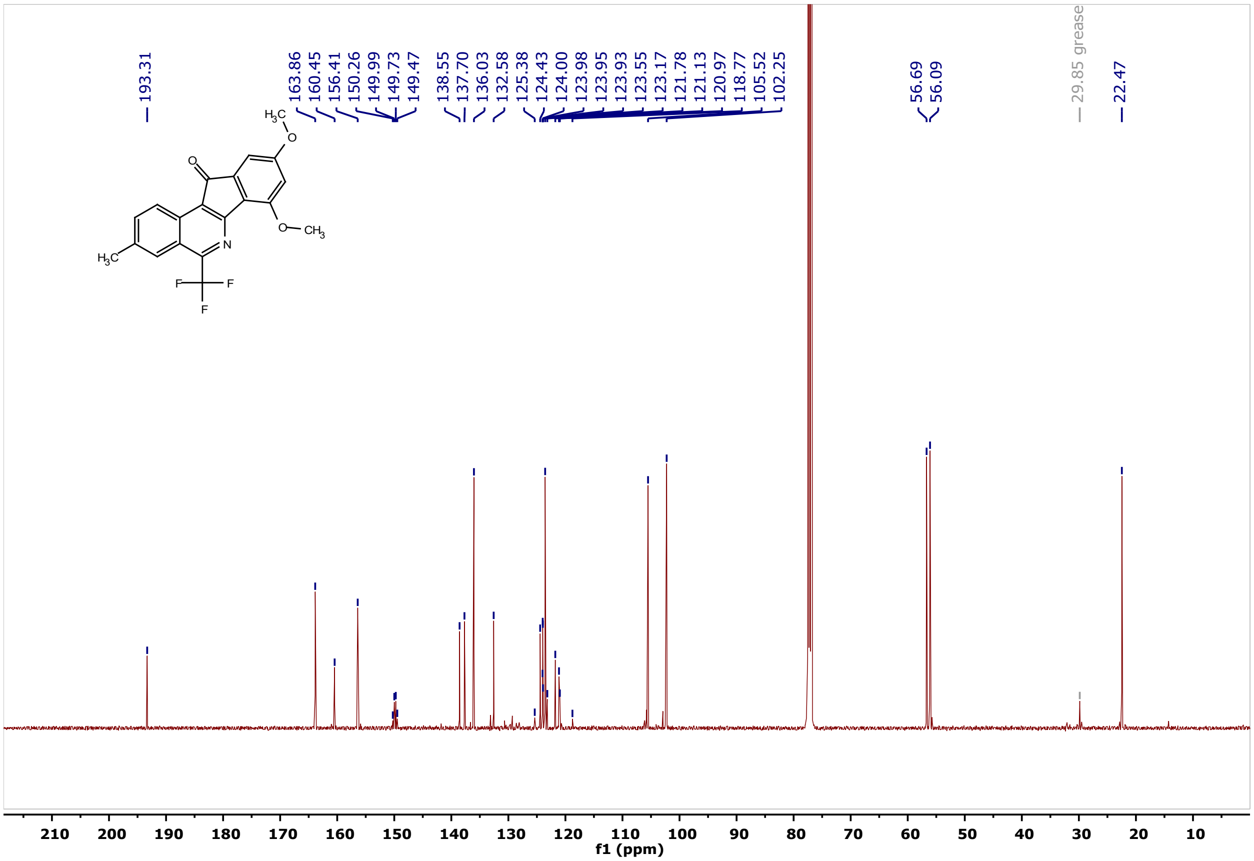

Figure S87. ^{19}F NMR spectrum of **4b** (377 MHz; $CDCl_3$)

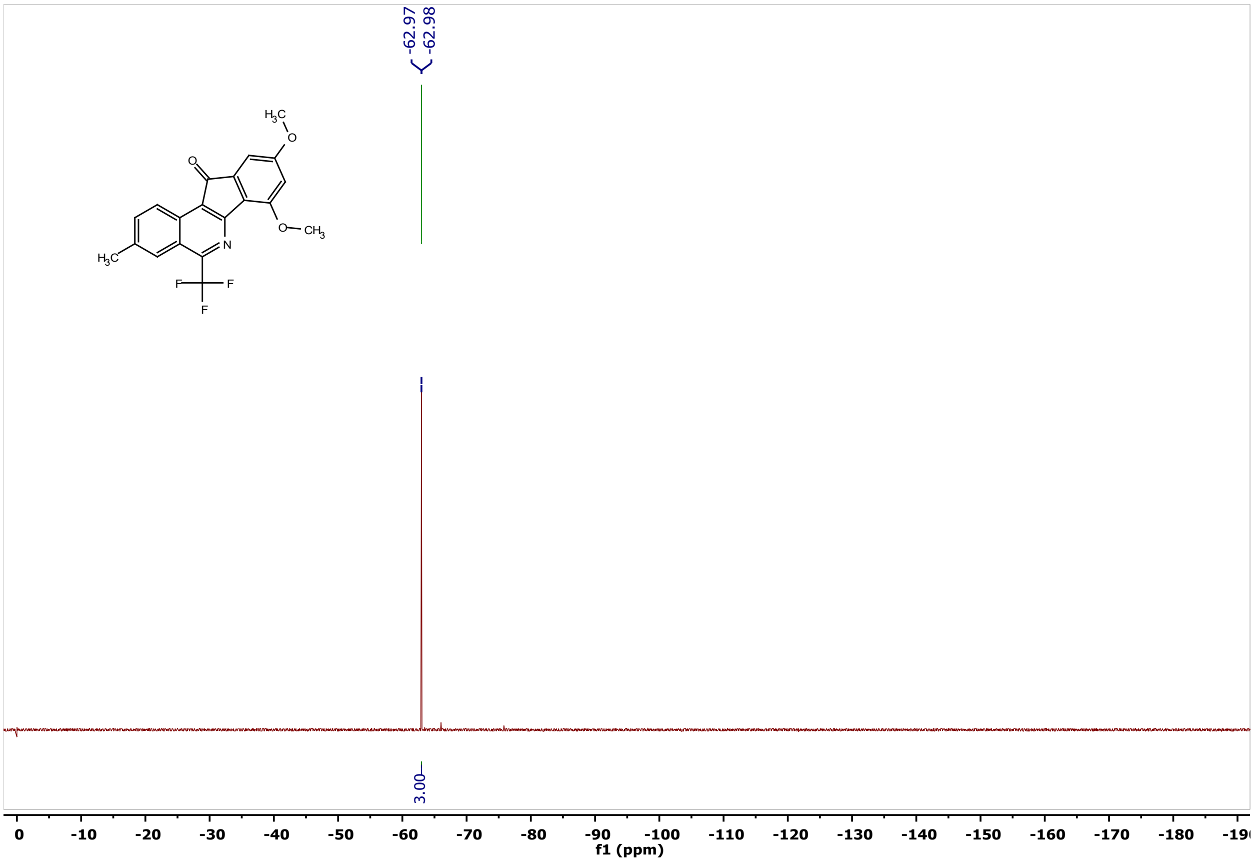

Figure S88. ^{1}H NMR spectrum of **4c** (401 MHz; $CDCl_3$)

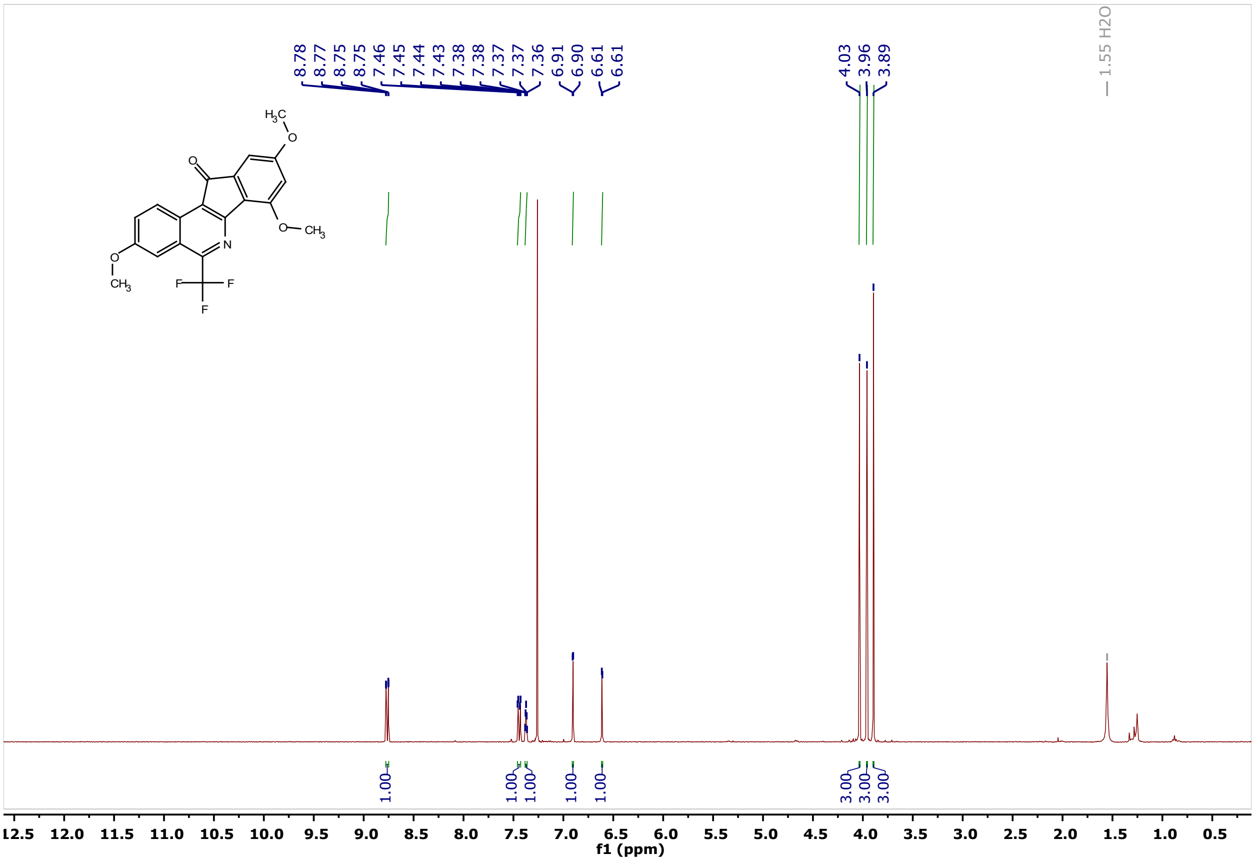

Figure S89. ^{13}C NMR spectrum of **4c** (126 MHz; $CDCl_3$)

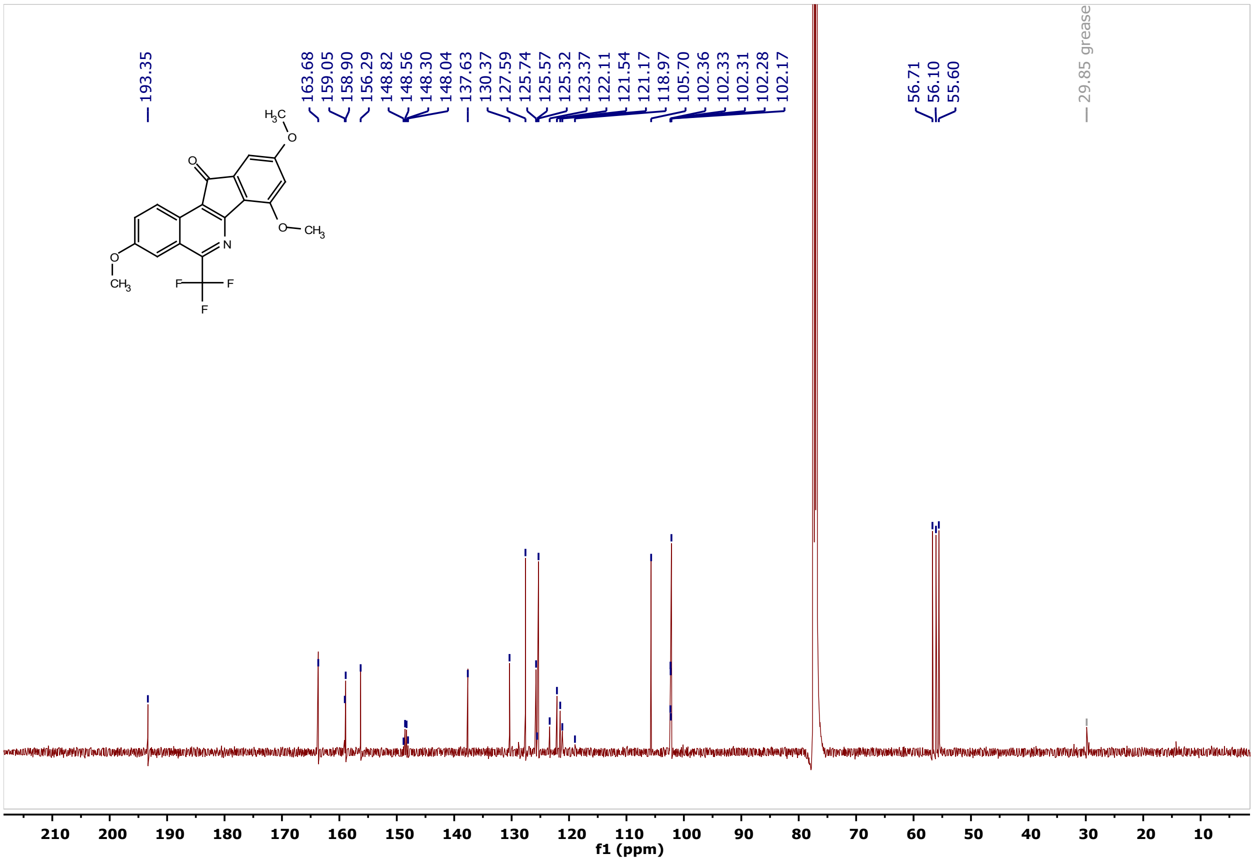

Figure S90. ^{19}F NMR spectrum of **4c** (377 MHz; $CDCl_3$)

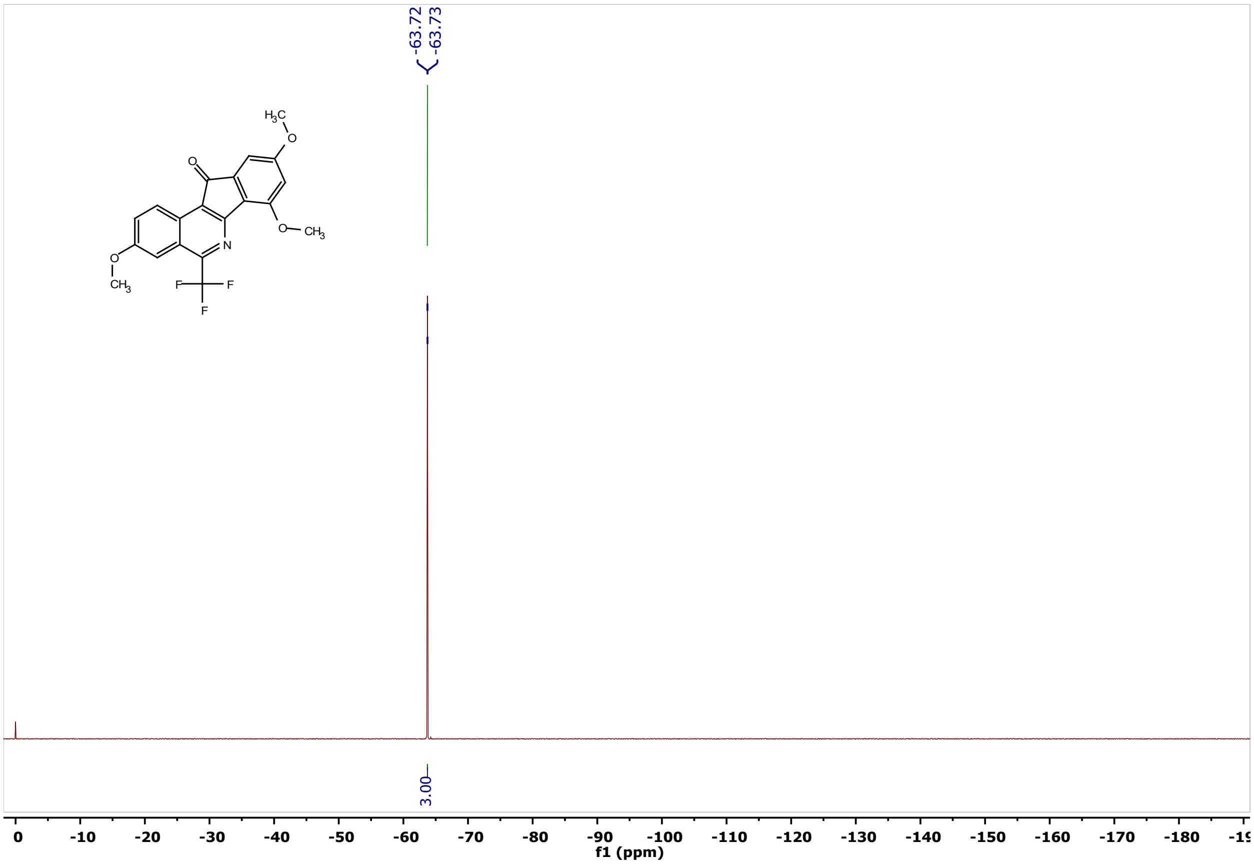

Figure S91. ^{1}H NMR spectrum of **4d** (401 MHz; $CDCl_3$)

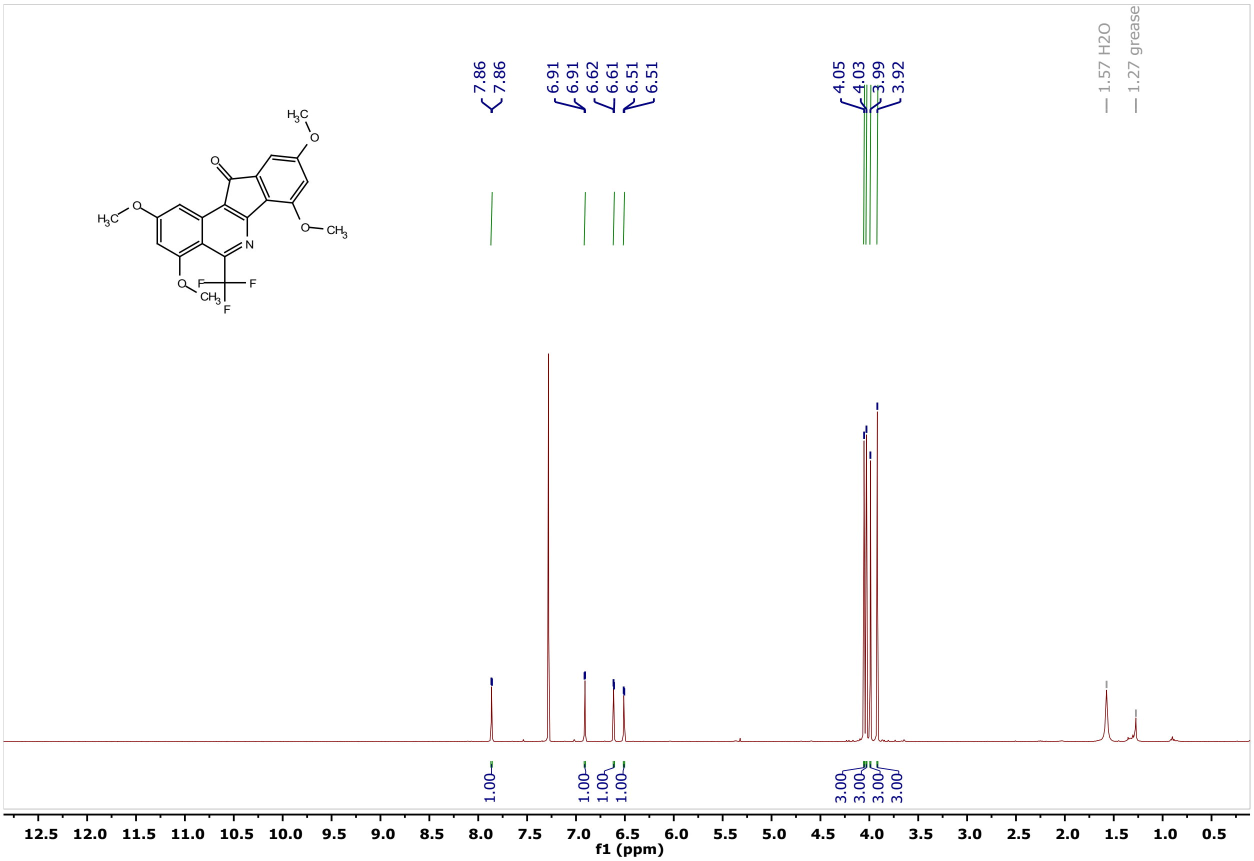

Figure S92. ^{13}C NMR spectrum of **4d** (126 MHz; $CDCl_3$)

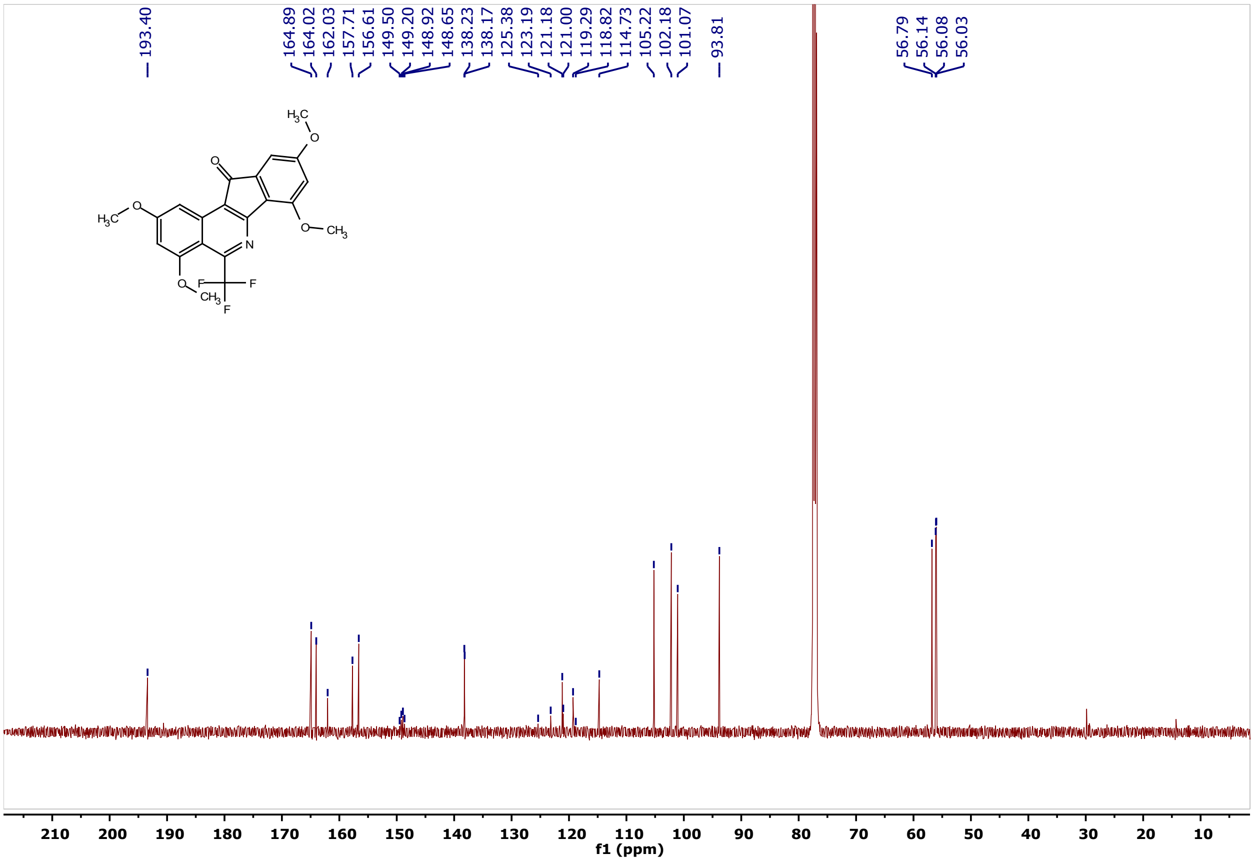

Figure S93. ^{19}F NMR spectrum of **4d** (377 MHz; $CDCl_3$)

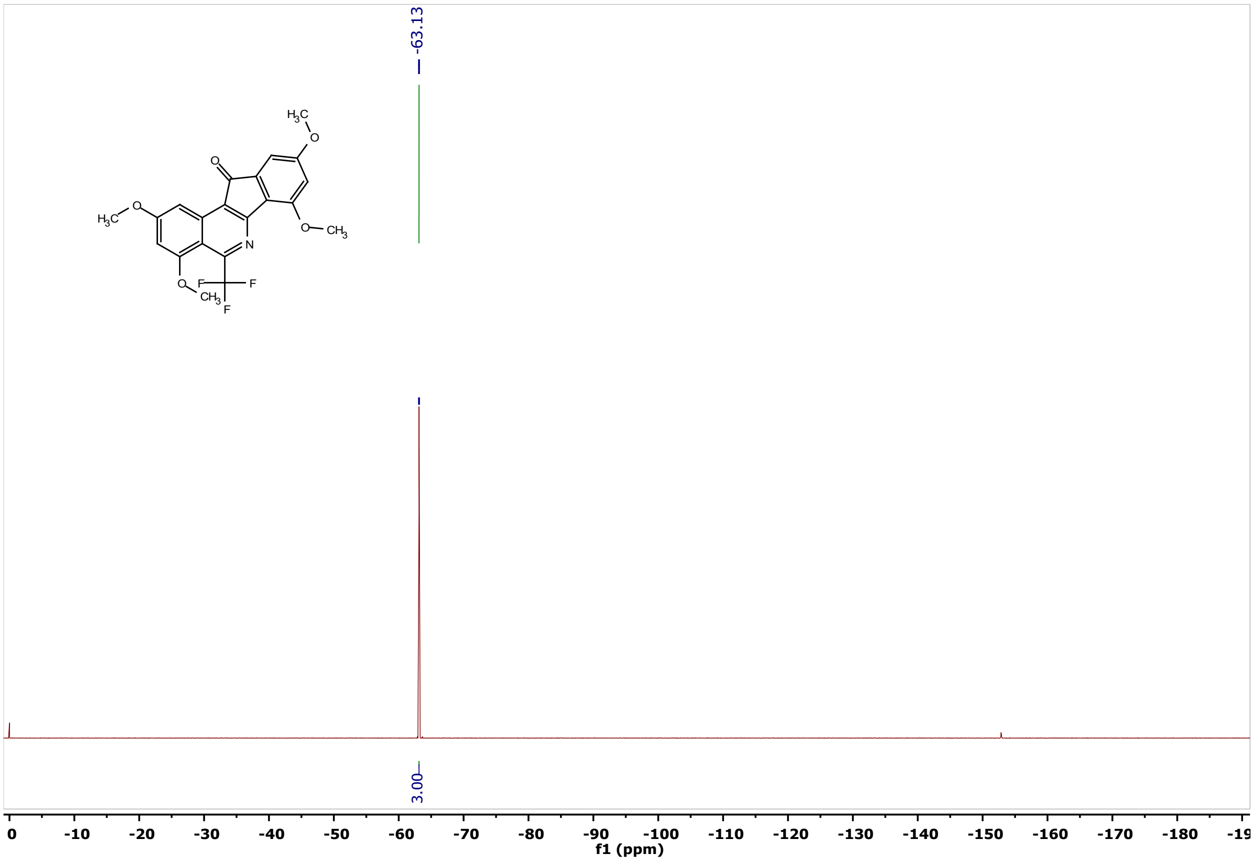

Figure S94. ^{1}H NMR spectrum of **4e** (401 MHz; $CDCl_3$)

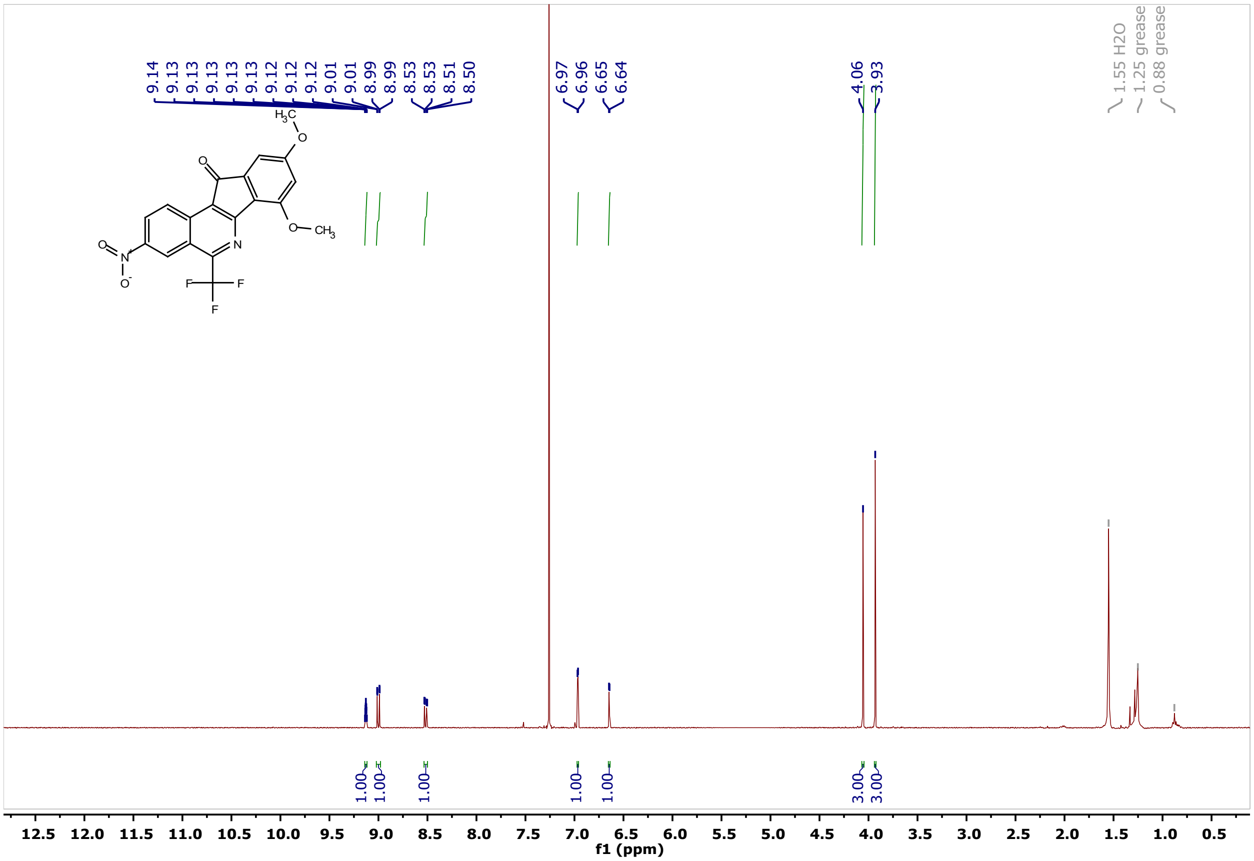

Figure S95. ^{13}C NMR spectrum of **4e** (126 MHz; $CDCl_3$)

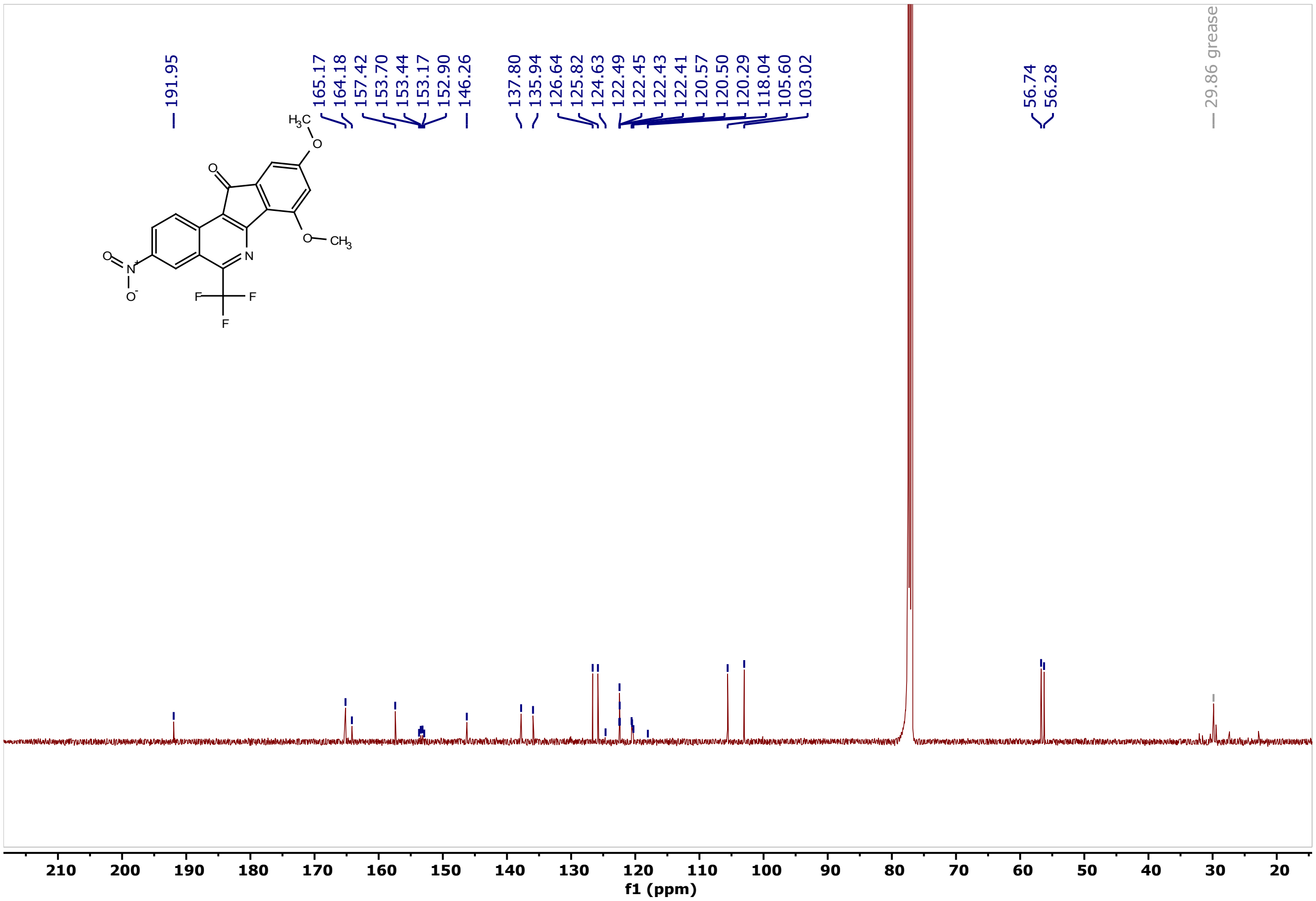

Figure S96. ^{19}F NMR spectrum of **4e** (377 MHz; $CDCl_3$)

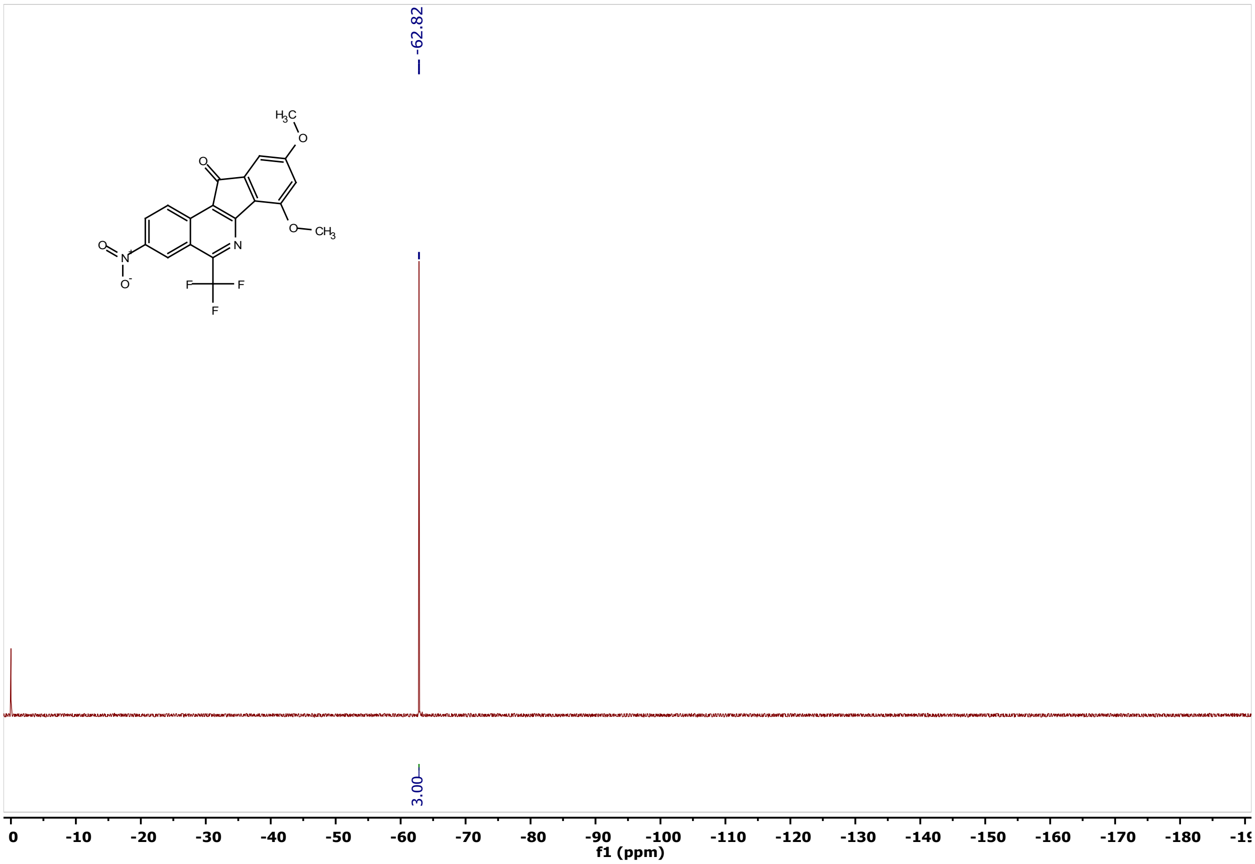

Figure S97. ^{1}H NMR spectrum of **4f** (401 MHz; $CDCl_3$)

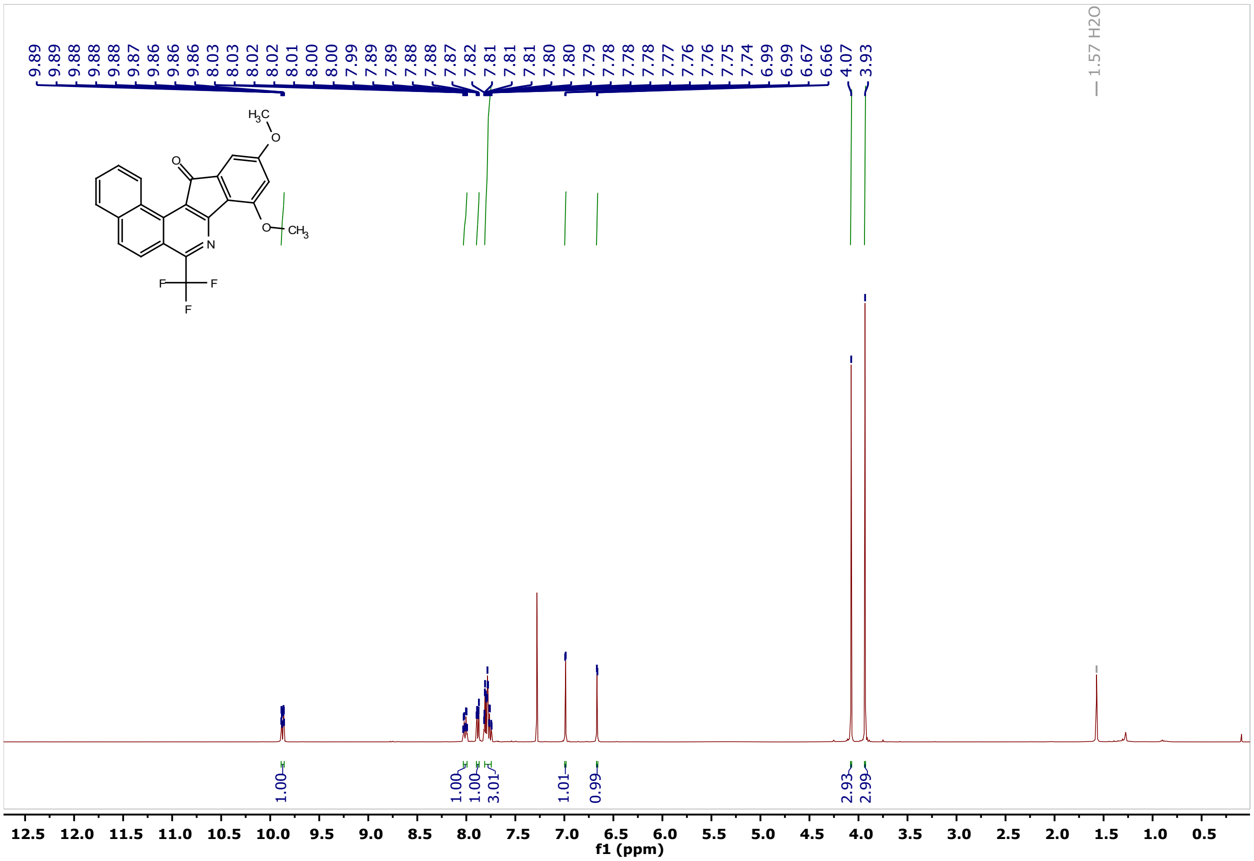

Figure S98. ^{13}C NMR spectrum of **4f** (101 MHz; $CDCl_3$)

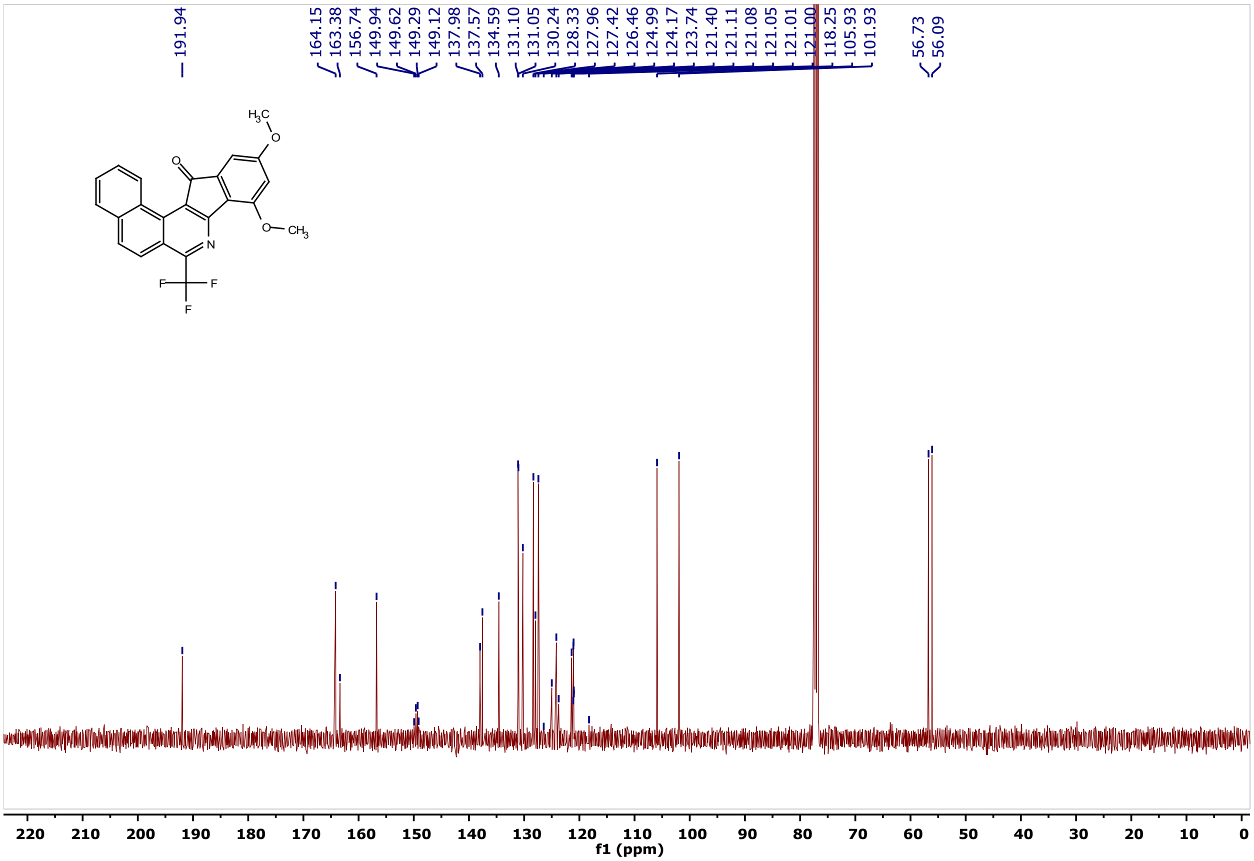

Figure S99. ^{19}F NMR spectrum of **4f** (377 MHz; $CDCl_3$)

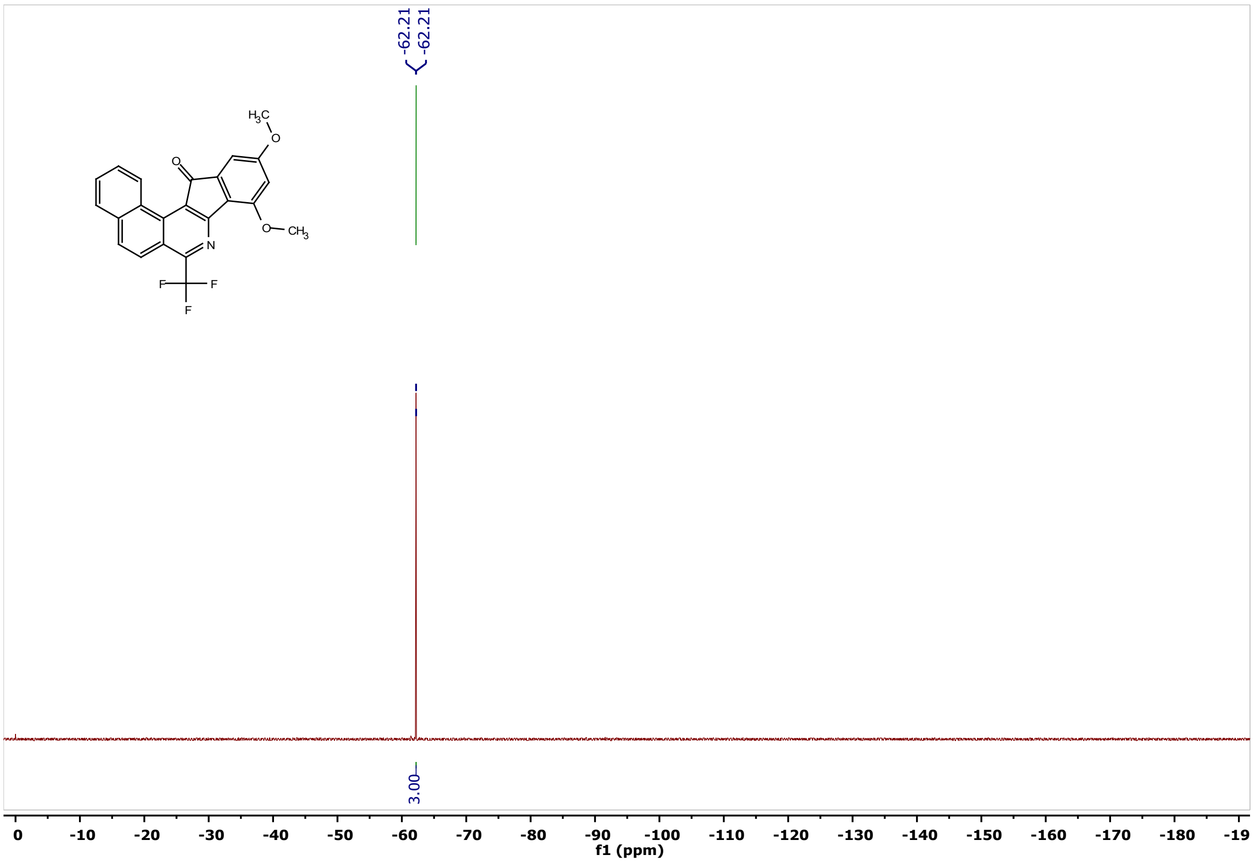

Figure S100. ^{1}H NMR spectrum of **5a** (401 MHz; $CDCl_3$)

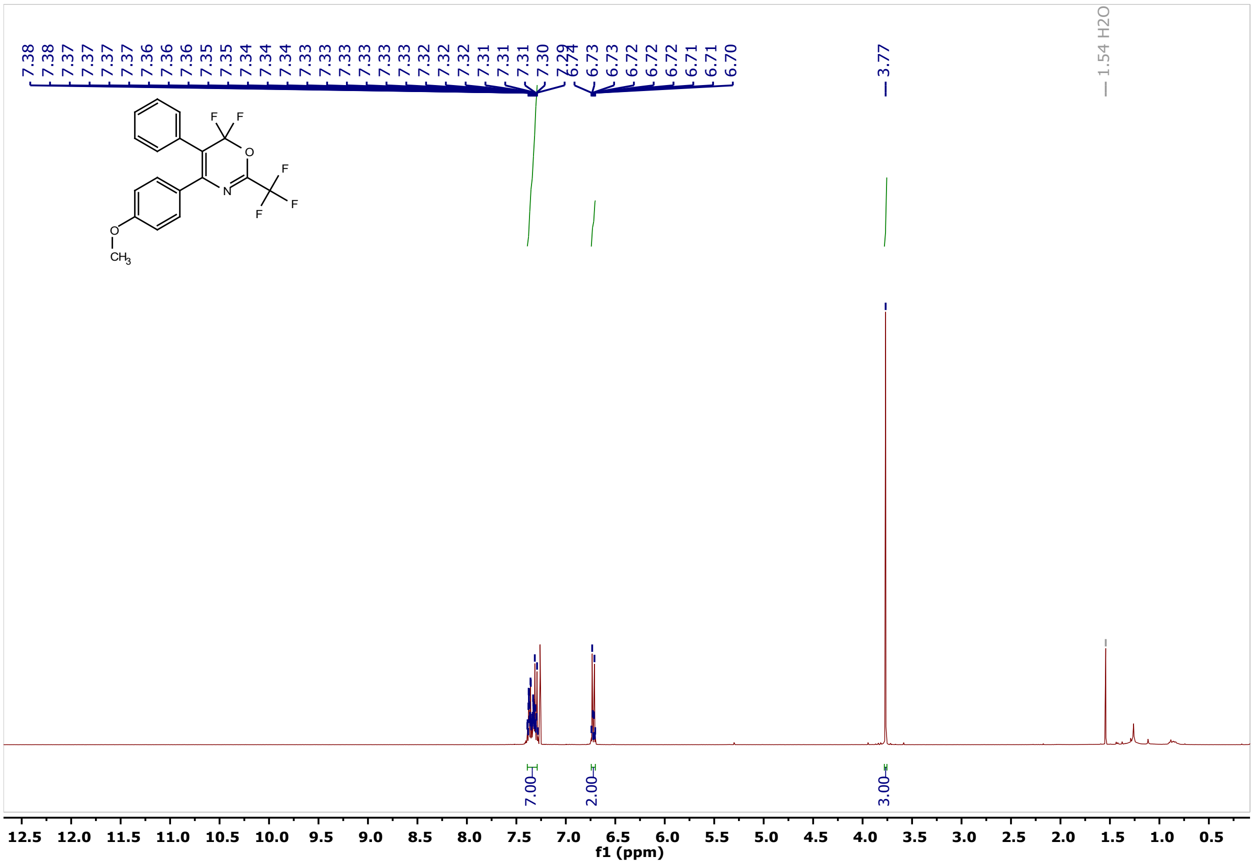

Figure S101. ^{13}C NMR spectrum of **5a** (101 MHz; $CDCl_3$)

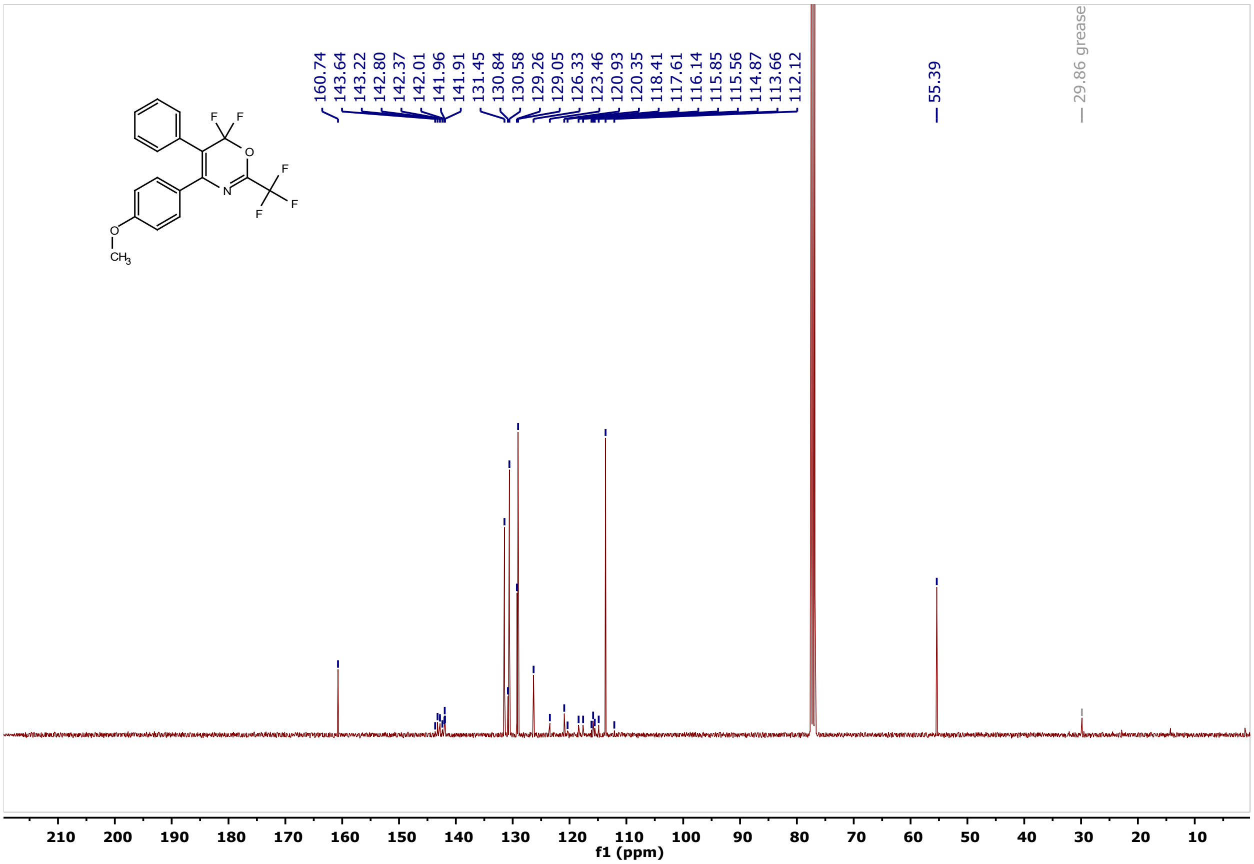

Figure S102. ^{19}F NMR spectrum of **5a** (377 MHz; $CDCl_3$)

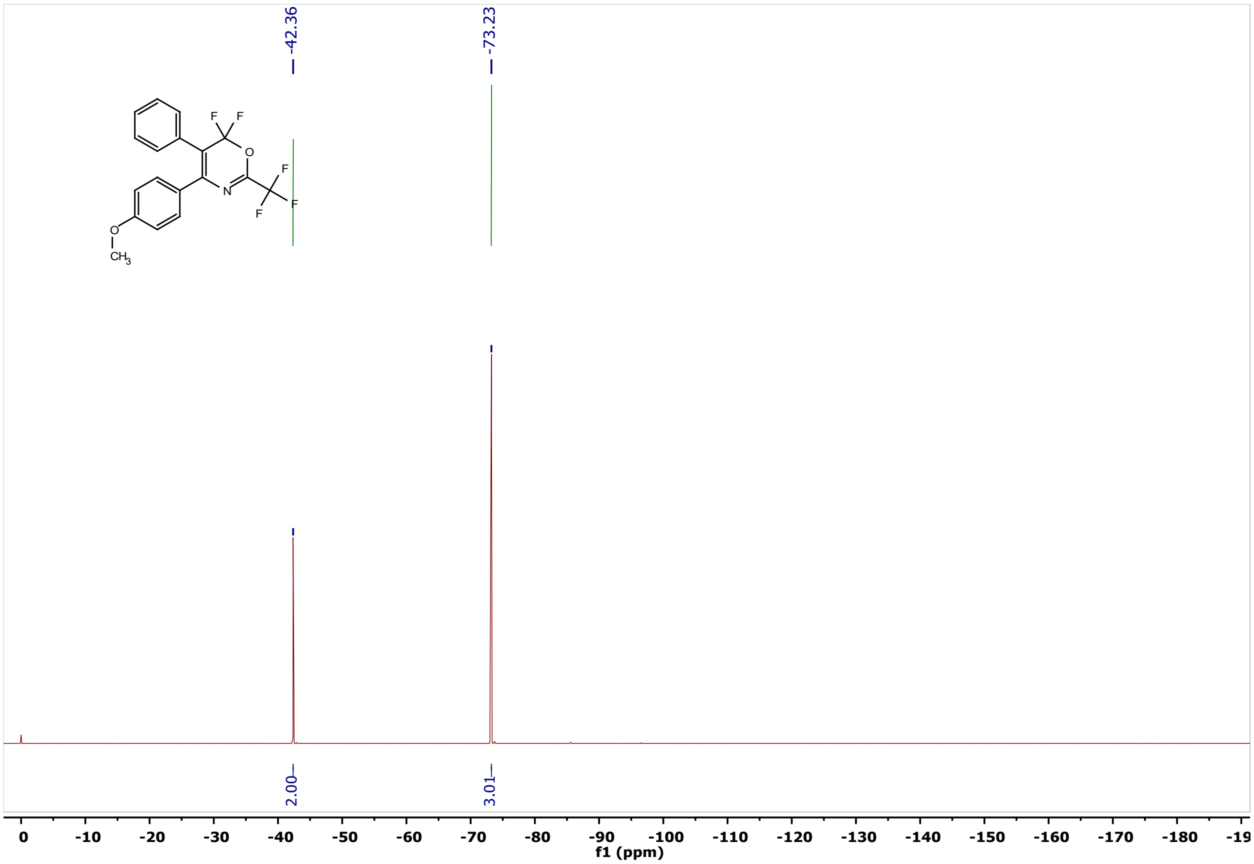

Figure S103. ^{1}H NMR spectrum of **5b** (401 MHz; $CDCl_3$)

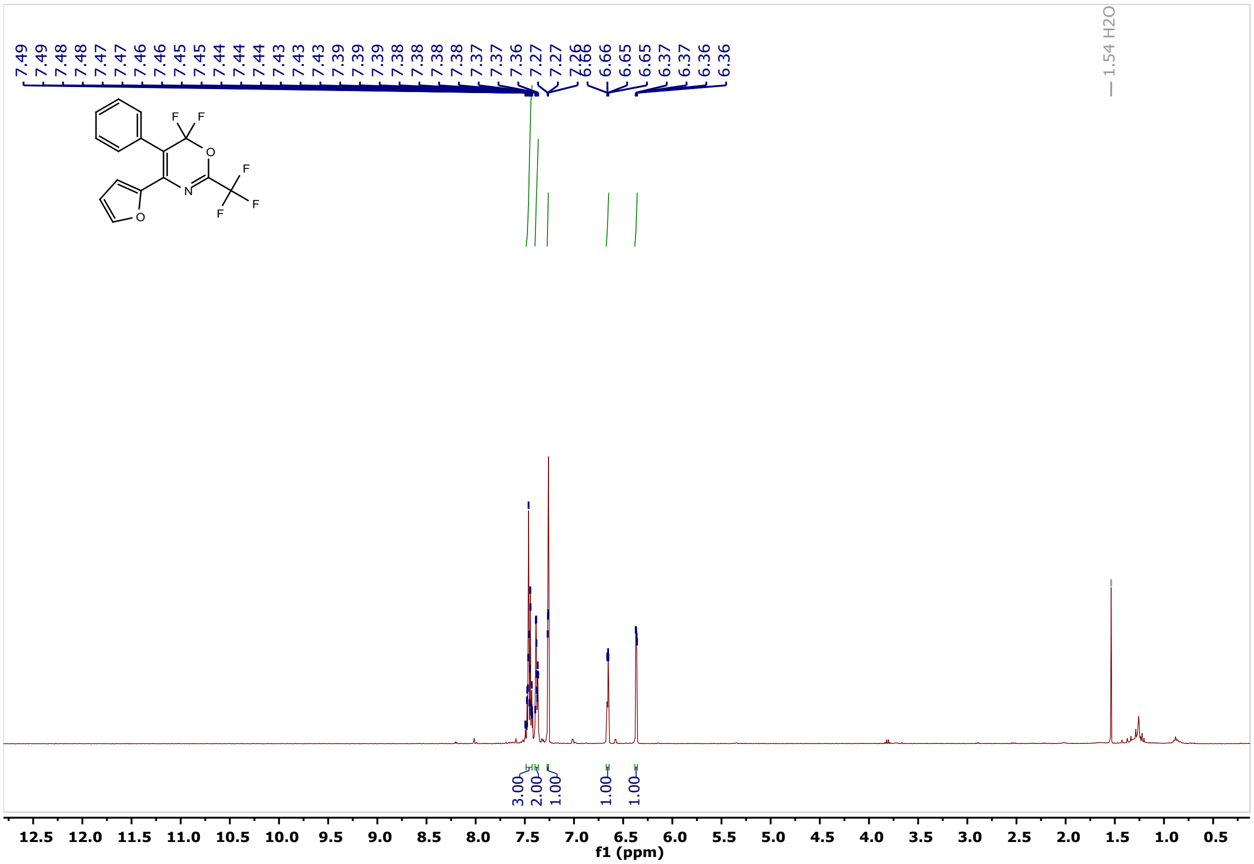

Figure S104. ^{13}C NMR spectrum of **5b** (101 MHz; $CDCl_3$)

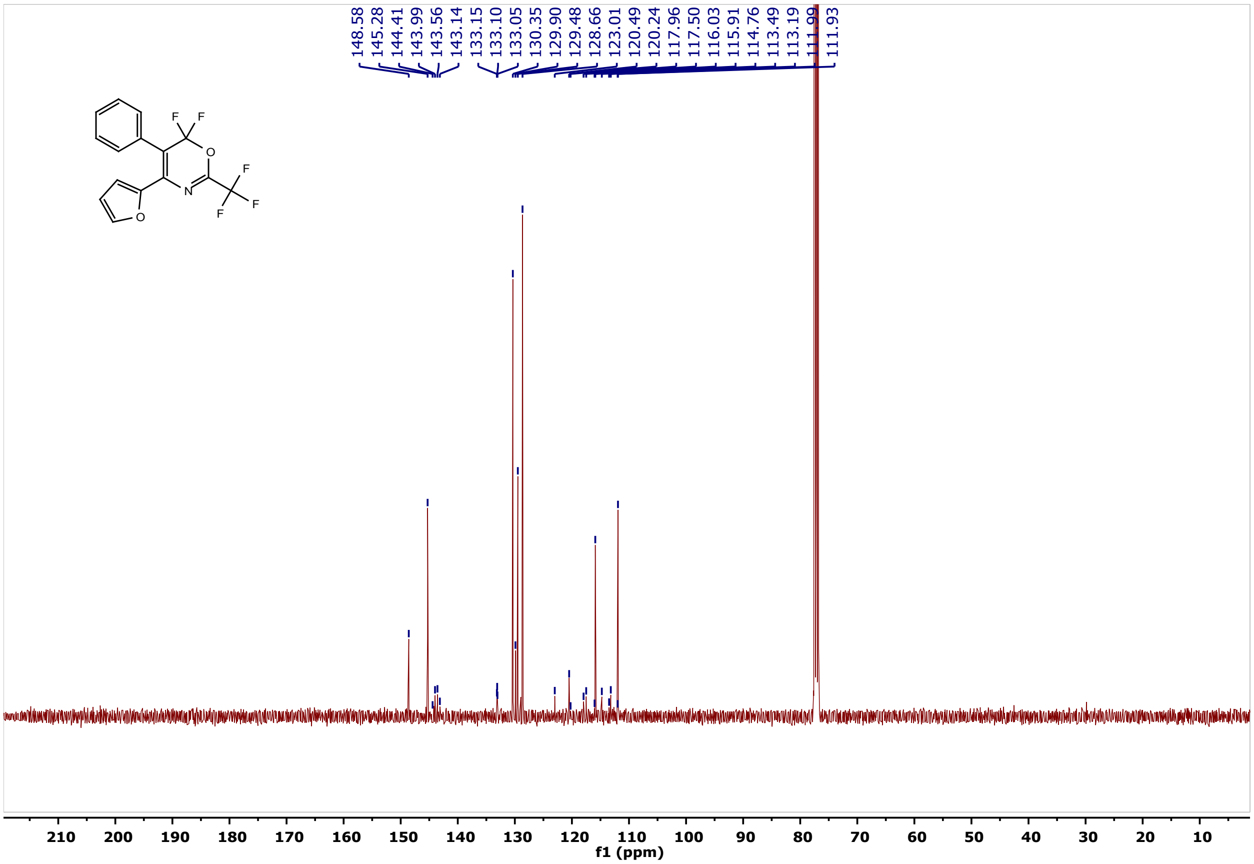

Figure S105. ^{19}F NMR spectrum of **5b** (377 MHz; $CDCl_3$)

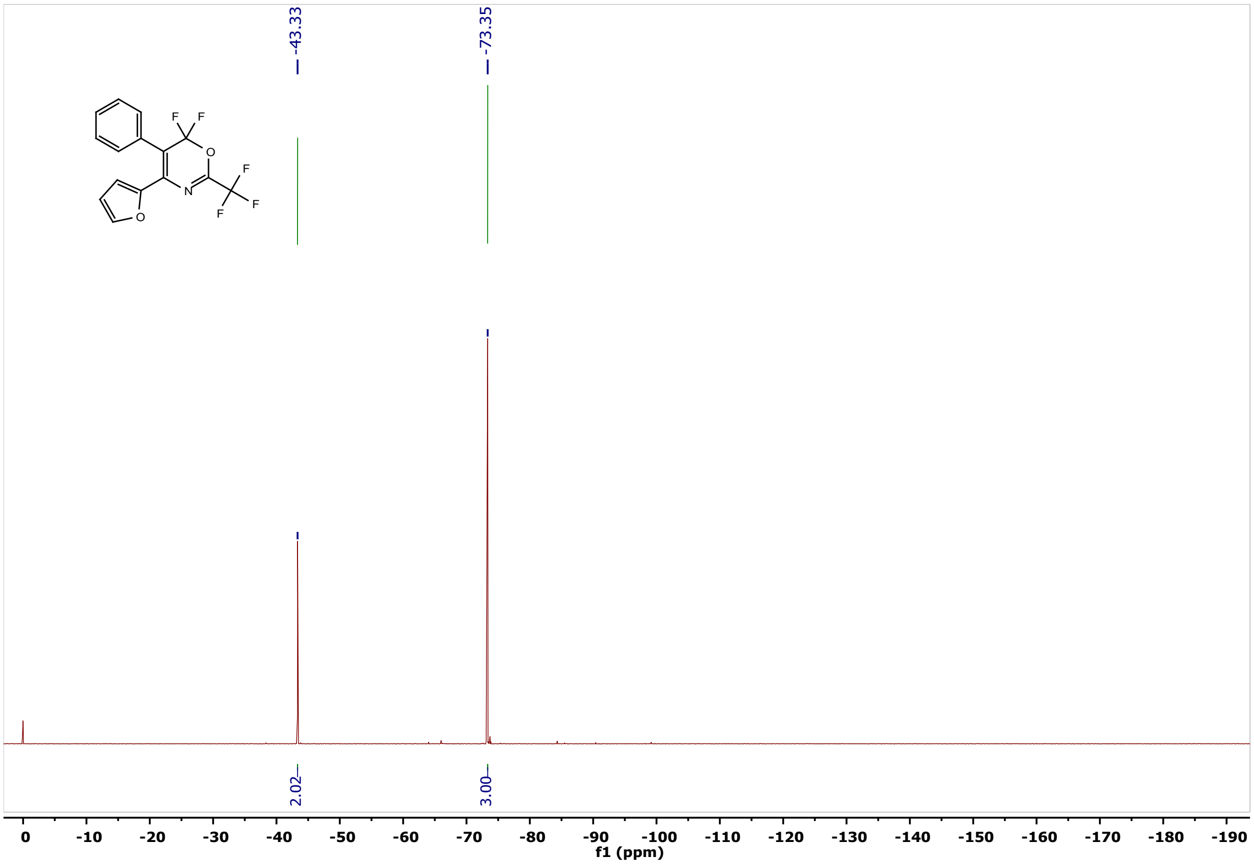

Figure S106. ^{1}H NMR spectrum of **5c** (401 MHz; $CDCl_3$)

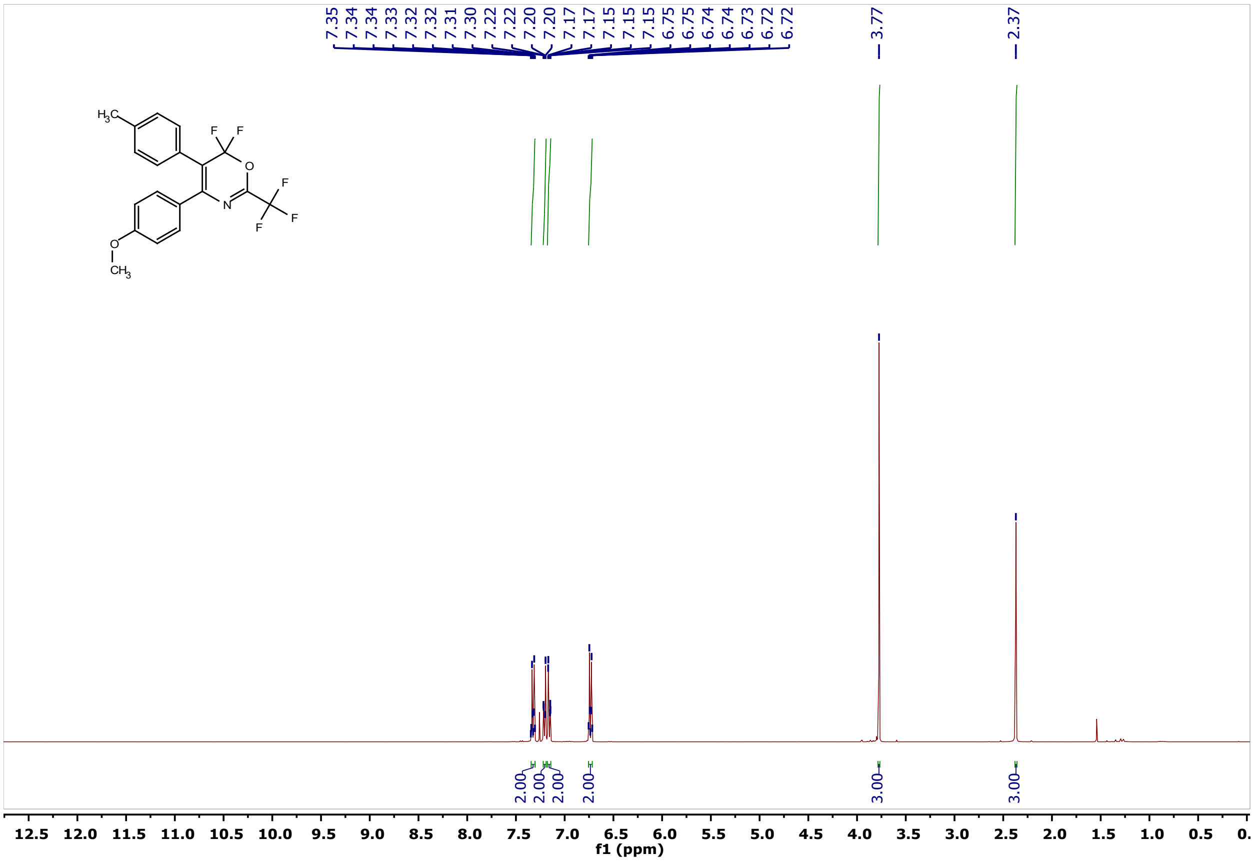

Figure S107. ^{13}C NMR spectrum of **5c** (101 MHz; $CDCl_3$)

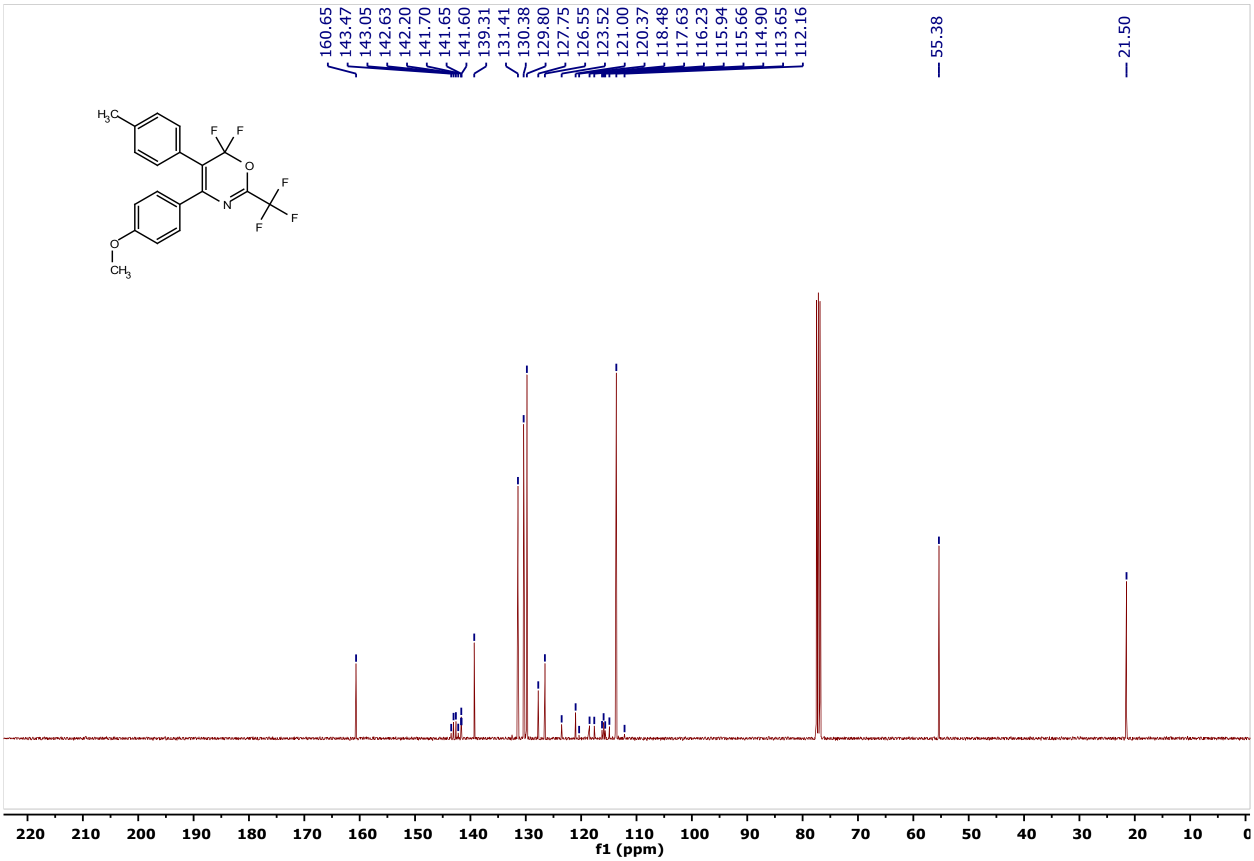

Figure S108. ^{19}F NMR spectrum of **5c** (377 MHz; $CDCl_3$)

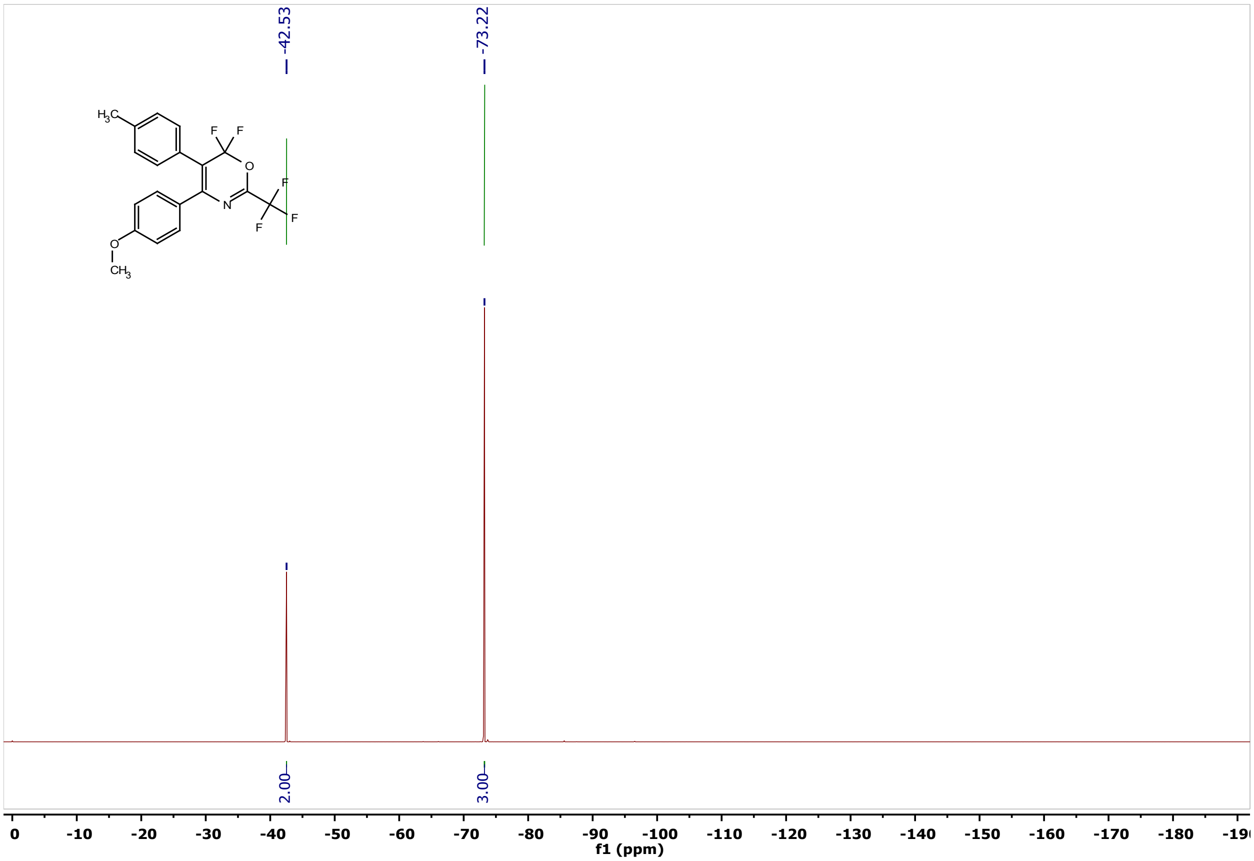

Figure S109. ^{1}H NMR spectrum of **5d** (401 MHz; $CDCl_3$)

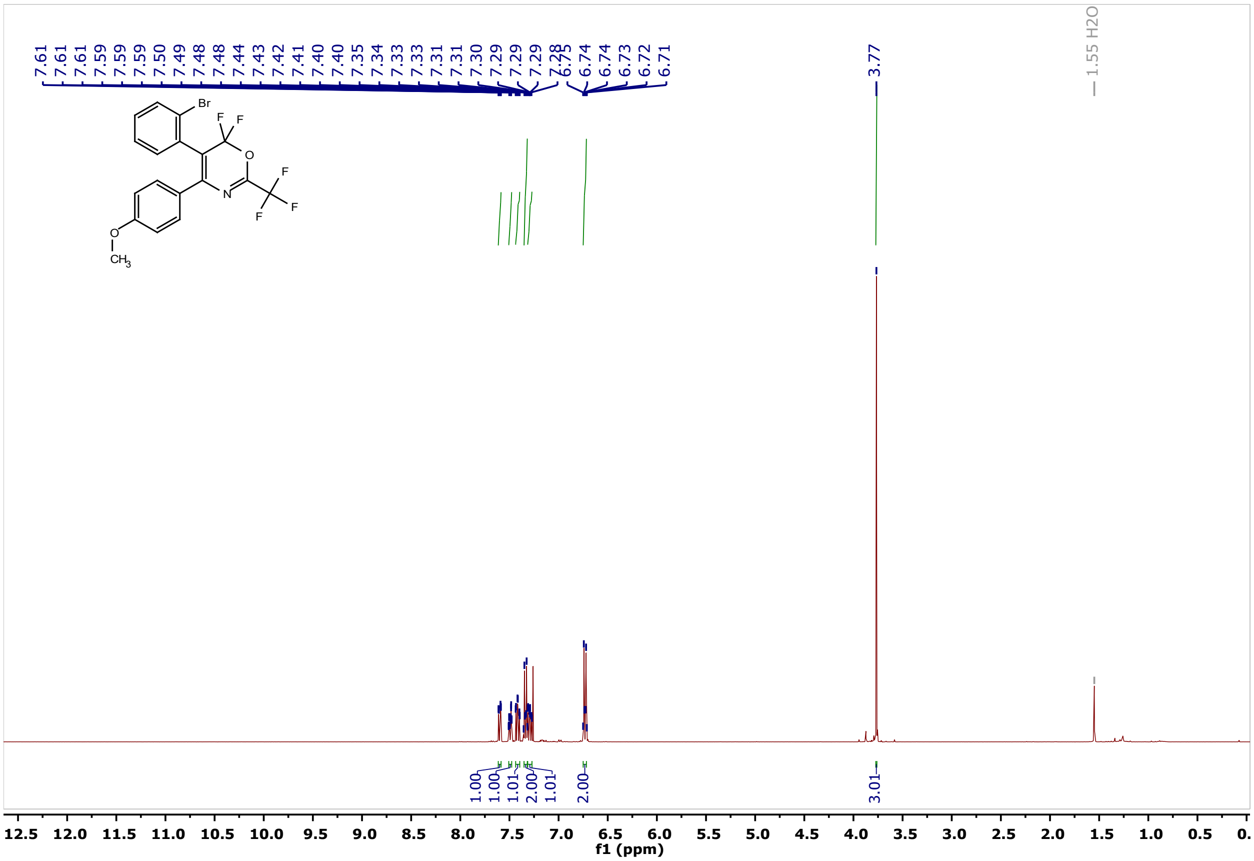

Figure S110. ^{13}C NMR spectrum of **5d** (101 MHz; $CDCl_3$)

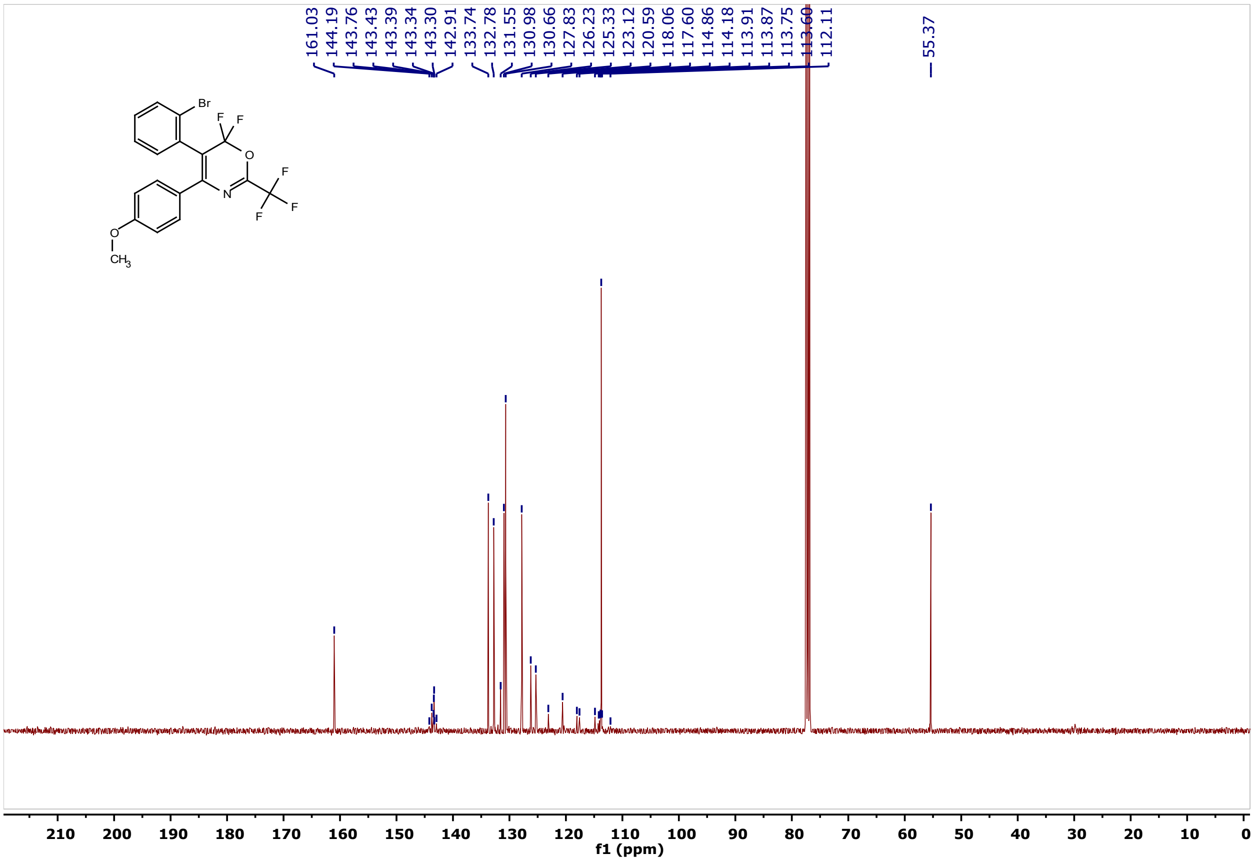

Figure S111. ^{19}F NMR spectrum of **5d** (377 MHz; $CDCl_3$)

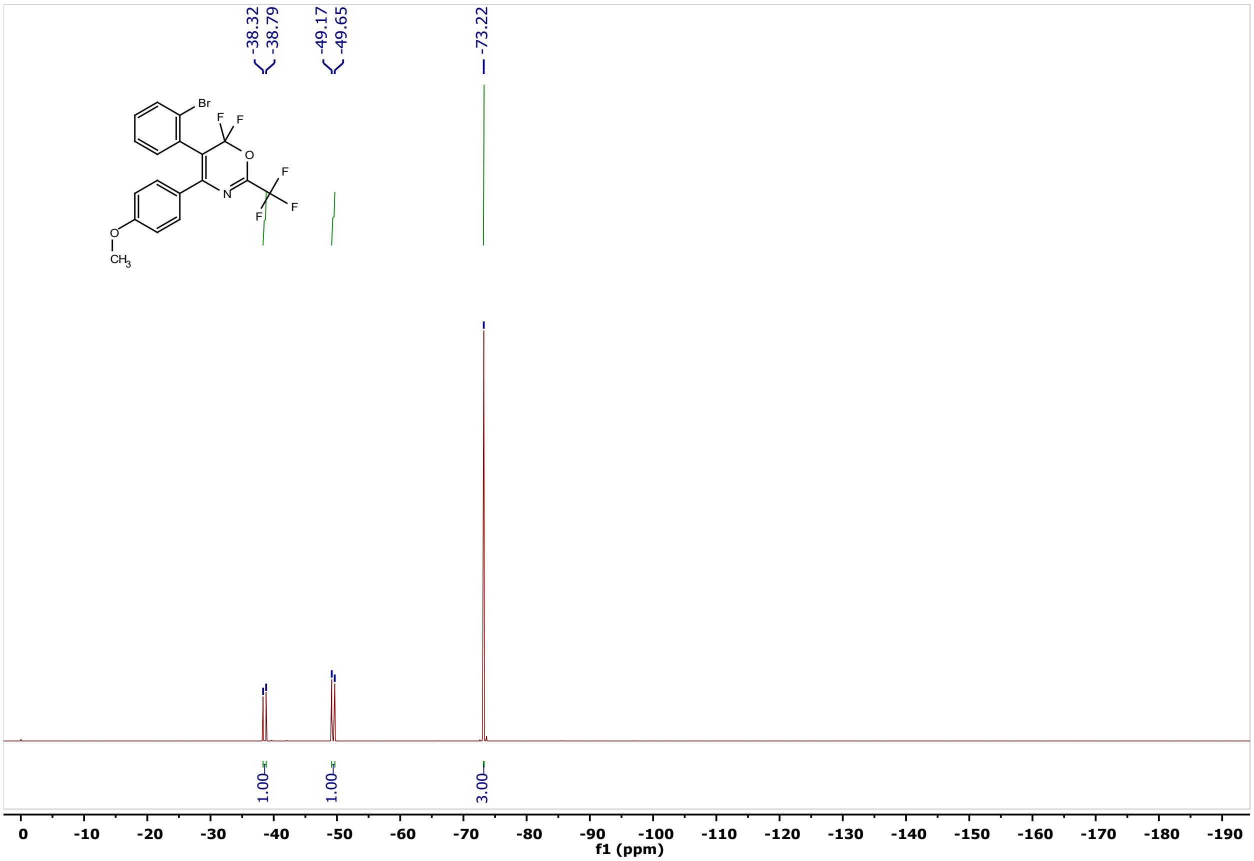

Figure S112. ^{1}H NMR spectrum of **6a** (401 MHz; $CDCl_3$)

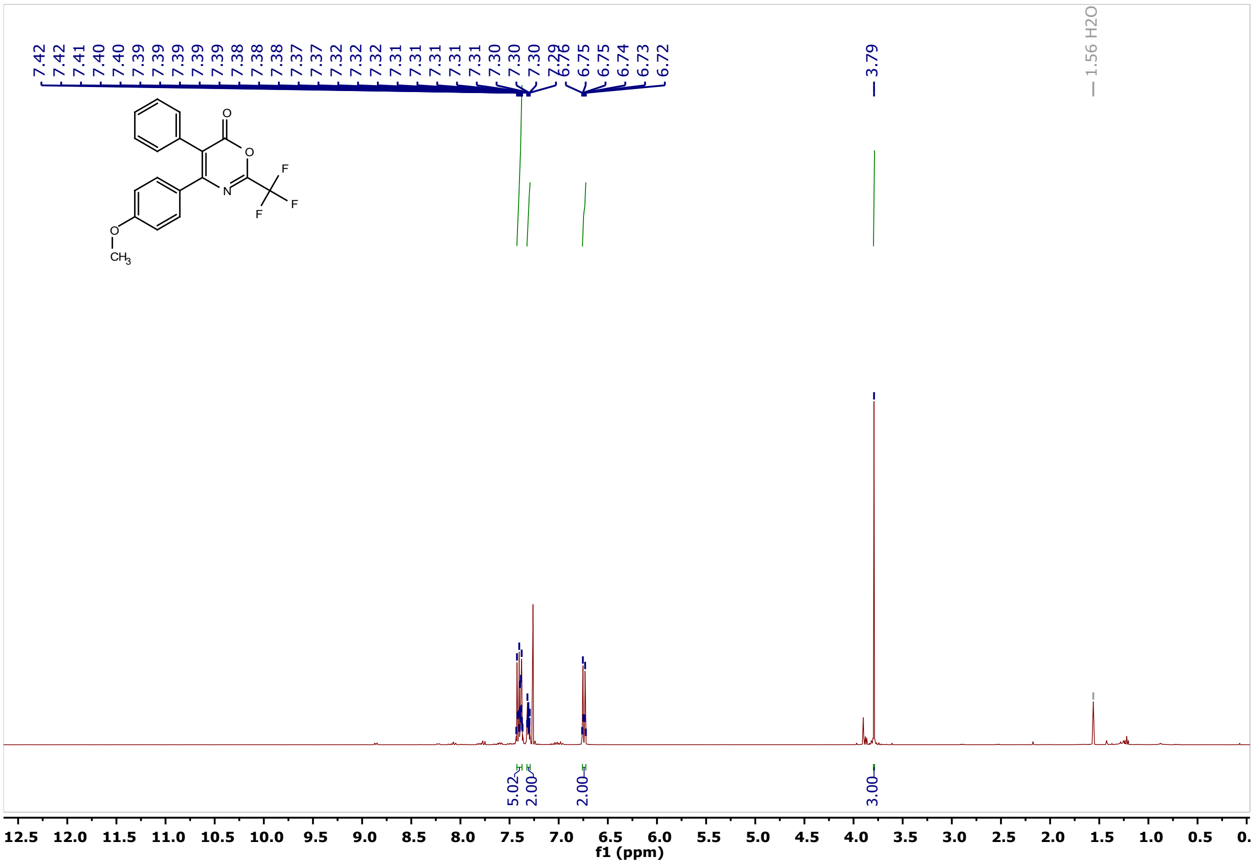

Figure S113. ^{13}C NMR spectrum of **6a** (101 MHz; $CDCl_3$)

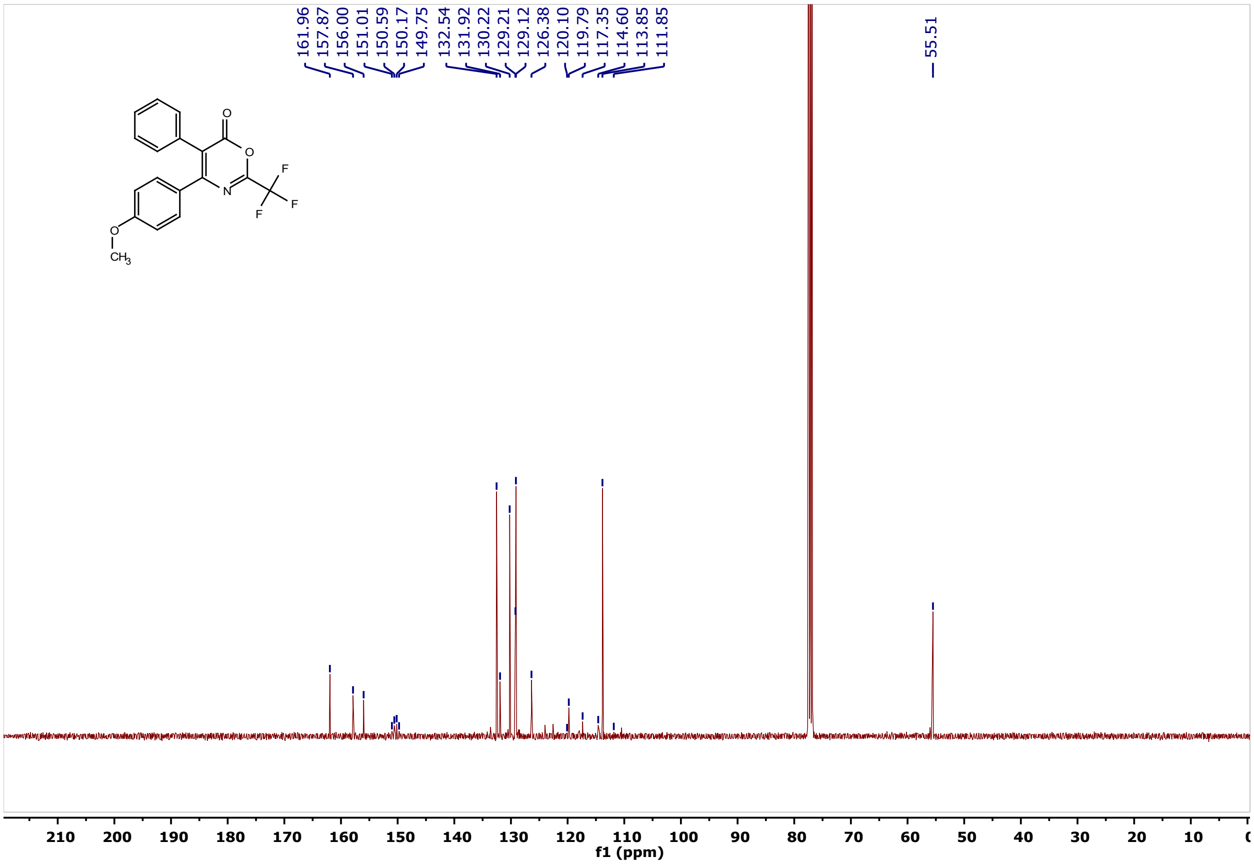

Figure S114. ^{19}F NMR spectrum of **6a** (377 MHz; $CDCl_3$)

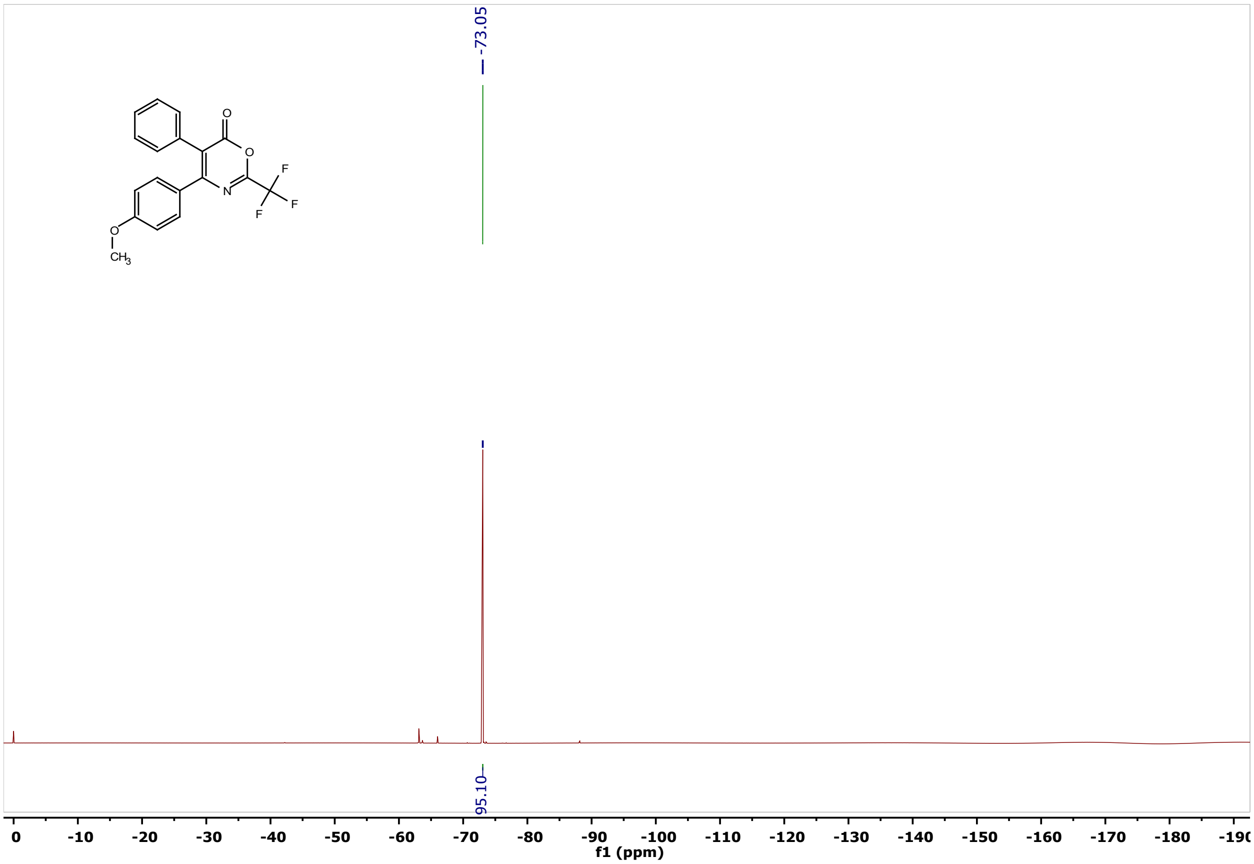

Figure S115. ^{1}H NMR spectrum of **6b** (401 MHz; $CDCl_3$)

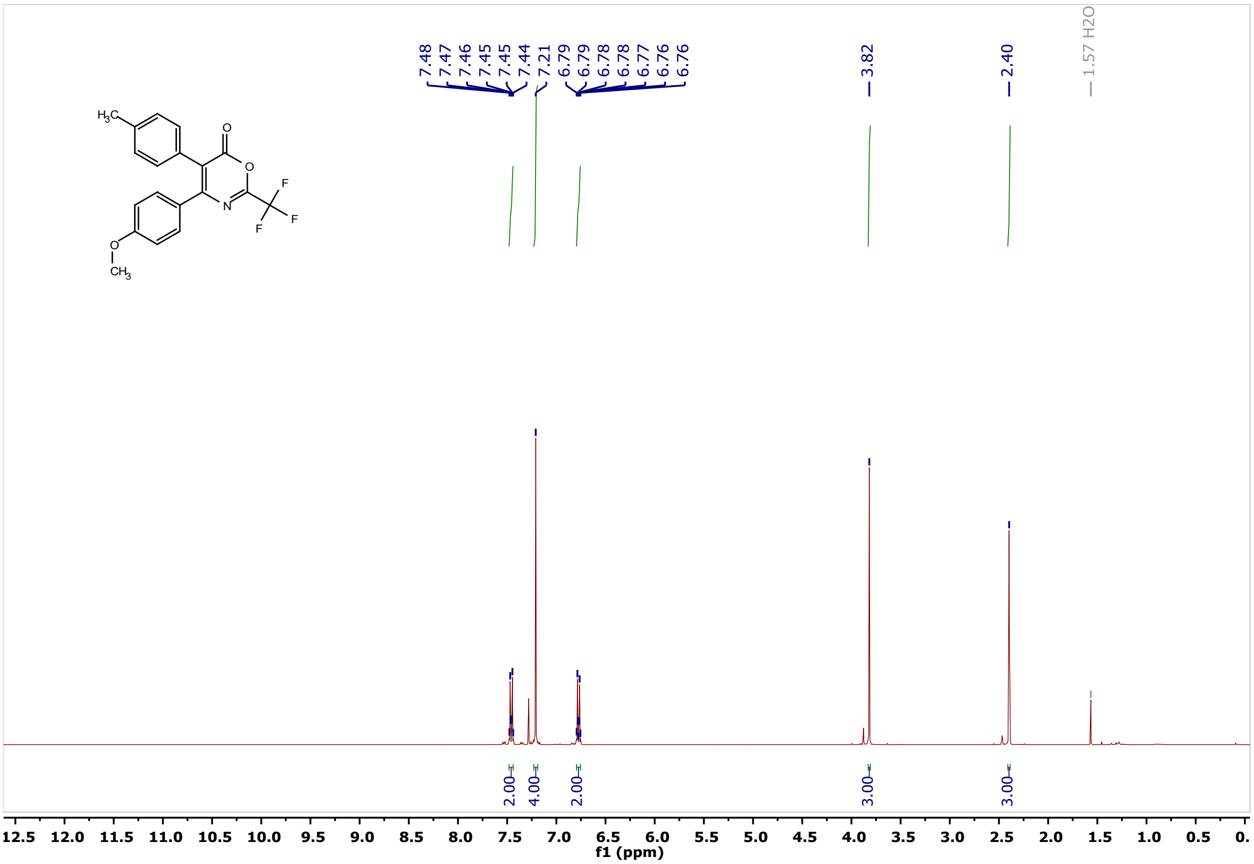

Figure S116. ^{13}C NMR spectrum of **6b** (101 MHz; $CDCl_3$)

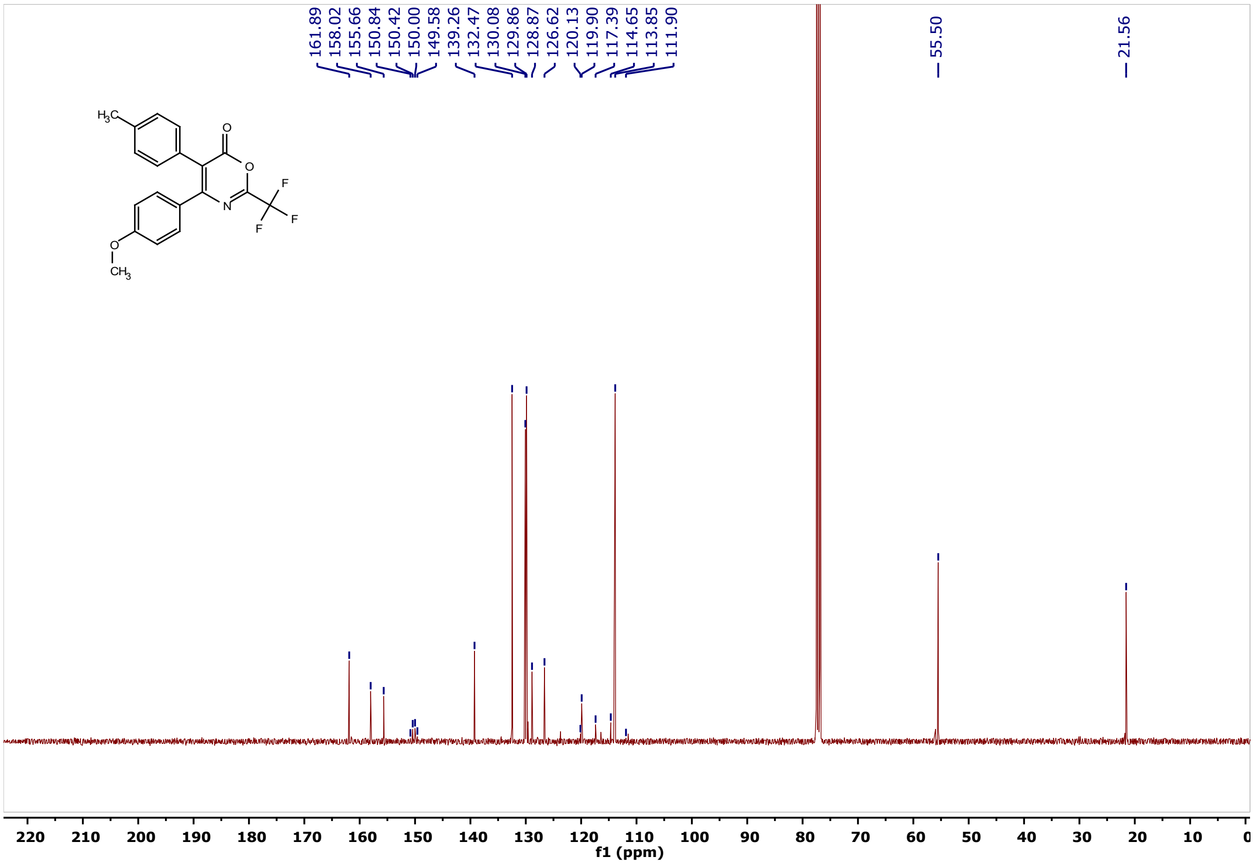

Figure S117. ^{19}F NMR spectrum of **6b** (377 MHz; $CDCl_3$)

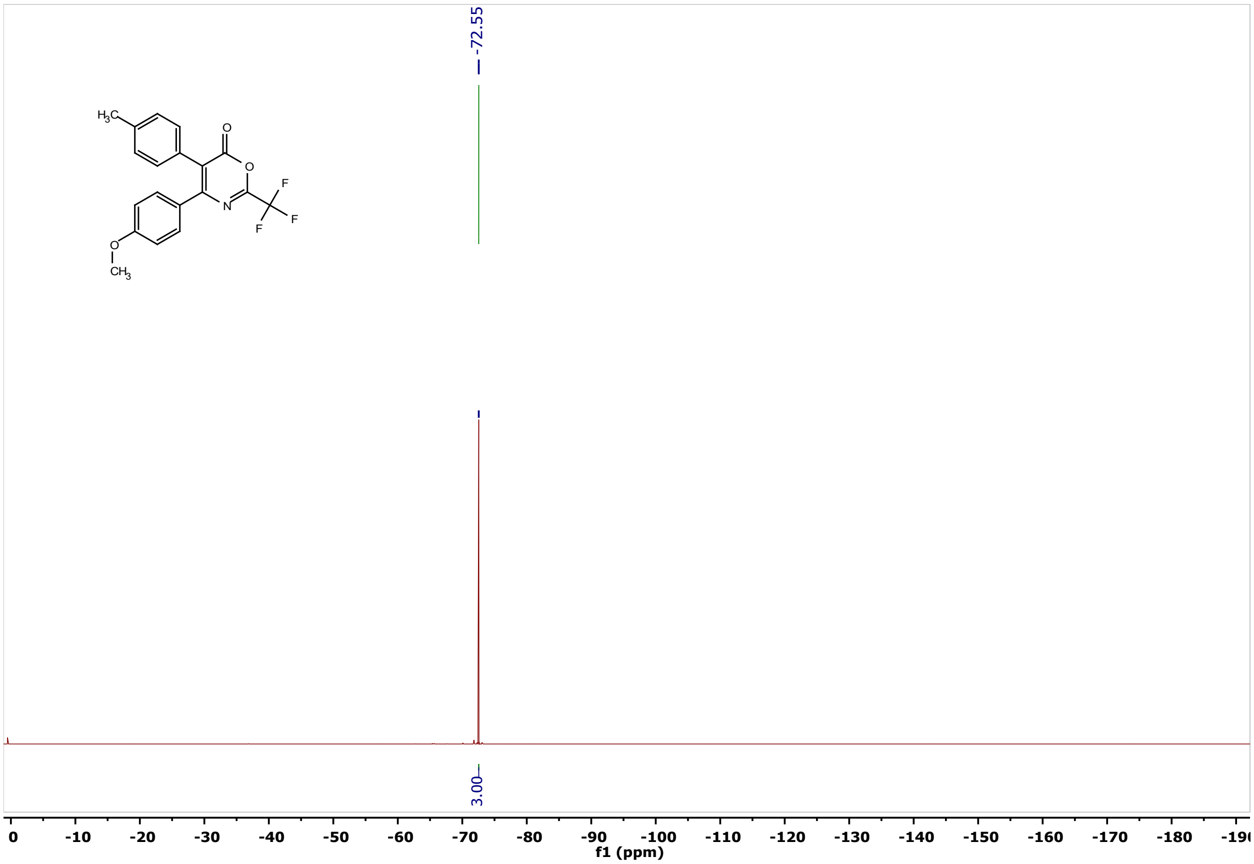

Figure S118. ^1H NMR spectrum of **6c** (401 MHz; $CDCl_3$)

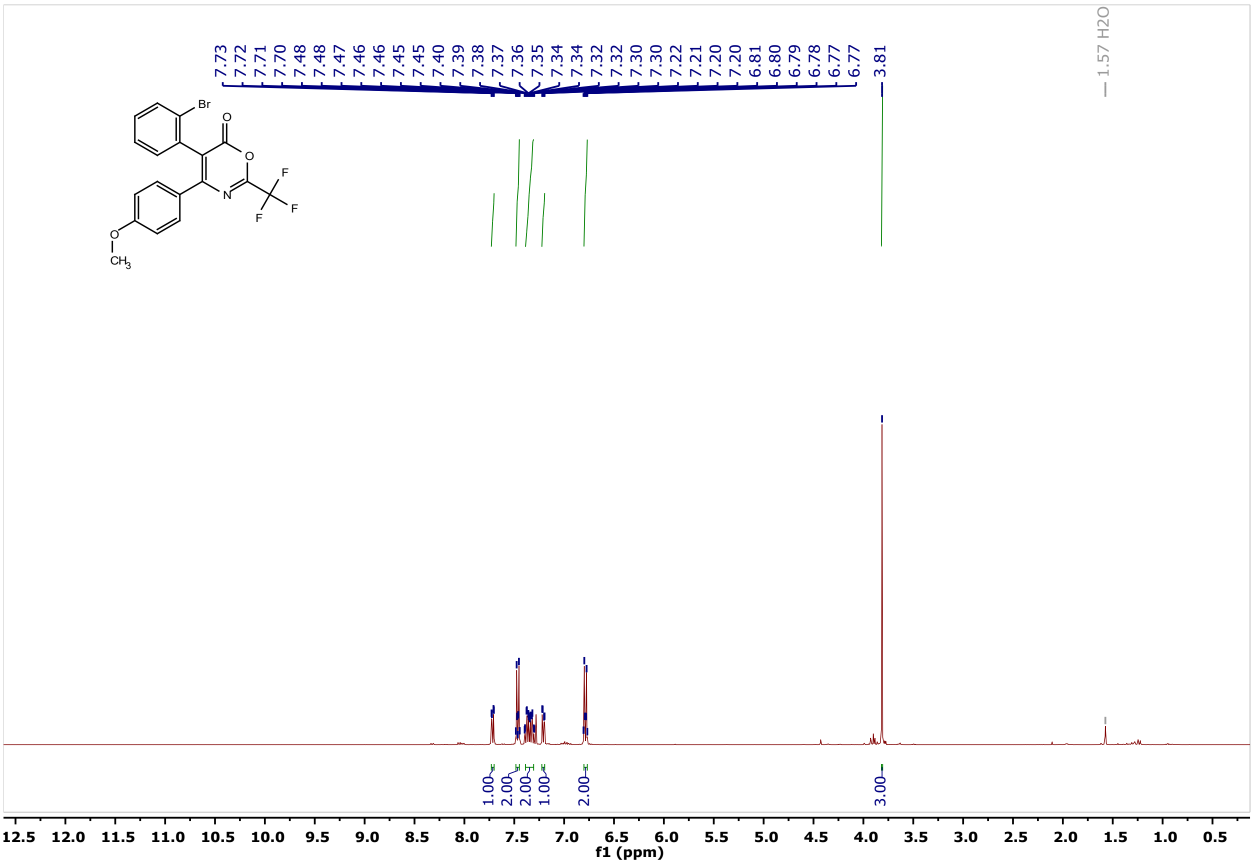

Figure S119. ^{13}C NMR spectrum of **6c** (101 MHz; $CDCl_3$)

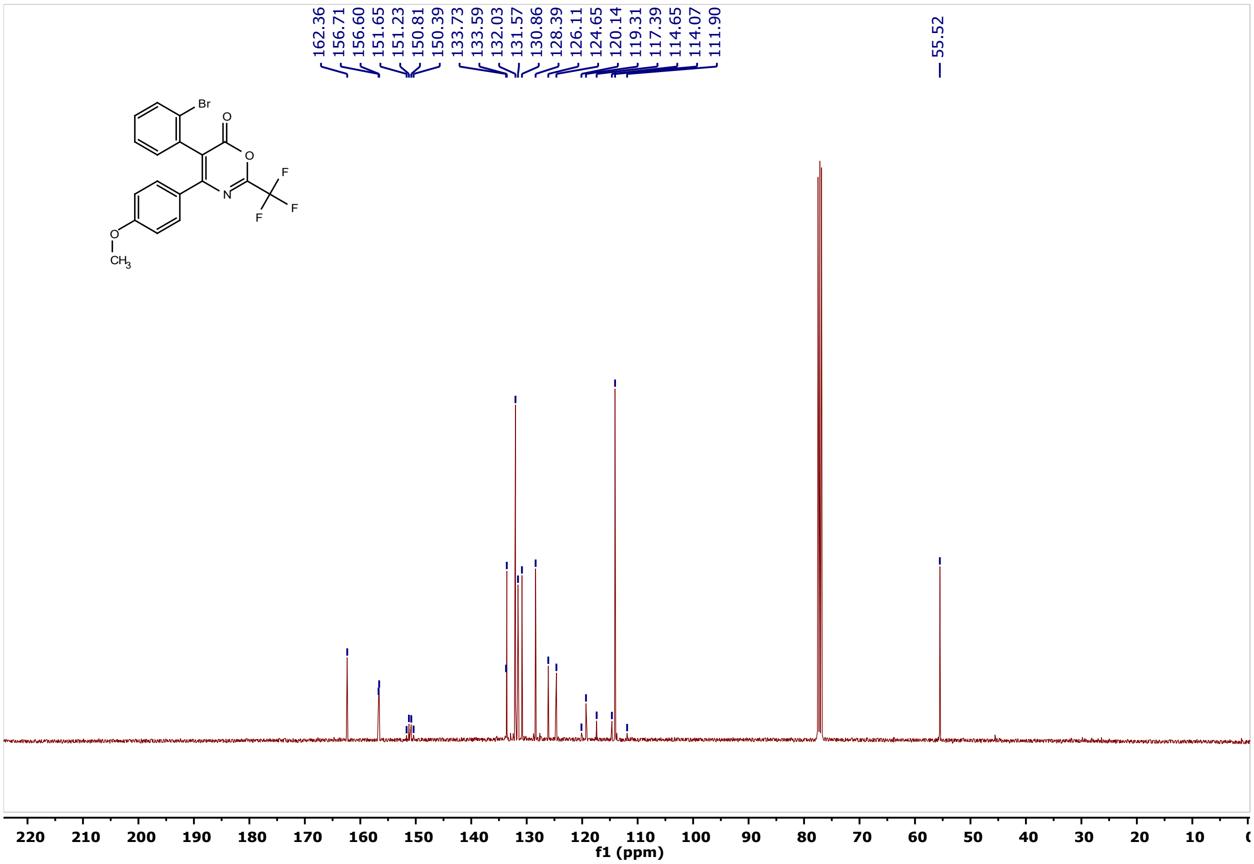

Figure S120. ^{19}F NMR spectrum of **6c** (377 MHz; $CDCl_3$)

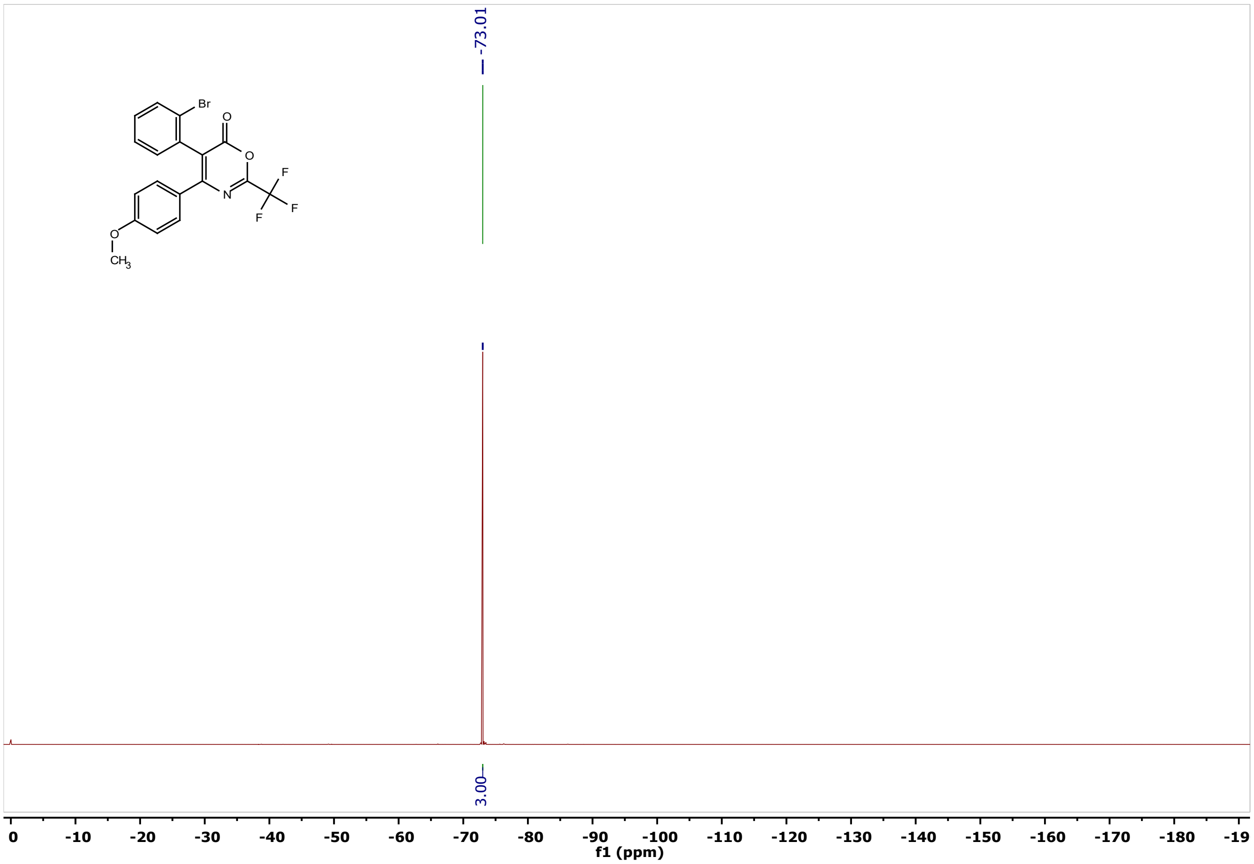

Figure S121. ^{1}H NMR spectrum of **6d** (401 MHz; $CDCl_3$)

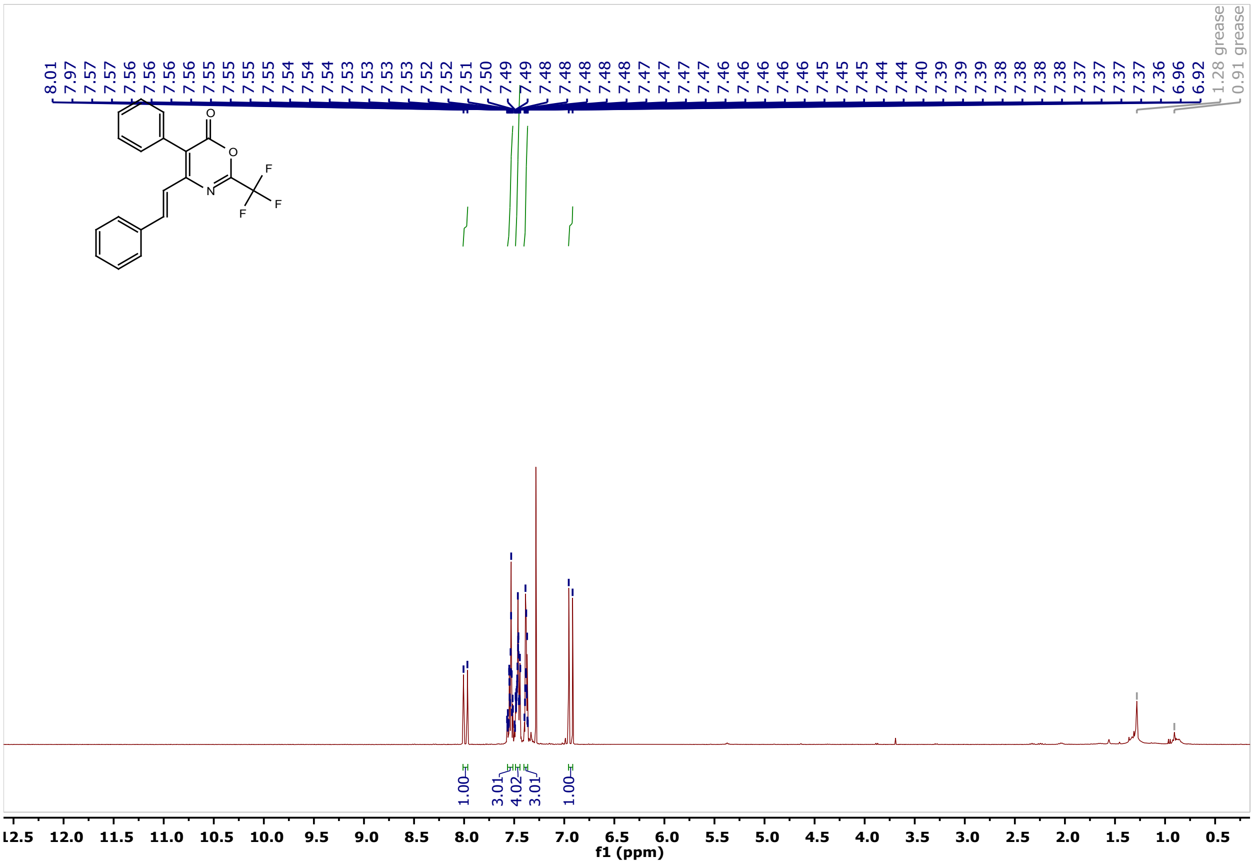

Figure S122. ^{13}C NMR spectrum of **6d** (101 MHz; $CDCl_3$)

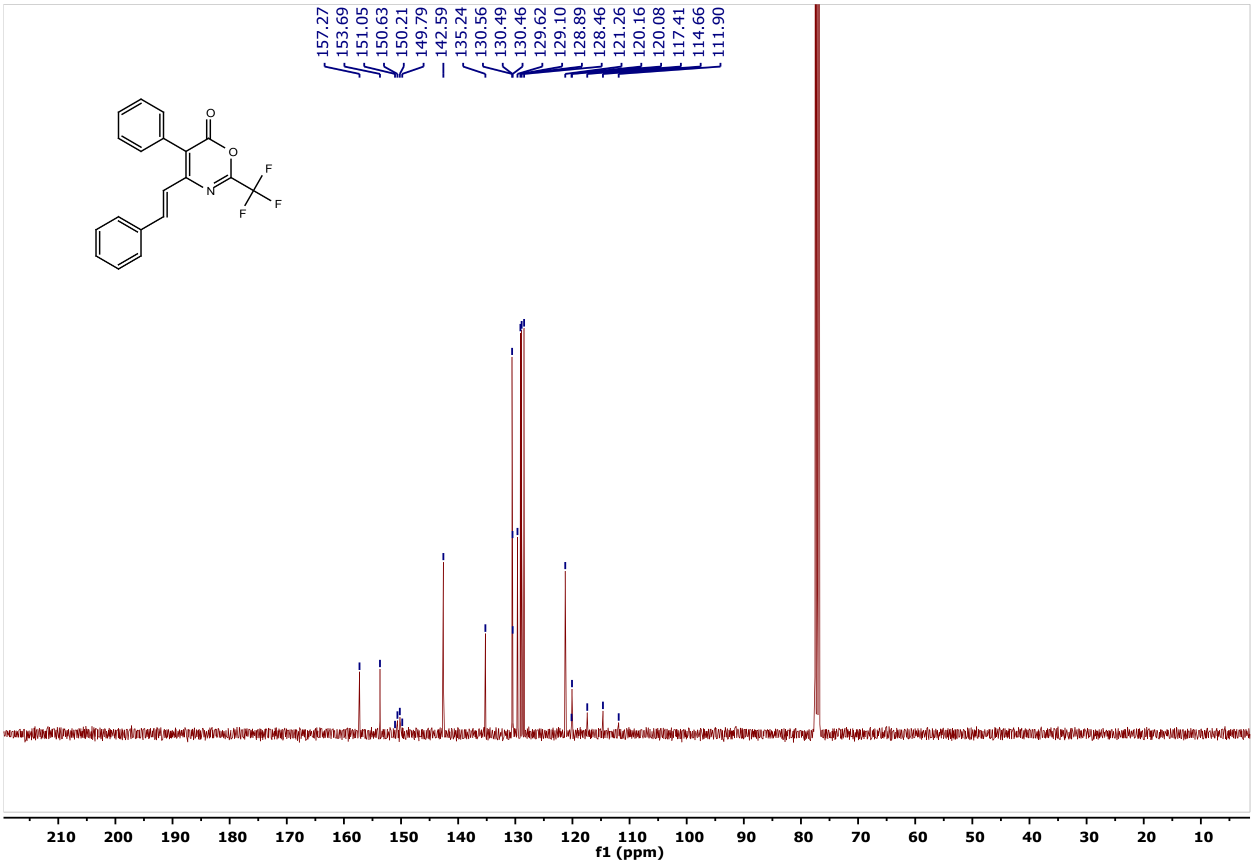

Figure S123. ^{19}F NMR spectrum of **6d** (377 MHz; $CDCl_3$)

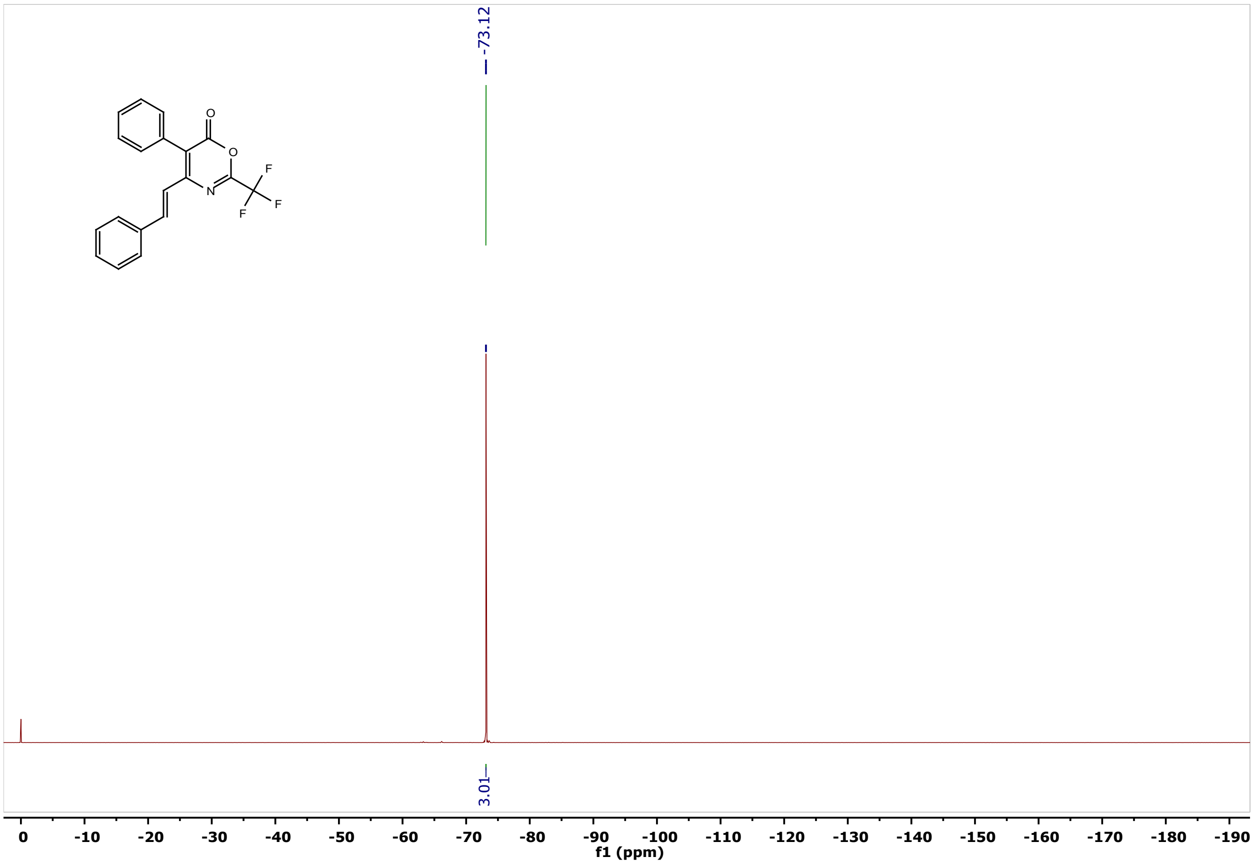

Figure S124. ^{1}H NMR spectrum of **6e** (401 MHz; $CDCl_3$)

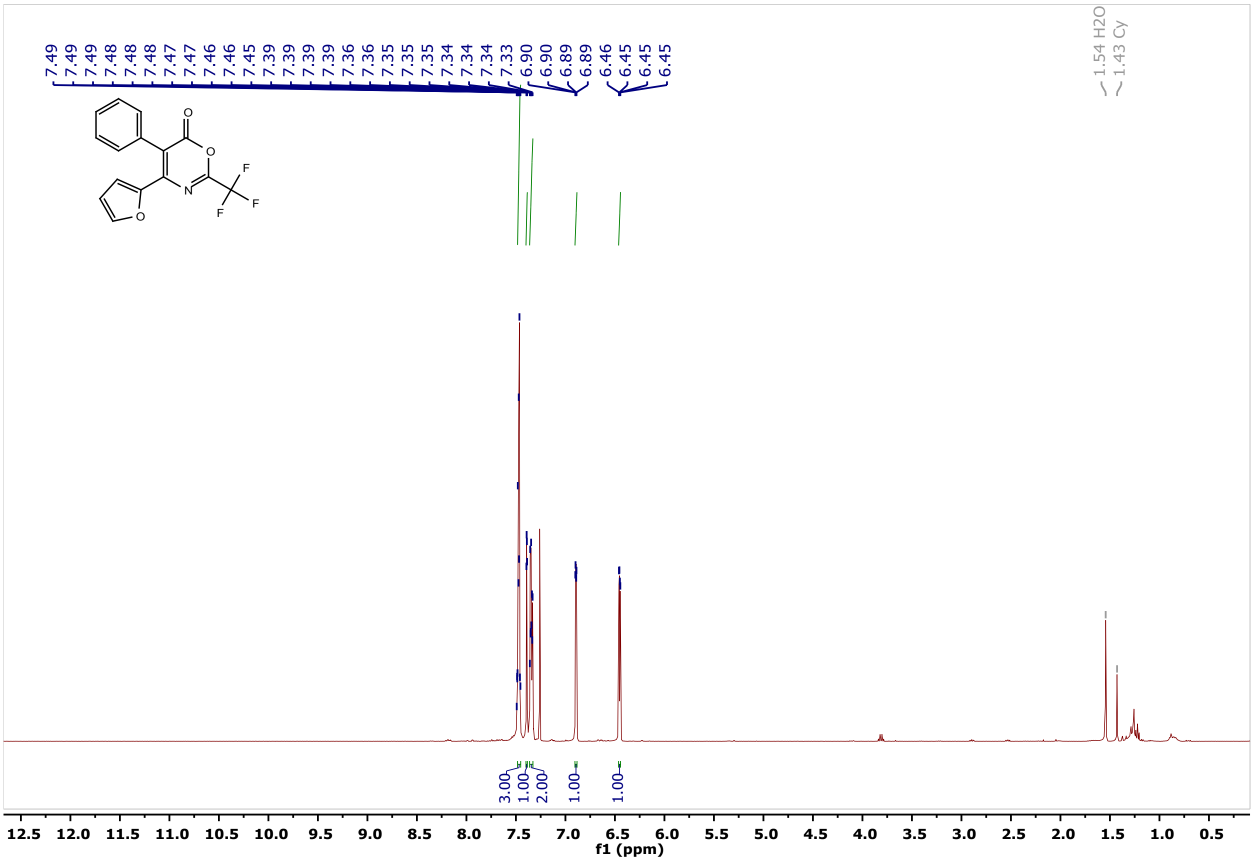

Figure S125. ^{13}C NMR spectrum of **6e** (101 MHz; $CDCl_3$)

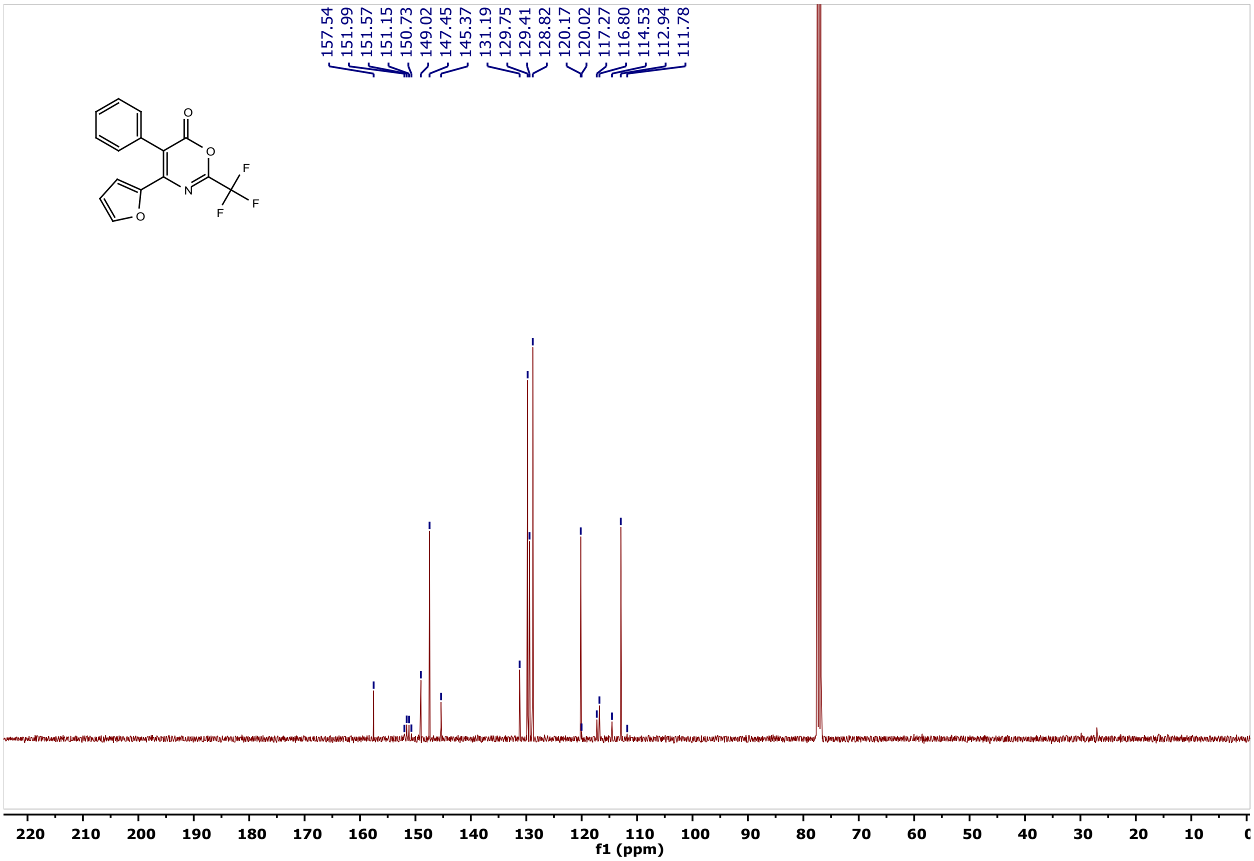

Figure S126. ^{19}F NMR spectrum of **6e** (377 MHz; $CDCl_3$)

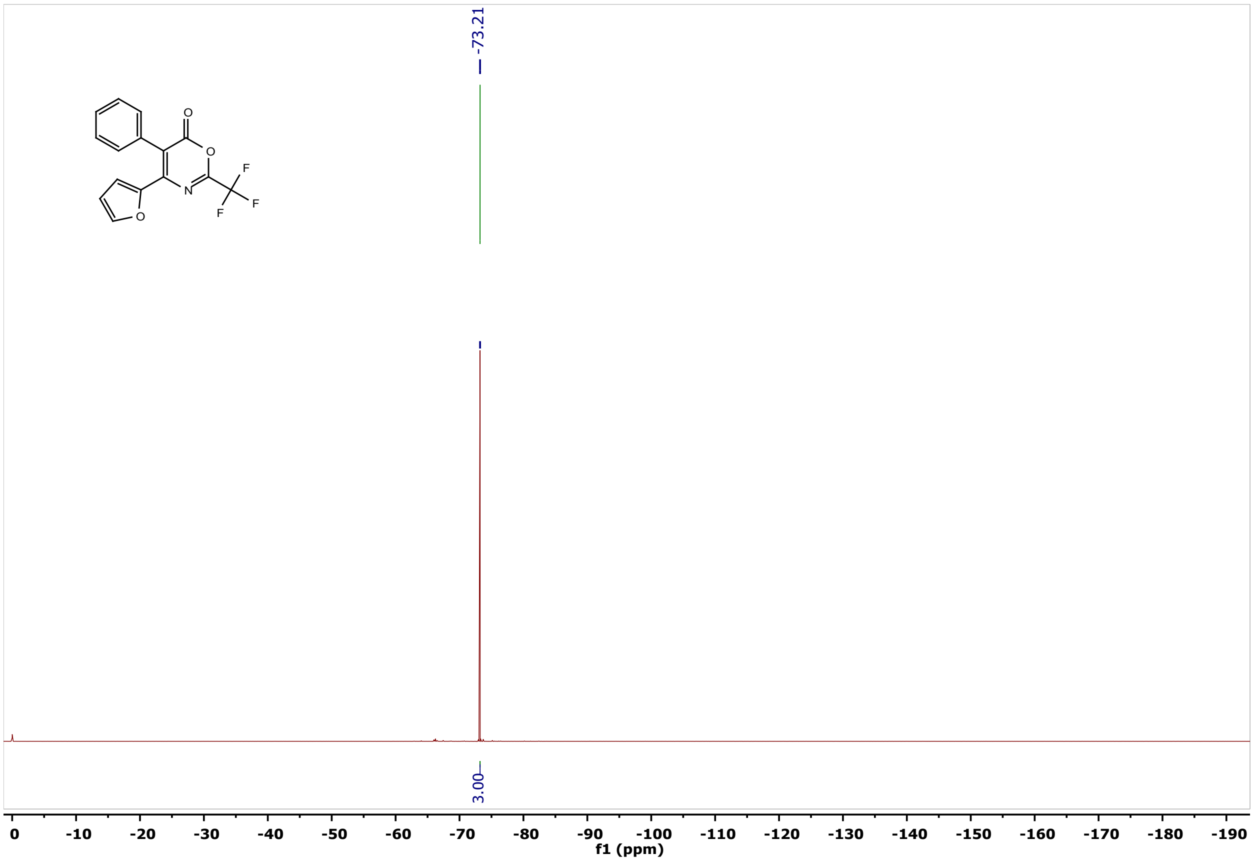

Figure S127. ^{1}H NMR spectrum of **6f** (401 MHz; $CDCl_3$)

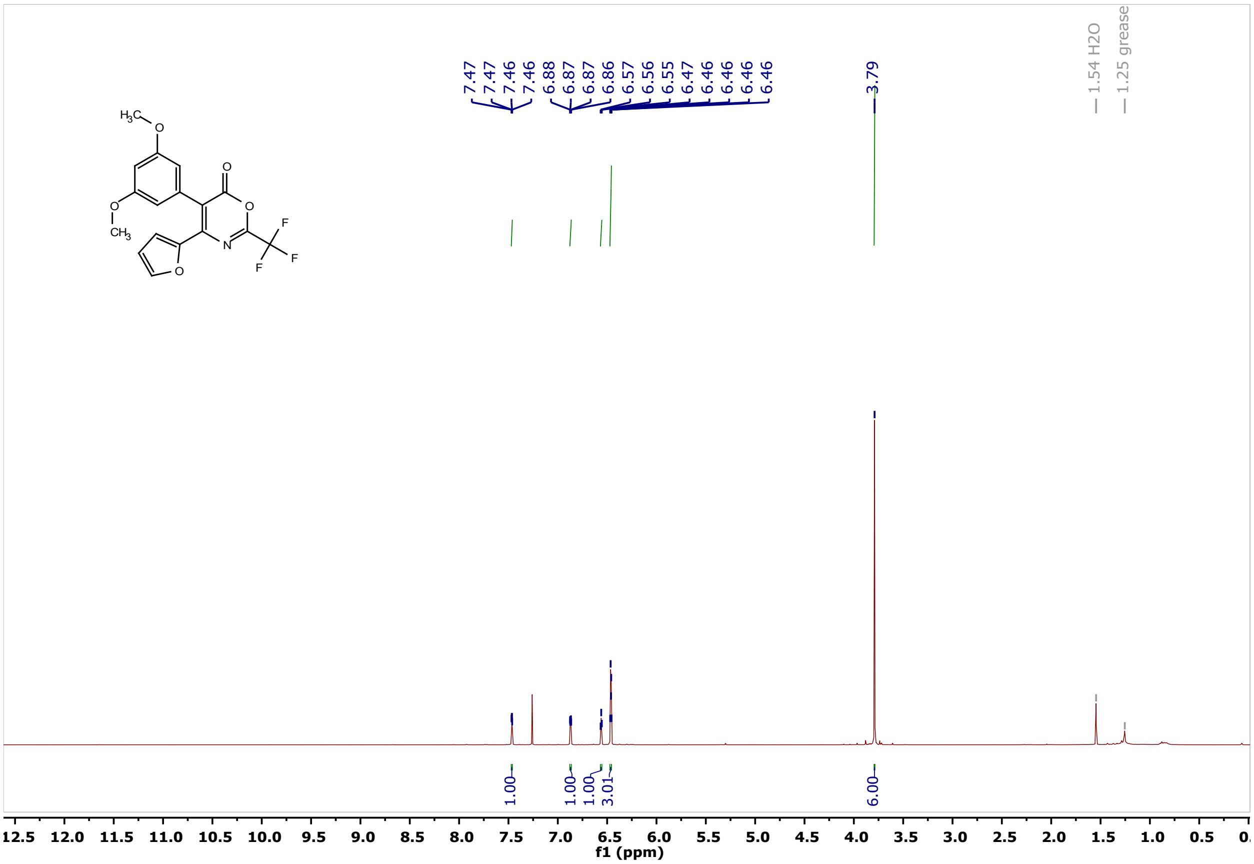

Figure S128. ^{13}C NMR spectrum of **6f** (101 MHz; $CDCl_3$)

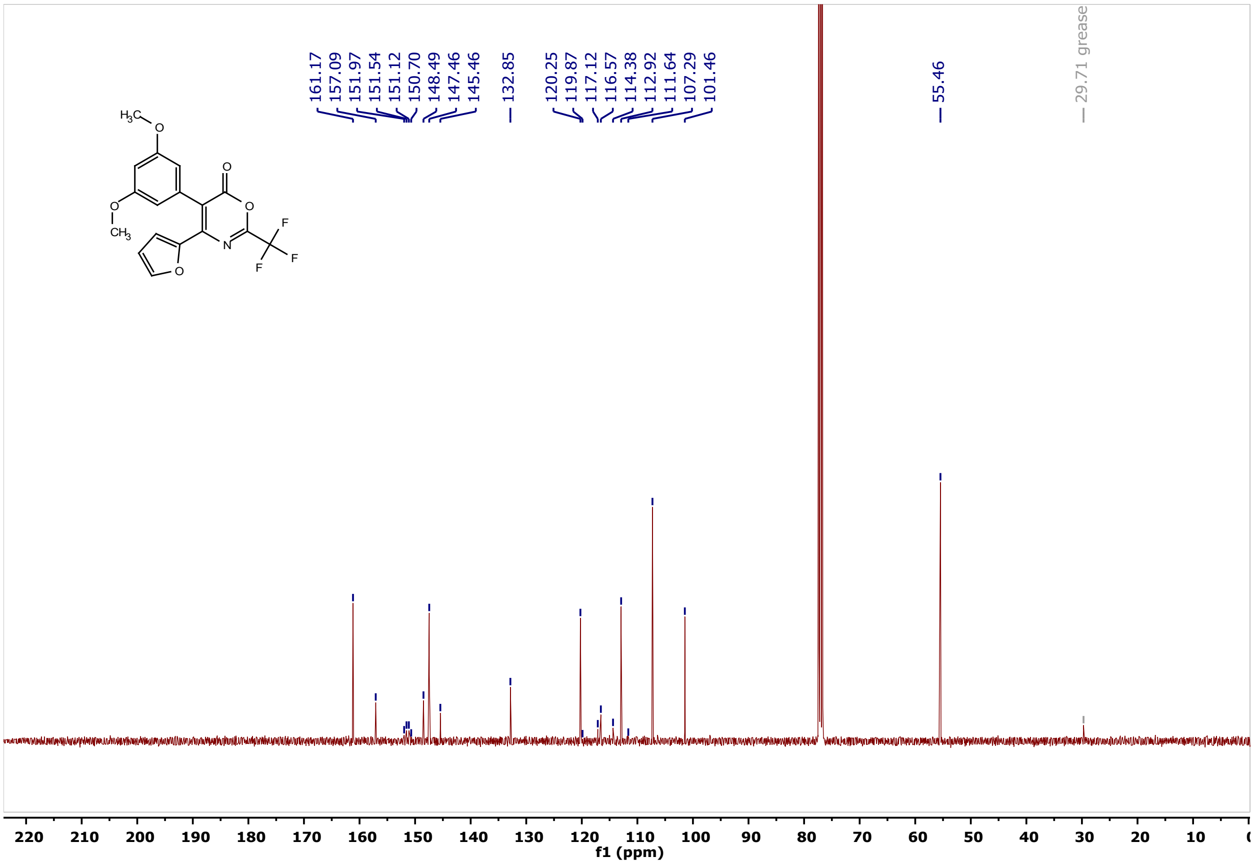

Figure S129. ^{19}F NMR spectrum of **6f** (377 MHz; $CDCl_3$)

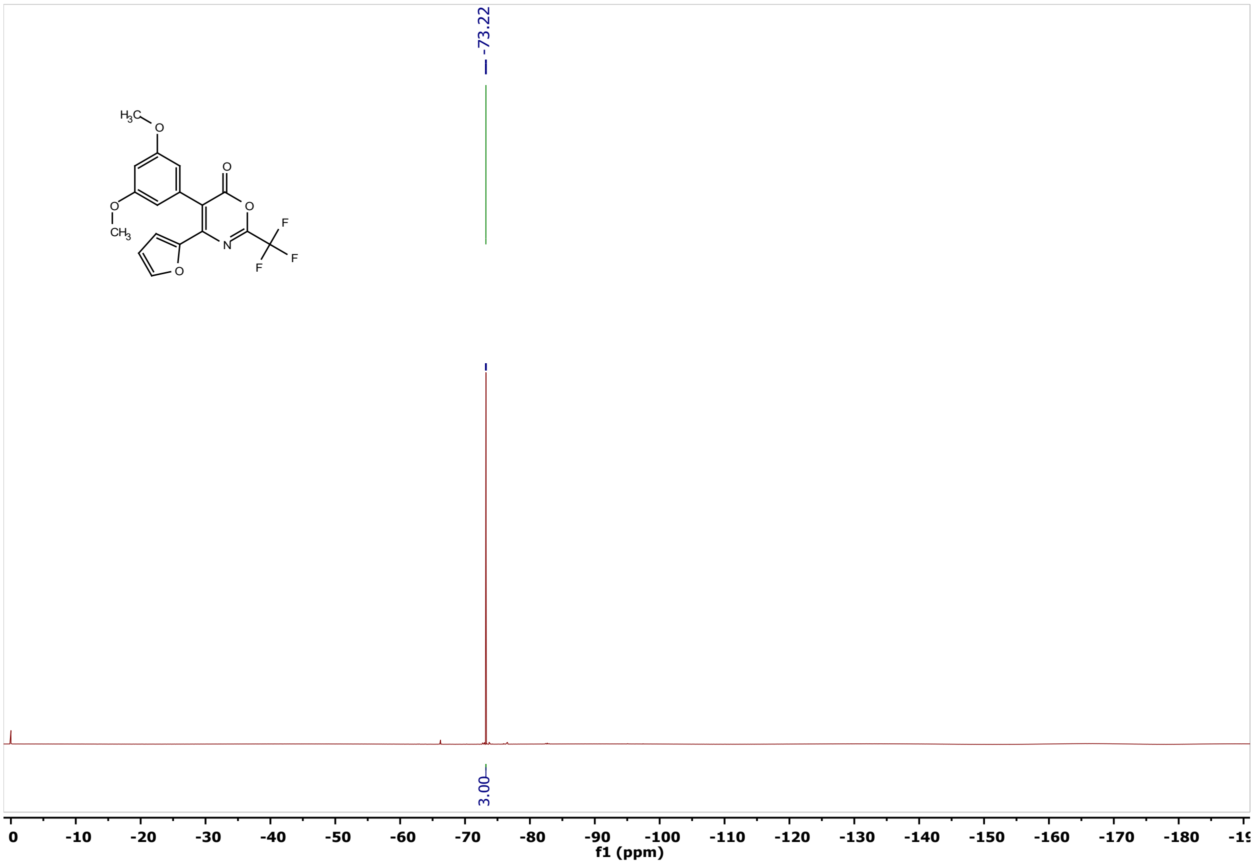

Figure S130. ^{1}H NMR spectrum of **6g** (401 MHz; $CDCl_3$)

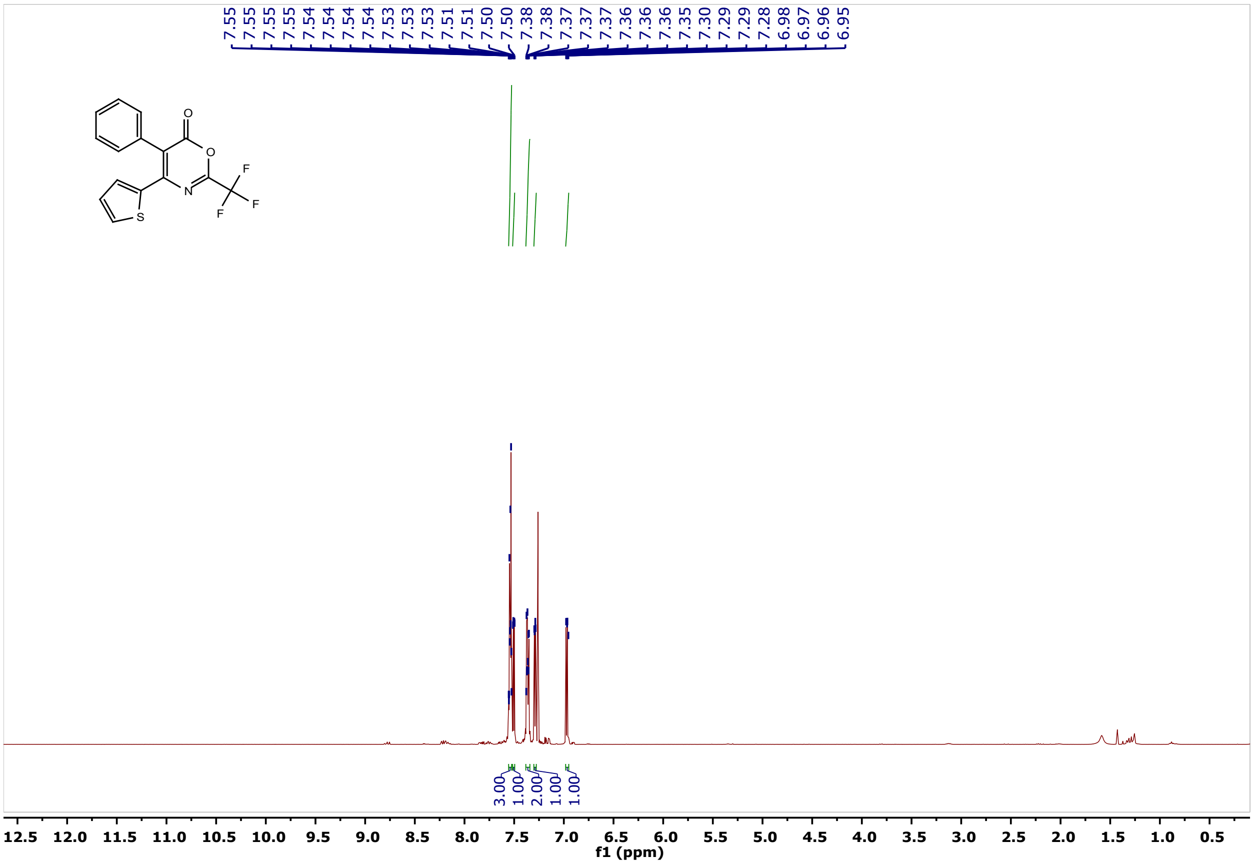

Figure S131. ^{13}C NMR spectrum of **6g** (101 MHz; $CDCl_3$)

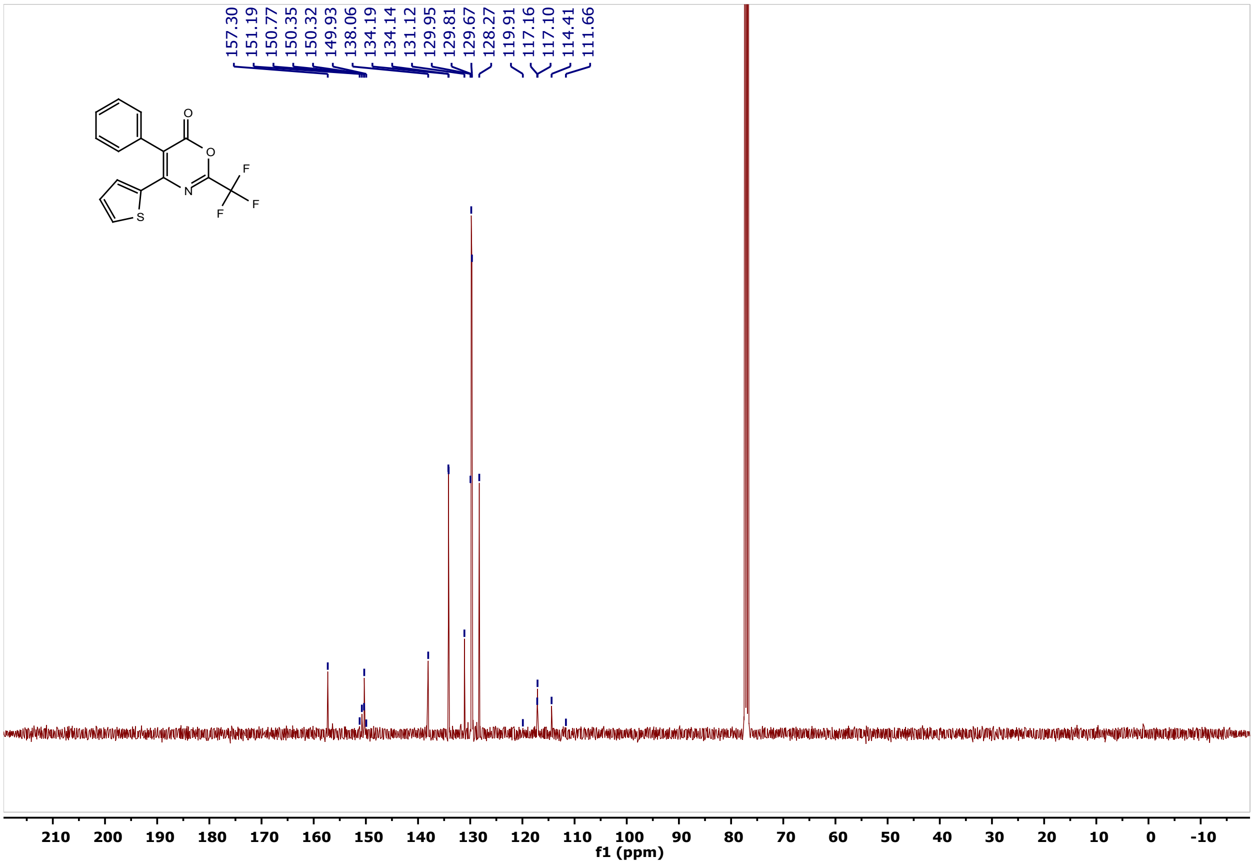

Figure S132. ^{19}F NMR spectrum of **6g** (377 MHz; $CDCl_3$)

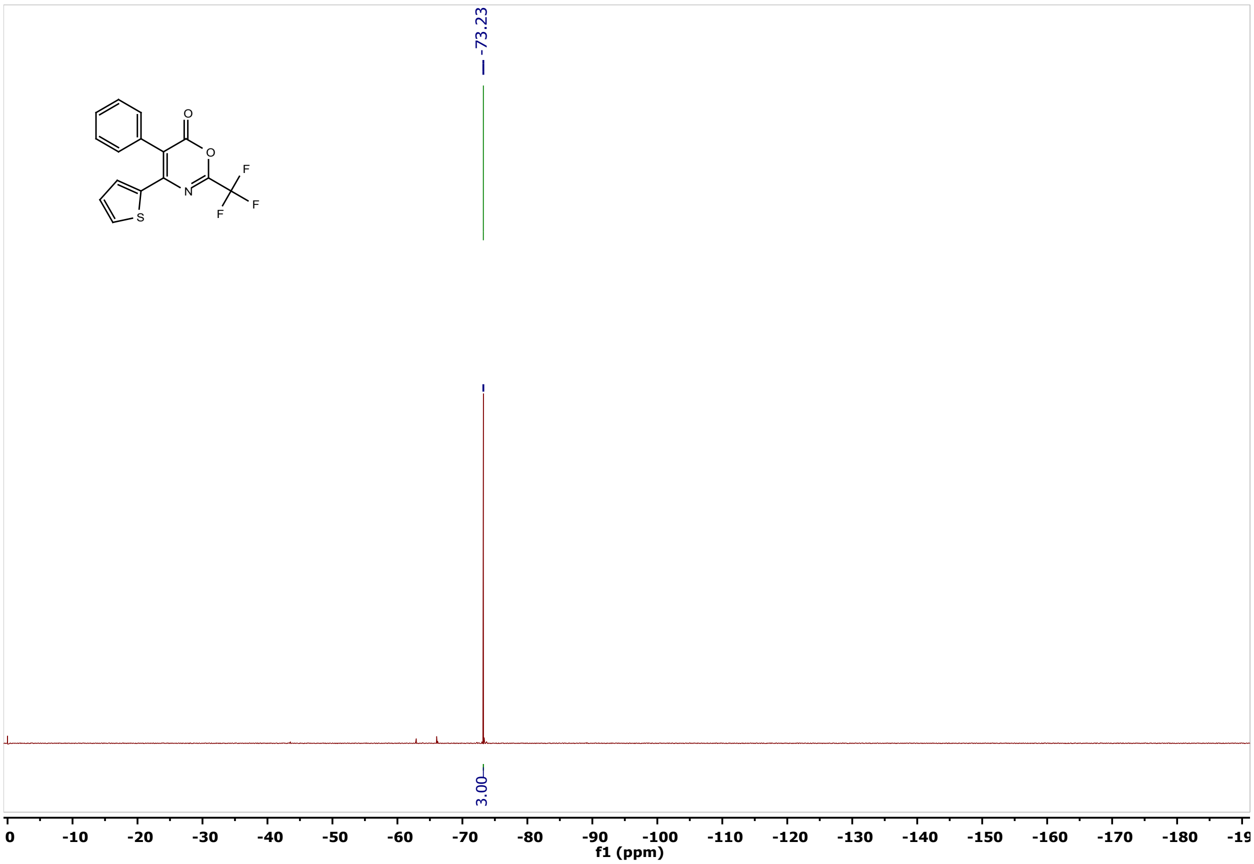

Figure S133. ^{1}H NMR spectrum of **6h** (401 MHz; $CDCl_3$)

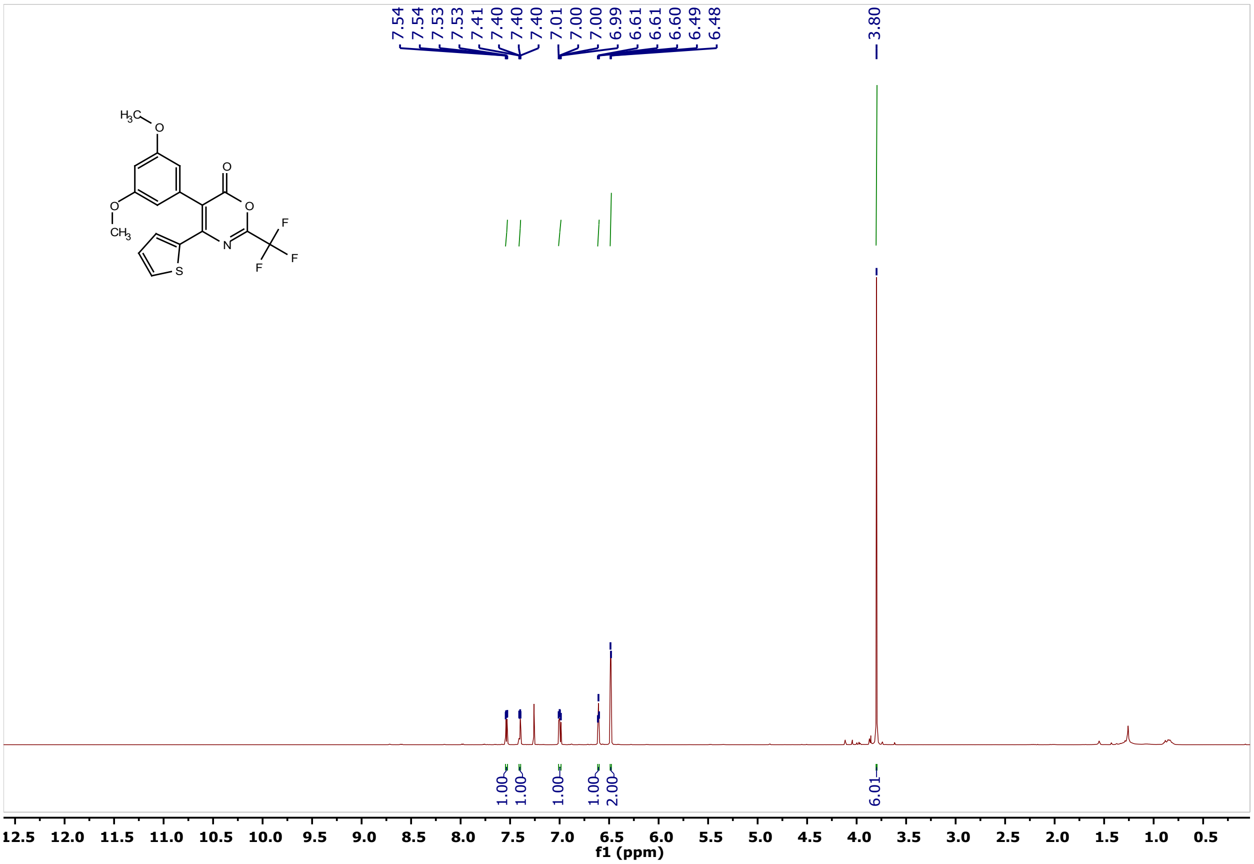

Figure S134. ^{13}C NMR spectrum of **6h** (101 MHz; $CDCl_3$)

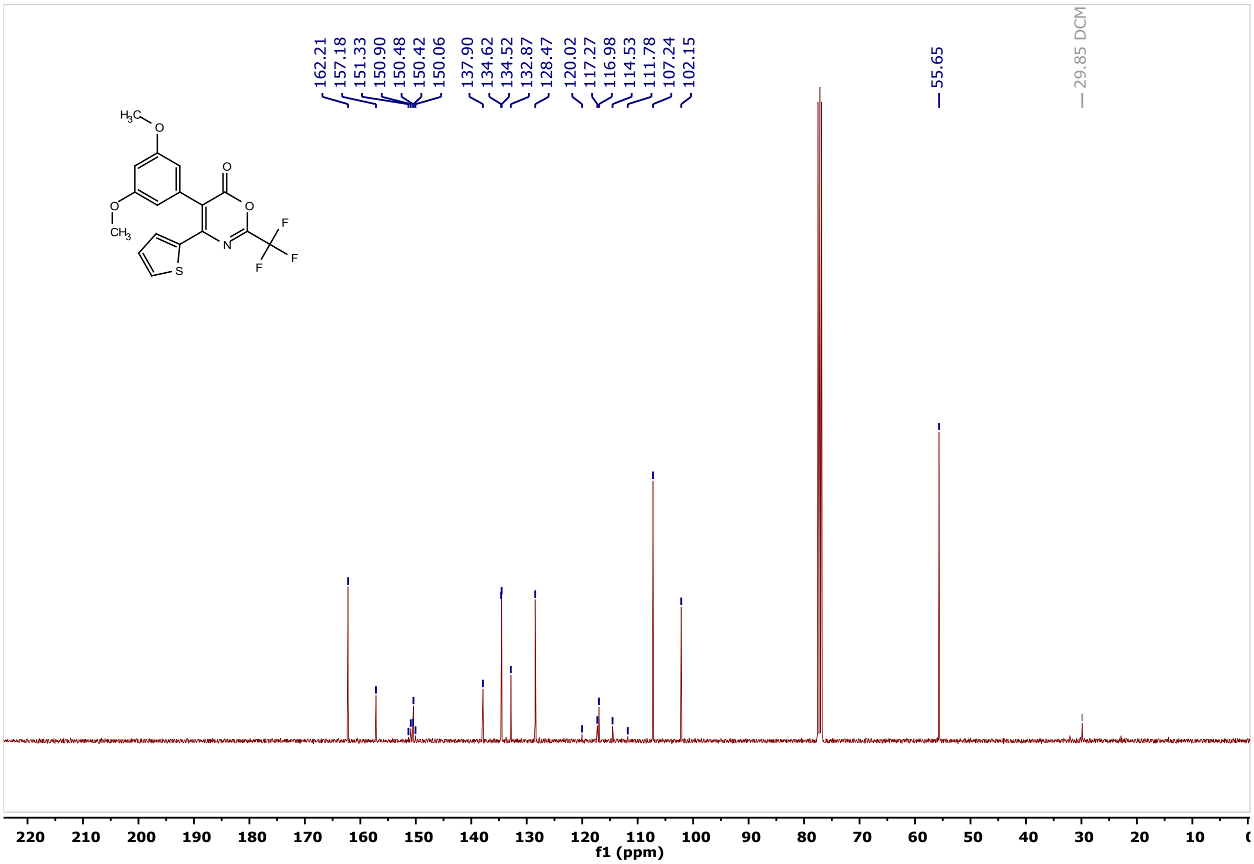

Figure S135. ^{19}F NMR spectrum of **6h** (377 MHz; $CDCl_3$)

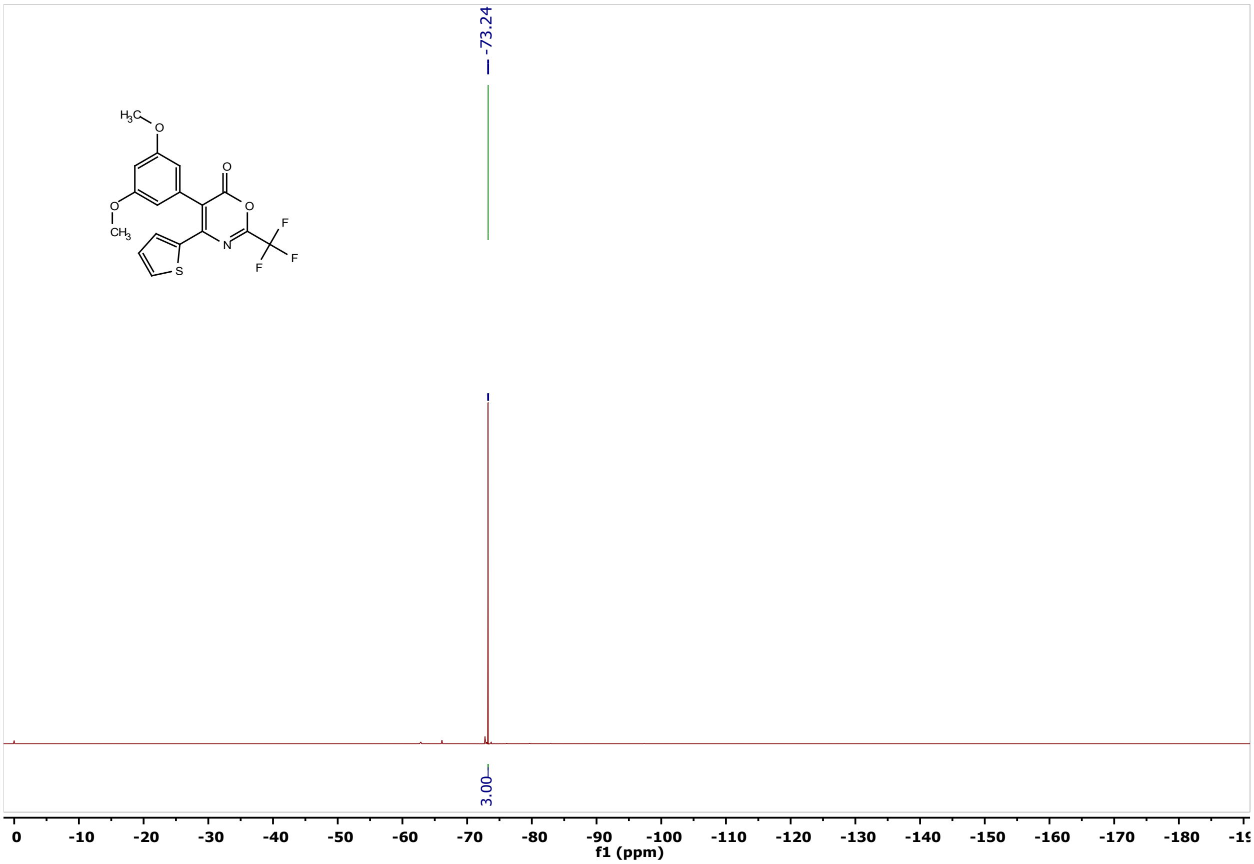

Figure S136. ^{1}H NMR spectrum of **6i** (401 MHz; $CDCl_3$)

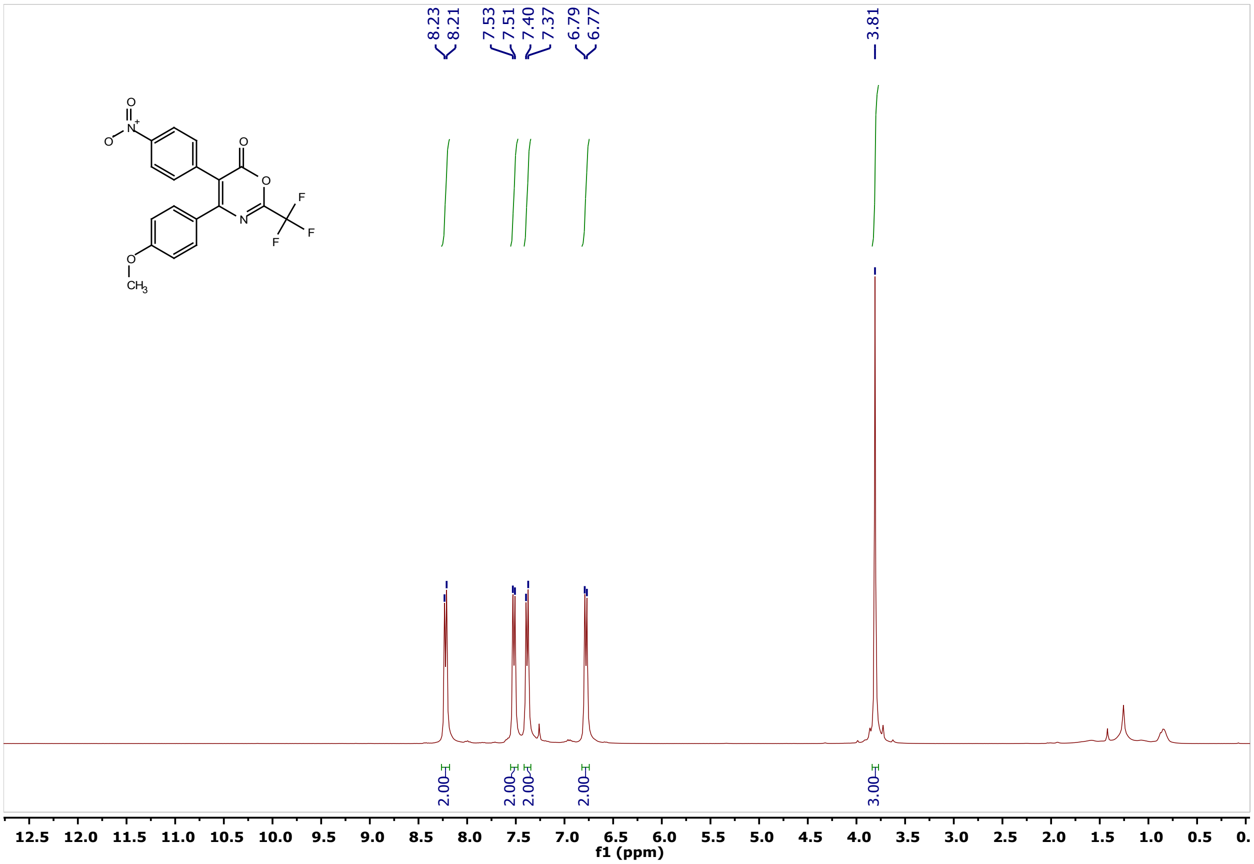

Figure S137. ^{13}C NMR spectrum of **6i** (101 MHz; $CDCl_3$)

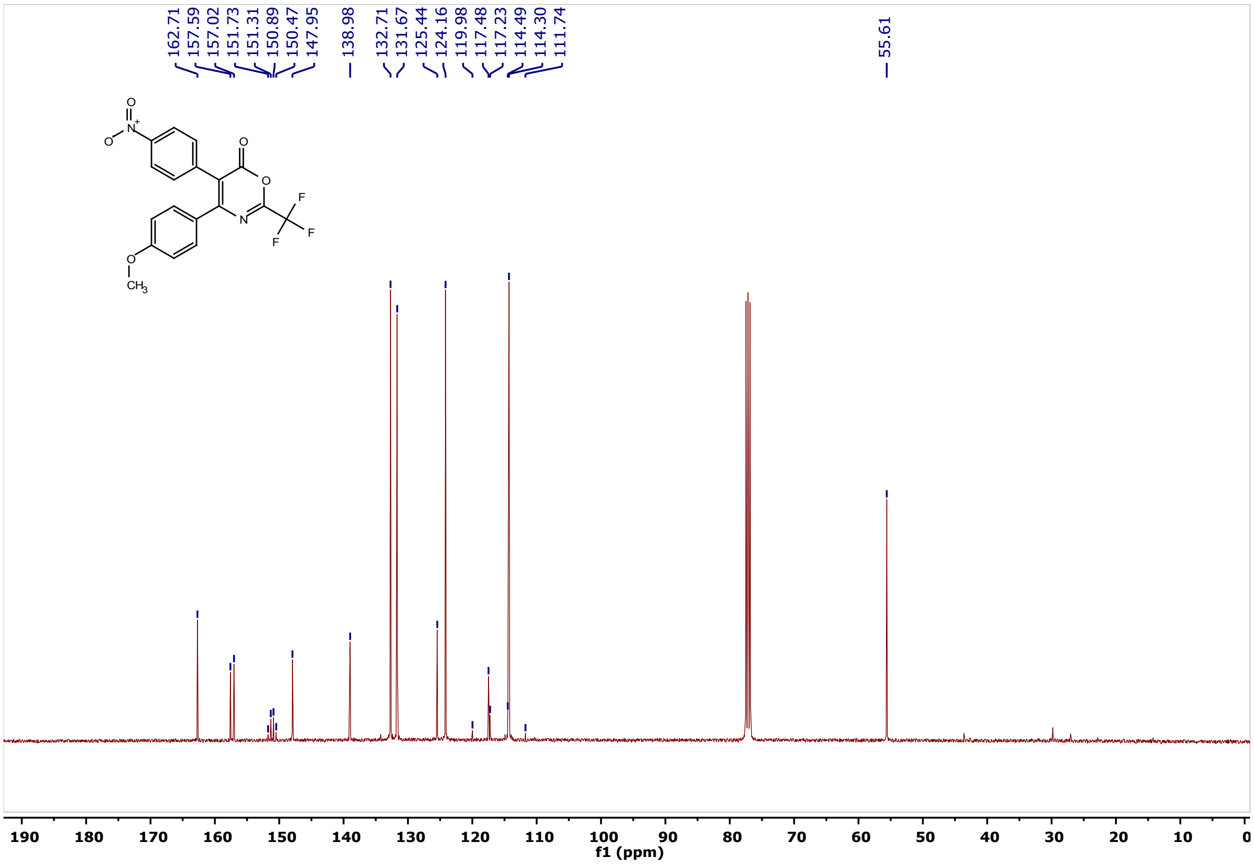

Figure S138. ^{19}F NMR spectrum of **6i** (377 MHz; $CDCl_3$)

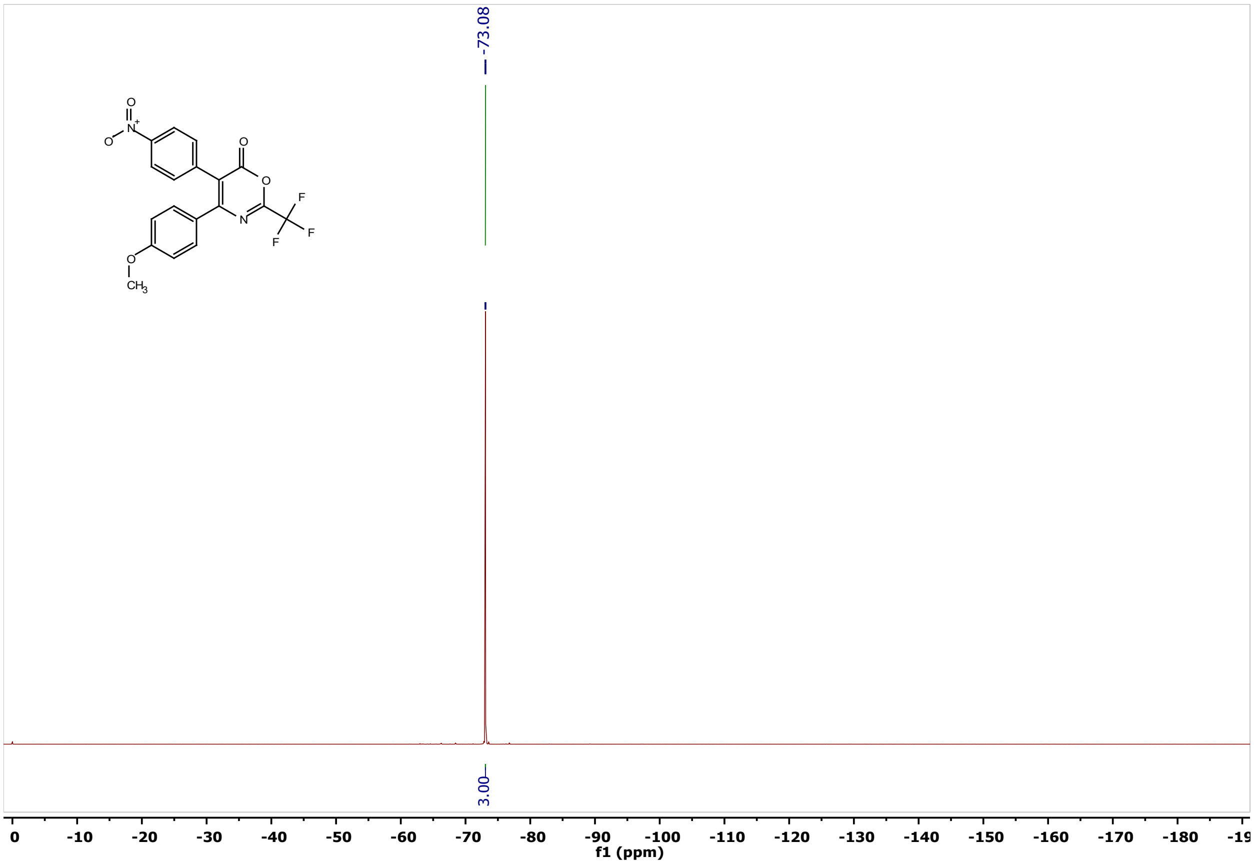

Supplement: RA-014-D4RA04794J-s001 [file RA-014-D4RA04794J-s001.pdf]
